# Oxa-Michael-based divergent synthesis of artificial glutamate analogs†

**DOI:** 10.1039/d2ra03744k

**Published:** 2022-08-10

**Authors:** Shuntaro Tsukamoto, Oriel Hlokoane, Kei Miyako, Raku Irie, Ryuichi Sakai, Masato Oikawa

**Affiliations:** Graduate School of Nanobioscience, Yokohama City University Seto 22-2, Kanazawa-ku Yokohama 236-0027 Japan moikawa@yokohama-cu.ac.jp; Department of Pharmacy, National University of Lesotho P.O. Roma 180 Maseru Lesotho; Faculty of Fisheries Sciences, Hokkaido University Hakodate 041-8611 Japan

## Abstract

Herein we report stereoselective generation of two skeletons, 1,3-dioxane and tetrahydropyranol, by oxa-Michael reaction as the key reaction from δ-hydroxyenone. The construction of the 1,3-dioxane skeleton, achieved through hemiacetal formation followed by oxa-Michael reaction from δ-hydroxyenone, was exploited to access structurally diverse heterotricyclic artificial glutamate analogs. On the other hand, formation of a novel tetrahydro-2*H*-pyranol skeleton was accomplished by the inverse reaction order: oxa-Michael reaction followed by hemiacetal formation. Thus, this study succeeded in showing that structural diversity in a compound collection can be acquired by interchanging the order of just two reactions. Among the skeletally diverse, heterotricyclic artificial glutamate analogs synthesized in this study, a neuronally active compound named TKM-50 was discovered in the mice *in vivo* assay.

## Abstract 2

By interchanging the order of reactions, two types of skeletons were created and a neuroactive artificial glutamate analog was developed.

## Graphical abstract

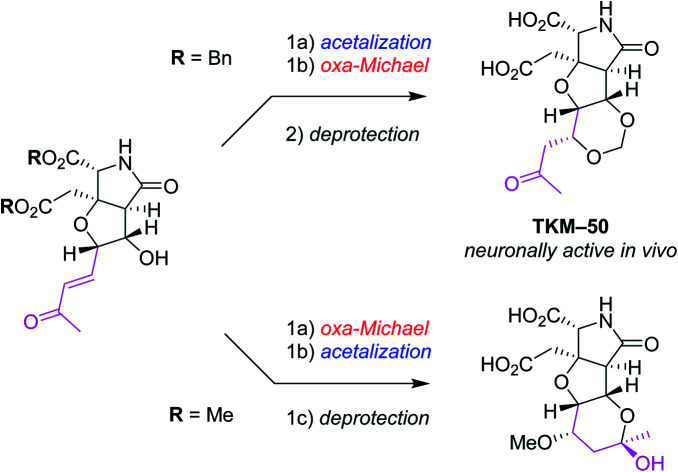

Ionotropic glutamate receptors (iGluRs) mediate the majority of the excitatory neurotransmissions such as learning, memory, and nociception in the mammalian central nervous system (CNS).^1^ To study and control the function of iGluRs, specific glutamate analogs have been developed in natural product chemistry^2^ and in medicinal chemistry.^3,4^ IKM-159 (Fig. 1A) is an artificial glutamate analog designed and developed based on dysiherbaine^5,6^ and kainic acid^7^ in our laboratories as an antagonist selective to (*S*)-2-amino-3-(3-hydroxy-5-methyl-4-isoxazolyl)propionic acid (AMPA)-type iGluR.^8–12^ The AMPA receptor consists of four subunits: GluA1, GluA2, GluA3, and GluA4.^3^*In vitro*, IKM-159 selectively inhibits the GluA1/GluA2 heterodimer and GluA4 homodimer.^10^*In vivo*, IKM-159 inhibits voluntary action of mice for 50 min to several hours upon intracerebroventricular injection. The potency and selectivity of IKM-159 are, however, not very satisfactory to selectively modulate the function of AMPA-type iGluR. As an attempt to improve the biological profiles of IKM-159, we have been studying its structural modification.

**Fig. 1:**
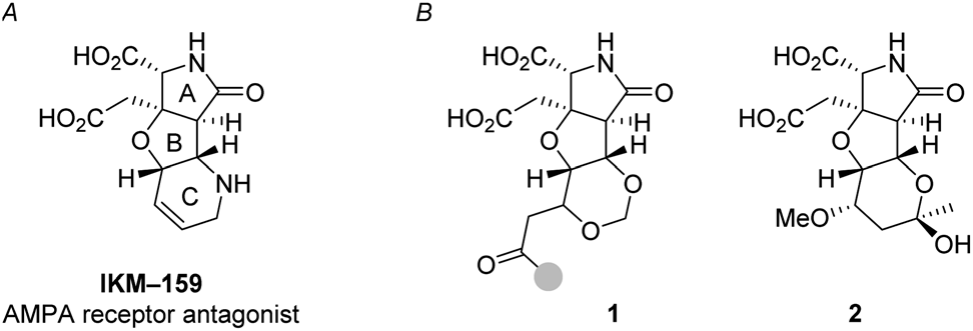
Background (A) and summary (B) of this work. (A) AMPA-type iGluR antagonist IKM-159. (B) Artificial glutamate analogs 1 and 2, generated by oxa-Michael-based transformations (this work). The gray circle in 1 denotes the position for the structural diversity.

From the first-generation studies on structure–activity relationships (SARs) of IKM-159, it had been shown that the ring size and the heteroatom of the C-ring were important for neuroactivity of IKM-159.^10,13^ We then studied the second-generation SAR on the oxa analogs generated by a Prins-Ritter three-component coupling strategy, although all analogs were found to lose the original neuronal activity of IKM-159.^14^ Herein, we report our continuous effort along this line employing the homoallylic alcohol such as 5 and 7 (see Scheme 2) as the common intermediates.^15^

One of the strategies in this work is the thermodynamically controlled, stereoselective formation of 1,3-dioxane (1 in Fig. 1B) by hemiacetal formation followed by oxa-Michael reaction from δ-hydroxyenone derivative that we recently developed (Scheme 1).^16^ The other strategy is the novel stereoselective formation of tetrahydropyranol (2 in Fig. 1B) by the inverse reaction order; oxa-Michael reaction followed by hemiacetal formation (see Scheme 5). Thus, this study succeeded in showing that structural diversity in a compound collection can be acquired by interchanging the order of just two reactions; hemiacetal formation and oxa-Michael reaction. Among the skeletally diverse, heterotricyclic artificial glutamate analogs thus synthesized, a compound named TKM-50 (1ar) was discovered to be neuronally active in the mice *in vivo* assay.

**Scheme 1:**
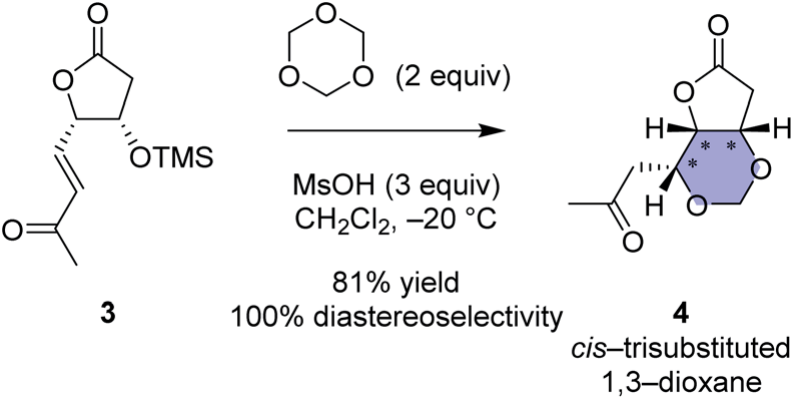
Our recent work regarding stereoselective 1,3-dioxane formation.^16^ For clarity and comparison, enantiomers of the reported compounds are shown in this scheme.

The substrate used for the 1,3-dioxane formation was prepared from the known dimethyl ester 5 (Scheme 2).^17^ Exposure of dimethyl ester 5 to hydrochloric acid (6 M) at 65 °C provided dicarboxylic acid 6.^17^ Without purification, dicarboxylic acid 6 was treated with BnBr and Cs_2_CO_3_ to furnish the common intermediate 7 in 72% yield (2 steps).^18^

**Scheme 2:**
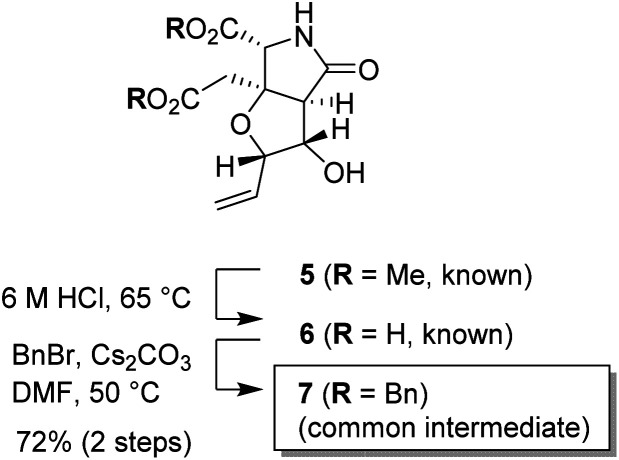
Preparation of the common intermediate 7 (racemate).

The alkene 7 was subjected to cross metathesis with methyl vinyl ketone (8) mediated by Hoveyda-Grubbs second generation catalyst (9)^19^ to provide enone 10 in 82% yield (Scheme 3). Upon exposure to paraformaldehyde as an equivalent of formaldehyde and 1,3,5-trioxane^16^ in the presence of MsOH, 1,3-dioxane ring formed smoothly by oxa-Michael reaction to give rise to desired (7R*)-heterotricycle 11r and the (7S*) epimer 11s (structure not shown) in the ratio of >9 : 1, as well as the *N*-hydroxymethylated product 12r (see Scheme 3) and the (7S*) epimer 12s (structure not shown). Since we had found that alkaline hydrolysis is of use to remove the *N*-hydroxymethyl group, the mixture of hemiaminals (12r/12s) and free amides (11r/11s) was treated with ammonium hydroxide^20^ to obtain free amide 11r in 73% isolated yield (2 steps), and free amide 11s in 10% yield (estimated by NMR, 2 steps). The formation of 1,3-dioxane ring of 11r was determined by the HMBC correlations (Fig. 2A), and the stereochemical configuration was established by a ^3^*J*_H,H_ value and NOESY correlations denoted in Fig. 2B. Both configuration and conformation of 11r are identical to those we observed recently in the simple case (3 → 4, see Scheme 1),^16^ showing that the 1,3-dioxane formation in this study is also thermodynamically controlled (see below for the mechanism).

**Scheme 3:**
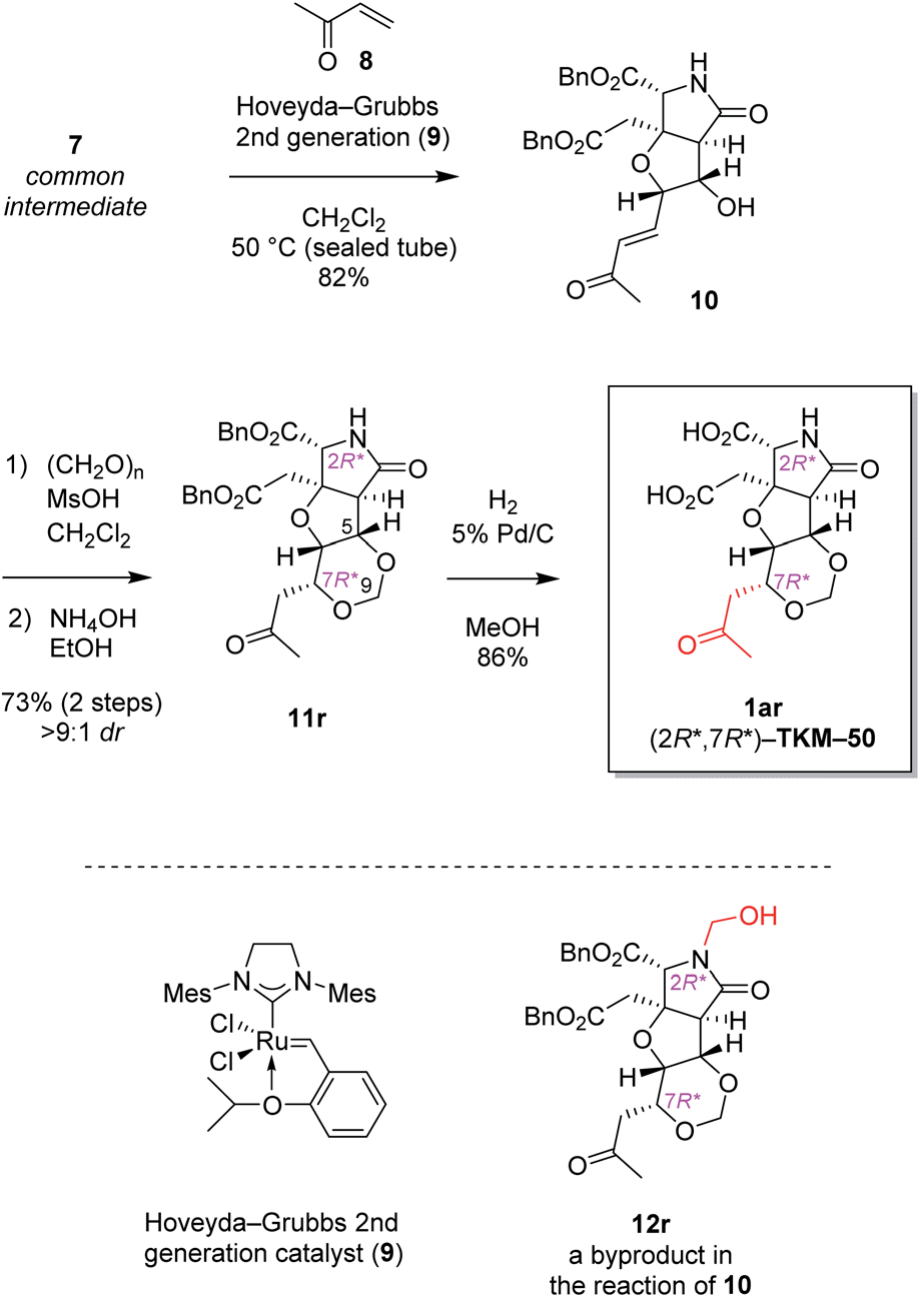
Stereoselective 1,3-dioxane formation leading to heterotricyclic artificial glutamate analog 1ar.^*a a*^dr denotes the diastereoselectivity in the 1,3-dioxane formation.

**Fig. 2:**
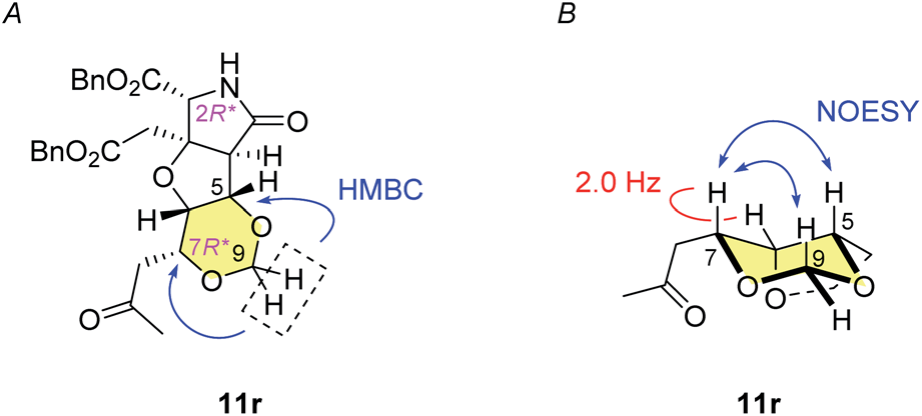
Structure analysis of 1,3-dioxane 11r. (A) HMBC correlations in 11r indicates formation of the 1,3-dioxane ring. (B) Small ^3^*J*_H,H_ value and NOESY correlations show the configuration and the conformation of 11r.

The proposed mechanism for the 1,3-dioxane formation is shown in Scheme 4A. Reaction of alcohol 10 and paraformaldehyde would form hemiacetal intermediate A under acidic conditions, which, then undergoes intramolecular oxa-Michael reaction to give 11r and 11s. Since the second conjugate addition is generally a thermodynamically controlled, reversible process, production of more stable (7R*) isomer 11r predominated over the (7S*) epimer 11s, as discussed also in our preliminary study.^16^ It should be also noted here that, in that preliminary study employing a simple substrate, the (7*S*) epimer had not been obtained.^16^ Generation of the less stable (7S*) epimer 11s in this study would be due to unfavorable steric interactions between the acetyl group and the benzyl ester on the near side in 11r (Scheme 4B), that make the energy difference between the two diastereomers (11r and 11s) smaller.

**Scheme 4:**
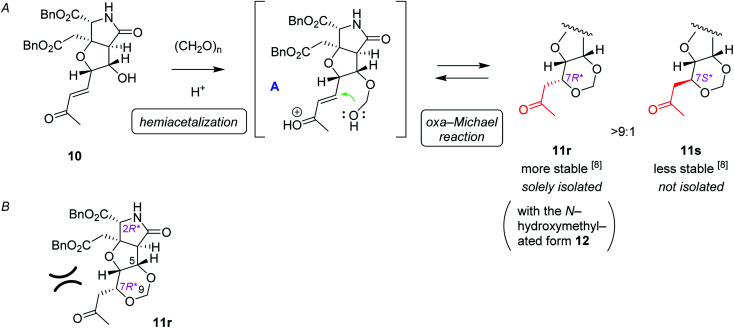
The plausible mechanism of 1,3-dioxane ring formation. (A) Stepwise mechanism that consists of hemiacetal formation followed by intramolecular oxa-Michael reaction. (B) The steric repulsion included in the stable isomer 11r.

Then two benzyl groups of 11r were removed by hydrogenolysis^21^ to cleanly provide glutamate analog 1ar ((2R*,7R*)-TKM-50) in 86% yield (Scheme 3).

With the same reaction sequences for 1ar (Scheme 3), two more analogs 1br and 1cr were furthermore synthesized (Fig. 3). The marked decrease in diastereoselectivity in these oxa-Michael reactions (see Fig. 3) suggests that the steric repulsion between the pentyl/methoxyphenyl group and the benzyl ester on the near side is extremely large. The minor (7S*) diastereomers obtained in these oxa-Michael reactions were also isolated and deprotected to give 1bs and 1cs (see the ESI†), which were subjected to *in vivo* assay (see below).

**Fig. 3:**
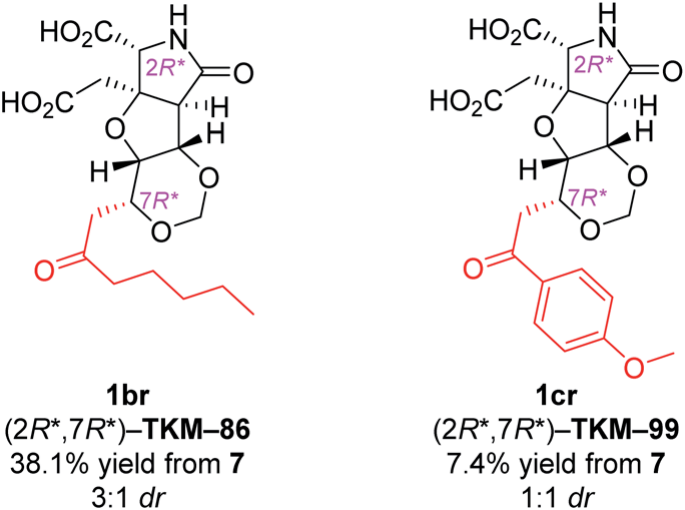
Other 1,3-dioxane analogs synthesized by the intramolecular oxa-Michael reaction.^*a a*^dr denotes the diastereoselectivity in the 1,3-dioxane formation.

We also found that another skeleton can be constructed from δ-hydroxyenone being used for 1,3-dioxane formation, under alkaline hydrolytic conditions. Thus, as shown in Scheme 5, the δ-hydroxyenone 13 derived from homoallylic alcohol 5 by cross metathesis was selectively transformed into cyclic hemiacetal 2 in 53% yield (1 M LiOH in water, MeOH, rt). In this transformation, dimethyl ester and δ-hydroxyenone moiety independently suffer hydrolysis and cyclization, respectively, to generate glutamate analog 2 efficiently. The configuration of 2 was determined by combined analysis of NMR and DFT calculation (see the ESI†).^22^

**Scheme 5:**
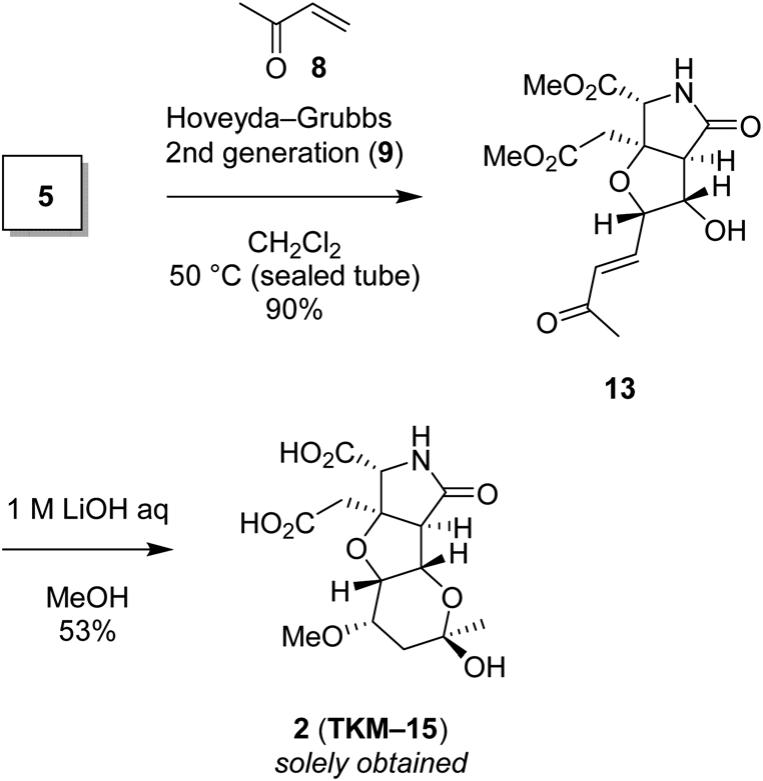
The heterotricyclic artificial glutamate analog 2, constructed by intermolecular oxa-Michael reaction of MeOH followed by acetalization.

The plausible mechanism for the hemiacetal formation is shown in Scheme 6. In view of the fact that the hydroxy and carbonyl groups are located apart in 13, the six-membered-ring formation should take place after saturation of the *trans*-alkene. It is, therefore, supposed that oxa-Michael reaction of MeOH to enone 13 first generates saturated ketone C*via* enolate B.^23^ Under alkaline conditions, the alkoxide C intramolecularly attacks carbonyl group to give rise to hemiacetal 2. Considering the fact that oxa-Michael reaction and the acetalization are thermodynamically controlled, reversible processes, energetically favorable diastereomer 2 would have been obtained predominantly (see the ESI† for discussions on thermodynamic stability of 2). A related example had been reported in 1992 by Shing *et al.*^24^

**Scheme 6:**
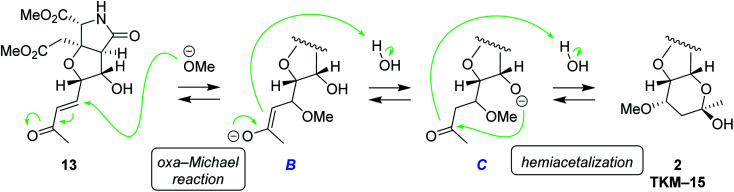
The plausible mechanism for hemiacetal formation under alkaline conditions.

Behavioral activities of all six compounds upon intracerebroventricular (i.c.v.) injection were evaluated in mice (Fig. 4).^25^ Injection of 1ar (TKM-50, 50 μg per mouse) resulted in loss of voluntary motor activity for 10 min after injection and then ataxia-like motions were recorded, thus annotated as hypoactive. The hypoactivity observed for 1ar (TKM-50) is thus somewhat weaker than IKM-159 which causes loss of mice spontaneous activity for up to 4 h.^12^ Other congeners, however, did not cause any noticeable behavioral changes at the same dose tested.

**Fig. 4:**
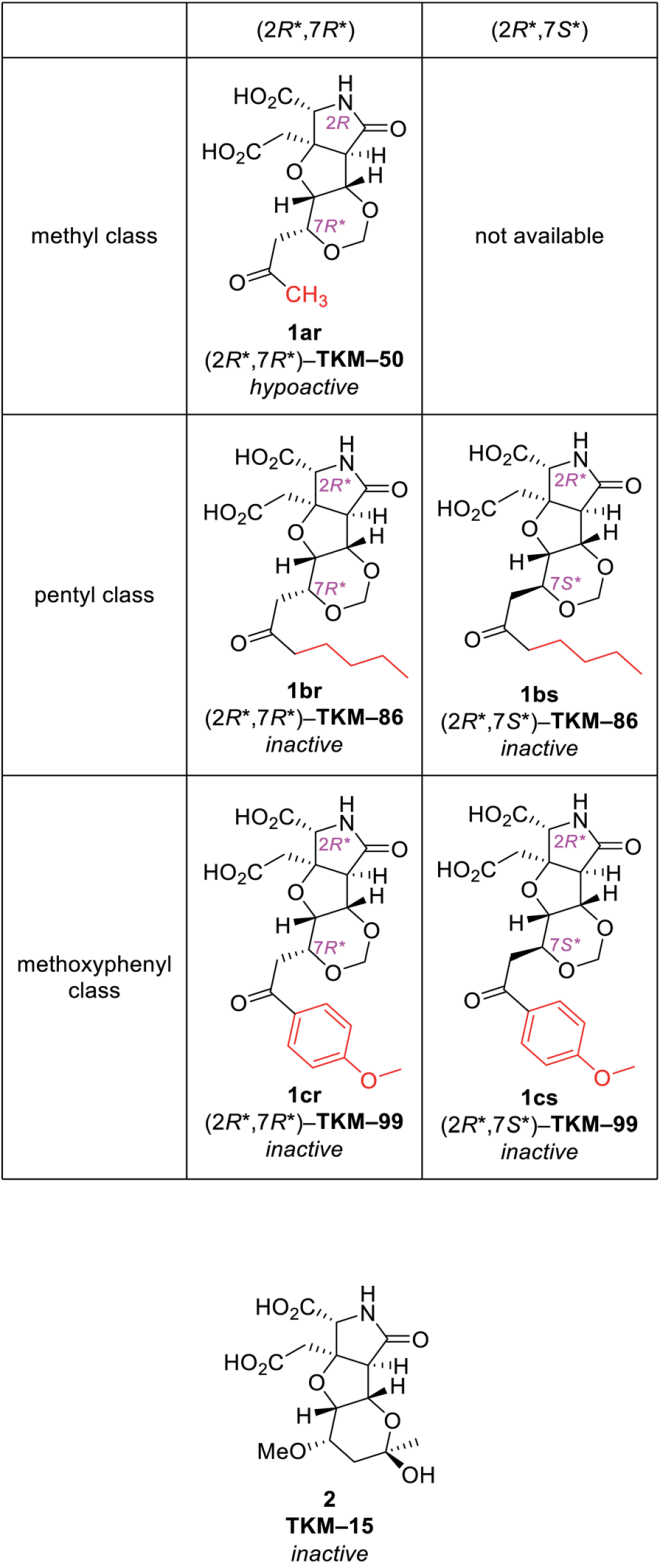
The *in vivo* activities on mice.

## Conclusions

In this paper, we reported synthesis of skeletally diverse artificial glutamate analogs from a common precursor. Since we employed thermodynamically controlled, reversible process for the key cyclizations, most of the reactions proceeded stereoselectively. The cases that were less selective (1br and 1cr in Fig. 3) could even be reasonably explained, supporting the origin of the stereoselectivity we proposed in Scheme 4.^16^

It is of interest to note that the formed skeleton changes significantly, just by interchanging the order of the oxa-Michael reaction and the hemiacetalization (see Schemes 4 and 6). Therefore, it is expected that our methodology is generally of use for discovery of biologically active small molecules.^26^ In fact, we succeeded in identifying neuroactive compound (1ar, TKM-50) in this study.

We are currently working on the construction of a larger compound library using this methodology and the development of alternative methodology for generation of other skeletons. The results will be reported in due course.

## Author contributions

ST: investigation, writing the first draft and editing; OH: investigation and editing; KM: investigation; RI: formal analysis and editing; RS: funding acquisition, investigation and writing the first draft; MO: conceptualization, formal analysis, funding acquisition, project administration, supervision, writing the first draft and final editing.

## Conflicts of interest

There are no conflicts to declare.

## Supplementary Material

RA-012-D2RA03744K-s001

RA-012-D2RA03744K-s002
