# Supplementary figures and images for: Oxa-Michael-based divergent synthesis of artificial glutamate analogs
Source: RSC Adv. 2022 Aug 10;12(34):22175–9. doi: 10.1039/d2ra03744k (PMC9364357; doi:10.1039/d2ra03744k)

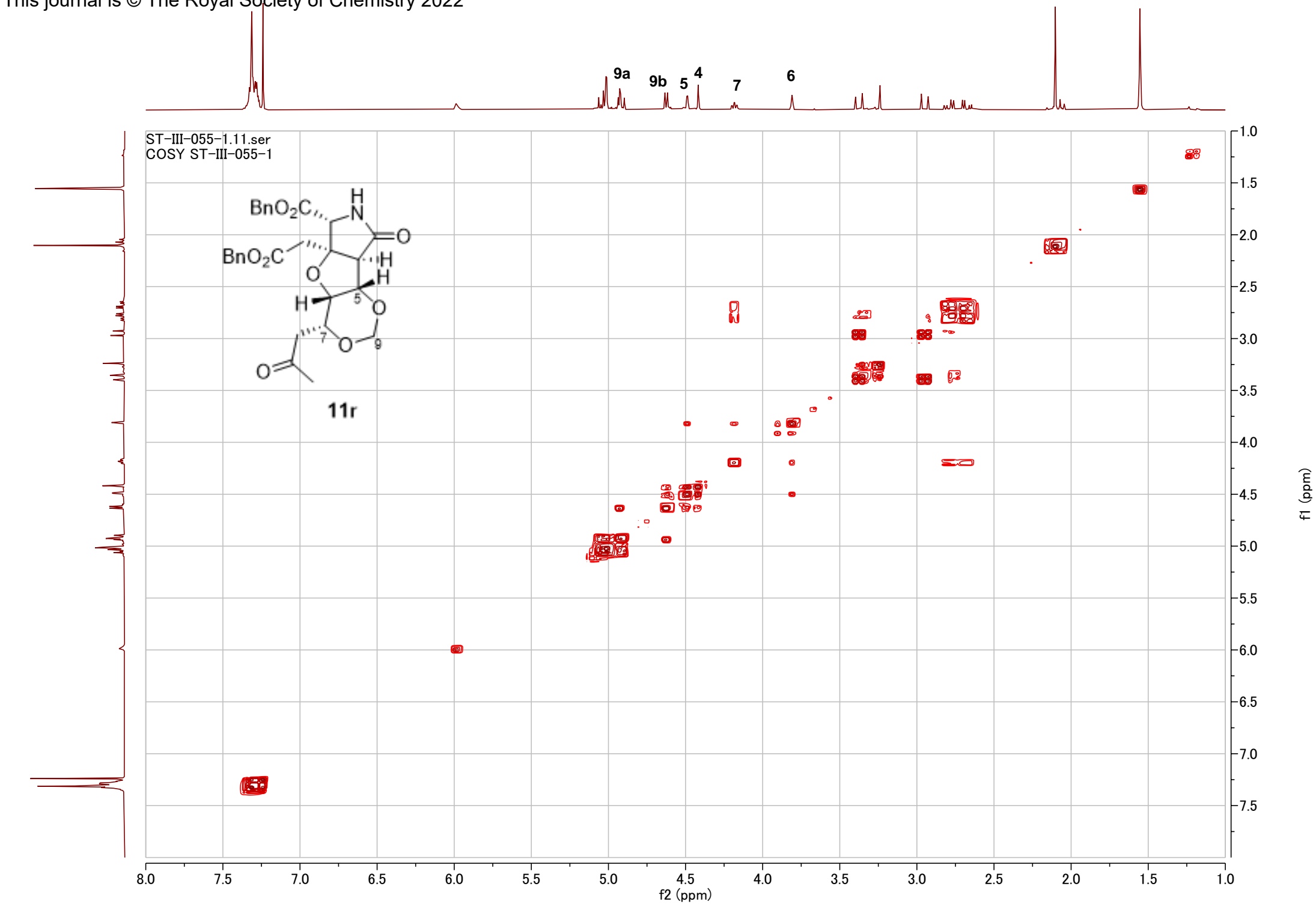

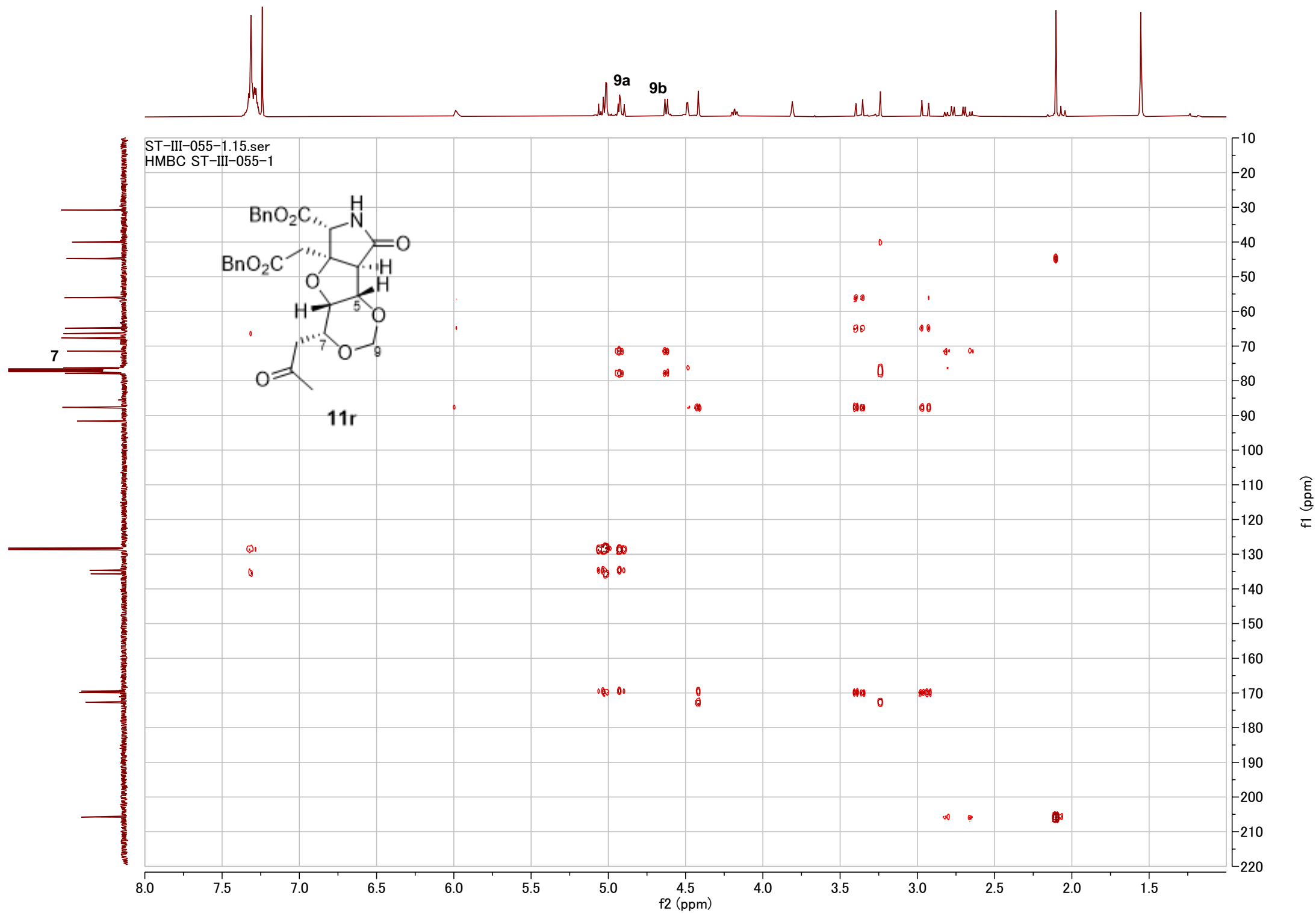

ST-III-054-1.34.ser  
NOESY ST-III-054-1

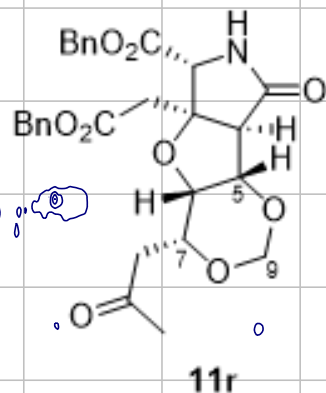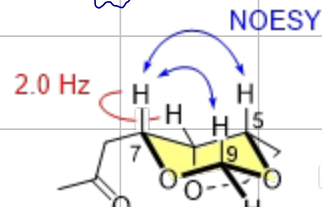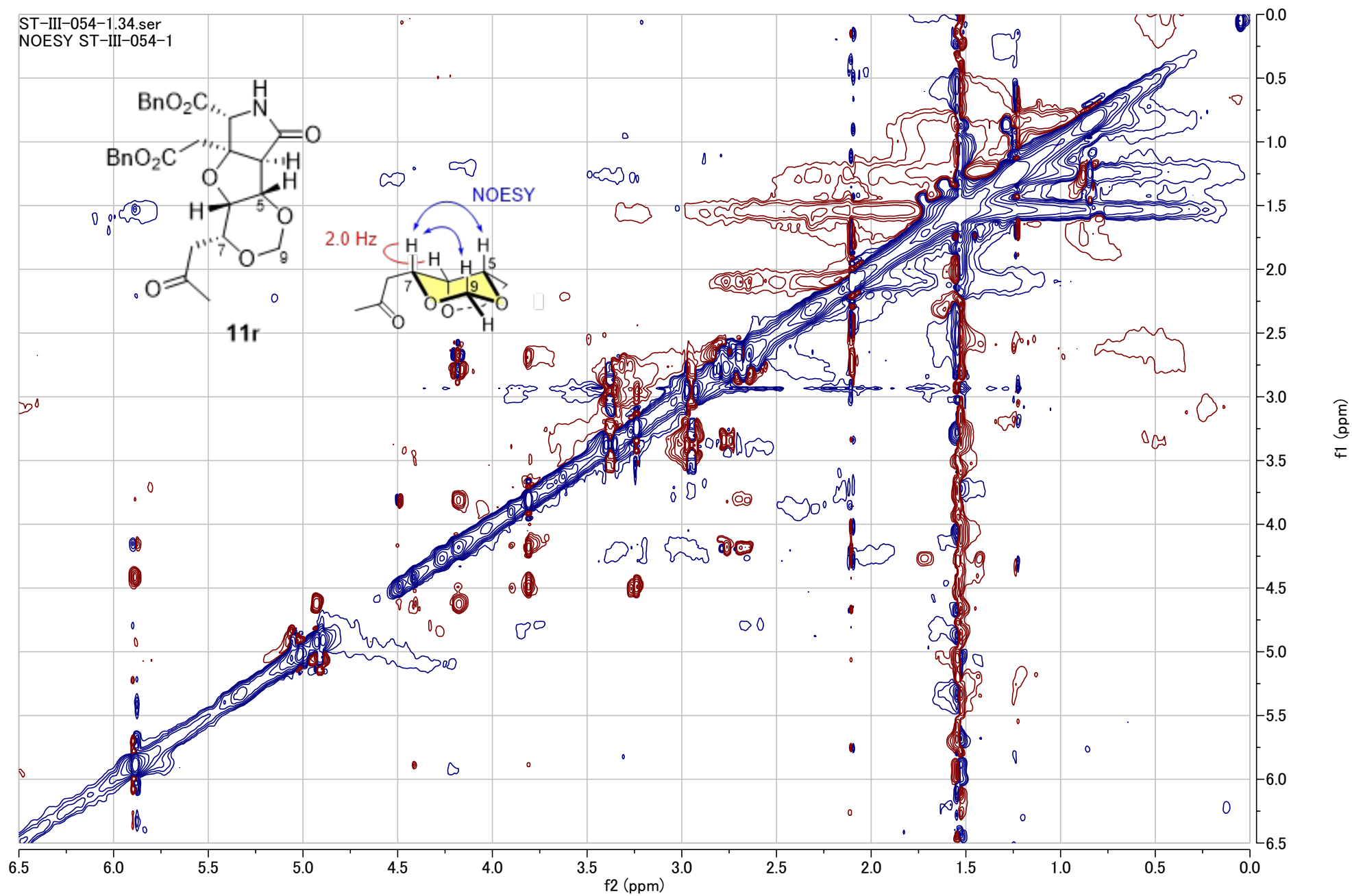

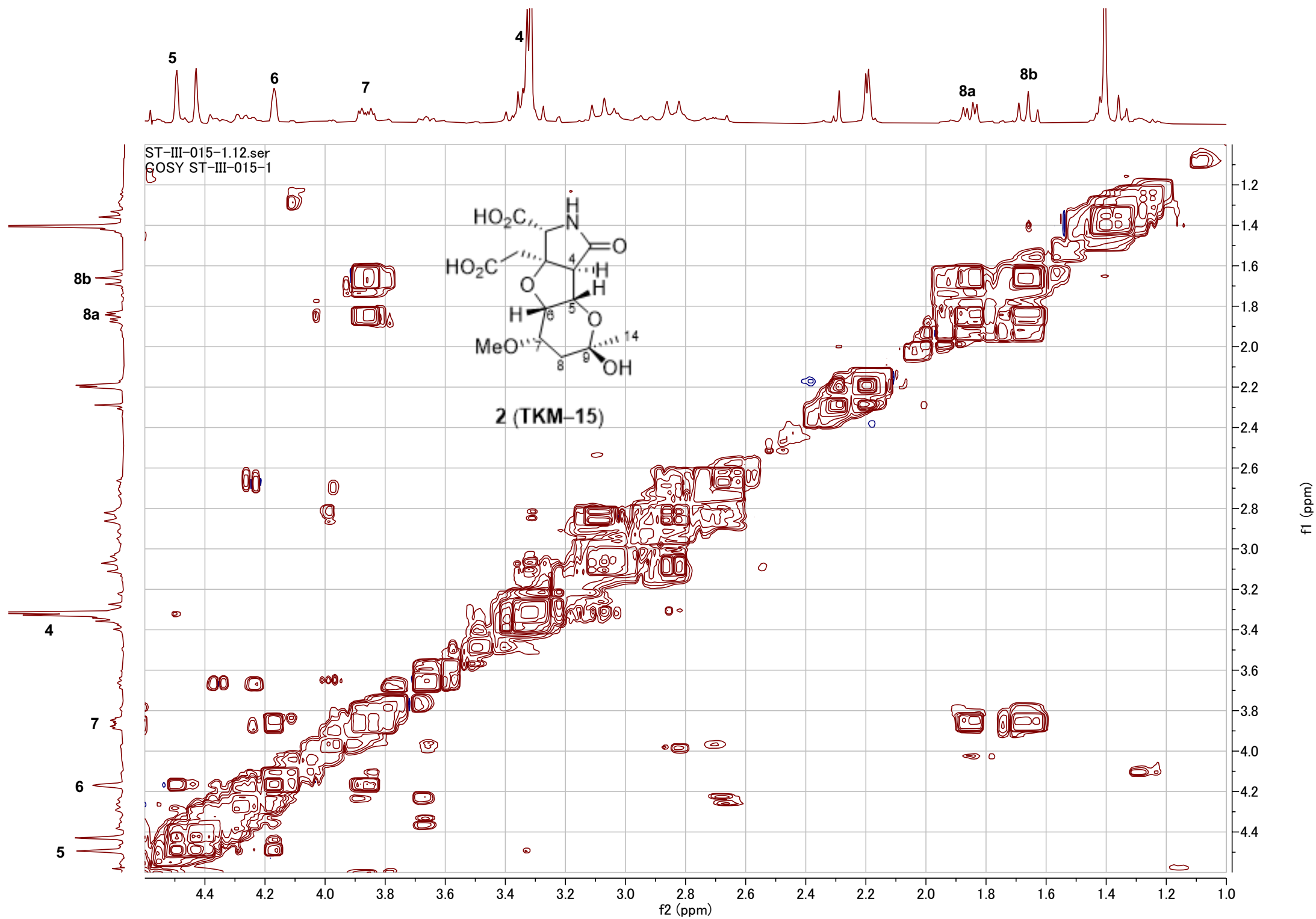

ST-III-015-1.14.ser  
HMBC ST-III-015-1

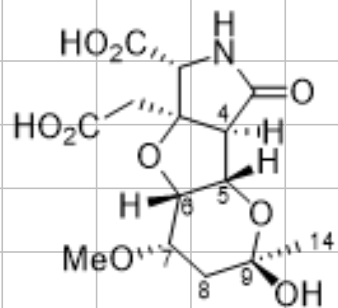

2 (TKM-15)

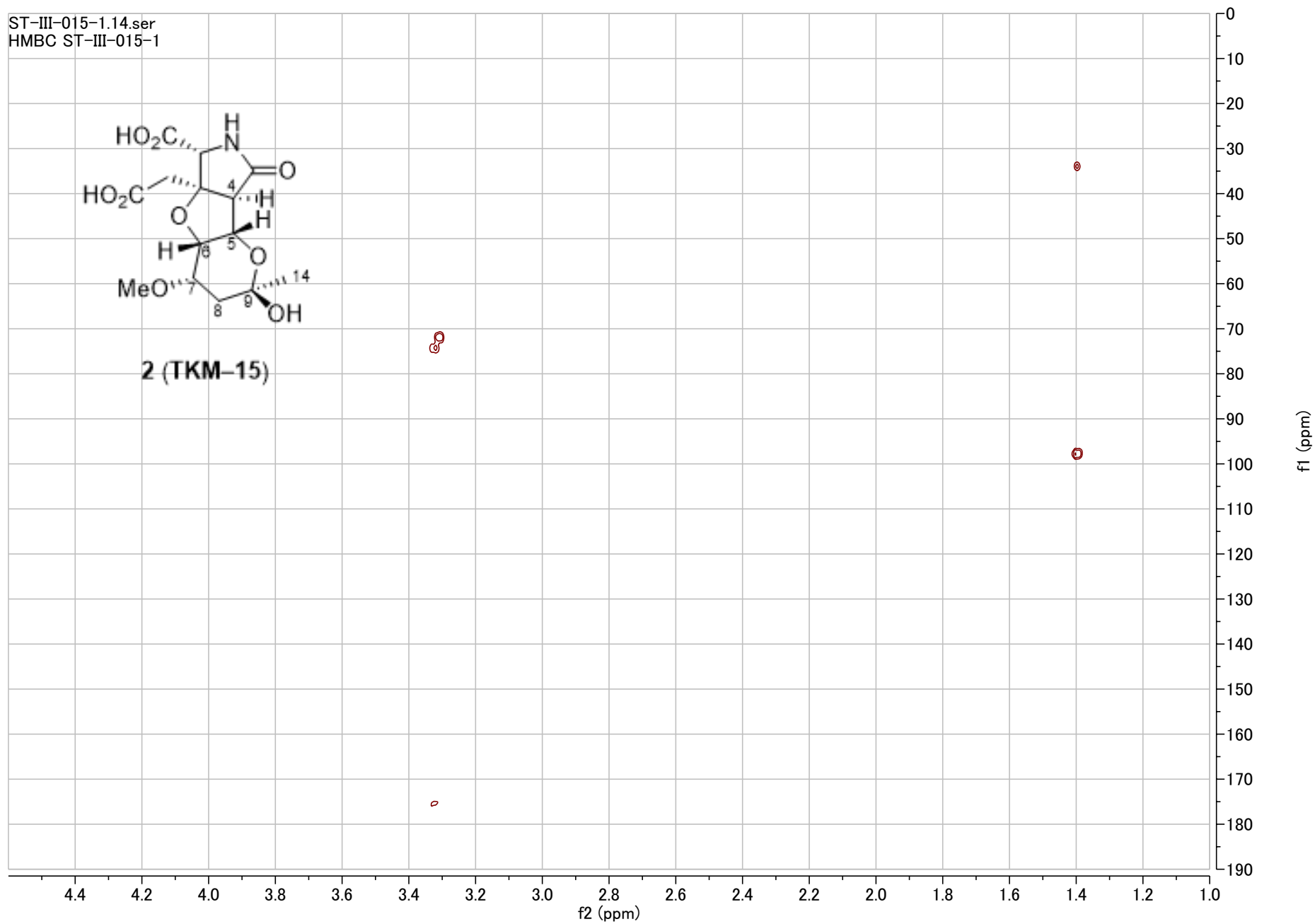

ST-III-015-1.15.ser  
NOESY\_500m ST-III-015-1

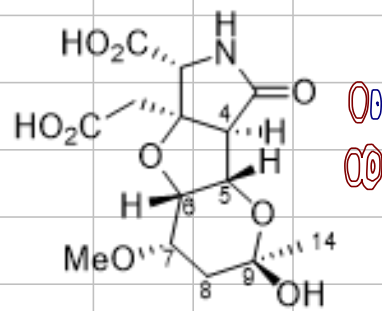

2 (TKM-15)

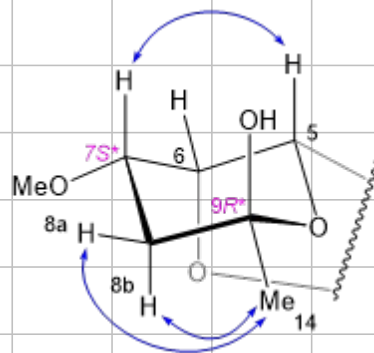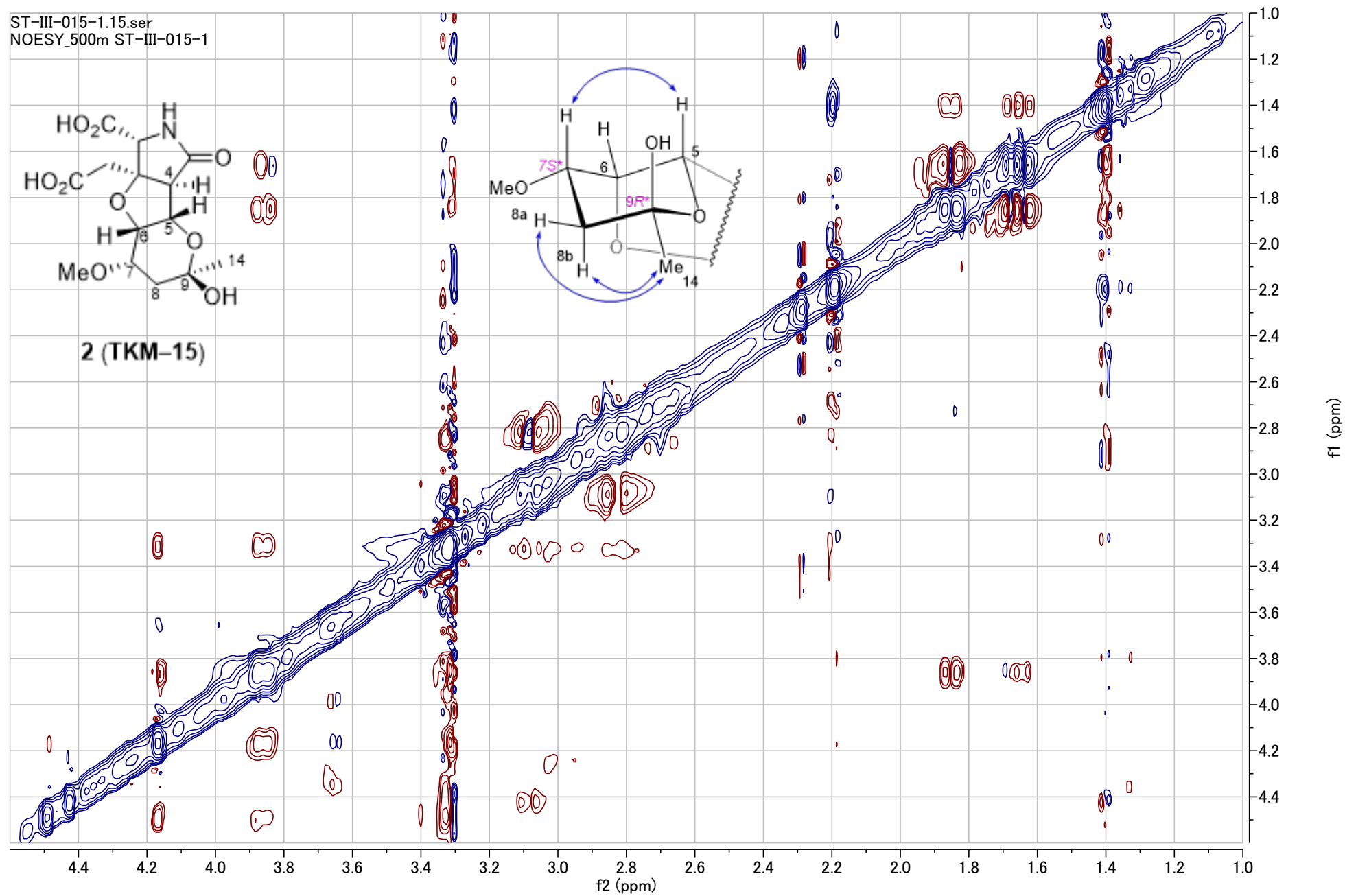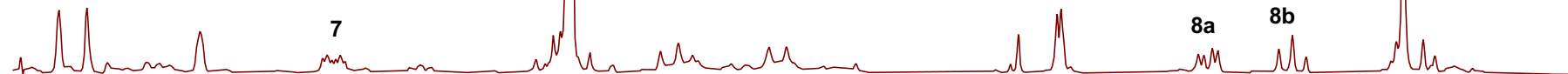

14

5

Supplement: RA-012-D2RA03744K-s001 [file RA-012-D2RA03744K-s001.pdf]
