# Supplementary material for: Oxa-Michael-based divergent synthesis of artificial glutamate analogs
Source: RSC Adv. 2022 Aug 10;12(34):22175–9. doi: 10.1039/d2ra03744k (PMC9364357; doi:10.1039/d2ra03744k)
Supplement: RA-012-D2RA03744K-s002 [file RA-012-D2RA03744K-s002.pdf]

Electronic Supplementary Material (ESI) for RSC Advances.
This journal is © The Royal Society of Chemistry 2022

Supporting Information

Oxa-Michael-based divergent synthesis of artificial glutamate analogs

[AUTHORS]
Shuntaro Tsukamoto,[a] Oriel Hlokoane,†[a] Kei Miyako,[b] Raku Irie,[a] Ryuichi Sakai,[b] Masato Oikawa*[a]

[AFFILIATIONS]
[a] Yokohama City University, Seto 22-2, Kanazawa-ku, Yokohama 236-0027, Japan

[b] Faculty of Fisheries Sciences, Hokkaido University, Hakodate 041-8611, Japan

† Present address: Department of Pharmacy, National University of Lesotho, P.O. Roma 180, Maseru, Lesotho.

[CONTACT INFORMATION]
moikawa@yokohama-cu.ac.jp

[CONTENTS]

Synthetic schemes --- **S3**

Synthetic procedures for all reactions --- **S7**

Configurational and conformational analysis of cyclic hemiacetal **2** (TKM-15) --- **S20**

Mouse behavioral assay --- **S111**

References and notes --- **S111**

NMR spectra of all new compounds --- **S112**

[SYNTHETIC SCHEMES]

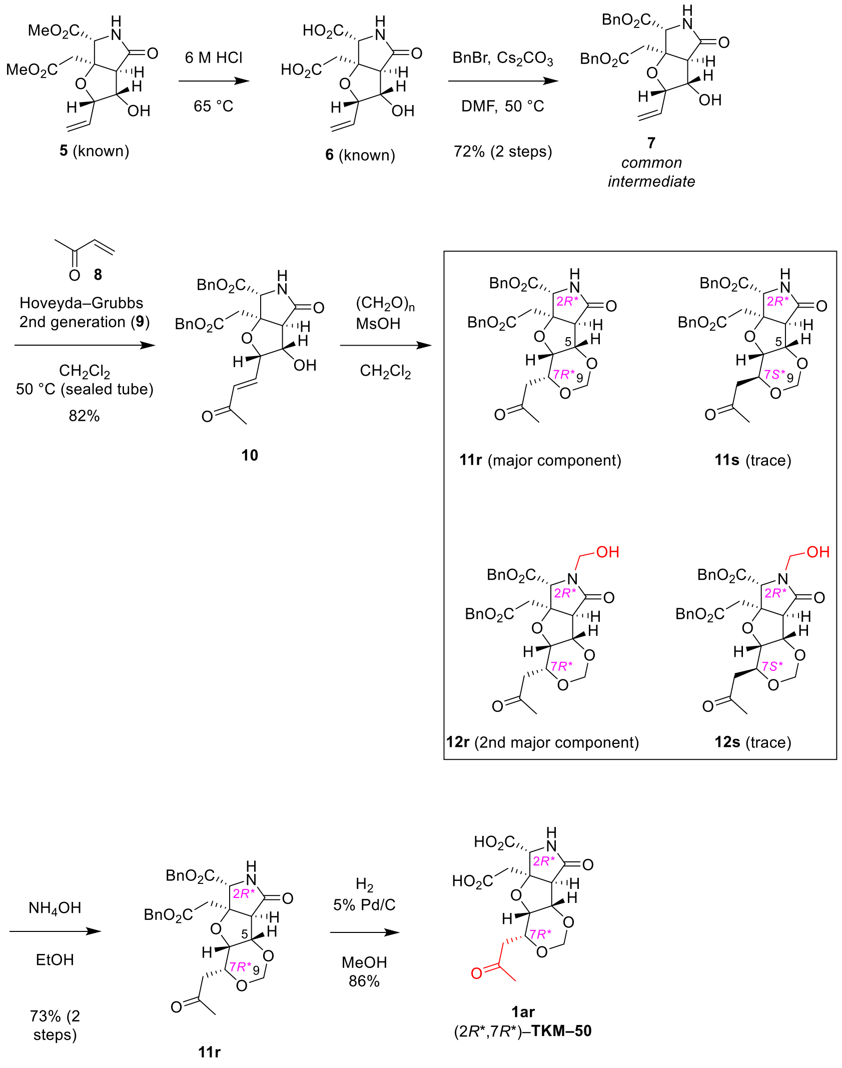

Scheme S1

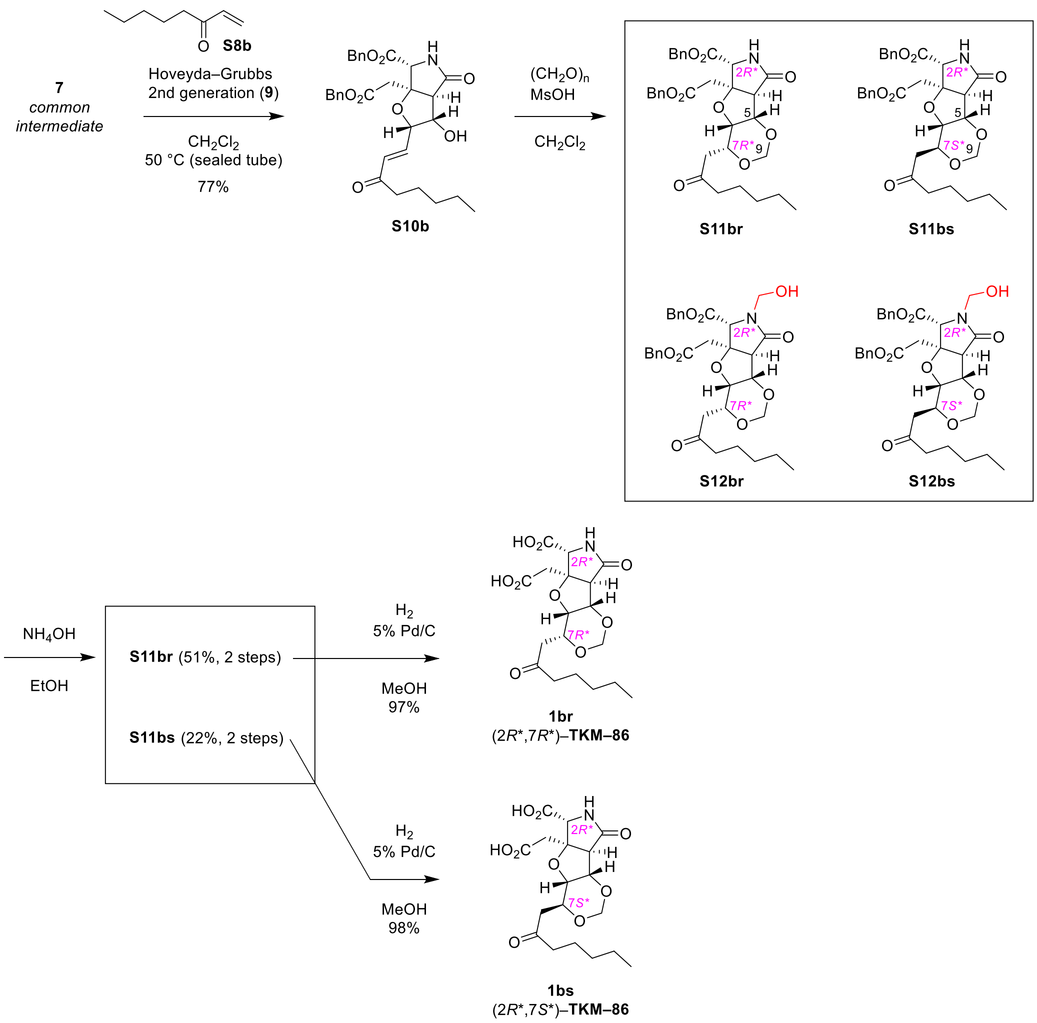

S8b
Hoveyda–Grubbs
2nd generation (9)
CH2Cl2
50 °C (sealed tube)
77%
7
common
intermediate
S10b
(CH2O)n
MsOH
CH2Cl2
S11br
S11bs
S12br
S12bs
NH4OH
EtOH
S11br (51%, 2 steps)
S11bs (22%, 2 steps)
H2
5% Pd/C
MeOH
97%
98%
1br
(2R*,7R*)–TKM–86
1bs
(2R*,7S*)–TKM–86

Scheme S2

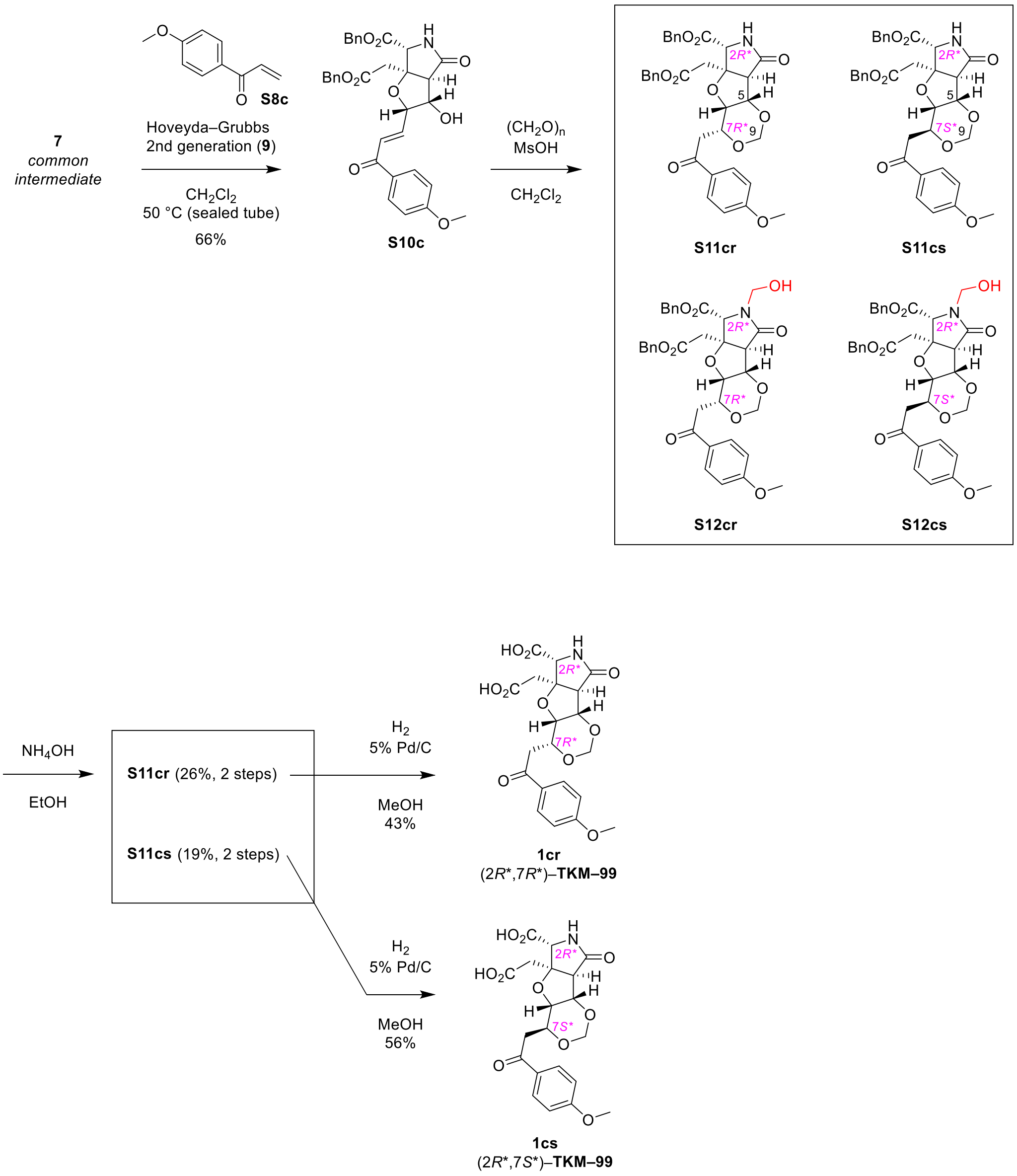
7
common intermediate
S8c
Hoveyda–Grubbs 2nd generation (9)
CH2Cl2
50 °C (sealed tube)
66%
S10c
(CH2O)n
MsOH
CH2Cl2
S11cr
S11cs
S12cr
S12cs
NH4OH
EtOH
S11cr (26%, 2 steps)
S11cs (19%, 2 steps)
H2
5% Pd/C
MeOH
43%
1cr
(2R*,7R*)–TKM–99
H2
5% Pd/C
MeOH
56%
1cs
(2R*,7S*)–TKM–99

Scheme S3

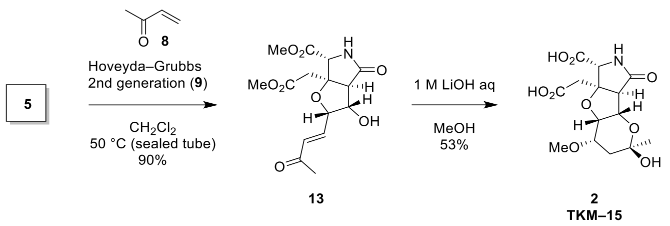
5
O
8
Hoveyda–Grubbs
2nd generation (9)
CH2Cl2
50 °C (sealed tube)
90%
MeO2C
H
N
O
MeO2C
O
H
H
H
OH
O
13
1 M LiOH aq
MeOH
53%
HO2C
H
N
O
HO2C
O
H
H
H
O
MeO
OH
2
TKM–15

Scheme S4

[SYNTHETIC PROCEDURES FOR ALL REACTIONS]

General methods

All reactions susceptible to moisture and air were carried out in an atmosphere of argon gas, using the glassware oven-dried over 3 h. CH_2Cl_2 and THF were purified by Glass Contour Solvent Dispensing System (Nikko Hansen). All other reagents were purchased at the highest commercial grade and used directly. Analytical thin-layer chromatography (TLC) was performed using Merck silica gel 60 F254 plate (0.25-mm thickness). Flash column chromatography was carried out using Kanto Chemical silica gel 60N (40-50 mesh) or Yamazen silica gel HiFlash (SiOH-30µ Premium, 30 µm, 60 Å) with automated flash column systems EPCLC-Wprep2XY-10VW (Yamazen Corporation). Reversed-phase silica gel column chromatography was carried out using Fuji Silysia Chromatorex DM1020T (ODS, 100-200 mesh). For high-performance liquid chromatography (HPLC), a JASCO LC-2000Plus series was used. Optical rotations were recorded on a JASCO P-1030 polarimeter. IR spectra were recorded on a JASCO FT/IR-400 spectrometer. ESI mass spectra were recorded on a Thermo Fisher Scientific Q Exactive Focus mass spectrometer. ^{1}H and ^{13}C NMR spectra were recorded on a BRUKER AVANCE 400 spectrometer or BRUKER AVANCE III HD 400 spectrometer. Chemical shift values are reported in δ (ppm) with reference to internal residual solvent [^{1}H NMR, $CDCl_3$ (7.24), D_2O (4.70), CD_3OD (3.30); ^{13}C NMR, $CDCl_3$ (77.0), D_2O (–), CD_3OD (49.0)]. Coupling constants (*J*) are reported in Hertz (Hz). The following abbreviations were used to designate the multiplicities; s = singlet, d = doublet, dd = double doublet, ddd = double double doublet, dddd = double double double doublet, m = multiplet, br = broad.

Benzyl (2*R,3*R**,3a*R**,6*S**,6a*S**)-6a-(2-(benzyloxy)-2-oxoethyl)-3-hydroxy-4-oxo-2-vinylhexahydro-2*H*-furo[2,3-*c*]pyrrole-6-carboxylate (7)**

A suspension of diester **5** (68.7 mg, 0.230 mmol) in hydrochloric acid (6 M, 15 mL) was stirred at 65 °C for 20 h. The mixture was then

concentrated to dryness by blowing of air to give a residue (70.6 mg), which was mainly composed of dicarboxylic acid **6**.

A portion of the residue thus obtained above (19.5 mg), without purification, was dissolved in DMF (3.5 mL). To the stirred mixture at rt were added Cs_2CO_3 (70.3 mg, 0.216 mmol) and BnBr (0.0427 mL, 0.360 mmol). After stirring at 50 °C for 7 h, the mixture was cooled to rt, poured into saturated aqueous NH_4Cl (4 mL), and extracted with EtOAc (4 × 4 mL). Combined extracts were dried over Na_2SO_4 and concentrated under reduced pressure. The residue was purified by column chromatography on silica gel (60N, 750 mg, EtOAc/hexane = 5:5) to give dibenzyl ester **7** (20.2 mg, 72%) as a yellow foam.

Data for dibenzyl ester 7: IR (ATR) 3526, 3185, 2889, 1739, 1715, 1703, 1457, 1362, 1207, 1076, 1038 cm^{-1}; ^1H NMR (400 MHz, $CDCl_3$) δ 7.37–7.27 (m, 10H), 6.11 (brs, 1H), 5.85 (ddd, *J* = 16.9, 10.6, 6.0 Hz, 1H), 5.39 (d, *J* = 16.9 Hz, 1H), 5.34 (d, *J* = 10.6 Hz, 1H), 5.11 (d, *J* = 12.0 Hz, 1H), 5.08 (d, *J* = 12.0 Hz, 1H), 5.05 (d, *J* = 12.3 Hz, 1H), 5.02 (d, *J* = 12.3 Hz, 1H), 4.41 (d, *J* = 3.3 Hz, 1H), 4.39–4.34 (m, 2H), 3.25 (s, 1H), 2.97 (d, *J* = 17.0 Hz, 1H), 2.90 (d, *J* = 17.0 Hz, 1H); ^{13}C NMR (100 MHz, $CDCl_3$) δ 173.9, 170.4, 169.6, 135.2, 134.5, 131.6, 128.8 (×3), 128.7 (×2), 128.6 (×2), 128.5 (×3), 119.7, 86.7, 84.3, 76.7, 67.8, 66.9, 65.6, 58.4, 39.3; HRMS (ESI, positive) calcd for $C_{25}H_{25}NO_7Na^+$ [$(M+Na)^+$] 474.1523, found 474.1523.

Benzyl (2*R,3*R**,3a*R**,6*S**,6a*S**)-6a-(2-(benzyloxy)-2-oxoethyl)-3-hydroxy-4-oxo-2-((*E*)-3-oxobut-1-en-1-yl)hexahydro-2*H*-furo[2,3-*c*]pyrrole-6-carboxylate (10)**

To a stirred solution of alkene **7** (5.97 mg, 0.0132 mmol) and 3-buten-2-one (0.0054 mL, 0.066 mmol) in CH_2Cl_2 (0.300 mL) at rt was added Hoveyda-Grubbs catalyst second generation (0.2 mg, 0.0003 mmol). After stirring at 50 °C in a sealed tube for 70 h, the mixture was concentrated under reduced pressure. The residue was purified by column chromatography on silica gel (60N, 600 mg, EtOAc/hexane = 4:6) to give enone **10** (5.37 mg, 82%) as a colorless oil: IR (ATR) 3587, 3299, 3180, 3030, 1747, 1734, 1716, 1679, 1557, 1456, 1364 cm^{-1}; ^1H

NMR (400 MHz, $CDCl_3$) δ 7.40–7.26 (m, 10H), 6.69 (dd, *J* = 16.1, 4.9 Hz, 1H), 6.28 (d, *J* = 16.1 Hz, 1H), 6.07 (brs, 1H), 5.12 (s, 2H), 5.09 (d, *J* = 12.2 Hz, 1H), 5.03 (d, *J* = 12.2 Hz, 1H), 4.55 (d, *J* = 3.5 Hz, 1H), 4.52 (m, 1H), 4.34 (s, 1H), 3.26 (s, 1H), 2.99 (d, *J* = 17.2 Hz, 1H), 2.84 (d, *J* = 17.2 Hz, 1H), 2.24 (s, 3H); ^{13}C NMR (100 MHz, $CDCl_3$) δ 197.9, 173.7, 170.4, 169.5, 139.6, 135.0, 134.4, 132.5, 128.9, 128.9 (×2), 128.8 (×2), 128.6 (×2), 128.6, 128.5 (×2), 87.1, 82.7, 77.0, 67.9, 67.1, 65.6, 58.7, 39.0, 27.3; HRMS (ESI, positive) calcd for $C_{27}H_{27}NO_8Na^+$ [$(M+Na)^+$] 516.1629, found 516.1625.

Benzyl (4*S,4a*R**,5a*S**,6*S**,8a*R**,8b*R**)-5a-(2-(benzyloxy)-2-oxoethyl)-8-oxo-4-(2-oxopropyl)octahydro-[1,3]dioxino[4',5':4,5]furo[2,3-*c*]pyrrole-6-carboxylate (11r)**

To a stirred solution of enone **10** (8.46 mg, 0.0171 mmol) in CH_2Cl_2 (0.300 mL) at rt were added paraformaldehyde (0.6 mg, 0.02 mmol) and MsOH (0.0022 mL, 0.034 mmol). After 16 h, the mixture was poured into water (0.5 mL) and extracted with EtOAc (6 × 0.5 mL). Combined extracts were dried over Na_2SO_4 and concentrated under reduced pressure to give a residue (8.82 mg), which is mainly a mixture composed of 1,3-dioxane **11r** and *N*-hydroxymethylated 1,3-dioxane **12r** (**11r**/**12r** = 2.2:1).

A portion of the residue thus obtained above (1.01 mg), without purification, was dissolved in EtOH (0.500 mL). To the stirred mixture at rt was added ammonium hydroxide (28%, 0.0006 mL, 0.009 mmol). After 2 h, the mixture was concentrated under reduced pressure to give a residue (0.92 mg), which is a mixture composed of 1,3-dioxane **11r** and *N*-hydroxymethylated 1,3-dioxane **12r** (**11r**/**12r** = 2.9:1).

The residue thus obtained, without purification, was dissolved in EtOH (0.250 mL). To the stirred mixture at rt was added ammonium hydroxide (28%, 0.0006 mL, 0.009 mmol). After 29 h, the mixture was concentrated under reduced pressure. The residue was purified by column chromatography on silica gel (60N, 500 mg, EtOAc/hexane = 7:3) to give 1,3-dioxane **11r** (0.75 mg, 73%) as a colorless oil: IR (ATR) 3566, 2935, 1742, 1732, 1718, 1696, 1520, 1363, 1208, 1077, 883 cm^{-}

[1]; ^{1}H NMR (400 MHz, $CDCl_3$) δ 7.37–7.25 (m, 10H), 5.99 (brs, 1H), 5.05 (d, *J* = 12.1 Hz, 1H), 5.02 (d, *J* = 12.3 Hz, 1H), 5.00 (d, *J* = 12.3 Hz, 1H), 4.93 (d, *J* = 6.7 Hz, 1H), 4.91 (d, *J* = 12.1 Hz, 1H), 4.63 (d, *J* = 6.7 Hz, 1H), 4.49 (d, *J* = 2.3 Hz, 1H), 4.42 (s, 1H), 4.18 (ddd, *J* = 7.0, 6.0, 2.3 Hz, 1H), 3.81 (dd, *J* = 2.3, 2.3 Hz, 1H), 3.38 (d, *J* = 17.4 Hz, 1H), 3.24 (s, 1H), 2.95 (d, *J* = 17.4 Hz, 1H), 2.79 (dd, *J* = 17.5, 7.0 Hz, 1H), 2.68 (dd, *J* = 17.5, 6.0 Hz, 1H), 2.10 (s, 3H); ^{13}C NMR (100 MHz, $CDCl_3$) δ 205.8, 172.7, 169.9, 169.5, 135.6, 134.6, 128.7, 128.7 (×2), 128.6 (×2), 128.5 (×2), 128.3, 128.2 (×2), 91.7, 87.7, 77.8, 76.3, 71.4, 67.7, 66.4, 64.8, 56.1, 44.7, 40.0, 30.7; HRMS (ESI, positive) calcd for $C_{28}H_{29}NO_9Na^+$ [$(M+Na)^+$] 546.1735, found 546.1733.

(4*S,4a*R**,5a*S**,6*S**,8a*R**,8b*R**)-5a-(Carboxymethyl)-8-oxo-4-(2-oxopropyl)octahydro-[1,3]dioxino[4',5':4,5]furo[2,3-*c*]pyrrole-6-carboxylic acid (1ar, (*2R**,*7R**)-TKM-50)**

A mixture of dibenzyl ester **11r** (4.55 mg, 0.0087 mmol) and Pd/C (5%, 0.9 mg) in MeOH (1 mL) was stirred under hydrogen atmosphere (balloon) at rt. After 43 h, the mixture was filtered through a pad of Celite and concentrated under reduced pressure to give glutamate analog **1ar** (**(*2R**,*7R**)-TKM-50**, 2.55 mg, 86%) as a colorless oil which was sufficiently pure for characterization.

Data for **(*2R**,*7R**)-TKM-50** (**1ar**): IR (ATR) 3360, 2929, 2843, 1739, 1722, 1709, 1692, 1359, 1175, 1078, 1020 cm^{-1}; ^{1}H NMR (400 MHz, D_2O) δ 4.94 (d, *J* = 6.9 Hz, 1H), 4.77 (d, *J* = 6.9 Hz, 1H), 4.57 (d, *J* = 2.2 Hz, 1H), 4.43 (s, 1H), 4.38 (ddd, *J* = 8.8, 4.1, 2.2 Hz, 1H), 3.87 (dd, *J* = 2.2, 2.2 Hz, 1H), 3.32 (s, 1H), 3.25 (d, *J* = 17.1 Hz, 1H), 2.95 (dd, *J* = 17.7, 8.8 Hz, 1H), 2.93 (d, *J* = 17.1 Hz, 1H), 2.82 (dd, *J* = 17.7, 4.1 Hz, 1H), 2.19 (s, 3H); ^{13}C NMR (100 MHz, D_2O) δ 211.8, 175.2, 173.9, 173.6, 91.3, 87.4, 77.6, 76.4, 71.2, 66.4, 56.6, 44.6, 40.0, 29.8; HRMS (ESI, negative) calcd for $C_{14}H_{16}NO_9^-$ [$(M-H)^-$] 342.0831, found 342.0844.

Benzyl (2*S,3*S**,3a*S**,6*R**,6a*R**)-6a-(2-(benzyloxy)-2-oxoethyl)-3-hydroxy-4-oxo-2-((*E*)-3-oxooct-1-en-1-yl)hexahydro-2*H*-furo[2,3-**

c]pyrrole-6-carboxylate (S10b)

To a stirred solution of alkene **7** (14.8 mg, 0.0328 mmol) and 1-octen-3-one (0.0249 mL, 0.164 mmol) in CH_2Cl_2 (0.400 mL) at rt was added Hoveyda-Grubbs catalyst second generation (1.0 mg, 0.0016 mmol). After stirring at 50 °C in a sealed tube for 18 h, the mixture was concentrated under reduced pressure. The residue was purified by column chromatography on silica gel (60N, 600 mg, EtOAc/hexane = 5:5) to give enone **S10b** (13.9 mg, 77%) as a brown oil: IR (ATR) 3545, 3382, 3223, 2951, 1742, 1716, 1704, 1682, 1387, 1191, 1038 cm^{-1}; ^{1}H NMR (400 MHz, $CDCl_3$) δ 7.39–7.25 (m, 10H), 6.71 (dd, *J* = 16.0, 4.8 Hz, 1H), 6.50 (brs, 1H), 6.30 (dd, *J* = 16.0, 1.4 Hz, 1H), 5.11 (d, *J* = 12.0 Hz, 1H), 5.09 (d, *J* = 12.0 Hz, 1H), 5.06 (d, *J* = 12.3 Hz, 1H), 5.03 (d, *J* = 12.3 Hz, 1H), 4.57–4.47 (m, 2H), 4.36 (s, 1H), 3.78 (d, *J* = 7.1 Hz, 1H), 3.25 (s, 1H), 2.98 (d, *J* = 17.2 Hz, 1H), 2.89 (d, *J* = 17.2 Hz, 1H), 2.50 (t, *J* = 7.3 Hz, 2H), 1.57 (tt, *J* = 7.3, 7.3 Hz, 2H), 1.36–1.19 (m, 4H), 0.86 (t, *J* = 6.9 Hz, 3H); ^{13}C NMR (100 MHz, $CDCl_3$) δ 200.2, 174.0, 170.3, 169.6, 138.6, 135.1, 134.5, 131.6, 128.8, 128.8 (×2), 128.7 (×2), 128.6 (×2), 128.5, 128.4 (×2), 87.1, 82.9, 76.8, 67.8, 67.0, 65.7, 58.7, 40.6, 39.1, 31.4, 23.6, 22.4, 13.9; HRMS (ESI, positive) calcd for $C_{31}H_{35}NO_8Na^+$ [$(M+Na)^+$] 572.2255, found 572.2247.

Benzyl (4*R,4a*S**,5a*R**,6*R**,8a*S**,8b*S**)-5a-(2-(benzyloxy)-2-oxoethyl)-8-oxo-4-(2-oxoheptyl)octahydro-[1,3]dioxino[4',5':4,5]furo[2,3-*c*]pyrrole-6-carboxylate (S11br), and benzyl (4*S**,4a*S**,5a*R**,6*R**,8a*S**,8b*S**)-5a-(2-(benzyloxy)-2-oxoethyl)-8-oxo-4-(2-oxoheptyl)octahydro-[1,3]dioxino[4',5':4,5]furo[2,3-*c*]pyrrole-6-carboxylate (S11bs)**

To a stirred solution of enone **S10b** (2.98 mg, 0.0054 mmol) in CH_2Cl_2 (0.500 mL) at -20 °C were added paraformaldehyde (0.2 mg, 0.007 mmol) and MsOH (0.0007 mL, 0.01 mmol). After stirring for 22 h, the mixture was allowed to warm to 0 °C, and stirring was continued for 30 h. The mixture was then poured into water (0.5 mL) and extracted with EtOAc (6 × 0.5 mL). Combined extracts were dried over Na_2SO_4 and concentrated under reduced pressure to give a residue (2.82 mg), which is a mixture composed of *N*-hydroxymethylated 1,3-dioxanes **S12br**,

S12bs and 1,3-dioxanes **S11br**, **S11bs** ((**S12br**, **S12bs**)/(**S11br**, **S11bs**) = 1:9.7).

The residue thus obtained above, without purification, was dissolved in EtOH (0.500 mL). To the stirred mixture at rt was added ammonium hydroxide (28%, 0.0016 mL, 0.023 mmol). After 13 h, the mixture was concentrated under reduced pressure. The residue was purified by column chromatography on silica gel (60N, 500 mg, EtOAc/hexane = 3:7) to give a mixture of 1,3-dioxane **S11br** and **S11bs** (2.37 mg, **S11br**/**S11bs** = 2.8:1) as a colorless oil.

Purification of a portion of the mixture (2.05 mg) by HPLC (XTerra™ MS, 4.6 × 150 mm, MeOH/H_2O = 8:2, 1.0 mL/min, 40 °C, detected at 254 nm) gave diastereomerically pure 1,3-dioxanes **S11br** (7*R**, 1.40 mg, 51%, t_R 4.9 min) and **S11bs** (7*S**, 0.61 mg, 22%, t_R 4.2 min).

Data for 1,3-dioxane S11br (7*R)**: retention time 4.9 min; IR (ATR) 3297, 2926, 2870, 1740, 1730, 1714, 1679, 1456, 1386, 1190, 1093 cm^{-1}; ^1H NMR (400 MHz, $CDCl_3$) δ 7.36–7.26 (m, 10H), 5.89 (brs, 1H), 5.04 (d, *J* = 12.1 Hz, 1H), 5.02 (d, *J* = 12.3 Hz, 2H), 5.00 (d, *J* = 12.3 Hz, 1H), 4.93 (d, *J* = 6.7 Hz, 1H), 4.90 (d, *J* = 12.1 Hz, 1H), 4.62 (d, *J* = 6.7 Hz, 1H), 4.49 (d, *J* = 2.3 Hz, 1H), 4.40 (s, 1H), 4.20 (ddd, *J* = 7.1, 6.0, 2.3 Hz, 1H), 3.81 (dd, *J* = 2.3, 2.3 Hz, 1H), 3.38 (d, *J* = 17.4 Hz, 1H), 3.24 (s, 1H), 2.96 (d, *J* = 17.4 Hz, 1H), 2.77 (dd, *J* = 17.3, 7.1 Hz, 1H), 2.64 (dd, *J* = 17.3, 6.0 Hz, 1H), 2.34 (t, *J* = 7.4 Hz, 2H), 1.57–1.46 (m, 1H), 1.34–1.15 (m, 4H), 0.86 (t, *J* = 7.0 Hz, 3H); ^{13}C NMR (100 MHz, $CDCl_3$) δ 208.2, 172.6, 169.9, 169.4, 135.6, 134.6, 128.7, 128.7 (×2), 128.6 (×2), 128.5 (×2), 128.3, 128.2 (×2), 91.7, 87.7, 77.8, 76.4, 71.6, 67.7, 66.4, 64.8, 56.1, 43.9, 43.6, 40.0, 31.3, 23.2, 22.4, 13.9; HRMS (ESI, positive) calcd for $C_{32}H_{37}NO_9Na^+$ [$(M+Na)^+$] 602.2361, found 602.2341.

Data for 1,3-dioxane S11bs (7*S)**: retention time 4.2 min; IR (ATR) 3222, 2921, 2898, 2850, 1738, 1724, 1712, 1696, 1532, 1379, 1217 cm^{-1}; ^1H NMR (400 MHz, $CDCl_3$) δ 7.35–7.25 (m, 10H), 5.88 (s, 1H), 5.04 (d, *J* = 12.1 Hz, 2H), 5.01 (d, *J* = 12.1 Hz, 1H), 4.90 (d, *J* = 12.1

Hz, 1H), 4.79 (d, *J* = 6.2 Hz, 1H), 4.75 (d, *J* = 6.2 Hz, 1H), 4.52–4.45 (m, 3H), 3.69 (dd, *J* = 2.4, 2.4 Hz, 1H), 3.38 (d, *J* = 17.4 Hz, 1H), 3.28 (s, 1H), 2.98 (d, *J* = 17.4 Hz, 1H), 2.82 (dd, *J* = 16.3, 7.9 Hz, 1H), 2.62 (dd, *J* = 16.3, 6.7 Hz, 1H), 2.40 (t, *J* = 7.5 Hz, 2H), 1.60–1.47 (m, 2H), 1.33–1.18 (m, 4H), 0.86 (t, *J* = 7.0 Hz, 3H); ^{13}C NMR (100 MHz, $CDCl_3$) δ 206.9, 172.7, 169.9, 169.4, 135.5, 134.6, 128.7, 128.7 (×2), 128.6 (×2), 128.5 (×2), 128.3 (×3), 87.9, 86.5, 78.5, 75.6, 69.0, 67.7, 66.5, 64.7, 56.0, 43.1, 43.1, 40.0, 31.3, 23.2, 22.4, 13.9; HRMS (ESI, positive) calcd for $C_{32}H_{37}NO_9Na^+$ [(M+Na)$^+$] 602.2361, found 602.2340.

(4*S,4a*R**,5a*S**,6*S**,8a*R**,8b*R**)-5a-(Carboxymethyl)-8-oxo-4-(2-oxoheptyl)octahydro-[1,3]dioxino[4',5':4,5]furo[2,3-*c*]pyrrole-6-carboxylic acid (1br, (*2R**,*7R**)-TKM-86)**

A mixture of dibenzyl ester **S11br** (2.53 mg, 0.0044 mmol) and Pd/C (5%, 0.5 mg) in MeOH (0.250 mL) and THF (0.250 mL) was stirred under hydrogen atmosphere (balloon) at rt. After 20 h, the mixture was filtered through a pad of Celite and concentrated under reduced pressure. The residue was purified by trituration with water three times to give glutamate analog **1br** (**(*2R**,*7R**)-TKM-86**, 1.69 mg, 97%) as a colorless oil: IR (ATR) 3543, 3273, 3192, 1737, 1722, 1708, 1687, 1412, 1368, 1190, 1033 cm^{-1}; ^{1}H NMR (400 MHz, D_2O) δ 4.92 (d, *J* = 6.9 Hz, 1H), 4.74 (d, *J* = 6.9 Hz, 1H), 4.55 (d, *J* = 2.3 Hz, 1H), 4.36 (ddd, *J* = 8.7, 4.4, 2.3 Hz, 1H), 4.30 (s, 1H), 3.83 (dd, *J* = 2.3, 2.3 Hz, 1H), 3.24 (s, 1H), 3.15 (d, *J* = 16.0 Hz, 1H), 2.93 (dd, *J* = 17.6, 8.7 Hz, 1H), 2.85 (d, *J* = 16.0 Hz, 1H), 2.78 (dd, *J* = 17.6, 4.4 Hz, 1H), 2.57–2.40 (m, 2H), 1.49 (tt, *J* = 7.3, 7.3 Hz, 2H), 1.27–1.12 (m, 4H), 0.79 (t, *J* = 7.0 Hz, 3H); ^{13}C NMR (100 MHz, D_2O) δ 214.6, 175.2 (×3), 91.2, 87.7, 77.8, 76.3, 71.4, 67.9, 56.6, 43.7, 42.9, 41.0, 30.5, 22.9, 21.7, 13.2; HRMS (ESI, negative) calcd for $C_{18}H_{24}NO_9^-$ [(M−H)$^-$] 398.1457, found 398.1458.

(4*R,4a*R**,5a*S**,6*S**,8a*R**,8b*R**)-5a-(Carboxymethyl)-8-oxo-4-(2-oxoheptyl)octahydro-[1,3]dioxino[4',5':4,5]furo[2,3-*c*]pyrrole-6-carboxylic acid (1bs, (*2R**,*7S**)-TKM-86)**

A mixture of dibenzyl ester **S11bs** (0.87 mg, 0.0015 mmol) and Pd/C

(5%, 0.16 mg) in MeOH (0.250 mL) and THF (0.250 mL) was stirred under hydrogen atmosphere (balloon) at rt. After 20 h, the mixture was filtered through a pad of Celite and concentrated under reduced pressure. The residue was purified by trituration with water three times to give glutamate analog **1bs** (**(*2R**,7*S**)-TKM-86**, 0.59 mg, 98%) as a colorless oil: IR (ATR) 3504, 3263, 3193, 2931, 1723, 1709, 1701, 1689, 1586, 1412, 1291 cm^{-1}; ^{1}H NMR (400 MHz, D_2O) δ 4.86 (d, *J* = 7.0 Hz, 1H), 4.76 (d, *J* = 7.0 Hz, 1H), 4.56 (m, 1H), 4.50 (d, *J* = 2.5 Hz, 1H), 4.23 (s, 1H), 3.68 (m, 1H), 3.31 (s, 1H), 3.14 (dd, *J* = 17.1, 9.2 Hz, 1H), 2.97 (d, *J* = 15.7 Hz, 1H), 2.83 (dd, *J* = 17.1, 5.0 Hz, 1H), 2.77 (d, *J* = 15.7 Hz, 1H), 2.50 (t, *J* = 7.3 Hz, 2H), 1.48 (tt, *J* = 7.3, 7.3 Hz, 2H), 1.31–1.13 (m, 4H), 0.78 (t, *J* = 7.0 Hz, 3H); ^{13}C NMR (100 MHz, D_2O) δ 214.0, 175.5 (×3), 88.0, 85.4, 76.7, 75.4, 69.0, 68.6, 56.8, 42.8 (×2), 41.8, 30.5, 22.8, 21.7, 13.2; HRMS (ESI, negative) calcd for $C_{18}H_{24}NO_9^-$ [(M−H)$^-$] 398.1457, found 398.1458.

Benzyl (2*S,3*S**,3a*S**,6*R**,6a*R**)-6a-(2-(benzyloxy)-2-oxoethyl)-3-hydroxy-2-((*E*)-3-(4-methoxyphenyl)-3-oxoprop-1-en-1-yl)-4-oxohexahydro-2*H*-furo[2,3-*c*]pyrrole-6-carboxylate (S10c)**

To a stirred solution of alkene **7** (10.41 mg, 0.0231 mmol) and 4-methoxyphenyl vinyl ketone (18.7 mg, 0.115 mmol) in CH_2Cl_2 (0.400 mL) at rt was added Hoveyda-Grubbs catalyst second generation (0.7 mg, 0.001 mmol). After stirring at 50 °C in a sealed tube for 12 h, the mixture was concentrated under reduced pressure. The residue was purified by column chromatography on silica gel (60N, 600 mg, EtOAc/hexane = 6:4) to give enone **S10c** (8.92 mg, 66%) as a brown oil: IR (ATR) 3628, 3276, 2893, 1747, 1735, 1716, 1702, 1599, 1456, 1259, 1210 cm^{-1}; ^{1}H NMR (400 MHz, $CDCl_3$) δ 7.92 (d, *J* = 8.9 Hz, 2H), 7.37–7.26 (m, 10H), 7.16 (dd, *J* = 15.5, 1.6 Hz, 1H), 6.94–6.85 (m, 3H), 5.92 (brs, 1H), 5.14 (d, *J* = 11.9 Hz, 1H), 5.09 (d, *J* = 11.9 Hz, 1H), 5.07 (s, 2H), 4.66 (m, 1H), 4.62 (m, 1H), 4.41 (s, 1H), 3.85 (s, 3H), 3.29 (s, 1H), 3.21 (brs, 1H), 3.04 (d, *J* = 17.0 Hz, 1H), 2.99 (d, *J* = 17.0 Hz, 1H); ^{13}C NMR (100 MHz, $CDCl_3$) δ 188.3, 173.7, 170.2, 169.6, 163.6, 139.7, 135.2, 134.5, 131.1, 130.3, 128.8, 128.8 (×2), 128.7 (×2), 128.6 (×2), 128.5 (×2), 128.4 (×2), 127.6, 113.9 (×2), 87.3, 83.3, 76.7, 67.8, 66.9, 65.4, 58.4, 55.5, 39.4; HRMS (ESI, positive)

calcd for $C_{33}H_{31}NO_9Na^+$ [$(M+Na)^+$] 608.1891, found 608.1882.

Benzyl (4*R,4a*S**,5a*R**,6*R**,8a*S**,8b*S**)-5a-(2-(benzyloxy)-2-oxoethyl)-4-(2-(4-methoxyphenyl)-2-oxoethyl)-8-oxooctahydro-[1,3]dioxino[4',5':4,5]furo[2,3-*c*]pyrrole-6-carboxylate (S11cr), and benzyl (4*S**,4a*S**,5a*R**,6*R**,8a*S**,8b*S**)-5a-(2-(benzyloxy)-2-oxoethyl)-4-(2-(4-methoxyphenyl)-2-oxoethyl)-8-oxooctahydro-[1,3]dioxino[4',5':4,5]furo[2,3-*c*]pyrrole-6-carboxylate (S11cs)**

To a stirred solution of enone **S10c** (7.71 mg, 0.0132 mmol) in CH_2Cl_2 (0.500 mL) at rt were added paraformaldehyde (0.5 mg, 0.02 mmol) and MsOH (0.0017 mL, 0.026 mmol). After 2 h, the mixture was poured into water (0.5 mL) and extracted with EtOAc (6 × 0.5 mL). Combined extracts were dried over Na_2SO_4 and concentrated under reduced pressure to give a residue (8.22 mg), which is a mixture composed of *N*–hydroxymethylated 1,3-dioxanes **S12cr**, **S12cs** and 1,3-dioxanes **S11cr**, **S11cs** ((**S12cr**, **S12cr**)/(**S11cr**, **S11cs**) = 1:1.7).

The residue thus obtained, without purification, was dissolved in EtOH (0.500 mL). To the stirred mixture at rt was added ammonium hydroxide (28%, 0.0043 mL, 0.064 mmol). After 19 h, the mixture was concentrated under reduced pressure. The residue was purified by column chromatography on silica gel (60N, 600 mg, EtOAc/hexane = 4:6) to give an inseparable mixture of 1,3–dioxanes **S11cr** and **S11cs** (6.75 mg, **S11cr**/**S11cs** = 1.2:1) as a brown oil.

Purification of a portion of the mixture (5.39 mg) by HPLC (XTerra™ MS, 4.6 × 150 mm, MeOH/H_2O = 7:3, 1.0 mL/min, 40 °C, detected at 254 nm) gave diastereomerically pure 1,3-dioxanes **S11cr** (7*R**, 1.65 mg, 26%, t_R 8.8 min) and **S11cs** (7*S**, 1.26 mg, 19%, t_R 7.4 min).

Data for 1,3–dioxane S11cr (7*R):** retention time 8.8 min; IR (ATR) 3312, 2931, 1739, 1724, 1714, 1700, 1261, 1173, 1073, 1032, 822 cm^{-1}; ^1H NMR (400 MHz, $CDCl_3$) δ 7.88 (d, *J* = 8.9 Hz, 2H), 7.34–7.25 (m, 10H), 6.89 (d, *J* = 8.9 Hz, 2H), 5.90 (brs, 1H), 5.03 (d, *J* = 12.1 Hz, 1H), 5.02 (s, 2H), 4.97 (d, *J* = 6.7 Hz, 1H), 4.89 (d, *J* = 12.1 Hz,

1H), 4.69 (d, *J* = 6.7 Hz, 1H), 4.54 (d, *J* = 2.3 Hz, 1H), 4.40 (ddd, *J* = 6.9, 6.6, 2.3 Hz, 1H), 4.38 (s, 1H), 3.94 (dd, *J* = 2.3, 2.3 Hz, 1H), 3.84 (s, 3H), 3.42 (d, *J* = 17.4 Hz, 1H), 3.31 (dd, *J* = 17.2, 6.9 Hz, 1H), 3.28 (s, 1H), 3.20 (dd, *J* = 17.2, 6.6 Hz, 1H), 3.00 (d, *J* = 17.4 Hz, 1H); ^{13}C NMR (100 MHz, $CDCl_3$) δ 195.6, 172.7, 169.9, 169.5, 163.8, 135.6, 134.7, 130.6 (×2), 129.9, 128.7, 128.7 (×2), 128.6 (×2), 128.5 (×2), 128.2, 128.1 (×2), 113.8 (×2), 91.8, 87.7, 77.9, 76.5, 72.2, 67.7, 66.4, 64.9, 56.1, 55.5, 40.1, 39.7; HRMS (ESI, positive) calcd for $C_{34}H_{33}NO_{10}Na^+$ [(M+Na)$^+$] 638.1997, found 638.1988.

Data for 1,3-dioxane S11cs (7*S):** retention time 7.4 min; IR (ATR) 3390, 2921, 2849, 1741, 1722, 1714, 1674, 1601, 1261, 1177, 1073 cm^{-1}; ^{1}H NMR (400 MHz, $CDCl_3$) δ 7.88 (d, *J* = 8.9 Hz, 2H), 7.34–7.25 (m, 10H), 6.92 (d, *J* = 8.9 Hz, 2H), 5.93 (s, 1H), 5.04 (d, *J* = 12.1 Hz, 1H), 5.02 (d, *J* = 12.4 Hz, 1H), 4.99 (d, *J* = 12.4 Hz, 1H), 4.90 (d, *J* = 12.1 Hz, 1H), 4.85 (d, *J* = 6.5 Hz, 1H), 4.83 (d, *J* = 6.5 Hz, 1H), 4.67 (m, 1H), 4.54 (d, *J* = 2.3 Hz, 1H), 4.50 (s, 1H), 3.85 (s, 3H), 3.82 (dd, *J* = 2.3, 2.3 Hz, 1H), 3.40 (d, *J* = 17.4 Hz, 1H), 3.34 (dd, *J* = 16.4, 6.7 Hz, 1H), 3.30 (s, 1H), 3.18 (dd, *J* = 16.4, 7.3 Hz, 1H), 3.00 (d, *J* = 17.4 Hz, 1H); ^{13}C NMR (100 MHz, $CDCl_3$) δ 194.5, 172.7, 169.9, 169.4, 163.9, 135.5, 134.6, 130.4 (×2), 129.5, 128.7, 128.7 (×2), 128.6 (×2), 128.5 (×2), 128.3 (×2), 128.3, 113.9 (×2), 87.8, 86.7, 78.6, 75.7, 69.5, 67.7, 66.5, 64.7, 56.1, 55.5, 40.1, 38.7; HRMS (ESI, positive) calcd for $C_{34}H_{33}NO_{10}Na^+$ [(M+Na)$^+$] 638.1997, found 638.1987.

(4*S,4a*R**,5a*S**,6*S**,8a*R**,8b*R**)-5a-(Carboxymethyl)-4-(2-(4-methoxyphenyl)-2-oxoethyl)-8-oxooctahydro-[1,3]dioxino[4',5':4,5]furo[2,3-*c*]pyrrole-6-carboxylic acid (1cr, (2*R**,7*R**)-TKM-99)**

A mixture of dibenzyl ester **S11cr** (1.65 mg, 0.0027 mmol) and Pd/C (5%, 0.3 mg) in MeOH (0.250 mL) and THF (0.250 mL) was stirred under hydrogen atmosphere (balloon) at rt. After 22 h, the mixture was filtered through a pad of Celite and concentrated under reduced pressure to give a residue.

The residue and Pd/C (5%, 0.3 mg) were suspended in MeOH (0.250 mL) and THF (0.250 mL), and stirred under hydrogen atmosphere (balloon) at rt. After 14 h, the mixture was filtered through a pad of Celite and concentrated under reduced pressure to give a residue.

The residue and Pd/C (5%, 0.3 mg) were suspended in MeOH (0.250 mL) and THF (0.250 mL), and stirred under hydrogen atmosphere (balloon) at rt. After 16 h, the mixture was filtered through a pad of Celite and concentrated under reduced pressure to give a mixture mainly composed of glutamate analog **1cr** (**(*2R**,*7R**)-TKM-99**). Other products generated by over-reduction at the benzylic position were also obtained.

Purification of the mixture by HPLC (XTerra™ MS, 4.6 × 150 mm, CH_3CN (0.05% TFA)/H_2O (0.05% TFA) = 3:7, 1.0 mL/min, 40 °C, detected at 220 nm) gave glutamate analog **1cr** (**(*2R**,*7R**)-TKM-99**, 0.50 mg, 43%) as a colorless oil: retention time 13.8 min; IR (ATR) 3418, 3369, 2923, 2851, 1713, 1704, 1693, 1684, 1599, 1512, 1417 cm^{-1}; ^1H NMR (400 MHz, D_2O) δ 7.99 (d, *J* = 8.9 Hz, 2H), 7.05 (d, *J* = 8.9 Hz, 2H), 4.92 (d, *J* = 6.8 Hz, 1H), 4.77 (d, *J* = 6.8 Hz, 1H), 4.53 (m, 1H), 4.50 (d, *J* = 2.3 Hz, 1H), 4.23 (brs, 1H), 3.88–3.83 (m, 4H), 3.57 (dd, *J* = 17.3, 8.9 Hz, 1H), 3.32 (s, 1H), 3.26 (dd, *J* = 17.3, 3.9 Hz, 1H), 2.92 (d, *J* = 14.9 Hz, 1H), 2.77 (d, *J* = 14.9 Hz, 1H); ^{13}C NMR (100 MHz, D_2O) δ 199.8, 175.2 (×2), 173.7, 164.0, 131.1 (×2), 129.4, 114.1 (×2), 91.4, 87.4, 77.7, 76.5, 72.1, 66.7, 56.6, 55.6, 40.3, 39.7; HRMS (ESI, negative) calcd for $C_{20}H_{20}NO_{10}^-$ [$(M-H)^-$] 434.1093, found 434.1091.

(4*R,4a*R**,5a*S**,6*S**,8a*R**,8b*R**)-5a-(Carboxymethyl)-4-(2-(4-methoxyphenyl)-2-oxoethyl)-8-oxooctahydro-[1,3]dioxino[4',5':4,5]furo[2,3-*c*]pyrrole-6-carboxylic acid (1cs, (*2R**,*7S**)-TKM-99)**

A mixture of dibenzyl ester **S11cs** (1.26 mg, 0.0010 mmol) and Pd/C (5%, 0.2 mg) in MeOH (0.250 mL) and THF (0.250 mL) was stirred under hydrogen atmosphere (balloon) at rt. After 22 h, the mixture was filtered through a pad of Celite and concentrated under reduced pressure to give a residue.

The residue and Pd/C (5%, 0.3 mg) were suspended in MeOH (0.250 mL) and THF (0.250 mL), and stirred under hydrogen atmosphere (balloon) at rt. After 14 h, the mixture was filtered through a pad of Celite and concentrated under reduced pressure to give a residue.

The residue and Pd/C (5%, 0.3 mg) were suspended in MeOH (0.250 mL) and THF (0.250 mL), and the suspension stirred under hydrogen atmosphere (balloon) at rt. After 16 h, the mixture was filtered through a pad of Celite and the filtrate concentrated under reduced pressure to give a mixture mainly composed of glutamate analog **1cs** (**(*2R**,*7S**)-TKM-99**). Other products generated by over-reduction at the benzylic position were also obtained.

Purification of the mixture by HPLC (XTerra™ MS, 4.6 × 150 mm, CH_3CN (0.05% TFA)/ H_2O (0.05% TFA) = 3:7, 1.0 mL/min, 40 °C, detected at 220 nm) gave glutamate analog **1cs** (**(*2R**,*7S**)-TKM-99)**, 0.50 mg, 56%) as a colorless oil: retention time 13.1 min; IR (ATR) 3428, 3294, 2930, 2852, 1733, 1724, 1714, 1685, 1542, 1471, 1291 cm^{-1}; ^1H NMR (400 MHz, D_2O) δ 7.96 (d, *J* = 8.9 Hz, 2H), 7.04 (d, *J* = 8.9 Hz, 2H), 4.93 (d, *J* = 6.9 Hz, 1H), 4.84–4.61 (m, 2H), 4.54 (s, 1H), 4.23 (brs, 1H), 3.85 (s, 3H), 3.79 (s, 1H), 3.63 (dd, *J* = 16.9, 8.6 Hz, 1H), 3.38 (dd, *J* = 16.8, 5.3 Hz, 1H), 3.37 (s, 1H), 2.92 (m, 1H), 2.82 (m, 1H); ^{13}C NMR (100 MHz, D_2O) δ 199.1, 175.0 (×2), 173.6, 164.0, 130.9 (×2), 129.1, 114.2 (×2), 87.2, 85.6, 77.6, 75.0, 69.6, 66.9, 56.8, 55.6, 40.2, 37.6; HRMS (ESI, negative) calcd for $C_{20}H_{20}NO_{10}^-$ [$(M-H)^-$] 434.1093, found 434.1094.

Methyl (2*R,3*R**,3a*R**,6*S**,6a*S**)-3-hydroxy-6a-(2-methoxy-2-oxoethyl)-4-oxo-2-((*E*)-3-oxobut-1-en-1-yl)hexahydro-2*H*-furo[2,3-*c*]pyrrole-6-carboxylate (13)**

To a stirred solution of homoallylic alcohol **5** (7.87 mg, 0.0263 mmol) and 3-buten-2-one (0.0107 mL, 0.1316 mmol) in CH_2Cl_2 (0.5 mL) at rt was added Hoveyda-Grubbs catalyst second generation (0.3 mg, 0.0005 mmol). After stirring at 50 °C in a sealed tube for 20 h, the mixture was concentrated under reduced pressure. The residue was purified by

column chromatography on silica gel (60N, 600 mg, EtOAc) to give enone **13** (8.06 mg, 90%) as a brown oil: IR (ATR) 3627, 2923, 1747, 1733, 1718, 1707, 1544, 1457, 1373, 1171, 1093 cm^{-1}; ^{1}H NMR (400 MHz, $CDCl_3$) δ 7.11 (brs, 1H), 6.73 (dd, *J* = 16.0, 4.1 Hz, 1H), 6.28 (d, *J* = 16.0 Hz, 1H), 4.57–4.53 (m, 2H), 4.36 (s, 1H), 3.73 (s, 3H), 3.66 (s, 3H), 3.28 (s, 1H), 3.03 (d, *J* = 17.5 Hz, 1H), 2.98 (d, *J* = 17.5 Hz, 1H), 2.25 (s, 3H); ^{13}C NMR (100 MHz, $CDCl_3$) δ 198.3, 174.3, 170.9, 170.2, 140.4, 132.2, 87.1, 82.7, 76.6, 65.5, 58.7, 52.8, 52.1, 39.1, 27.3; HRMS (ESI, positive) calcd for $C_{15}H_{19}NO_8Na^+$ [$(M+Na)^+$] 364.1003, found 364.1003.

(*2R,4*R**,4a*R**,5a*S**,6*S**,8a*R**,8b*R**)-5a-(Carboxymethyl)-2-hydroxy-4-methoxy-2-methyl-8-oxodecahydropyrano[2',3':4,5]furo[2,3-*c*]pyrrole-6-carboxylic acid (2, TKM-15)**

To a stirred solution of enone **13** (8.11 mg, 0.0238 mmol) in MeOH (2 mL) at rt was added aqueous LiOH (1 M, 1.31 mL, 1.31 mmol). After 70 h, to the mixture was added Amberlyst 15 ion-exchange resin (wet, 2 g). After stirring at rt for 1 h, insoluble materials were removed by filtration. The filtrate was concentrated by blowing of air at 50 °C. The residue was purified by column chromatography on reversed-phase silica gel (DM1020T, 500 mg, water) to give glutamate analog **2** (**TKM-15**, 4.35 mg, 53%) as a brown oil: IR (ATR) 3411, 3310, 2950, 2906, 2841, 1722, 1705, 1694, 1442, 1408, 1216 cm^{-1}; ^{1}H NMR (400 MHz, D_2O) δ 4.59 (s, 1H), 4.54 (m, 1H), 4.17 (m, 1H), 3.87 (ddd, *J* = 12.5, 4.6, 4.6 Hz, 1H), 3.37 (s, 1H), 3.29 (s, 3H), 3.18 (d, *J* = 16.2 Hz, 1H), 2.95 (d, *J* = 16.2 Hz, 1H), 1.86 (dd, *J* = 12.5, 4.6 Hz, 1H), 1.64 (dd, *J* = 12.5, 12.5 Hz, 1H), 1.40 (s, 3H); ^{13}C NMR (100 MHz, D_2O) δ 175.4, 173.6, 171.8, 98.0, 85.3, 74.7, 74.5, 71.9, 65.2, 56.9, 54.7, 41.3, 34.1, 28.2; HRMS (ESI, negative) calcd for $C_{14}H_{18}NO_9^-$ [$(M-H)^-$] 344.0987, found 344.0984.

[CONFIGURATIONAL AND CONFORMATIONAL ANALYSIS OF CYCLIC HEMIACETAL 2 (TKM-15)]

The planar structure of **TKM-15** (**2**) was analyzed using 1D and 2D NMR data. As shown in Figure S1A, formation of the hemiacetal ring was confirmed by HMBC correlations from H14 to C8/C9, in addition to the C4-C8 spin system established by COSY, TOCSY, and HSQC spectra.

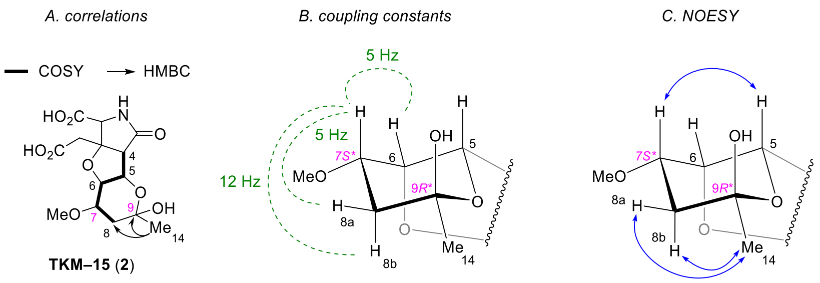

Figure S1. Structure analysis of hemiacetal product **TKM-15** (**2**). (A) COSY and HMBC correlations observed. (B) $J_{H,H}$ values. (C) NOESY correlations observed.

The relative configuration of the hemiacetal ring in **TKM-15** (**2**) was established to be (7*S**,9*R**) as shown in Figures S1B and S1C. The 7*S** stereochemistry was determined by the vicinal coupling constants ($^3J_{H6,H7}$ = 5 Hz, $^3J_{H7,H8a}$ = 5 Hz, $^3J_{H7,H8b}$ = 12 Hz) and NOE correlations (H5/H7). Additional NOE correlations at H8a/H14 and H8b/H14 were observed in the same intensity, which suggested equatorial orientation of the 14-Me group, since the axial orientation of the 14-Me group should exhibit stronger NOE effects with H8a than H8b.

Oxa-Michael reaction and acetalization are known as thermodynamically controlled reactions. Therefore, the analysis that the structure of the product is the one illustrated in Figure S1 is consistent with its thermodynamic stability; the 7-methoxy and the 9-hydroxy groups take equatorial and axial orientations, respectively.

This analysis was supported by density functional theory (DFT) calculation[1] of the possible diastereomers, **S2A**, **S2B**, **S2C**, and **S2D** (for structures, see Figure S2). The calculations were performed with Spartan '18 (Wavefunction, Irvine, CA, U.S.A.).[2] The theoretical ^{13}C NMR shifts for four hemiacetals **S2A**, **S2B–S2D** were independently obtained by the calculation sequence; 1) conformational search with MMFF94,[3] 2) structural optimizations with HF/3-21G level, 3) energy calculations at ωB97X-D/6-31G* level, 4) structural optimizations with ωB97X-D/6-31G* level, 5) energy calculations at ωB97X-V/6-311+G(2df,2p)[6-311+G*] level fixing the geometries to generate Boltzmann distribution, 6) empirically corrected calculations of the ^{13}C NMR chemical shifts at ωB97X-D/6-31G* level with ωB97X-D/6-31G* model,[4] and 7) correction of the ^{13}C NMR shift values based on the Boltzmann weighting. Parameters regarding solvents (D_2O) were not added in these calculations.

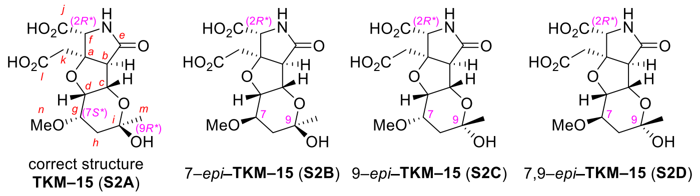

Figure S2. A set of possible diastereomers for **TKM-15** (**2**) obtained in the alkali-mediated transformation. The configurations were analyzed first by NMR, and then confirmed by NMR calculation (see text).

The experimental ^{13}C values for **TKM-15** (**2**) (2_{obs}), and the calculated ^{13}C values for **S2A** ($S2A_{calc}$), **S2B** ($S2B_{calc}$), **S2C** ($S2C_{calc}$), and **S2D** ($S2D_{calc}$) are summarized in Table S1. The calculated ^{13}C NMR shifts of **S2A** were consistent well with the experimental values for **TKM-15** (**2**); the root mean square deviation (RMS) value of correct structure **S2A** was the smallest among those of the candidates (**S2A**, 1.79 ppm; 7-*epi* stereoisomer **S2B**, 2.29 ppm; 9-*epi* stereoisomer **S2C**, 2.73 ppm; 7,9-*epi* stereoisomer **S2D**, 2.71 ppm). Furthermore, analysis using DP4 probability statistics[1a, 5] revealed that **S2A** was by far the highest probability (**S2A**, 98.4%; 7-*epi* stereoisomer **S2B**, 1.6%;

9-*epi* stereoisomer **S2C**, 0.0%; 7,9-*epi* stereoisomer **S2D**, 0.0%). The relative configuration of the hemiacetal **TKM-15** (**2**) was thus unambiguously assigned to be (7*S**,9*R**).

Table S1. Comparison of experimental ^{13}C shifts of **TKM-15** (**2**), and calculated ^{13}C shifts for four candidates **S2A** and **S2B**-**S2D**.

Position[a]	Experimental for **2** (2_{obs})[b]	Calculated[c]			
		**S2A** ($S2A_{calc}$)	**S2B** ($S2B_{calc}$)	**S2C** ($S2C_{calc}$)	**S2D** ($S2D_{calc}$)
a	85.3	89.3	89.7	90.3	90.0
b	54.7	55.0	55.4	54.9	55.0
c	74.5	76.8	73.3	77.3	76.9
d	74.7	77.8	77.4	80.5	80.3
e	171.8	172.4	172.3	172.6	173.4
f	65.2	65.2	65.3	65.8	67.9
g	71.9	72.2	76.7	74.2	75.1
h	34.1	34.6	30.3	35.7	31.9
i	98	98.3	97.5	99.6	98.3
j	173.6	173.0	173.0	173.0	173.1
k	41.3	39.1	39.6	39.6	41.2
l	175.4	174.1	173.4	174.8	174.8
m	28.2	30.1	28.4	23.4	24.1
n	56.9	55.3	56.3	57.2	55.9
RMS/ppm		1.79	2.29	2.73	2.71
DP4/%		98.4	1.6	0.0	0.0

[a] For numbering, see Figure S2.

[b] Experimental ^{13}C NMR data were collected at 100 MHz in D_2O.

[c] Calculated ^{13}C NMR data were obtained employing ωB97X-V/6-311+G(2df,2p)-[6-311+G*]//ωB97X-D/6-31G* model. For detail, see text.

Final geometries used for NMR calculations (ωB97X-D/6-31G*)

(7*S**,9*R**)-**S2A**, M0003:M0001

I	Atom	X	Y	Z
1	C	-0.0834830380	0.3767716184	-1.2526613461
2	C	-1.5450285438	0.0454700098	-0.9040640582
3	C	-1.4813492209	-0.4396060073	0.5358788120
4	C	-0.1634221875	-1.2205914809	0.5331578063
5	O	0.6987249970	-0.4371176551	-0.3295136230
6	C	-1.8967348930	-1.1276022031	-1.8091367097
7	N	-0.9024365382	-1.2544474283	-2.7206775027
8	C	0.1460043413	-0.2401057358	-2.6479918958
9	C	0.4813200949	-1.3591713949	1.9067201077
10	C	0.5105906006	-0.0133678173	2.6130833183
11	C	-0.8851933874	0.5757794216	2.6747213054
12	O	-1.3857903791	0.7526375059	1.3400257309
13	O	-2.8718139042	-1.8403974910	-1.6962683959
14	C	-0.0324619572	0.6748488315	-3.8317473612
15	O	-0.2289961984	1.8601504461	-3.8594869593
16	O	0.0528684090	-0.0675998885	-4.9630097929
17	C	0.3294768314	1.8174011960	-1.0330694446
18	C	1.7632014304	2.0230436535	-1.4097977801
19	O	2.3940406481	1.3746440538	-2.2069468001
20	O	2.2891017040	3.0910848460	-0.7719378675
21	H	-2.2341515099	0.8674886208	-1.0207067060
22	H	-2.3286657512	-1.0406774696	0.8178815971
23	H	-0.3343722085	-2.1899371744	0.0859050586
24	C	-0.9568271203	1.9449268400	3.3261780872
25	O	-1.6885674526	-0.3855069035	3.3545618501
26	O	1.8465801997	-1.7790139472	1.8432308072
27	C	2.1310737207	-2.9876205442	1.1213464781
28	H	1.1349842285	-0.6576054906	-2.6817147901
29	H	-0.1123614944	-2.0599059736	2.4787632790
30	H	-0.9195843698	-1.9379908478	-3.4459928678
31	H	0.8998984628	-0.1423077916	3.6116397797

32	H	1.1583687965	0.6391419961	2.0487865431
33	H	-0.0485753530	0.4678469163	-5.7636012629
34	H	0.1830304765	2.0475304933	0.0066406569
35	H	-0.2605008089	2.4890167878	-1.6379880999
36	H	3.2053470943	3.2594258649	-1.0368149817
37	H	-1.9711334026	2.3212846088	3.2671461374
38	H	-0.6582024539	1.8705522201	4.3629645950
39	H	-0.3065685911	2.6358784725	2.8090614131
40	H	-2.5997390030	-0.0741639123	3.4484945398
41	H	2.0002498713	-2.8457873875	0.0570004254
42	H	1.5068089863	-3.8109914777	1.4559275256
43	H	3.1642888738	-3.2234083810	1.3240123920

(7*S**,9*R**)-**S2A,** M0003:M0002

ATOM		X	Y	Z
1	C	0.1315534715	0.2777574217	-1.2231962281
2	C	-1.3031732893	-0.1323065890	-0.8460033275
3	C	-1.1837982305	-0.5971056581	0.5935098011
4	C	0.1853071533	-1.2807237047	0.5867347336
5	O	0.9817677899	-0.4223489154	-0.2710500064
6	C	-1.6281969385	-1.3072518748	-1.7488310266
7	N	-0.6389554531	-1.4062733900	-2.6739380065
8	C	0.3922578921	-0.3962305387	-2.6062878719
9	C	0.8379901268	-1.3750248248	1.9588072769
10	C	0.7978779791	-0.0218281270	2.6499226310
11	C	-0.6304946539	0.4860342404	2.7113189831
12	O	-1.1505410724	0.6130833511	1.3802018144
13	O	-2.5802557162	-2.0489529989	-1.6352527584
14	C	0.3938342574	0.5323353738	-3.7931912993
15	O	1.1268212732	1.4847442236	-3.8942349246
16	O	-0.4344723717	0.1401652335	-4.7734044243
17	C	0.4625318040	1.7509903015	-1.0817208927
18	C	-0.4280795051	2.6467969852	-1.8774832054
19	O	-1.3723188149	2.3110079746	-2.5528062226
20	O	-0.0740072011	3.9377309342	-1.7297438152
21	H	-2.0233733704	0.6558145412	-0.9713049166

22	H	-1.9841883193	-1.2477185768	0.9021010871
23	H	0.0891016434	-2.2567703298	0.1309136411
24	C	-0.7763717208	1.8570769156	3.3464405048
25	O	-1.3713904801	-0.5088966467	3.4139258379
26	O	2.2204728766	-1.7343023758	1.8925266466
27	C	2.5292078464	-3.0272778556	1.3508375099
28	H	1.3834858250	-0.8225419837	-2.5414029551
29	H	0.2805264600	-2.0973031720	2.5412686029
30	H	-0.7156042252	-2.0346360899	-3.4439031267
31	H	1.1962576761	-0.1175527244	3.6488051148
32	H	1.4054538096	0.6606555344	2.0762758327
33	H	-0.4351445109	0.7588520177	-5.5186706729
34	H	1.4857670227	1.9459058535	-1.3645102079
35	H	0.3300239630	1.9940452927	-0.0378081544
36	H	-0.6343159321	4.5320515929	-2.2503121332
37	H	-1.8114427232	2.1730402232	3.2914107523
38	H	-0.4663851970	1.8136710848	4.3817496121
39	H	-0.1716889927	2.5788251002	2.8158527571
40	H	-2.2990431316	-0.2494678795	3.5049081057
41	H	2.3439947687	-3.0665181255	0.2848011822
42	H	1.9583865726	-3.8105902829	1.8407762751
43	H	3.5806216385	-3.1889615313	1.5319674742

(7*S**,9*R**)-**S2A,** M0003:M0003

ATOM		X	Y	Z
1	C	0.0871918796	0.2366940958	-1.2524271021
2	C	-1.3476984061	-0.1716680592	-0.8783909851
3	C	-1.2347708690	-0.6195122515	0.5677429146
4	C	0.1265535190	-1.3205058545	0.5651008532
5	O	0.9315624330	-0.4919791640	-0.3142119970
6	C	-1.6462454186	-1.3746117850	-1.7565346129
7	N	-0.6456735611	-1.4797660970	-2.6702804417
8	C	0.3425999756	-0.4216907785	-2.6382789470
9	C	0.7840340142	-1.4028503205	1.9360329826
10	C	0.7546469080	-0.0439928188	2.6164711747
11	C	-0.6698798434	0.4744440621	2.6780904550

12	O	-1.1914766714	0.5954055357	1.3466341760
13	O	-2.5817020986	-2.1336380726	-1.6261815141
14	C	0.1978880383	0.4396555941	-3.8640819799
15	O	-0.5203454863	0.2385834634	-4.7994747369
16	O	1.1087065039	1.4515103339	-3.8398422115
17	C	0.4396865959	1.6998605751	-1.0735356306
18	C	-0.4042620406	2.6292775944	-1.8753964077
19	O	-1.3802131452	2.3433785170	-2.5264239859
20	O	0.0304235046	3.9023144689	-1.7620353289
21	H	-2.0718022953	0.6094423745	-1.0264316861
22	H	-2.0414077160	-1.2604011065	0.8800597305
23	H	0.0141495163	-2.3028428094	0.1279860526
24	C	-0.8050241838	1.8514301659	3.3028097496
25	O	-1.4158530480	-0.5097659657	3.3894954174
26	O	2.1642031004	-1.7706406238	1.8695506431
27	C	2.4651682075	-3.0627196885	1.3206958158
28	H	1.3552328293	-0.7973362348	-2.6174705011
29	H	0.2240420747	-2.1162588678	2.5268474323
30	H	-0.6951994431	-2.1393260762	-3.4168020766
31	H	1.1552620280	-0.1350489384	3.6148528931
32	H	1.3660040766	0.6301396619	2.0368336010
33	H	1.0720263701	1.9909605212	-4.6438389870
34	H	1.4762254264	1.8740435734	-1.3143735982
35	H	0.2752201040	1.9263611961	-0.0305602086
36	H	-0.5346089486	4.5236099250	-2.2450444748
37	H	-1.8373425076	2.1754785438	3.2442655581
38	H	-0.4962646955	1.8130798030	4.3386507170
39	H	-0.1936652430	2.5643183373	2.7679263481
40	H	-2.3419741985	-0.2445519295	3.4793069685
41	H	2.2817018407	-3.0931229532	0.2542403080
42	H	1.8878923488	-3.8449646095	1.8046756871
43	H	3.5149885250	-3.2327933376	1.5033479354

(7*S**,9*R**)-**S2A,** M0003:M0004

ATOM		X	Y	Z
1	C	0.1208451071	0.2183212192	-1.3756846676

2	C	-1.3155567113	-0.2236266224	-1.0351429139
3	C	-1.2183258270	-0.6791461645	0.4088445083
4	C	0.1684730503	-1.3211246394	0.4436132058
5	O	0.9582385032	-0.4119920605	-0.3673561574
6	C	-1.5978208227	-1.4081398771	-1.9391591826
7	N	-0.5735221741	-1.5077700882	-2.8239716591
8	C	0.4525509098	-0.4937000247	-2.7249250933
9	C	0.7587961501	-1.4120257392	1.8354109543
10	C	0.7239334215	-0.0380400786	2.4945897285
11	C	-0.7117997271	0.4645081583	2.5176350357
12	O	-1.2264438256	0.5428071663	1.1839831946
13	O	-2.5488952360	-2.1553347636	-1.8545951316
14	C	0.5245914716	0.3982648899	-3.9373343454
15	O	1.2343807664	1.3699816314	-4.0175968689
16	O	-0.2077028329	-0.0559016225	-4.9671364976
17	C	0.3939733691	1.7066576259	-1.2734941297
18	C	-0.4869246300	2.5369579739	-2.1465960301
19	O	-1.3826316163	2.1384639350	-2.8529699244
20	O	-0.1876723318	3.8455853886	-2.0357134127
21	H	-2.0512553968	0.5470269270	-1.1771795793
22	H	-2.0119252536	-1.3426592796	0.7081734757
23	H	0.1490775629	-2.2997122905	-0.0134134060
24	C	-0.8695024721	1.8558988089	3.1046567677
25	O	-1.4506492706	-0.5074676035	3.2562312519
26	O	2.0902083838	-1.9161052001	1.6821990239
27	C	2.6868647049	-2.4806547387	2.8604008203
28	H	1.4375089860	-0.9202408043	-2.5933436925
29	H	0.1545144864	-2.0985440390	2.4142577661
30	H	-0.6047703770	-2.1532997269	-3.5826277720
31	H	1.0902227597	-0.0859757858	3.5103554871
32	H	1.3371782214	0.6315474635	1.9117591382
33	H	-0.1597818421	0.5332386830	-5.7343890741
34	H	1.4224598254	1.9264743959	-1.5163217338
35	H	0.2044076369	1.9830328705	-0.2466846228
36	H	-0.7412764967	4.3947920769	-2.6102229586
37	H	-1.9056245079	2.1644737085	3.0307921862

38	H	-0.5684404859	1.8498277009	4.1436007595
39	H	-0.2641718175	2.5621938709	2.5543809317
40	H	-2.3846001276	-0.2607229750	3.3109667866
41	H	2.0701933285	-3.2733087731	3.2726468978
42	H	2.8540066590	-1.7344869216	3.6279903533
43	H	3.6368684785	-2.8900746755	2.5533705799

(7*S**,9*R**)-**S2A**, M0003:M0005

I	Atom	X	Y	Z
1	C	-0.2812499823	-0.1071453205	-1.3317450215
2	C	-1.6219736791	0.2959703531	-0.7150505563
3	C	-1.5073531369	-0.0641239289	0.7504848383
4	C	-0.5631054976	-1.2856410866	0.7435834013
5	O	0.0813173400	-1.2870485104	-0.5554726475
6	C	-2.6168793949	-0.6320666721	-1.4041012646
7	N	-2.0223144044	-1.1004151553	-2.5270034343
8	C	-0.6391387900	-0.6826602543	-2.7164346358
9	C	0.4730026032	-1.2054447987	1.8764656851
10	C	-0.1983819655	-0.6019882411	3.1059657709
11	C	-0.6560168964	0.8358877237	2.8229926962
12	O	-0.8685543199	1.0351231834	1.4185892536
13	O	-3.7277030901	-0.9039038686	-1.0008214842
14	C	-0.5792702184	0.2855338162	-3.8692290295
15	O	-0.0670979976	1.3724743850	-3.9134399723
16	O	-1.1890344021	-0.2664159696	-4.9454475019
17	C	0.8198480532	0.9262772205	-1.2167178298
18	C	2.0560175483	0.4933332865	-1.9349091763
19	O	2.1643304558	-0.4124542433	-2.7241429461
20	O	3.1125356512	1.2709392458	-1.5959927229
21	H	-1.8878369033	1.3336541529	-0.8494566859
22	H	-2.4662992058	-0.2756886970	1.1950792848
23	H	-1.1324017064	-2.1984640914	0.8390007684
24	C	0.3526204122	1.8916554336	3.2343845184
25	O	-1.8969061825	0.9722218509	3.5101127638
26	O	1.5880263553	-0.3758340928	1.5249251297

27	C	2.7109269797	-1.0558514020	0.9156535615
28	H	0.0225629885	-1.5046444217	-2.9242794753
29	H	0.8264600151	-2.2040573550	2.1059256519
30	H	-2.4849893064	-1.7018855289	-3.1722075553
31	H	-1.0750825490	-1.1813983438	3.3645118894
32	H	0.4815264379	-0.6113751595	3.9455224004
33	H	-1.1571285421	0.3121408935	-5.7213608178
34	H	1.0391750423	1.0419716644	-0.1685356888
35	H	0.5177995743	1.8734044545	-1.6349803432
36	H	3.9051477412	1.0440703763	-2.1043113200
37	H	1.2964920534	1.6633364910	2.7683761461
38	H	0.4550428520	1.8933210977	4.3116450885
39	H	0.0143080179	2.8663374634	2.9028618110
40	H	-2.2551090878	1.8624999875	3.3886011770
41	H	2.3909334460	-1.6123310205	0.0501820946
42	H	3.1862668064	-1.7190835915	1.6296662686
43	H	3.3994868844	-0.2802313269	0.6211099096

(7*S**,9*R**)-**S2A**, M0003:M0006

I	Atom	X	Y	Z
1	C	0.3173399356	0.1378331367	-1.4334478176
2	C	-0.1078752716	1.5779086634	-1.0911581580
3	C	-0.5748678499	1.4816703343	0.3525698735
4	C	-1.2678680260	0.1173236553	0.3602277151
5	O	-0.3972451456	-0.6910493874	-0.4707766589
6	C	-1.3130540641	1.8481514906	-1.9815128215
7	N	-1.4045001421	0.8283153228	-2.8678878082
8	C	-0.3264954582	-0.1559824533	-2.8049423642
9	C	-1.3782380312	-0.4918027334	1.7429566805
10	C	-0.0030084531	-0.5204691121	2.3986409510
11	C	0.5455044655	0.8971933046	2.4585726185
12	O	0.6381818094	1.4443570920	1.1374245641
13	O	-2.0741346795	2.7869099649	-1.8743853303
14	C	0.5375160081	0.0579719455	-4.0207909728
15	O	1.7022382254	0.3462526706	-4.0921943838

16	O	-0.2298071833	-0.1130146118	-5.1255800204
17	C	1.7916792711	-0.1647838050	-1.2609244618
18	C	2.0897704362	-1.5842937657	-1.6297797511
19	O	1.4739396143	-2.2665301162	-2.4096896366
20	O	3.2032276086	-2.0300139446	-1.0063827408
21	H	0.6652265335	2.3197217799	-1.2164554683
22	H	-1.2154522684	2.2941521211	0.6503755986
23	H	-2.2478069179	0.1839570209	-0.0877927935
24	C	1.9429368273	0.9974233114	3.0435908109
25	O	-0.4032596306	1.6463387850	3.2165501149
26	O	-1.9348058544	-1.7990539688	1.5694600253
27	C	-2.5371584956	-2.3885420913	2.7326017706
28	H	-0.6849230118	-1.1684683827	-2.8075081352
29	H	-2.0377518322	0.1303519321	2.3335267011
30	H	-2.1098623274	0.7858862131	-3.5710001082
31	H	-0.0605406227	-0.9106736297	3.4047720739
32	H	0.6445932051	-1.1393720343	1.7978146751
33	H	0.2724927772	0.0085835923	-5.9445822168
34	H	2.0444484965	0.0104761794	-0.2312165646
35	H	2.3968812407	0.4650813253	-1.8950250386
36	H	3.4307821129	-2.9331956261	-1.2719797861
37	H	2.2835464246	2.0246597741	2.9929626091
38	H	1.9296675779	0.6718971190	4.0748683551
39	H	2.6275865576	0.3829304659	2.4767361089
40	H	-0.1308573399	2.5714590698	3.2933409565
41	H	-3.3084765700	-1.7459539473	3.1455404705
42	H	-1.8081602019	-2.5977071967	3.5064184856
43	H	-2.9814097499	-3.3158994631	2.4060618785

(7*S**,9*R**)-**S2A,** M0003:M0007

ATOM		X	Y	Z
1	C	-0.3652723446	0.1166362923	-1.3433452839
2	C	0.0052831040	1.4686637178	-0.7352998428
3	C	-0.3060209542	1.3326728635	0.7368309493
4	C	-1.4863139106	0.3398339998	0.7674560959
5	O	-1.4914957217	-0.3172987359	-0.5270384378

6	C	-0.9559581036	2.4372861627	-1.4038411206
7	N	-1.4423313323	1.8260327568	-2.5152486086
8	C	-1.0110923452	0.4621492648	-2.7150871185
9	C	-1.3369334534	-0.6868892450	1.9024986963
10	C	-0.7112608789	0.0054791392	3.1089615225
11	C	0.6977410919	0.5143233442	2.7726897056
12	O	0.8388527896	0.7287202014	1.3629447315
13	O	-1.2436414391	3.5454237762	-1.0068303028
14	C	-0.0832345826	0.2805217700	-3.8881767294
15	O	0.3545200222	-0.7944383920	-4.2172115540
16	O	0.1596527606	1.4107851748	-4.5659048308
17	C	0.6926520424	-0.9690446560	-1.2972580929
18	C	2.0017542271	-0.5794409038	-1.8955272587
19	O	2.3036950845	0.4952841773	-2.3591999947
20	O	2.8745993668	-1.6060945065	-1.8496916517
21	H	1.0254841354	1.7553948762	-0.9198358342
22	H	-0.5386217907	2.2780615648	1.2005321816
23	H	-2.4196229574	0.8721353777	0.8793712435
24	C	1.8025327762	-0.4540803471	3.1510016316
25	O	0.8167450183	1.7603313389	3.4535343029
26	O	-0.4902766551	-1.7835179474	1.5290166162
27	C	-1.1700225836	-2.9303792856	0.9706898428
28	H	-1.8387028338	-0.2193872024	-2.8487236663
29	H	-2.3168531759	-1.0663924615	2.1689601369
30	H	-2.0121491699	2.3138361577	-3.1705268855
31	H	-1.3132650393	0.8592781443	3.3921063474
32	H	-0.6651479223	-0.6757252049	3.9466221130
33	H	0.7975219115	1.2743582220	-5.2819214275
34	H	0.3467759966	-1.8531356533	-1.8139086578
35	H	0.8378125722	-1.2120432843	-0.2546209457
36	H	3.7270382346	-1.3738223435	-2.2464128474
37	H	1.5989300516	-1.4070017602	2.6910559715
38	H	1.8407338290	-0.5569794399	4.2276906469
39	H	2.7536200586	-0.0782488840	2.7918223170
40	H	1.6868112581	2.1505220476	3.2904749066
41	H	-1.7099305214	-2.6537458089	0.0802962177

42	H	-1.8518538615	-3.3587542624	1.6976757205
43	H	-0.4027547548	-3.6513100451	0.7333791944

(7*S**,9*R**)-**S2A**, M0003:M0008

I	Atom	X	Y	Z
1	C	0.0840193440	0.2974278596	-1.2986902749
2	C	-1.4084749485	-0.0374565970	-1.1217872690
3	C	-1.5399098640	-0.3642863606	0.3594026457
4	C	-0.2244264587	-1.1055988318	0.6221620653
5	O	0.7377353475	-0.3949696410	-0.1963340664
6	C	-1.6152574064	-1.3091199605	-1.9322778396
7	N	-0.4893212764	-1.5171916406	-2.6584880922
8	C	0.5082780605	-0.4576869283	-2.5773702084
9	C	0.2310707173	-1.0704182303	2.0748785508
10	C	0.1657071194	0.3514721115	2.6076608990
11	C	-1.2354366976	0.9044162296	2.4291554599
12	O	-1.5724760293	0.9099949687	1.0316578298
13	O	-2.5942496139	-2.0237962378	-1.8921288975
14	C	0.5000772064	0.3213328619	-3.8716307435
15	O	0.5372199970	1.5093997610	-4.0453768744
16	O	0.4926102876	-0.5513831359	-4.9120964703
17	C	0.4291274705	1.7669768380	-1.1580904972
18	C	1.9030994021	1.9805458343	-0.9987405595
19	O	2.4265181078	2.7842045865	-0.2767870220
20	O	2.6326078752	1.1808464377	-1.8251295996
21	H	-2.0856853157	0.7491079124	-1.4166518227
22	H	-2.4108093400	-0.9545745667	0.5869597638
23	H	-0.3197325430	-2.1217215089	0.2662039588
24	C	-1.4068319543	2.3378910864	2.8971215490
25	O	-2.1022251878	0.0041473419	3.1145528908
26	O	1.5928893032	-1.4759515908	2.2341125314
27	C	1.9314092351	-2.8098686618	1.8246001456
28	H	1.5039180888	-0.8289568322	-2.4146109618
29	H	-0.4316874577	-1.7061107890	2.6465198548
30	H	-0.4156133286	-2.2501654795	-3.3295379047

31	H	0.4276749885	0.3526937363	3.6546998200
32	H	0.8710559909	0.9486107188	2.0510197128
33	H	0.5177901347	-0.1009078017	-5.7688657495
34	H	-0.0536962157	2.1212157045	-0.2650841194
35	H	0.0938330066	2.3329458616	-2.0119379530
36	H	3.5845842551	1.3160598689	-1.7103279308
37	H	-2.4122180838	2.6738124144	2.6732448322
38	H	-1.2340623165	2.3959476957	3.9631023188
39	H	-0.7083040447	2.9806301230	2.3811470441
40	H	-3.0213228117	0.3032127741	3.0751278319
41	H	1.9054523405	-2.9113178709	0.7473334496
42	H	1.2676142165	-3.5455496886	2.2689285496
43	H	2.9374483991	-2.9858603733	2.1723531526

(7*S**,9*R**)-**S2A**, M0003:M0009

ATOM		X	Y	Z
1	C	0.0774679415	0.1741998448	-1.4096165030
2	C	-1.3605135918	-0.2555756074	-1.0662875384
3	C	-1.2673863316	-0.6922542693	0.3843141494
4	C	0.1058580696	-1.3650365240	0.4184092585
5	O	0.9104719584	-0.4933031717	-0.4186682579
6	C	-1.6246323325	-1.4697624436	-1.9391747603
7	N	-0.5914463286	-1.5850410374	-2.8130226736
8	C	0.3923029777	-0.5231248343	-2.7652695753
9	C	0.7041642690	-1.4478038938	1.8074206052
10	C	0.6990132457	-0.0666290505	2.4515374271
11	C	-0.7272219505	0.4617964249	2.4819077531
12	O	-1.2503730905	0.5354277032	1.1506897814
13	O	-2.5636115165	-2.2284302793	-1.8322454341
14	C	0.3017271392	0.2986631144	-4.0230745888
15	O	-0.3531898626	0.0515930904	-4.9936661256
16	O	1.1853095996	1.3339469247	-3.9781294876
17	C	0.3847285879	1.6519744856	-1.2691164403
18	C	-0.4487142572	2.5270671155	-2.1399016430
19	O	-1.3892024159	2.1880657940	-2.8172396719
20	O	-0.0532244475	3.8157524975	-2.0613558769

21	H	-2.0938483969	0.5128054240	-1.2335974158
22	H	-2.0724376167	-1.3381464997	0.6910334970
23	H	0.0589393886	-2.3510432965	-0.0190139223
24	C	-0.8564632147	1.8618554687	3.0553613441
25	O	-1.4765221230	-0.4897019608	3.2356874610
26	O	2.0250741110	-1.9791058754	1.6555870618
27	C	2.6082549240	-2.5578157223	2.8337676902
28	H	1.4033232864	-0.8962785992	-2.6877094831
29	H	0.0895661329	-2.1147471161	2.3979658076
30	H	-0.6074300603	-2.2617255636	-3.5456908094
31	H	1.0733751664	-0.1105106309	3.4645017745
32	H	1.3194066563	0.5858980217	1.8570897046
33	H	1.1865147831	1.8433258026	-4.8023845921
34	H	1.4257657672	1.8457386064	-1.4720354490
35	H	0.1698545819	1.9084500151	-0.2420021108
36	H	-0.6143063984	4.3997388277	-2.5931837036
37	H	-1.8864205185	2.1899590765	2.9803250787
38	H	-0.5537632872	1.8589077749	4.0937220095
39	H	-0.2378894764	2.5513680781	2.4984480818
40	H	-2.4068313373	-0.2295094963	3.2901180362
41	H	1.9747189397	-3.3380949921	3.2438304622
42	H	2.7894939245	-1.8166182410	3.6031211914
43	H	3.5500971036	-2.9862749853	2.5275478876

(7*S**,9*R**)-**S2A**, M0003:M0010

I	Atom	X	Y	Z
1	C	0.1162122054	0.3868246450	-1.4393307726
2	C	-1.3528065573	0.1112635903	-1.0428582935
3	C	-1.2611960544	-0.4690048260	0.3695678079
4	C	0.0836296921	-1.2020050161	0.3157798700
5	O	0.9170501759	-0.2938538593	-0.4505090607
6	C	-1.8336253210	-0.9803422113	-1.9786299137
7	N	-0.8432799622	-1.2323366810	-2.8741962800
8	C	0.3100532745	-0.3644497382	-2.8045794350
9	C	0.7247265233	-1.4371887540	1.6757824519

10	C	0.7265669642	-0.1431793188	2.4718255401
11	C	-0.6917252896	0.3811091841	2.5895098950
12	O	-1.2108507708	0.6511608809	1.2731319522
13	O	-2.8890176424	-1.5694521610	-1.8994825536
14	C	0.3489263850	0.5425750880	-4.0052048659
15	O	-0.5358507963	0.7506110736	-4.7870508544
16	O	1.5748581278	1.1132924662	-4.1074042741
17	C	0.5623024636	1.8522129364	-1.4793837193
18	C	0.1993658207	2.5645403991	-0.2103195286
19	O	0.9031724591	2.7786610085	0.7405367197
20	O	-1.0825155280	3.0059407245	-0.2866431417
21	H	-1.9819304631	0.9836623158	-1.0729668379
22	H	-2.0872229542	-1.1191870443	0.6021804985
23	H	-0.0399972337	-2.1274562496	-0.2307545302
24	C	-0.8015538040	1.6869196721	3.3551164802
25	O	-1.4610414631	-0.6615849437	3.1842044856
26	O	2.0911518271	-1.8453624848	1.5727803890
27	C	2.3374633116	-3.1334591730	0.9911248618
28	H	1.2453888588	-0.8964766286	-2.7461168756
29	H	0.1395649910	-2.1849150356	2.1953809271
30	H	-0.9810916631	-1.8814429303	-3.6192261849
31	H	1.1308218699	-0.3292383018	3.4557717940
32	H	1.3282296652	0.5838737655	1.9512666816
33	H	1.6454688126	1.7117487051	-4.8661797518
34	H	0.0668191826	2.3664239229	-2.2877653570
35	H	1.6310490776	1.8745419313	-1.6109371071
36	H	-1.4204227334	3.2997285973	0.5709535403
37	H	-1.8316913515	2.0285871707	3.3450813464
38	H	-0.4891443433	1.5338276972	4.3793945781
39	H	-0.1742491243	2.4284195225	2.8850828878
40	H	-2.3741160886	-0.3774012746	3.3302781871
41	H	2.1252989113	-3.1407012598	-0.0711575510
42	H	1.7490750724	-3.9074975644	1.4752337332
43	H	3.3861334727	-3.3393898407	1.1407122607

(7*S**,9*R**)-**S2A,** M0003:M0011

I	Atom	X	Y	Z
1	C	-0.3134043437	0.1085020779	-1.2547513873
2	C	-1.7459222489	0.0886541025	-0.6916074143
3	C	-1.5548073977	-0.2401853794	0.7806927118
4	C	-0.4044302361	-1.2481549200	0.7278462531
5	O	0.4531835548	-0.6941354711	-0.3093908265
6	C	-2.4167945480	-1.0917592810	-1.3799068641
7	N	-1.5865032688	-1.5200852062	-2.3614002478
8	C	-0.3821619149	-0.7208218423	-2.5565347586
9	C	0.3520868153	-1.3686551363	2.0418748788
10	C	0.7834841321	0.0198302296	2.5092046187
11	C	-0.4547921852	0.8937157041	2.6548388559
12	O	-1.1229474239	0.9967828611	1.3888668272
13	O	-3.4821082154	-1.5795612134	-1.0674324168
14	C	-0.5392795166	0.0463965678	-3.8459424022
15	O	-0.4399505765	1.2273469043	-4.0471119856
16	O	-0.8157966454	-0.8315711082	-4.8403962394
17	C	0.3453106155	1.4707093430	-1.3098254598
18	C	1.7302449425	1.3752772284	-1.8679694001
19	O	2.1776086543	0.4800097829	-2.5396157935
20	O	2.4545660878	2.4728977946	-1.5556842101
21	H	-2.2971176029	1.0058282279	-0.8290679734
22	H	-2.4431030300	-0.6258727402	1.2505506714
23	H	-0.7654982470	-2.2186849100	0.4211332087
24	C	-0.1565147527	2.3206617633	3.0794597630
25	O	-1.3031698934	0.2279713544	3.5852821043
26	O	1.4037227151	-2.3350278776	1.9907977536
27	C	2.5807381220	-2.0694831962	1.1965306342
28	H	0.5097714294	-1.3168851038	-2.6248928597
29	H	-0.3382745732	-1.7591810043	2.7713493816
30	H	-1.8205435578	-2.2704366794	-2.9744048847
31	H	1.2828764474	-0.0549220907	3.4645254653
32	H	1.4376928987	0.4764714078	1.7830657347
33	H	-0.9128016459	-0.3965486872	-5.7002341350
34	H	0.3776609897	1.8575740311	-0.3066299576

35	H	-0.2060203147	2.1524995676	-1.9383745369
36	H	3.3370405150	2.4535153167	-1.9541240850
37	H	0.5224736930	2.7834582056	2.3776803494
38	H	0.2841884866	2.3207799107	4.0670135080
39	H	-1.0758542838	2.8937420980	3.0931825482
40	H	-2.1161441582	0.7298701466	3.7366025068
41	H	2.3398535634	-1.9527652203	0.1541836842
42	H	3.2213703579	-2.9255953874	1.3426423447
43	H	3.1000665601	-1.1821621711	1.5379740348

(7*S**,9*R**)-**S2A,** M0003:M0012

I	Atom	X	Y	Z
1	C	-0.0466812055	-0.0313368387	-1.2309408590
2	C	-1.4945925169	-0.0318379854	-0.7079253351
3	C	-1.3500625119	-0.3711827484	0.7629493573
4	C	-0.1968452166	-1.3756405620	0.7475545786
5	O	0.7017414411	-0.8037184064	-0.2490736455
6	C	-2.1833677401	-1.1673681580	-1.4409314717
7	N	-1.3411377055	-1.6052498935	-2.4121933389
8	C	-0.0863784177	-0.8981475396	-2.5288150662
9	C	0.4979633127	-1.5106676651	2.0938815122
10	C	0.9136703148	-0.1288162842	2.5939714640
11	C	-0.3255437784	0.7515907036	2.6853844268
12	O	-0.9341696309	0.8633004853	1.3922440068
13	O	-3.2735599877	-1.6254699298	-1.1765544677
14	C	0.0511301698	-0.1373272001	-3.8228034829
15	O	0.9787025591	0.5909796142	-4.0757714378
16	O	-0.9134627819	-0.4270154964	-4.7088866783
17	C	0.6286068847	1.3254530431	-1.2998651762
18	C	-0.0942396230	2.3130125431	-2.1550028545
19	O	-1.1520376423	2.1440642603	-2.7137440255
20	O	0.5710054150	3.4823238034	-2.2117845174
21	H	-2.0106922041	0.8963919382	-0.8737945975
22	H	-2.2517151760	-0.7584728354	1.2058303091
23	H	-0.5409623641	-2.3429656543	0.4110847320

24	C	-0.0364872197	2.1738981848	3.1317739310
25	O	-1.2187292873	0.0898084904	3.5758724904
26	O	1.5449815363	-2.4846079767	2.0846089058
27	C	2.7759172707	-2.1928419513	1.3898527142
28	H	0.7648548804	-1.5618807944	-2.4670305819
29	H	-0.2303447798	-1.9035857115	2.7847994421
30	H	-1.6339834454	-2.2773695630	-3.0874361852
31	H	1.3651434488	-0.2139080113	3.5721928280
32	H	1.6066654751	0.3292528175	1.9048601845
33	H	-0.8205214597	0.0794073888	-5.5293290229
34	H	1.6414099924	1.2364908115	-1.6615743281
35	H	0.6423846888	1.7084539682	-0.2899506303
36	H	0.1216125633	4.1312552935	-2.7731525178
37	H	0.6808034009	2.6350544147	2.4677616057
38	H	0.3539626995	2.1655319195	4.1403950708
39	H	-0.9505876339	2.7550280749	3.1027297167
40	H	-2.0362912151	0.5953630517	3.6849934396
41	H	3.3035701474	-1.3644153410	1.8472599937
42	H	2.6003091176	-1.9691280488	0.3513389748
43	H	3.3779582255	-3.0837062113	1.4852205357

(7*S**,9*R**)-**S2A,** M0003:M0013

I	Atom	X	Y	Z
1	C	-0.0332966554	0.3257634582	-1.2942811398
2	C	-1.4865734643	-0.0488678363	-0.9657640877
3	C	-1.4255627969	-0.5257206904	0.4763253124
4	C	-0.0851827168	-1.2677717064	0.4940346000
5	O	0.7637471960	-0.4596299222	-0.3612279072
6	C	-1.7848884126	-1.2372612274	-1.8646893968
7	N	-0.7833864718	-1.3257956581	-2.7739072273
8	C	0.2391696745	-0.2785219323	-2.6945399661
9	C	0.5479632008	-1.3826487238	1.8745944053
10	C	0.5357092701	-0.0316667443	2.5706666680
11	C	-0.8769632511	0.5175254631	2.6167108196
12	O	-1.3698497331	0.6730183111	1.2760361412

13	O	-2.7216107578	-1.9990166031	-1.7480421280
14	C	-0.0165431977	0.6357296013	-3.8602828773
15	O	0.5712920394	0.6650397790	-4.9022380566
16	O	-1.1355957462	1.3878582094	-3.6388382917
17	C	0.3097949181	1.7859243794	-1.0732807650
18	C	1.7334258100	2.0630197057	-1.4381060915
19	O	2.3401916594	1.5197477670	-2.3279188756
20	O	2.2679638077	3.0527001430	-0.6949400100
21	H	-2.1931745926	0.7515288117	-1.1072000512
22	H	-2.2588528514	-1.1487658913	0.7517272201
23	H	-0.2215367741	-2.2433307094	0.0483187234
24	C	-0.9951125943	1.8875074279	3.2598916938
25	O	-1.6579562733	-0.4643952916	3.2934827038
26	O	1.9245344180	-1.7665995914	1.8270326192
27	C	2.2532628122	-2.9677230565	1.1111852092
28	H	1.2366127933	-0.6632782977	-2.7521270007
29	H	-0.0344097293	-2.0936762767	2.4449419502
30	H	-0.7361458724	-2.0787947388	-3.4265294286
31	H	0.9209905610	-0.1419718498	3.5729342845
32	H	1.1683840996	0.6331136941	2.0038248546
33	H	-1.3910235246	1.9044341593	-4.4177742030
34	H	0.1347433950	2.0154524811	-0.0392500343
35	H	-0.3246009429	2.4069328553	-1.6888568260
36	H	3.1803923430	3.2531146763	-0.9519177356
37	H	-2.0177522557	2.2364192176	3.1825055575
38	H	-0.7111663488	1.8255457625	4.3015190064
39	H	-0.3540621814	2.5924012300	2.7499673087
40	H	-2.5848206145	-0.1935942727	3.3541515251
41	H	2.1387864461	-2.8298190719	0.0443805536
42	H	1.6449050025	-3.8074726853	1.4340024042
43	H	3.2881983123	-3.1764543558	1.3334785393

(7*S**,9*R**)-**S2A,** M0003:M0014

ATOM		X	Y	Z
1	C	0.1831644476	0.4149875513	-1.4172918905
2	C	-1.2891313413	0.1313561325	-1.0351322726

3	C	-1.2132612714	-0.4371186640	0.3838928035
4	C	0.1342311017	-1.1663159340	0.3514498391
5	O	0.9734112503	-0.2664301763	-0.4166218484
6	C	-1.7473670297	-0.9798256743	-1.9600422161
7	N	-0.7326487997	-1.2565321586	-2.8203969026
8	C	0.3975138310	-0.3586070777	-2.7690392422
9	C	0.7624539929	-1.3845725655	1.7205185185
10	C	0.7454285361	-0.0844819607	2.5065081999
11	C	-0.6780826901	0.4294324609	2.6049987017
12	O	-1.1826353290	0.6882358922	1.2805494419
13	O	-2.8084231924	-1.5616887409	-1.8996069774
14	C	0.5075635461	0.4958231528	-4.0116497742
15	O	1.3403448504	1.3479668722	-4.1820356537
16	O	-0.3997936801	0.1709004713	-4.9548558749
17	C	0.6398669236	1.8770915706	-1.4430166040
18	C	0.2419456077	2.5917337665	-0.1865008272
19	O	0.9208877730	2.8194773046	0.7792684713
20	O	-1.0420621920	3.0245754526	-0.2947233086
21	H	-1.9254000584	0.9977893656	-1.0813487619
22	H	-2.0394156958	-1.0897169321	0.6095347733
23	H	0.0178871799	-2.0984390225	-0.1848443219
24	C	-0.8084344098	1.7379573866	3.3625245204
25	O	-1.4468608113	-0.6168583567	3.1947829237
26	O	2.1332941252	-1.7822693667	1.6358928047
27	C	2.3976847603	-3.0670642466	1.0551509474
28	H	1.3383547622	-0.8791811856	-2.6614542213
29	H	0.1779522488	-2.1331922503	2.2395493696
30	H	-0.8638153257	-1.8933733177	-3.5765297535
31	H	1.1404240409	-0.2599271586	3.4961052013
32	H	1.3468938724	0.6436165637	1.9871551485
33	H	-0.2953076439	0.7051246650	-5.7566865478
34	H	0.1958073549	2.3995010616	-2.2719838358
35	H	1.7135471290	1.8886924840	-1.5326259098
36	H	-1.3962836205	3.3290817172	0.5524689098
37	H	-1.8419551737	2.0685708848	3.3402133389
38	H	-0.5048512130	1.5934874022	4.3907461786

39	H	-0.1850889471	2.4831750799	2.8936138812
40	H	-2.3625635401	-0.3375244175	3.3332112719
41	H	2.2014688441	-3.0729079429	-0.0101787495
42	H	1.8078087564	-3.8467377730	1.5283722528
43	H	3.4454470302	-3.2658123160	1.2200579960

(7*S**,9*R**)-**S2A**, M0003:M0015

I	Atom	X	Y	Z
1	C	0.0692631549	0.2892839905	-1.6095177054
2	C	-1.3884269387	-0.0555599617	-1.2220676659
3	C	-1.2765558286	-0.5908953275	0.2060740982
4	C	0.1100025132	-1.2403566640	0.1899113964
5	O	0.8911148744	-0.2830650699	-0.5726752969
6	C	-1.8015109507	-1.1953287846	-2.1318105350
7	N	-0.7853172928	-1.4271072161	-3.0020638006
8	C	0.3338601296	-0.5156042228	-2.9326381713
9	C	0.7286482053	-1.4122330042	1.5606369941
10	C	0.6898852837	-0.0808512234	2.3014921600
11	C	-0.7555010578	0.3814782223	2.3955443589
12	O	-1.2935527997	0.5619019057	1.0744421660
13	O	-2.8308712807	-1.8293023798	-2.0521895559
14	C	0.3835572923	0.3371405580	-4.1719092144
15	O	-0.4763186336	0.4714811589	-4.9967234662
16	O	1.5884989640	0.9543178729	-4.2552820467
17	C	0.4228750355	1.7760331194	-1.7171356481
18	C	0.0176790066	2.5142868239	-0.4767253179
19	O	0.7065731376	2.8000027627	0.4667179663
20	O	-1.2861808632	2.8817945054	-0.5677759226
21	H	-2.0630691067	0.7805721446	-1.2795415091
22	H	-2.0680144844	-1.2768893682	0.4555519649
23	H	0.0815297784	-2.1880840357	-0.3282750801
24	C	-0.9268849531	1.7145014877	3.1009773110
25	O	-1.4683765410	-0.6670590992	3.0502098641
26	O	2.0579806124	-1.8978735142	1.3410982967
27	C	2.7030414420	-2.4945988606	2.4768267582

28	H	1.2846441122	-1.0102714943	-2.8173968929
29	H	0.1445455393	-2.1406722520	2.1076557695
30	H	-0.8738378025	-2.1123448515	-3.7216752514
31	H	1.0860369588	-0.1836344752	3.3020602665
32	H	1.2552704322	0.6502432737	1.7465840401
33	H	1.6649391928	1.5167037602	-5.0405706883
34	H	-0.1044747159	2.2211919766	-2.5464606732
35	H	1.4880631646	1.8587665277	-1.8519866661
36	H	-1.6395408572	3.1939400301	0.2770070637
37	H	-1.9726595262	2.0041886569	3.0799177077
38	H	-0.6064881914	1.6229956977	4.1301545280
39	H	-0.3379342445	2.4637220813	2.5954800962
40	H	-2.3987499105	-0.4296008681	3.1677914342
41	H	2.1065263863	-3.3027330406	2.8892667522
42	H	2.8978682656	-1.7709048110	3.2589095547
43	H	3.6418624973	-2.8895760316	2.1201105603

(7*S**,9*R**)-**S2A**, M0003:M0016

I	Atom	X	Y	Z
1	C	-0.0981338205	-0.2994484501	-1.2244218658
2	C	1.3344342997	0.1159036826	-0.8509794864
3	C	1.2197797640	0.5786537601	0.5906175928
4	C	-0.1448086490	1.2699898334	0.5867318598
5	O	-0.9430546213	0.4196223101	-0.2758597651
6	C	1.6403989734	1.3035188664	-1.7474832383
7	N	0.6393108677	1.4067512129	-2.6584810303
8	C	-0.3776307545	0.3779550970	-2.5991319254
9	C	-0.7977408655	1.3592724596	1.9590676624
10	C	-0.7707354915	0.0014782640	2.6409031134
11	C	0.6540347335	-0.5162019882	2.7036115303
12	O	1.1774216601	-0.6350659256	1.3716291713
13	O	2.5865334518	2.0527588116	-1.6328688975
14	C	-0.3250722473	-0.4714357615	-3.8376799471
15	O	0.3241571064	-0.2657818917	-4.8223708512
16	O	-1.2443012062	-1.4719547217	-3.7595762708

17	C	-0.4680983603	-1.7588145374	-1.0576152733
18	C	0.3126858823	-2.7498800897	-1.8645789765
19	O	0.1433655617	-3.9373632592	-1.8675285654
20	O	1.2946962432	-2.1707394564	-2.6147682248
21	H	2.0608624825	-0.6647331822	-0.9728856458
22	H	2.0258574067	1.2222931223	0.8976642200
23	H	-0.0428969617	2.2484266703	0.1384000192
24	C	0.7932689189	-1.8948247465	3.3238451484
25	O	1.3956843182	0.4719571849	3.4149755003
26	O	-2.1777422331	1.7274871122	1.8917723183
27	C	-2.4831388415	3.0279759693	1.3646608057
28	H	-1.3780307209	0.7793477642	-2.5281788923
29	H	-0.2343735333	2.0719591606	2.5465136409
30	H	0.6839181747	2.0672792308	-3.4038111467
31	H	-1.1707619331	0.0942473536	3.6389265358
32	H	-1.3812633291	-0.6709467964	2.0595991644
33	H	-1.2914833759	-1.9980918416	-4.5712591886
34	H	-1.5100264678	-1.8905048907	-1.3068722445
35	H	-0.3214415710	-1.9964656369	-0.0159437274
36	H	1.7983358409	-2.8114997389	-3.1384332143
37	H	1.8277384455	-2.2117640493	3.2717598913
38	H	0.4751531703	-1.8638220010	4.3567773421
39	H	0.1911867527	-2.6080577801	2.7796743623
40	H	2.3248985078	0.2174161820	3.5027681855
41	H	-2.2958324417	3.0803505259	0.2997996828
42	H	-1.9127942600	3.8050142544	1.8645679070
43	H	-3.5343608770	3.1877379171	1.5464627235

(7*S**,9*R**)-**S2A,** M0003:M0017

I	Atom	X	Y	Z
1	C	-0.2049537705	0.3292574888	-1.4480982247
2	C	-1.6209374512	-0.1541355807	-1.0941805479
3	C	-1.4962883828	-0.5919134668	0.3597862342
4	C	-0.1110456845	-1.2450974979	0.3665237772
5	O	0.6561621132	-0.3969172074	-0.5241696224

6	C	-1.8302644366	-1.3795741054	-1.9738402298
7	N	-0.8127768211	-1.4183318211	-2.8701956090
8	C	0.0917045364	-0.2743237143	-2.8345433585
9	C	0.5469922651	-1.2683187377	1.7309130132
10	C	0.5471300335	0.1377198014	2.3192293659
11	C	-0.8910961128	0.6228828793	2.4189808941
12	O	-1.5007283053	0.6368807160	1.1192128344
13	O	-2.7240341972	-2.1907700636	-1.8581407076
14	C	-0.1898389309	0.5820962869	-4.0460821585
15	O	-0.3938480497	1.7640452104	-4.1211515964
16	O	-0.1483249460	-0.2053479204	-5.1513873765
17	C	0.0747108904	1.8089654798	-1.2138053388
18	C	1.5472840372	1.9803417537	-1.0174448611
19	O	2.1011937777	2.4611384063	-0.0651044446
20	O	2.2355426405	1.5184426654	-2.0974462220
21	H	-2.3981010894	0.5824821580	-1.2260238812
22	H	-2.2835418976	-1.2553254141	0.6751380878
23	H	-0.1602219210	-2.2450803243	-0.0362528914
24	C	-1.0270822338	2.0394007249	2.9474127136
25	O	-1.5677411120	-0.3250500027	3.2427773142
26	O	1.8645515695	-1.7907318604	1.5254797830
27	C	2.5429301508	-2.2713427633	2.6967988209
28	H	1.1284418886	-0.5595476459	-2.8535312205
29	H	-0.0262975623	-1.9208207883	2.3756289037
30	H	-0.7564264931	-2.1051312716	-3.5900925389
31	H	0.9739487294	0.1400226960	3.3121318457
32	H	1.1125978353	0.7920202798	1.6748007890
33	H	-0.3035379949	0.2966060326	-5.9648459116
34	H	-0.4176635491	2.0901142115	-0.3018424018
35	H	-0.2574812549	2.4049950684	-2.0462325031
36	H	3.1936009605	1.5613702602	-1.9624153351
37	H	-2.0675526124	2.3400502281	2.9168132494
38	H	-0.6685086612	2.0811170041	3.9668234885
39	H	-0.4520781356	2.7182738847	2.3342812673
40	H	-2.4967606274	-0.0814882396	3.3581007675
41	H	1.9529392021	-3.0210690807	3.2147662310

42	H	2.7741956917	-1.4712335335	3.3891782852
43	H	3.4632059117	-2.7166721965	2.3520493160

(7*S**,9*R**)-**S2A**, M0003:M0018

I	Atom	X	Y	Z
1	C	-0.0936692912	0.3644017924	-1.3093109735
2	C	-1.5270737092	-0.0550292586	-0.9596362435
3	C	-1.4212905726	-0.5272288949	0.4833249904
4	C	-0.0610903451	-1.2361213185	0.4738771112
5	O	0.7423442827	-0.4301218532	-0.4272430134
6	C	-1.8012168618	-1.2365513209	-1.8742701249
7	N	-0.8166021934	-1.2676765815	-2.8100429178
8	C	0.1530640581	-0.1770076250	-2.7334599695
9	C	0.6280122504	-1.2941145641	1.8301457633
10	C	0.6239381381	0.0860057843	2.4666196303
11	C	-0.8046159857	0.5844548289	2.5822862025
12	O	-1.3884613847	0.6802783240	1.2722866308
13	O	-2.7054590119	-2.0351691011	-1.7573686340
14	C	-0.1736553473	0.8059622255	-3.8234672571
15	O	0.4625626688	1.0395738954	-4.8113298211
16	O	-1.3815281320	1.3910416619	-3.5799543510
17	C	0.2317705229	1.8271967920	-1.0409126374
18	C	1.7129221383	1.9716595147	-0.9094871748
19	O	2.3271939481	2.3738707006	0.0392966067
20	O	2.3166945231	1.5776584014	-2.0642000740
21	H	-2.2561469503	0.7262499070	-1.0908584889
22	H	-2.2290719681	-1.1726159313	0.7835027244
23	H	-0.1865063800	-2.2232023787	0.0514149604
24	C	-0.9320697048	1.9688819949	3.1912625210
25	O	-1.5109631937	-0.3994009943	3.3340606928
26	O	2.0030385001	-1.6727801478	1.7316979534
27	C	2.2857080993	-2.9685217640	1.1827285952
28	H	1.1661344834	-0.5079162785	-2.8404080679
29	H	0.0806552433	-1.9877384284	2.4542313727
30	H	-0.7686230610	-1.9906108213	-3.4953139816

31	H	1.0696652997	0.0270578586	3.4481302942
32	H	1.2001347856	0.7518753772	1.8416529525
33	H	-1.6626717743	1.9743401015	-4.3004755070
34	H	-0.2227532155	2.0955135643	-0.1050877196
35	H	-0.1445678628	2.4523189789	-1.8339918247
36	H	3.2830284728	1.6100080484	-2.0135951075
37	H	-1.9682751671	2.2844796455	3.1611293447
38	H	-0.5903767594	1.9457880069	4.2171039256
39	H	-0.3399070460	2.6767365094	2.6291843279
40	H	-2.4348137971	-0.1417145823	3.4603668134
41	H	2.0847777925	-3.0009606092	0.1194063722
42	H	1.7124706190	-3.7466775025	1.6781447652
43	H	3.3372938885	-3.1441939576	1.3485593383

(7*S**,9*R**)-**S2A**, M0003:M0019

ATOM		X	Y	Z
1	C	0.1331105198	0.2622081914	-1.2529069620
2	C	-1.3155810366	-0.0975203245	-0.8848606816
3	C	-1.2174646714	-0.5699358215	0.5558804855
4	C	0.1310751190	-1.2926662935	0.5558924608
5	O	0.9568353243	-0.4498909372	-0.2907656245
6	C	-1.6726927507	-1.2605125922	-1.7887943779
7	N	-0.6875289599	-1.3840310676	-2.7167926147
8	C	0.3832421499	-0.4155974808	-2.6400455746
9	C	0.7716904073	-1.4133639731	1.9317444360
10	C	0.7645240186	-0.0640337379	2.6313433641
11	C	-0.6488979253	0.4845091687	2.6842319737
12	O	-1.1523476936	0.6344256553	1.3487618967
13	O	-2.6407422707	-1.9806932440	-1.6748562988
14	C	0.3126558978	0.5757800021	-3.7653996653
15	O	-0.6435501688	0.8393019027	-4.4398827849
16	O	1.5087409868	1.1968052848	-3.8730898750
17	C	0.5301721706	1.7349517021	-1.1092892627
18	C	-0.1890682715	2.7193038138	-1.9884009464
19	O	0.3195103469	3.4813264066	-2.7703226320
20	O	-1.5295028173	2.7030342709	-1.7892544409

21	H	-1.9974779323	0.7252447723	-0.9998200590
22	H	-2.0387090814	-1.1981703532	0.8553589072
23	H	0.0123705340	-2.2624446421	0.0923787288
24	C	-0.7605385487	1.8556441317	3.3260254907
25	O	-1.4248732927	-0.4925685822	3.3736097720
26	O	2.1436883519	-1.8109549924	1.8726946226
27	C	2.4219037012	-3.1053012304	1.3173161239
28	H	1.3640693721	-0.8582831474	-2.6189544011
29	H	0.1893705179	-2.1228947325	2.5053124508
30	H	-0.7518424800	-2.0617687210	-3.4457068041
31	H	1.1526484757	-0.1772796411	3.6323716993
32	H	1.3953258808	0.6047829063	2.0669477842
33	H	1.4707243638	2.0187553997	-4.3850744121
34	H	1.5860439771	1.8330025542	-1.2959867749
35	H	0.3002953843	1.9789459482	-0.0825623949
36	H	-1.9943485447	3.3155804681	-2.3797632252
37	H	-1.7836573475	2.2054459171	3.2565989353
38	H	-0.4684242767	1.7948996219	4.3655761513
39	H	-0.1242832576	2.5609231471	2.8105481946
40	H	-2.3442885398	-0.2047807182	3.4626980126
41	H	2.2457708263	-3.1255413560	0.2492780186
42	H	1.8253705994	-3.8787163147	1.7920216548
43	H	3.4666809416	-3.2979213616	1.5059386493

(7*R**,9*R**)-**S2B**, M0004:M0001

I	Atom	X	Y	Z
1	C	-0.4856692755	0.1627061765	-1.3821669079
2	C	-0.6764245119	-1.2887640374	-0.9028329824
3	C	-0.1122434127	-1.2847901841	0.5104702086
4	C	1.0938085317	-0.3593763007	0.3392481261
5	O	0.6160291625	0.6648684212	-0.5645539961
6	C	0.2324545498	-2.1104211499	-1.8066896662
7	N	0.6657656277	-1.2971844076	-2.8029184347
8	C	0.0822823988	0.0372284697	-2.8133636994
9	C	1.5440068096	0.3188865332	1.6212012580
10	C	0.3591192736	0.8727623056	2.4099917522
11	C	-0.7002990398	-0.2124117697	2.6318397962
12	O	-1.1161029265	-0.6526624305	1.3166730140
13	O	0.5467144710	-3.2687559694	-1.6347222702
14	C	-0.9150503913	0.1222649987	-3.9440746548
15	O	-2.0329912414	0.5643281399	-3.9326353238
16	O	-0.3466310192	-0.3591647364	-5.0762562177
17	C	-1.6662542353	1.0837813950	-1.1601544529
18	C	-1.3939473983	2.4584786905	-1.6834570924
19	O	-0.5204919365	2.7866847480	-2.4460528349
20	O	-2.3020939223	3.3399879409	-1.2076383778
21	H	-1.6965525102	-1.6394604865	-0.9223037173
22	H	0.1417259250	-2.2645644745	0.8767261682
23	H	1.9120947225	-0.9009602182	-0.1124498317
24	C	-1.9622875822	0.2931787603	3.2976304321
25	O	-0.1976101437	-1.2836942275	3.3927597496
26	O	2.1738347786	-0.6873630909	2.4549381187
27	C	3.5822687770	-0.9291080359	2.2651925608
28	H	0.8131746751	0.8147191431	-2.9509050263
29	H	2.2499091429	1.1050812635	1.3874985619
30	H	1.2219341390	-1.6296643373	-3.5603338148
31	H	0.7093802452	1.2376350477	3.3655606755
32	H	-0.0791567588	1.6825102210	1.8468850683
33	H	-0.9412058445	-0.2976189181	-5.8383762179

34	H	-1.8509092838	1.1211454742	-0.1006376828
35	H	-2.5523720936	0.7225711565	-1.6578929008
36	H	-2.1736811210	4.2273058107	-1.5735682286
37	H	-2.6493638340	-0.5362292257	3.3857759528
38	H	-1.7286280853	0.6647493567	4.2861374255
39	H	-2.4170507587	1.0747155649	2.7053132839
40	H	0.7657378251	-1.3609938330	3.2795432246
41	H	4.1416828520	-0.0068639133	2.3642285689
42	H	3.7838831497	-1.3679217354	1.2969263571
43	H	3.8812102700	-1.6176161357	3.0394440281

(7*R**,9*R**)-**S2B,** M0004:M0002

ATOM		X	Y	Z
1	C	-0.2826456991	0.3581832103	-1.3693833025
2	C	-0.4530829054	-1.1010263290	-0.9090364448
3	C	0.0860037962	-1.1006101657	0.5102634927
4	C	1.2650791989	-0.1363774351	0.3810968001
5	O	0.7658381089	0.8947967168	-0.5067719673
6	C	0.4538352106	-1.9051580684	-1.8217953257
7	N	0.8767793632	-1.0825118416	-2.8184312836
8	C	0.3521269404	0.2629440422	-2.7907697964
9	C	1.6758011337	0.5226971877	1.6857725551
10	C	0.4635195183	1.0314062346	2.4629958826
11	C	-0.5754912711	-0.0820730027	2.6373487673
12	O	-0.9513059005	-0.5052070803	1.3062476411
13	O	0.7788951702	-3.0620617529	-1.6669498220
14	C	-0.5805225546	0.5686870408	-3.9345292222
15	O	-1.1583629191	1.6201057677	-4.0579348469
16	O	-0.6327277978	-0.4083422473	-4.8516579426
17	C	-1.4883167109	1.2600268977	-1.1866440894
18	C	-2.7148549106	0.7712693482	-1.8840313456
19	O	-2.8309395396	-0.2602117986	-2.5024038617
20	O	-3.7394485507	1.6281218856	-1.7148046137
21	H	-1.4669983570	-1.4534095486	-0.9669318043
22	H	0.3614180204	-2.0766111694	0.8711343800
23	H	2.1057248472	-0.6415257127	-0.0728919036

24	C	-1.8623890145	0.3829300642	3.2854428881
25	O	-0.0662167780	-1.1554235534	3.3915605559
26	O	2.3119849050	-0.4930346434	2.5031188482
27	C	3.7349660254	-0.6719502793	2.3593810458
28	H	1.1332247971	1.0095184431	-2.8181884749
29	H	2.3691329291	1.3295208934	1.4866216025
30	H	1.3807222129	-1.4387429971	-3.6010628580
31	H	0.7849354120	1.3824860186	3.4338592509
32	H	0.0182464149	1.8436842175	1.9088338191
33	H	-1.2577339121	-0.2068728673	-5.5636737504
34	H	-1.2777803199	2.2627544299	-1.5265709743
35	H	-1.6966146592	1.2752507453	-0.1272675392
36	H	-4.5434437973	1.3282523513	-2.1640411306
37	H	-2.5323084354	-0.4630191688	3.3448492940
38	H	-1.6577050262	0.7418433814	4.2849838316
39	H	-2.3231276048	1.1648056450	2.6978678650
40	H	0.8993670489	-1.2163532060	3.2899019652
41	H	4.2560054747	0.2567501089	2.5575623958
42	H	3.9929628074	-1.0235585307	1.3688772988
43	H	4.0254473287	-1.4119532321	3.0880521202

(7*R**,9*R**)-**S2B,** M0004:M0003

ATOM		X	Y	Z
1	C	-0.2303341970	0.2594062151	-1.4759816926
2	C	-0.3174152244	-1.2009240866	-0.9967867055
3	C	0.1847183336	-1.1451132451	0.4349407892
4	C	1.2945899421	-0.0997541479	0.3287899620
5	O	0.7459681001	0.8809813678	-0.5875364170
6	C	0.6580377897	-1.9617776670	-1.8750661088
7	N	1.0612241290	-1.1255358334	-2.8686575594
8	C	0.4583448848	0.1865062255	-2.8739656828
9	C	1.6175402862	0.6112903792	1.6287901925
10	C	0.3501197962	1.0409101192	2.3666475250
11	C	-0.6049684233	-0.1485341621	2.5276620793
12	O	-0.9146689254	-0.6114692639	1.1941960142
13	O	1.0390258634	-3.0983141095	-1.7006797763

14	C	-0.4472130727	0.4244915027	-4.0549340886
15	O	-1.0883342193	1.4347774119	-4.2077081898
16	O	-0.3962659863	-0.5558092508	-4.9687124968
17	C	-1.5010554695	1.0777309932	-1.3525089144
18	C	-2.6633551850	0.4931161036	-2.0850930315
19	O	-2.6790241593	-0.5460636733	-2.7011288472
20	O	-3.7567326775	1.2679886320	-1.9535307422
21	H	-1.3050281887	-1.6191332439	-1.0688778670
22	H	0.5154590494	-2.0957941132	0.8162274120
23	H	2.1935155152	-0.5406854784	-0.0742666017
24	C	-1.9363901383	0.2176217235	3.1468251917
25	O	-0.0289795038	-1.1716680919	3.3057158033
26	O	2.3424389854	-0.3769516242	2.4111467383
27	C	3.3867874985	0.1235010037	3.2726713991
28	H	1.1937524320	0.9786336654	-2.8817897495
29	H	2.2547935654	1.4606363687	1.4206394414
30	H	1.6083769330	-1.4606938457	-3.6313626580
31	H	0.6002681533	1.4352418793	3.3431541810
32	H	-0.1373161281	1.8083702456	1.7846911019
33	H	-1.0033916278	-0.3987648403	-5.7068467937
34	H	-1.3491189384	2.0870461491	-1.7037508683
35	H	-1.7484159405	1.0940460124	-0.3016198759
36	H	-4.5204788195	0.9060525628	-2.4266562338
37	H	-2.5398758656	-0.6774148857	3.1971921889
38	H	-1.7819727084	0.5966155768	4.1480161355
39	H	-2.4428143700	0.9585117613	2.5440178755
40	H	0.9313947658	-1.2076682089	3.1580110713
41	H	2.9881737128	0.7870023005	4.0299288622
42	H	4.1356366106	0.6486002791	2.6929971522
43	H	3.8329834224	-0.7370087066	3.7451997841

(7*R**,9*R**)-**S2B**, M0004:M0004

I	Atom	X	Y	Z
1	C	-0.4160528617	0.0404329451	-1.4975018555
2	C	-0.5239233785	-1.4124709544	-0.9981884424

3	C	-0.0086608821	-1.3477483308	0.4319945828
4	C	1.1332525680	-0.3399867734	0.2898779175
5	O	0.6152220292	0.6321659025	-0.6494806182
6	C	0.4662904537	-2.1867339845	-1.8574933780
7	N	0.8770619253	-1.3625818922	-2.8539182334
8	C	0.2144730279	-0.0656613429	-2.9034247482
9	C	1.4858410371	0.3927003371	1.5702558579
10	C	0.2382402305	0.8734311200	2.3104443768
11	C	-0.7443206851	-0.2867962345	2.5133808709
12	O	-1.0825346145	-0.7736458134	1.1937364815
13	O	0.8467791878	-3.3200096633	-1.6562417715
14	C	-0.7375875544	-0.0625878373	-4.0746035467
15	O	-1.8982477741	0.2484168483	-4.1086463299
16	O	-0.0681811748	-0.4577781613	-5.1850781972
17	C	-1.6655106374	0.8797714417	-1.3352051241
18	C	-1.4649457227	2.2636084197	-1.8663914398
19	O	-0.6071762322	2.6312323383	-2.6291046000
20	O	-2.4217084593	3.0995357049	-1.4035109697
21	H	-1.5169273939	-1.8319743544	-1.0421392867
22	H	0.2993085789	-2.3024113634	0.8220410247
23	H	2.0164851603	-0.8202832049	-0.1016668168
24	C	-2.0598357261	0.1265759600	3.1365229885
25	O	-0.1846549771	-1.3062857829	3.3068923091
26	O	2.1927956024	-0.5951846173	2.3703826191
27	C	3.2753524761	-0.1097738717	3.1925189231
28	H	0.9017229871	0.7524822044	-3.0258358146
29	H	2.1427113517	1.2191630797	1.3340913421
30	H	1.4920650545	-1.6632660010	-3.5783539400
31	H	0.5096392440	1.2875170722	3.2729626165
32	H	-0.2369171616	1.6370056238	1.7136366472
33	H	-0.6310567561	-0.4526406611	-5.9732525212
34	H	-1.8978005158	0.9155273033	-0.2854109874
35	H	-2.5011627441	0.4525852315	-1.8673675260
36	H	-2.3397639522	3.9887090869	-1.7782900122
37	H	-2.6841831493	-0.7519828252	3.2136945511
38	H	-1.8856392264	0.5245447699	4.1270032349

39	H	-2.5539746811	0.8654198974	2.5214516257
40	H	0.7734660933	-1.3676139077	3.1511185697
41	H	2.9206555515	0.5893428253	3.9394900555
42	H	4.0286670749	0.3702064463	2.5805625556
43	H	3.7007366261	-0.9729569806	3.6790470094

(*7R**,*9R**)-**S2B**, M0004:M0005

I	Atom	X	Y	Z
1	C	-0.3546473918	0.0775506142	-1.4993634621
2	C	-0.5441897418	-1.3834217821	-1.0617040678
3	C	-0.0822384108	-1.3922045215	0.3884107742
4	C	1.1050882493	-0.4253384161	0.3392876391
5	O	0.6573043787	0.5953432628	-0.5918751872
6	C	0.4419866091	-2.1750231893	-1.9085123645
7	N	0.9302244206	-1.3319626707	-2.8537482769
8	C	0.3298133197	0.0040582578	-2.8820004535
9	C	1.4278852396	0.3226310457	1.6208604182
10	C	0.1684165195	0.7588820071	2.3605244958
11	C	-0.8071493482	-0.4159620922	2.4706252431
12	O	-1.1612539270	-0.7798331068	1.1210341093
13	O	0.7737723525	-3.3258904950	-1.7320260200
14	C	-0.6514100391	0.0125618491	-4.0203169792
15	O	-1.7943632884	-0.3574893666	-3.9686021703
16	O	-0.0607575513	0.4093495929	-5.1648366505
17	C	-1.5955606107	0.9323675346	-1.3664067766
18	C	-1.4085976734	2.2960205107	-1.9419263790
19	O	-0.5333360825	2.6432497807	-2.6928901684
20	O	-2.3994758361	3.1268154526	-1.5485855484
21	H	-1.5517612204	-1.7492652824	-1.1487485779
22	H	0.1578651565	-2.3618417144	0.7722919878
23	H	1.9910568601	-0.9263535711	-0.0113086638
24	C	-2.1081951335	-0.0803047934	3.1658314762
25	O	-0.1926301835	-1.5485978384	3.0616179161
26	O	2.1849400138	-0.5925133959	2.4614355419
27	C	3.2857368695	-0.0353842637	3.2148229598

28	H	1.0410873875	0.7905419345	-2.9970189103
29	H	2.0345746689	1.1713294815	1.3441560304
30	H	1.6236251126	-1.6173433815	-3.5084124276
31	H	0.4340438883	1.1152511925	3.3470626098
32	H	-0.3149665515	1.5552422891	1.8208659476
33	H	-0.6495237834	0.3898473803	-5.9315814920
34	H	-1.8094918354	0.9908661403	-0.3154769179
35	H	-2.4362892902	0.4737218423	-1.8606188109
36	H	-2.3303554200	4.0136745489	-1.9264709175
37	H	-2.7266477866	-0.9635817356	3.1539073277
38	H	-1.9134008776	0.2096604288	4.1881811902
39	H	-2.6083837689	0.7223210586	2.6453483618
40	H	0.7483317347	-1.4134018967	3.2353836537
41	H	2.9508331289	0.7153406040	3.9189522043
42	H	4.0178623161	0.4072705465	2.5537674751
43	H	3.7301775260	-0.8581838421	3.7480638606

(7*R**,9*R**)-**S2B**, M0004:M0006

I	Atom	X	Y	Z
1	C	-0.2895613015	0.2905034041	-1.3943265655
2	C	-0.4678863453	-1.1619698640	-0.9177954190
3	C	0.0822066125	-1.1505046128	0.4980383199
4	C	1.2781991200	-0.2103022110	0.3414806515
5	O	0.7951230196	0.8120060732	-0.5654292373
6	C	0.4477768555	-1.9742967228	-1.8172063931
7	N	0.8773150119	-1.1585417586	-2.8176577508
8	C	0.3103479224	0.1736872133	-2.8239972149
9	C	1.7123165152	0.4635933289	1.6310546364
10	C	0.5179964786	1.0017425247	2.4162637637
11	C	-0.5353707102	-0.0932787002	2.6196442901
12	O	-0.9350701819	-0.5279229442	1.2987509838
13	O	0.7791754539	-3.1262169780	-1.6439156026
14	C	-0.6276418564	0.3265381103	-3.9913648083
15	O	-0.8023615064	-0.4607228815	-4.8748372659
16	O	-1.1803018875	1.5708166549	-3.9929890709

17	C	-1.4656316894	1.2211633623	-1.1736703818
18	C	-2.7157215467	0.7846454627	-1.8569072877
19	O	-2.8953086045	-0.2557859136	-2.4424259424
20	O	-3.6930759559	1.7043805332	-1.7147360692
21	H	-1.4833739786	-1.5117936949	-0.9722469105
22	H	0.3450859935	-2.1262961734	0.8685456011
23	H	2.1059023825	-0.7407044449	-0.1064138621
24	C	-1.8066058136	0.3998818621	3.2777267753
25	O	-0.0332465505	-1.1638528303	3.3818257400
26	O	2.3415824783	-0.5506833077	2.4556195180
27	C	3.7575805467	-0.7613796279	2.2859749553
28	H	1.0619875277	0.9450007999	-2.9085160982
29	H	2.4154715412	1.2567121465	1.4122343209
30	H	1.3991311748	-1.5173012462	-3.5881573758
31	H	0.8576682457	1.3605009863	3.3780643525
32	H	0.0781758157	1.8140261509	1.8574834100
33	H	-1.7529850843	1.7109788691	-4.7618945443
34	H	-1.2267629800	2.2240949892	-1.4908496882
35	H	-1.6569549212	1.2167758897	-0.1109633715
36	H	-4.5264634741	1.4111182230	-2.1122166126
37	H	-2.4892122227	-0.4344099494	3.3547442340
38	H	-1.5841007251	0.7666918933	4.2705757538
39	H	-2.2618252856	1.1826254080	2.6869511096
40	H	0.9301457089	-1.2405305694	3.2697865204
41	H	4.3034839536	0.1529843390	2.4836361837
42	H	3.9898954821	-1.1088252516	1.2877112747
43	H	4.0428947811	-1.5151485423	3.0024050778

(7*R**,9*R**)-**S2B,** M0004:M0007

I	Atom	X	Y	Z
1	C	-0.1832479216	0.2184032638	-1.5049124553
2	C	-0.3460037752	-1.2383105155	-1.0363799816
3	C	0.1256387562	-1.2115622252	0.4071761031
4	C	1.2965432832	-0.2317407323	0.3215712573
5	O	0.8302624187	0.7745661917	-0.6128036727

6	C	0.6376446150	-2.0272153778	-1.8834711575
7	N	1.1051355642	-1.1982639926	-2.8555974266
8	C	0.5064850237	0.1191465028	-2.8953452387
9	C	1.6326517759	0.4624668429	1.6276421400
10	C	0.3789094651	0.9604902735	2.3455979204
11	C	-0.6424106607	-0.1749817256	2.4895309423
12	O	-0.9550770882	-0.6208091007	1.1505542212
13	O	0.9810805755	-3.1729248488	-1.6955853915
14	C	-0.3606779529	0.2516184001	-4.1186063591
15	O	-0.4568513296	-0.5347725542	-5.0147128018
16	O	-0.9485714798	1.4796102350	-4.1500643673
17	C	-1.3999022001	1.1108422564	-1.3584151159
18	C	-2.5895214697	0.6365840624	-2.1197894212
19	O	-2.6990149713	-0.4089558534	-2.7133122713
20	O	-3.6021088130	1.5257116492	-2.0426111902
21	H	-1.3469918474	-1.6164089054	-1.1435983995
22	H	0.3971356778	-2.1799898425	0.7903364401
23	H	2.1768194825	-0.7266462086	-0.0580912545
24	C	-1.9624084556	0.2638961333	3.0857146935
25	O	-0.1375096801	-1.2274721551	3.2770154425
26	O	2.2888682904	-0.5632611246	2.4223172576
27	C	3.3424592666	-0.1190096594	3.3033436457
28	H	1.2419622732	0.9095509909	-2.9321993298
29	H	2.3187915748	1.2757673052	1.4315127698
30	H	1.6834297382	-1.5413112604	-3.5921112711
31	H	0.6346905344	1.3396619526	3.3266449141
32	H	-0.0566093941	1.7545939123	1.7581030928
33	H	-1.4741851153	1.6082713674	-4.9537624734
34	H	-1.1737378789	2.1217361143	-1.6586843507
35	H	-1.6573097973	1.0969703085	-0.3096861925
36	H	-4.3983848291	1.2070670698	-2.4930428479
37	H	-2.6158248151	-0.5960123158	3.1225397995
38	H	-1.8048192203	0.6312362215	4.0906602140
39	H	-2.4159512494	1.0335459592	2.4768201764
40	H	0.8208211357	-1.3204577953	3.1409653544
41	H	2.9665508342	0.5678446709	4.0513501212

42	H	4.1303101704	0.3620723892	2.7374140902
43	H	3.7309294893	-1.0015478794	3.7859723735

(7*R**,9*R**)-**S2B,** M0004:M0008

I	Atom	X	Y	Z
1	C	-0.3137061187	0.2316885413	-1.7016288605
2	C	-0.5386573290	-1.2051615021	-1.1756342877
3	C	0.0267945151	-1.1843000478	0.2466761733
4	C	1.1667547353	-0.1712852823	0.1064595556
5	O	0.6069453811	0.8405342668	-0.7682092894
6	C	0.3410192928	-2.0951181908	-2.0328117090
7	N	0.9105858213	-1.3204076241	-2.9955966999
8	C	0.4515268166	0.0471263582	-3.0588890057
9	C	1.5723758468	0.5221023421	1.3924518743
10	C	0.3533816562	0.9572054281	2.2022217417
11	C	-0.5958959395	-0.2263071604	2.4134065047
12	O	-1.0075570124	-0.6593172523	1.0904238048
13	O	0.5517271557	-3.2732298614	-1.8537120916
14	C	-0.4138888209	0.2496661990	-4.2741779683
15	O	-0.9132734310	-0.5974762704	-4.9593918960
16	O	-0.5569350732	1.5767247087	-4.5173632323
17	C	-1.5451905551	1.1362909606	-1.8129283035
18	C	-2.3099443682	1.1631704622	-0.5225716740
19	O	-2.2434568799	1.9909566135	0.3475689343
20	O	-3.1581187743	0.1072849789	-0.4767317191
21	H	-1.5695879298	-1.5134682754	-1.1816337605
22	H	0.3531813788	-2.1550171172	0.5781841543
23	H	2.0306803873	-0.6340811937	-0.3459833214
24	C	-1.8741035336	0.1510702693	3.1307507295
25	O	0.0162488636	-1.2811519934	3.1174014937
26	O	2.3412035553	-0.4776640231	2.1171888696
27	C	3.4117661555	0.0172091105	2.9490338441
28	H	1.2514337784	0.7684760900	-3.0815353851
29	H	2.2022838337	1.3676933361	1.1495289094
30	H	1.4870990163	-1.7263296146	-3.7014337532

31	H	0.6611990801	1.3491317335	3.1631899026
32	H	-0.1763850647	1.7169056417	1.6496444743
33	H	-1.1150805869	1.7557738184	-5.2889482314
34	H	-2.2089976411	0.7579348713	-2.5740987062
35	H	-1.2135712486	2.1314288723	-2.0569343685
36	H	-3.4825901268	-0.0693885997	0.4175334089
37	H	-2.4978230173	-0.7307056299	3.1963953856
38	H	-1.6403581722	0.4845180901	4.1327914542
39	H	-2.3818361088	0.9336694879	2.5877724651
40	H	0.9675967932	-1.3125054545	2.9191043100
41	H	3.0345351008	0.6523414856	3.7405203861
42	H	4.1265846993	0.5717985410	2.3537799763
43	H	3.8920338687	-0.8477871136	3.3781859114

(7*R**,9*R**)-**S2B**, M0004:M0009

I	Atom	X	Y	Z
1	C	-0.4900032785	0.0306699168	-1.4973088191
2	C	-0.5935789111	-1.4205796390	-0.9943829791
3	C	-0.0614145942	-1.3533187810	0.4316746274
4	C	1.0861055519	-0.3518398661	0.2751260020
5	O	0.5739283302	0.6079674968	-0.6800582116
6	C	0.3878782000	-2.1934181139	-1.8645685976
7	N	0.7759161664	-1.3716323873	-2.8715106034
8	C	0.1048746201	-0.0794146202	-2.9174134412
9	C	1.4379197596	0.4082074279	1.5396772028
10	C	0.1896410624	0.9039238569	2.2694656544
11	C	-0.7826826768	-0.2578369980	2.5105977310
12	O	-1.1265401550	-0.7744755777	1.2002559110
13	O	0.7802575903	-3.3227794275	-1.6631701175
14	C	-0.8645152024	-0.0772767333	-4.0751686884
15	O	-2.0112707238	0.2776411381	-4.1143094170
16	O	-0.2182724757	-0.5285607959	-5.1806921786
17	C	-1.7217531256	0.8868701903	-1.2684489830
18	C	-1.3974507850	2.3405785843	-1.4101835379
19	O	-1.7586206274	3.2288608515	-0.6875635822

20	O	-0.6496379761	2.5877452142	-2.5237036355
21	H	-1.5874309784	-1.8392574246	-1.0185111970
22	H	0.2464569494	-2.3089207300	0.8185134068
23	H	1.9684109959	-0.8406483987	-0.1062775638
24	C	-2.0989048457	0.1576613853	3.1296681087
25	O	-0.2122630328	-1.2581748524	3.3179073995
26	O	2.1518881443	-0.5633361551	2.3545932546
27	C	3.2267700964	-0.0660839593	3.1809035134
28	H	0.7828646086	0.7442154626	-3.0475516139
29	H	2.0933089538	1.2295053000	1.2831799801
30	H	1.3738145983	-1.6762626932	-3.6076243782
31	H	0.4616304926	1.3490236460	3.2176081031
32	H	-0.2946783452	1.6451044056	1.6519316334
33	H	-0.7857414843	-0.5289331740	-5.9651765582
34	H	-2.0397259870	0.7121958520	-0.2575103360
35	H	-2.5048791600	0.6390626745	-1.9662006448
36	H	-0.4484030192	3.5296047822	-2.6226666827
37	H	-2.7156398416	-0.7242986513	3.2225456921
38	H	-1.9236235970	0.5724012551	4.1127496620
39	H	-2.5974284424	0.8847183081	2.5050581463
40	H	0.7438114008	-1.3233435094	3.1520103673
41	H	2.8650454379	0.6355521593	3.9215591781
42	H	3.9815128600	0.4137986621	2.5708856685
43	H	3.6524234461	-0.9249160818	3.6740905240

(7*R**,9*R**)-**S2B**, M0004:M0010

I	Atom	X	Y	Z
1	C	-0.4026433105	0.3353608528	-1.5796307377
2	C	-0.7187033456	-1.0831688282	-1.0500034304
3	C	-0.0863497449	-1.1213377062	0.3439022438
4	C	1.1271465405	-0.2072979536	0.1481596477
5	O	0.6152655665	0.8555350929	-0.6932652525
6	C	0.0470908943	-2.0326830111	-1.9514349447
7	N	0.6293573745	-1.2979121317	-2.9380779164
8	C	0.2725002817	0.1010200260	-2.9761171005

9	C	1.6542171684	0.4319312602	1.4203257335
10	C	0.5179199986	0.9563555474	2.2933819039
11	C	-0.5179966868	-0.1418700155	2.5460858842
12	O	-1.0292560658	-0.5217864840	1.2400421872
13	O	0.1768499322	-3.2245048967	-1.7855595235
14	C	-0.6384972153	0.3731806477	-4.1435539533
15	O	-1.2379652015	-0.4320065857	-4.7985170688
16	O	-0.6907168012	1.7073958684	-4.3823494375
17	C	-1.5566420644	1.3419467023	-1.6212360239
18	C	-2.2514561519	1.4195149838	-0.2939142826
19	O	-2.0750981312	2.2325315223	0.5745433498
20	O	-3.1796084523	0.4356471322	-0.2112935035
21	H	-1.7703701879	-1.3074248547	-1.0094520386
22	H	0.1751200359	-2.1179118260	0.6556754811
23	H	1.9119724464	-0.7439547028	-0.3661729929
24	C	-1.7244915146	0.3397188777	3.3239570351
25	O	0.0308344341	-1.2546456362	3.2095299504
26	O	2.3432928950	-0.6016583979	2.1705845128
27	C	3.7479148638	-0.7850574094	1.9081966665
28	H	1.1222970329	0.7599439576	-3.0411878001
29	H	2.3406802481	1.2286957562	1.1653612557
30	H	1.1326015492	-1.7430849028	-3.6756275940
31	H	0.9232777742	1.2988386358	3.2356166165
32	H	0.0268559992	1.7668811444	1.7786433230
33	H	-1.2748630139	1.9311809639	-5.1223718526
34	H	-2.2876581777	1.0302922048	-2.3502327106
35	H	-1.1539534531	2.3080508918	-1.8750596690
36	H	-3.4691671740	0.2743056600	0.6977212388
37	H	-2.4171784426	-0.4863724132	3.4178868719
38	H	-1.4151735965	0.6449729572	4.3144855070
39	H	-2.1892657555	1.1663265378	2.8085945688
40	H	0.9877731040	-1.3139787016	3.0451199319
41	H	4.2943171129	0.1280610043	2.1099904156
42	H	3.9237524545	-1.0875756398	0.8835747627
43	H	4.0860167804	-1.5634561305	2.5736787454

(*7R**,*9R**)-**S2B**, M0004:M0011

I	Atom	X	Y	Z
1	C	-0.5541909875	0.1739055375	-1.3780866380
2	C	-0.7467023709	-1.2784307210	-0.9015326482
3	C	-0.1688928336	-1.2823043176	0.5079941529
4	C	1.0417124636	-0.3621286725	0.3300950949
5	O	0.5684879387	0.6595105069	-0.5794983361
6	C	0.1496678793	-2.1001044509	-1.8169917904
7	N	0.5704901168	-1.2850763757	-2.8169337617
8	C	-0.0134107873	0.0493120744	-2.8190764664
9	C	1.4941965635	0.3283698058	1.6047640369
10	C	0.3107494331	0.8888734540	2.3915154943
11	C	-0.7407289424	-0.1991925466	2.6359026111
12	O	-1.1646121992	-0.6534993973	1.3254462061
13	O	0.4655702644	-3.2588561689	-1.6509555846
14	C	-1.0214850152	0.1410618594	-3.9405182786
15	O	-2.1264832114	0.6117575784	-3.9363323824
16	O	-0.4700284039	-0.3757308998	-5.0683835855
17	C	-1.7205526723	1.1021738017	-1.0999993594
18	C	-1.3391879950	2.5401000054	-1.2689820705
19	O	-1.6871936924	3.4541372507	-0.5726556554
20	O	-0.5598591198	2.7414060619	-2.3691920400
21	H	-1.7687286797	-1.6237573060	-0.9072725307
22	H	0.0851276646	-2.2651807500	0.8650759451
23	H	1.8569190548	-0.9096541443	-0.1194050829
24	C	-2.0016937266	0.3059505736	3.3030729091
25	O	-0.2287192622	-1.2623285647	3.3999475285
26	O	2.1311388034	-0.6678404807	2.4451683195
27	C	3.5393498646	-0.9115770638	2.2550094459
28	H	0.7157825754	0.8261192599	-2.9605682845
29	H	2.1981789915	1.1127123247	1.3600388634
30	H	1.1080252013	-1.6197553013	-3.5861631870
31	H	0.6655168575	1.2689767420	3.3391737728
32	H	-0.1370654873	1.6876188874	1.8201243975
33	H	-1.0674152741	-0.3186588910	-5.8282899538

34	H	-2.0040496641	0.9497139761	-0.0746723180
35	H	-2.5511289228	0.8923985794	-1.7544226601
36	H	-0.3317698581	3.6756448233	-2.4820098427
37	H	-2.6846189835	-0.5258235033	3.3978526618
38	H	-1.7646249093	0.6827106495	4.2885658560
39	H	-2.4594310583	1.0841488919	2.7094274343
40	H	0.7340611005	-1.3374961954	3.2820710070
41	H	4.0963144249	0.0151988123	2.3178690142
42	H	3.7363995278	-1.3834401571	1.3015207068
43	H	3.8448853312	-1.5709655484	3.0513069993

(7*R**,9*R**)-**S2B,** M0004:M0012

I	Atom	X	Y	Z
1	C	0.3284714666	0.3005969788	-1.6861887462
2	C	-1.1049338100	0.5889508901	-1.1798479415
3	C	-1.1274925544	0.0382026009	0.2489824864
4	C	-0.1608335367	-1.1439410038	0.1343475998
5	O	0.8837980946	-0.6370141135	-0.7327191612
6	C	-2.0201321189	-0.2669322303	-2.0349372366
7	N	-1.2561588617	-0.9011557897	-2.9649323584
8	C	0.1252898932	-0.4906300468	-3.0291009832
9	C	0.4993818042	-1.5633510220	1.4334499078
10	C	0.9700322374	-0.3544767862	2.2384401928
11	C	-0.1790334847	0.6411033088	2.4248432207
12	O	-0.5762844803	1.0583586531	1.0922597993
13	O	-3.2121662726	-0.4096677951	-1.8794670214
14	C	0.4441808199	0.2785325837	-4.2909290451
15	O	1.5337732396	0.7129815577	-4.5613846293
16	O	-0.6116152228	0.3970634461	-5.1195635569
17	C	1.2967436751	1.4832890192	-1.7882765767
18	C	1.3250709378	2.2616708266	-0.5066078613
19	O	2.1298903177	2.1777146010	0.3833751448
20	O	0.3022504473	3.1523739129	-0.4952686991
21	H	-1.3715071354	1.6311776961	-1.1994941138
22	H	-2.1158298007	-0.2456230474	0.5676752730

23	H	-0.6536781976	-1.9931874466	-0.3140144831
24	C	0.2363195545	1.9098191888	3.1378792522
25	O	-1.2673567431	0.0753470181	3.1169311689
26	O	-0.5382561657	-2.2871996470	2.1515703299
27	C	-0.0922545232	-3.3601210751	3.0076638790
28	H	0.8117541042	-1.3237141851	-2.9880438096
29	H	1.3233520601	-2.2272495424	1.2073850806
30	H	-1.6796146692	-1.4423151810	-3.6874905485
31	H	1.3364363452	-0.6684496621	3.2074594614
32	H	1.7568612328	0.1410700750	1.6922076121
33	H	-0.3829890506	0.8706825902	-5.9335847739
34	H	0.9920349755	2.1530157259	-2.5735685030
35	H	2.2806663221	1.0967791603	-1.9957852567
36	H	0.1251032806	3.4988152689	0.3905813800
37	H	-0.6221979515	2.5666660808	3.1867789333
38	H	0.5471658802	1.6716175148	4.1461339771
39	H	1.0444152437	2.3831680668	2.6014518740
40	H	-1.3317348000	-0.8757315468	2.9255326295
41	H	0.5451169201	-2.9917220346	3.8015033726
42	H	0.4450106202	-4.1036790783	2.4320552850
43	H	-0.9790500937	-3.8028355308	3.4326974451

(7*R**,9*R**)-**S2B**, M0004:M0013

ATOM		X	Y	Z
1	C	0.4107678900	0.4082639736	-1.5613152419
2	C	-1.0126861854	0.7245830983	-1.0426895832
3	C	-1.0626558359	0.0976438230	0.3540938680
4	C	-0.1477865415	-1.1168540690	0.1700259599
5	O	0.9210234554	-0.6096570952	-0.6652863759
6	C	-1.9557311625	-0.0492352163	-1.9445097966
7	N	-1.2120386471	-0.6599568806	-2.9071536798
8	C	0.1822499433	-0.2905104490	-2.9502085579
9	C	0.4815543159	-1.6390661880	1.4492505741
10	C	0.9958293448	-0.4998269899	2.3245203146
11	C	-0.1057713814	0.5356200647	2.5639711651
12	O	-0.4743432354	1.0436766269	1.2534465552

13	O	-3.1523828956	-0.1598466158	-1.7977302255
14	C	0.5230405214	0.5468929526	-4.1619898406
15	O	1.6264892249	0.9579956090	-4.4117989897
16	O	-0.5323056410	0.7585938925	-4.9726081648
17	C	1.4288336069	1.5523223370	-1.5892162975
18	C	1.4823418336	2.2533295080	-0.2643491589
19	O	2.2781677936	2.0844687092	0.6214199091
20	O	0.4965743382	3.1831949501	-0.2046645927
21	H	-1.2385683504	1.7760342451	-1.0069208163
22	H	-2.0623112220	-0.1634328106	0.6567523092
23	H	-0.6814434964	-1.9034691428	-0.3447011311
24	C	0.3672791499	1.7444722399	3.3433493852
25	O	-1.2245447217	-0.0130357319	3.2178639244
26	O	-0.5565151936	-2.3294624325	2.1922932020
27	C	-0.7335255689	-3.7348827469	1.9304555693
28	H	0.8416255924	-1.1460285883	-2.9641296689
29	H	1.2823096243	-2.3239867230	1.2026015962
30	H	-1.6536348589	-1.1384500660	-3.6624934449
31	H	1.3294270520	-0.9025418856	3.2711514351
32	H	1.8109843717	-0.0092169864	1.8166877764
33	H	-0.2911283279	1.2766251216	-5.7554084622
34	H	1.1583561157	2.2794197215	-2.3350374605
35	H	2.3969476975	1.1367760315	-1.8149183193
36	H	0.3261744507	3.4825795920	0.6995584747
37	H	-0.4605300205	2.4362858782	3.4284125614
38	H	0.6644006392	1.4377921870	4.3372353132
39	H	1.1974851398	2.2094258891	2.8339417552
40	H	-1.2801715644	-0.9706092393	3.0560380792
41	H	0.1793280662	-4.2783796072	2.1412797553
42	H	-1.0262224941	-3.9131109566	0.9033430964
43	H	-1.5168928225	-4.0744360296	2.5894372289

(7*R**,9*R**)-**S2B,** M0004:M0014

I	Atom	X	Y	Z
1	C	-0.3738032629	0.0756052948	-1.5566181463

2	C	-0.5019448772	-1.3880090768	-1.1168247833
3	C	-0.0037511505	-1.3742688791	0.3225342549
4	C	1.1603646433	-0.3815625131	0.2304026046
5	O	0.7074993171	0.5999535620	-0.7364477798
6	C	0.4771315809	-2.1270391756	-2.0133630352
7	N	0.8370281203	-1.2783615310	-3.0145031231
8	C	0.1792799192	0.0253724687	-2.9956411630
9	C	1.4685722676	0.3660668767	1.5135274852
10	C	0.1929923078	0.8668577970	2.1902736622
11	C	-0.7875679254	-0.2948169107	2.3946067719
12	O	-1.0912278097	-0.8150325140	1.0774310354
13	O	0.8975672844	-3.2501010588	-1.8439781133
14	C	-0.8871116654	0.0207691264	-4.0560644010
15	O	-0.8929014271	0.6349941744	-5.0848409317
16	O	-1.8744256211	-0.8579645897	-3.7249772674
17	C	-1.5964868978	0.9487125648	-1.2958544177
18	C	-1.1435390479	2.3706572699	-1.2396336117
19	O	-1.1789380748	3.1171335067	-0.2991527790
20	O	-0.6402552164	2.7401775841	-2.4492086103
21	H	-1.4990113493	-1.7850303055	-1.2017701468
22	H	0.2875773511	-2.3426753702	0.6914479606
23	H	2.0503318914	-0.8772658399	-0.1246208489
24	C	-2.1214778568	0.1288229584	2.9702977073
25	O	-0.2514833258	-1.2972117279	3.2239042263
26	O	2.1474907822	-0.6091162601	2.3523674072
27	C	3.1878994294	-0.1063974914	3.2176208476
28	H	0.8544927242	0.8385350072	-3.1716271505
29	H	2.1364089868	1.1862393075	1.2864245576
30	H	1.5118985376	-1.5365487444	-3.7017439367
31	H	0.4280663039	1.3089080556	3.1500491131
32	H	-0.2620345595	1.6133907382	1.5554040929
33	H	-2.5431914426	-0.9464733582	-4.4202227015
34	H	-1.9902412938	0.6698606624	-0.3364235617
35	H	-2.3345609303	0.8106111094	-2.0678283140
36	H	-0.2983645350	3.6460208265	-2.4560945393
37	H	-2.7518971948	-0.7464245058	3.0364375863

38	H	-1.9753217159	0.5348612876	3.9619120958
39	H	-2.5905484122	0.8671392576	2.3354771097
40	H	0.7092884340	-1.3661599810	3.0902373476
41	H	2.7934239239	0.5941089909	3.9427489385
42	H	3.9624262645	0.3773304540	2.6356920221
43	H	3.6003455222	-0.9616690477	3.7286425354

(7*R**,9*R**)-**S2B,** M0004:M0015

I	Atom	X	Y	Z
1	C	-0.4618707559	0.1550237351	-1.4499625957
2	C	-0.6525736639	-1.2969235408	-0.9912936459
3	C	-0.0994370801	-1.3015765790	0.4275005113
4	C	1.1151052939	-0.3789255896	0.2763367861
5	O	0.6854993360	0.6202251389	-0.6828264044
6	C	0.2516049123	-2.0923734183	-1.9177642717
7	N	0.6038873396	-1.2762190865	-2.9481471121
8	C	0.0196031614	0.0615471597	-2.9123994692
9	C	1.5195046983	0.3498086632	1.5460081284
10	C	0.3042714898	0.9225938084	2.2724755959
11	C	-0.7369684338	-0.1737244573	2.5233384989
12	O	-1.1217410150	-0.6823809970	1.2215914010
13	O	0.6315958734	-3.2300646784	-1.7476722366
14	C	-1.0947257407	0.1094114839	-3.9219200815
15	O	-1.1153123849	0.7221813545	-4.9518171673
16	O	-2.1105876565	-0.7151176146	-3.5432034182
17	C	-1.6215930645	1.0983408695	-1.1436815303
18	C	-1.0979063092	2.4974535341	-1.1179584619
19	O	-1.1071344982	3.2668682022	-0.1948511746
20	O	-0.5738457079	2.8206660026	-2.3311596386
21	H	-1.6721639029	-1.6398939381	-1.0387637119
22	H	0.1543360490	-2.2821565865	0.7929612364
23	H	1.9474636515	-0.9365541067	-0.1282650618
24	C	-2.0215290849	0.3403627595	3.1384532970
25	O	-0.2373275159	-1.2028588382	3.3409566408
26	O	2.1413962174	-0.6204479491	2.4272808210

27	C	3.5637598957	-0.8143823082	2.3026046763
28	H	0.7310016112	0.8337104937	-3.1287913299
29	H	2.2227019533	1.1348638840	1.3005170447
30	H	1.2309269229	-1.5768194043	-3.6631605434
31	H	0.6220065854	1.3400010395	3.2182732225
32	H	-0.1322548668	1.6979120510	1.6599646075
33	H	-2.8134201220	-0.7681175947	-4.2080961777
34	H	-1.9883498878	0.8460896230	-0.1661747666
35	H	-2.3984509132	0.9968887896	-1.8829073511
36	H	-0.1914917744	3.7103567160	-2.3500856384
37	H	-2.7039731365	-0.4920434651	3.2371226024
38	H	-1.8153040120	0.7456180313	4.1198056818
39	H	-2.4655585594	1.1011979275	2.5119532624
40	H	0.7282821859	-1.2749757848	3.2461641606
41	H	4.0904972129	0.1161016038	2.4755588696
42	H	3.8274810661	-1.1983934979	1.3253919715
43	H	3.8425946303	-1.5332734361	3.0566427728

(7*R**,9*R**)-**S2B**, M0004:M0016

I	Atom	X	Y	Z
1	C	-0.4203760712	0.2060312572	-1.4316050605
2	C	-0.6155245903	-1.2525765472	-0.9853454937
3	C	-0.0670494025	-1.2747901070	0.4328831745
4	C	1.1439908485	-0.3503158586	0.2936358684
5	O	0.6777804320	0.6918269235	-0.5975372012
6	C	0.3055235226	-2.0524033080	-1.8908906744
7	N	0.7116328860	-1.2311391388	-2.8914976348
8	C	0.1582171025	0.1264995222	-2.8670979801
9	C	1.5816038796	0.2960100622	1.5954987094
10	C	0.3929024396	0.8452769201	2.3812839637
11	C	-0.6773833760	-0.2365772924	2.5688334444
12	O	-1.0746996658	-0.6438177022	1.2357472342
13	O	0.6575407884	-3.1998111061	-1.7175397749
14	C	-0.8349670792	0.1858025084	-3.9940968327
15	O	-0.6398188902	0.6078936147	-5.0974009900

16	O	-1.9991504378	-0.4370808050	-3.6526775873
17	C	-1.6161203113	1.1062295673	-1.1889851463
18	C	-1.3805749440	2.4832968818	-1.7188365521
19	O	-0.7095369304	2.7556529112	-2.6845785291
20	O	-2.0630445913	3.4186752166	-1.0278007498
21	H	-1.6346738601	-1.5979988852	-1.0337198085
22	H	0.1763390974	-2.2610120076	0.7877223175
23	H	1.9685869338	-0.8850043805	-0.1552760757
24	C	-1.9478951559	0.2631713543	3.2225808957
25	O	-0.1962633643	-1.3261816158	3.3148319499
26	O	2.1852938569	-0.7466701891	2.4060195665
27	C	3.6021196626	-0.9740933430	2.2654940811
28	H	0.9042883909	0.8801603992	-3.0153405758
29	H	2.3033349843	1.0762655549	1.3927178349
30	H	1.3758616577	-1.5296911951	-3.5733781527
31	H	0.7342378097	1.1923909492	3.3466979294
32	H	-0.0336930863	1.6662099552	1.8255612800
33	H	-2.6116615172	-0.5041141396	-4.4005722533
34	H	-1.8028920836	1.1304028344	-0.1326113128
35	H	-2.4823527571	0.7007794681	-1.6913881017
36	H	-1.9494532450	4.3058314244	-1.4004663454
37	H	-2.6371638875	-0.5663943717	3.2871068980
38	H	-1.7293407525	0.6195761395	4.2200930982
39	H	-2.3907040651	1.0547683020	2.6348469848
40	H	0.7659756490	-1.4191253813	3.2027198803
41	H	4.1566679081	-0.0720631793	2.4925460013
42	H	3.8539172154	-1.3071907104	1.2670630432
43	H	3.8585249999	-1.7447005025	2.9747586771

(7*R**,9*R**)-**S2B,** M0004:M0017

I	Atom	X	Y	Z
1	C	0.1209755088	0.3543792823	-1.5743867316
2	C	-1.3448109535	0.5043843513	-1.1400521740
3	C	-1.3515601924	-0.0018982195	0.2941306413
4	C	-0.3717485027	-1.1748040785	0.2021877028

5	O	0.6427330829	-0.7053134393	-0.7204713039
6	C	-2.0978059892	-0.4722487548	-2.0293862780
7	N	-1.2442610981	-0.8820536579	-3.0046341943
8	C	0.0642522938	-0.2306074786	-3.0026054123
9	C	0.3191220925	-1.5249685778	1.5059428347
10	C	0.8127704972	-0.2770344630	2.2371066487
11	C	-0.3313077932	0.7313096379	2.4029857339
12	O	-0.7789880057	1.0653115460	1.0684101209
13	O	-3.2347604767	-0.8570600231	-1.8650526453
14	C	0.0273754013	0.8085228464	-4.0916077127
15	O	0.5738451495	0.7736604335	-5.1561638118
16	O	-0.8141713974	1.8256975206	-3.7406955814
17	C	0.9949363426	1.5744323138	-1.3599336483
18	C	2.4242138770	1.2263578374	-1.6425053757
19	O	2.8040249770	0.5128761299	-2.5360875143
20	O	3.2780250397	1.8369130410	-0.7917179637
21	H	-1.7298436598	1.5057271860	-1.2260899346
22	H	-2.3280260556	-0.2810326074	0.6503362672
23	H	-0.8661294406	-2.0509119703	-0.1873191599
24	C	0.0964197909	2.0448755915	3.0206530150
25	O	-1.3811828009	0.2046219275	3.1781854483
26	O	-0.7051704795	-2.1960560866	2.2908085671
27	C	-0.2689777007	-3.2812901375	3.1369392838
28	H	0.8766109115	-0.9071121602	-3.1736202053
29	H	1.1343477597	-2.2056703636	1.3001315387
30	H	-1.5089052854	-1.5675572066	-3.6788859921
31	H	1.2025498391	-0.5428167626	3.2112970241
32	H	1.5970243566	0.1747690032	1.6481166368
33	H	-0.9399594495	2.4660789815	-4.4567162372
34	H	0.8786014446	1.8875948053	-0.3395870526
35	H	0.6825713867	2.3721096158	-2.0188859030
36	H	4.2025026729	1.6331063923	-0.9982059813
37	H	-0.7687009117	2.6907507820	3.0652738707
38	H	0.4616883963	1.8735688286	4.0240980759
39	H	0.8649684457	2.5122892045	2.4213546932
40	H	-1.4644917548	-0.7526218704	3.0276283318

41	H	0.4206828499	-2.9362408496	3.8970214827
42	H	0.2055811283	-4.0542715424	2.5456788328
43	H	-1.1550212972	-3.6777670083	3.6063240630

(7*R**,9*R**)-**S2B**, M0004:M0018

I	Atom	X	Y	Z
1	C	-0.4501329964	0.1738716310	-1.4762126297
2	C	-0.6769360446	-1.2756448913	-1.0303883638
3	C	-0.1204217522	-1.3050312372	0.3873247897
4	C	1.1164606809	-0.4129697045	0.2392930824
5	O	0.6986782610	0.6129010981	-0.6934683846
6	C	0.2152072362	-2.0840193777	-1.9596743704
7	N	0.6241031994	-1.2583074287	-2.9608617893
8	C	0.0510247037	0.0878296649	-2.9312666987
9	C	1.5505456338	0.2864204847	1.5146918382
10	C	0.3554613642	0.8855114212	2.2535673344
11	C	-0.7179706741	-0.1824000434	2.4941244959
12	O	-1.1217043293	-0.6586058018	1.1873319102
13	O	0.5458574015	-3.2396641806	-1.8075285452
14	C	-1.0308948401	0.1192519874	-3.9770712779
15	O	-0.9672553185	0.6145010986	-5.0650330977
16	O	-2.1132763248	-0.6067136219	-3.5707360226
17	C	-1.5810531877	1.1478492726	-1.1918925614
18	C	-1.0534526305	2.5313315611	-1.4082783877
19	O	-0.5503290523	2.9202689868	-2.4322007192
20	O	-1.1845460293	3.3180521540	-0.3168134143
21	H	-1.7037363650	-1.5955043796	-1.0760412417
22	H	0.1058789190	-2.2942691422	0.7463527131
23	H	1.9305327821	-0.9846651591	-0.1814703044
24	C	-1.9839305107	0.3582575438	3.1242208770
25	O	-0.2422460961	-1.2358214584	3.2947590063
26	O	2.1462219673	-0.7091129673	2.3850186218
27	C	3.5565515107	-0.9677517525	2.2355740089
28	H	0.7686721020	0.8569657365	-3.1359455890
29	H	2.2748849557	1.0533604087	1.2736204299

30	H	1.2390058353	-1.5701266071	-3.6815008262
31	H	0.6885156565	1.2810651993	3.2031980093
32	H	-0.0605189798	1.6785648786	1.6490894445
33	H	-2.7844590718	-0.6876663165	-4.2649642992
34	H	-1.8829064416	1.0133464208	-0.1722195092
35	H	-2.4049634531	0.9548731403	-1.8600623453
36	H	-0.8259342332	4.2060460917	-0.4648221529
37	H	-2.6856727034	-0.4580383047	3.2183094418
38	H	-1.7609437179	0.7474196185	4.1082965635
39	H	-2.4134301024	1.1351690202	2.5076862578
40	H	0.7210199809	-1.3320761284	3.1972502417
41	H	4.1282170671	-0.0632099879	2.4022202301
42	H	3.7864277473	-1.3600772582	1.2535261875
43	H	3.8134478500	-1.7011816697	2.9829970463

(7*R**,9*R**)-**S2B,** M0004:M0019

I	Atom	X	Y	Z
1	C	0.1339585467	0.2970449247	-1.5486821219
2	C	-1.3298086422	0.4736126455	-1.1124625748
3	C	-1.3337654310	0.0195097569	0.3389455433
4	C	-0.3635585203	-1.1622451587	0.2846610150
5	O	0.6602973140	-0.7175683315	-0.6391228032
6	C	-2.1022246129	-0.5291178355	-1.9529132231
7	N	-1.2647918864	-0.9867086372	-2.9177644691
8	C	0.0723814510	-0.3866744418	-2.9380697537
9	C	0.3055673431	-1.4836715575	1.6070841816
10	C	0.8074679981	-0.2245394480	2.3125553839
11	C	-0.3209402184	0.8085911460	2.4270010687
12	O	-0.7393943945	1.1023117395	1.0723951998
13	O	-3.2427134291	-0.8924220447	-1.7613510284
14	C	0.0938849101	0.5268409767	-4.1319539109
15	O	0.5292669489	0.2760491843	-5.2186903155
16	O	-0.5821332461	1.6829463256	-3.8721936230
17	C	0.9915499814	1.5383711057	-1.3982356468
18	C	2.3779222426	1.3093520079	-1.9066724386

19	O	2.6769249539	0.5752672957	-2.8167859690
20	O	3.2873165435	2.0746622971	-1.2689580019
21	H	-1.7082994621	1.4757114028	-1.2251794254
22	H	-2.3116842166	-0.2340638252	0.7090649057
23	H	-0.8593846418	-2.0461031950	-0.0856665416
24	C	0.1165616603	2.1366611716	3.0053924812
25	O	-1.3919074733	0.3249992775	3.2004299993
26	O	-0.7442418460	-2.1121291932	2.3952837976
27	C	-0.3535329379	-3.2051361820	3.2534489919
28	H	0.8521840070	-1.1139453258	-3.0355042311
29	H	1.1113211371	-2.1847363816	1.4357017865
30	H	-1.5415494342	-1.7079111064	-3.5490384686
31	H	1.1745443701	-0.4708370830	3.3006206057
32	H	1.6098366787	0.1960761236	1.7259220146
33	H	-0.6756172944	2.2379881332	-4.6609558112
34	H	1.0062771067	1.8056447782	-0.3589240592
35	H	0.5583368551	2.3492890275	-1.9655343975
36	H	4.1789692236	1.9628462234	-1.6312555481
37	H	-0.7419973957	2.7924145421	3.0198651911
38	H	0.4711792442	1.9956425286	4.0173680699
39	H	0.8950600508	2.5756115397	2.3976021638
40	H	-1.4859036716	-0.6361840700	3.0839581357
41	H	0.3516160875	-2.8812034252	4.0087132988
42	H	0.0861538430	-4.0050680630	2.6713126727
43	H	-1.2551297429	-3.5571788486	3.7285878557

(7*R**,9*R**)-**S2B**, M0004:M0020

I	Atom	X	Y	Z
1	C	0.3249030365	-0.2042244257	-1.4579974014
2	C	0.3986478595	1.2420708398	-0.9314875308
3	C	-0.1636014373	1.1526874682	0.4767438905
4	C	-1.2485163734	0.0904153968	0.3115122808
5	O	-0.6340599869	-0.8704590947	-0.5815738683
6	C	-0.5079243237	2.0532553480	-1.8350422180
7	N	-0.9082486539	1.2463812355	-2.8538865261

8	C	-0.3733090228	-0.0986801137	-2.8495094755
9	C	-1.6128499984	-0.6430680175	1.5875851599
10	C	-0.3673179147	-1.0659427271	2.3661913904
11	C	0.5622846589	0.1349830022	2.5815339238
12	O	0.9158319214	0.6218199184	1.2668273697
13	O	-0.8427973553	3.2046824493	-1.6631143637
14	C	0.4429578413	-0.3365980367	-4.0872892008
15	O	0.4890040334	0.3649217123	-5.0571817026
16	O	1.0427491155	-1.5547579778	-4.0323885858
17	C	1.6028334059	-1.0116565382	-1.3220252159
18	C	2.7445491160	-0.5816362254	-2.1869074614
19	O	3.7710343580	-1.1749460937	-2.3646410758
20	O	2.5127809901	0.6351996423	-2.7637592226
21	H	1.3938371721	1.6452689349	-0.9272637220
22	H	-0.5228091806	2.0911399282	0.8620750577
23	H	-2.1360069466	0.5182439474	-0.1299349576
24	C	1.8746259137	-0.2182007020	3.2465607083
25	O	-0.0599207617	1.1386589494	3.3489061615
26	O	-2.3840495526	0.3225449766	2.3536987550
27	C	-3.4578303143	-0.2032438515	3.1631293992
28	H	-1.1511277041	-0.8497964673	-2.8419917821
29	H	-2.2278593071	-1.4983257869	1.3412611716
30	H	-1.4112319639	1.6085007189	-3.6351742369
31	H	-0.6469899058	-1.4807794974	3.3258945552
32	H	0.1539108871	-1.8140203111	1.7890225619
33	H	1.5186494394	-1.7763063258	-4.8465818288
34	H	1.4045216552	-2.0508714935	-1.5311842673
35	H	1.9004427927	-0.9104133948	-0.2870770290
36	H	3.2462624087	0.9361576648	-3.3205620722
37	H	2.4598591300	0.6859662118	3.3324500840
38	H	1.6875715846	-0.6135674779	4.2355750694
39	H	2.4158767378	-0.9420855564	2.6538777693
40	H	-1.0141154004	1.1632273680	3.1639597804
41	H	-3.0841028922	-0.8738195931	3.9265660814
42	H	-4.1751701984	-0.7277653414	2.5445998816
43	H	-3.9332948635	0.6450393368	3.6286026926

(7*R**,9*R**)-**S2B**, M0004:M0021

ATOM		X	Y	Z
1	C	-0.2998143984	0.3081286447	-1.3964971325
2	C	-0.4781923427	-1.1367432883	-0.9000689255
3	C	0.0849437831	-1.1063124805	0.5114252838
4	C	1.2638940870	-0.1483860591	0.3453001965
5	O	0.7481025626	0.8670462383	-0.5514006858
6	C	0.4046336278	-1.9703296593	-1.8071472171
7	N	0.8062178070	-1.1794592196	-2.8395639321
8	C	0.3162020975	0.1797432139	-2.8275848572
9	C	1.6996117662	0.5321230012	1.6306857328
10	C	0.5024398909	1.0599955239	2.4183756677
11	C	-0.5379627568	-0.0456750021	2.6298145643
12	O	-0.9359838221	-0.4886995075	1.3112732114
13	O	0.7332497067	-3.1222212099	-1.6260933306
14	C	-0.7062905399	0.4095763223	-3.9032458394
15	O	-1.3529278731	-0.4173518726	-4.4826430766
16	O	-0.8390176359	1.7427457768	-4.0928587049
17	C	-1.5002199203	1.2435422053	-1.2280446123
18	C	-2.7388606171	0.8991575920	-2.0068177052
19	O	-3.3204205238	1.6232753911	-2.7740232691
20	O	-3.1881413956	-0.3481323228	-1.7325731356
21	H	-1.5009576022	-1.4663074479	-0.9203502013
22	H	0.3621538997	-2.0755417199	0.8886428592
23	H	2.0959276163	-0.6625576913	-0.1145847764
24	C	-1.8124981973	0.4350621455	3.2903752096
25	O	-0.0204124513	-1.1086211191	3.3930221397
26	O	2.3426939371	-0.4759268587	2.4521264502
27	C	3.7629143022	-0.6606162501	2.2880104719
28	H	1.0942085674	0.9181672117	-2.9200225627
29	H	2.3942985602	1.3314502049	1.4074338156
30	H	1.3512229196	-1.5510527201	-3.5877082167
31	H	0.8409683037	1.4280075865	3.3770563485
32	H	0.0508839054	1.8632591500	1.8561041757
33	H	-1.6458877644	1.9848182586	-4.5720302310

34	H	-1.2084519681	2.2436624869	-1.5005117494
35	H	-1.7420204301	1.1859440735	-0.1768608017
36	H	-3.9712156572	-0.5816300778	-2.2543282129
37	H	-2.4853678645	-0.4066170353	3.3718072035
38	H	-1.5913955392	0.8080268389	4.2812674468
39	H	-2.2782754074	1.2102324326	2.6979329855
40	H	0.9438392983	-1.1728865844	3.2809720631
41	H	4.2913454407	0.2625005319	2.4921027024
42	H	4.0057989283	-0.9989204263	1.2889854957
43	H	4.0587636997	-1.4124762783	3.0022451514

(7*S**,9*S**)-**S2C,** M0001:M0001

I	Atom	X	Y	Z
1	C	-0.0204326187	-0.1348656184	-1.3585004227
2	C	0.4731422526	-1.4752306644	-0.8118805584
3	C	0.8871358237	-1.1908280316	0.6173845558
4	C	1.3921216360	0.2668101654	0.5455009060
5	O	0.8463740069	0.8214029226	-0.6757434885
6	C	1.7047276442	-1.7642169188	-1.6629458101
7	N	1.6124102192	-1.0049956736	-2.7802028905
8	C	0.4785014971	-0.0907983345	-2.8170240047
9	C	0.9494817003	1.0738618488	1.7743877358
10	C	0.9469847156	0.1271989592	2.9758052154
11	C	-0.1910474584	-0.8994529645	2.8196572966
12	O	-0.2910617945	-1.2878619644	1.4237857604
13	O	2.6044117088	-2.5233493359	-1.3702994823
14	C	-0.4964675517	-0.5571432328	-3.8667645041
15	O	-1.6850067099	-0.7111415392	-3.7716038050
16	O	0.1651696420	-0.7590486321	-5.0306953993
17	C	-1.4660572143	0.1978725893	-1.0571608240
18	C	-1.8703567431	1.4923921498	-1.6833454400
19	O	-1.2809448197	2.1040842947	-2.5398892891
20	O	-3.0380566305	1.9389937741	-1.1642292141
21	H	-0.2459431486	-2.2786458239	-0.8604961016
22	H	1.6551121360	-1.8765544874	0.9420457595
23	H	2.4698672618	0.2807508992	0.4747615650
24	O	-0.3906572997	1.5784972634	1.6213128910
25	C	-0.5182697433	2.8855886660	1.0069859758
26	H	0.7630659637	0.9217357842	-3.0434625894
27	H	1.6244852958	1.9048600210	1.9375529403
28	H	2.2838522733	-1.0429384122	-3.5149050057
29	H	1.9098888089	-0.3647117731	3.0464880783
30	H	0.7673557766	0.6695169594	3.8924329546
31	H	-0.4257498637	-1.0370725702	-5.7457752903
32	H	-1.5640909580	0.2677145003	0.0119136949
33	H	-2.1327989627	-0.5643208793	-1.4278132195

34	H	-3.3528687549	2.7382102857	-1.6115727583
35	H	-0.0241387761	2.8957277149	0.0512409438
36	H	-0.1017597734	3.6463432799	1.6563298242
37	H	-1.5766158267	3.0484685784	0.8799156322
38	O	-1.4177955185	-0.3227185174	3.1813937436
39	H	-1.5194116095	0.4972834885	2.6708982403
40	C	-0.0055584735	-2.1434380383	3.6644694183
41	H	0.8953348104	-2.6716431800	3.3801062623
42	H	-0.8617875368	-2.7835329025	3.5095936618
43	H	0.0474546135	-1.8728046504	4.7103470418

(7*S**,9*S**)-**S2C**, M0001:M0002

ATOM		X	Y	Z
1	C	0.0852022618	0.1445725507	-1.3842060551
2	C	0.6042781747	-1.1895236896	-0.8501117333
3	C	0.9750343552	-0.9138844366	0.5893385113
4	C	1.4404042819	0.5559525094	0.5553943570
5	O	0.8773655181	1.1273093463	-0.6527160616
6	C	1.8461408311	-1.4488495997	-1.6859997791
7	N	1.7669914912	-0.6615617501	-2.7898856788
8	C	0.6318644667	0.2304644917	-2.8374585336
9	C	0.9747005346	1.3232184174	1.8023679337
10	C	0.9582664659	0.3453522592	2.9780878802
11	C	-0.1576462429	-0.6961248337	2.7665123637
12	O	-0.2252830191	-1.0441011155	1.3609340701
13	O	2.7497464225	-2.2058102592	-1.4038715153
14	C	-0.3845297388	-0.1261309591	-3.8909415075
15	O	-1.3745879684	0.5330446302	-4.0919715769
16	O	-0.0559301602	-1.2021205422	-4.6178196012
17	C	-1.3795504332	0.4558840001	-1.1446141973
18	C	-2.3196191865	-0.5930520087	-1.6384645212
19	O	-2.0299806657	-1.6429115597	-2.1620990226
20	O	-3.5974856711	-0.2327196757	-1.4128966089
21	H	-0.1015808611	-1.9950931600	-0.9487727705
22	H	1.7492480410	-1.5833765311	0.9338346589
23	H	2.5176910974	0.5997775763	0.4843869656

24	O	-0.3655308366	1.8297642868	1.6476826608
25	C	-0.4779974038	3.1595384326	1.0860609352
26	H	0.9197441106	1.2586805466	-3.0016636298
27	H	1.6446044873	2.1511228138	2.0003758477
28	H	2.4228752482	-0.7408542710	-3.5353596383
29	H	1.9284490336	-0.1298677812	3.0642011865
30	H	0.7437536485	0.8630051344	3.9017612180
31	H	-0.7522503720	-1.4428003046	-5.2466601400
32	H	-1.6498776534	1.3899632406	-1.6163043224
33	H	-1.5041824713	0.5525049935	-0.0770254293
34	H	-4.2247937672	-0.9029496774	-1.7216176482
35	H	-0.0241906500	3.1895197192	0.1107706425
36	H	-0.0068551043	3.8832395341	1.7413686609
37	H	-1.5337439146	3.3715809966	1.0171496228
38	O	-1.4010576835	-0.1483222085	3.1220190565
39	H	-1.5024066199	0.6860908339	2.6355560485
40	C	0.0326693364	-1.9590558250	3.5818167112
41	H	0.9487660210	-2.4640706862	3.3029612277
42	H	-0.8074933132	-2.6115141481	3.3922263342
43	H	0.0587779092	-1.7158912905	4.6356530780

(7*S**,9*S**)-**S2C**, M0001:M0003

I	Atom	X	Y	Z
1	C	-0.0790672802	-0.5793809150	-1.4161201906
2	C	1.3724220791	-0.2367363080	-1.0098405423
3	C	1.2291482641	0.4183239473	0.3660520348
4	C	-0.1127247192	1.1376274290	0.2208698971
5	O	-0.9176052418	0.1751747772	-0.5094071834
6	C	1.8315976924	0.8182129931	-1.9979080618
7	N	0.8517139063	0.9770143381	-2.9271019989
8	C	-0.2518355018	0.0476366672	-2.8425070899
9	C	-0.7689483275	1.4511665084	1.5553759210
10	C	-0.8543163172	0.1893403750	2.3967661112
11	C	0.5082699879	-0.4689062976	2.6040122337
12	O	1.1392283685	-0.6570260540	1.3062460153

13	O	2.8578076026	1.4570174256	-1.9287186704
14	C	-0.1679345231	-0.9562585278	-3.9621988007
15	O	0.7765150542	-1.1785961640	-4.6661408442
16	O	-1.3530573422	-1.6030980096	-4.0873521391
17	C	-0.4959506011	-2.0562035588	-1.3456045313
18	C	-0.1960090709	-2.6207171124	0.0089843946
19	O	-0.9712153030	-2.7199006779	0.9314093310
20	O	1.0817318015	-3.0424981171	0.0794944185
21	H	2.0310202931	-1.0872240892	-0.9756785896
22	H	2.0512785785	1.0815269936	0.5859593243
23	H	0.0181239241	2.0288731053	-0.3769927735
24	O	-2.1132225032	1.9174423231	1.4192894484
25	C	-2.2977050673	3.1949221720	0.7903063676
26	H	-1.2176046977	0.5239966523	-2.8738767688
27	H	-0.1629855593	2.2096151581	2.0440347349
28	H	0.9814036538	1.5861799165	-3.7067357032
29	H	-1.2931999273	0.4169357894	3.3573942620
30	H	-1.4983452427	-0.4946597271	1.8663505166
31	H	-1.3442974348	-2.2657580228	-4.7944615763
32	H	0.0605796724	-2.6290551891	-2.0704313957
33	H	-1.5537949577	-2.1201248115	-1.5375756189
34	H	1.4056620040	-3.0923534839	0.9930298888
35	H	-2.0623732664	3.1602265857	-0.2662346812
36	H	-1.6912094907	3.9614354639	1.2638137670
37	H	-3.3407109470	3.4432777781	0.9101763771
38	O	0.3672862631	-1.7622231477	3.1426383778
39	H	-0.2884194584	-2.2658583523	2.6302790763
40	C	1.4448132653	0.2837728889	3.5321902693
41	H	1.6500450238	1.2851941526	3.1774007624
42	H	2.3727861115	-0.2666132314	3.5964840076
43	H	1.0010992344	0.3382783564	4.5163296227

(7*S**,9*S**)-**S2C**, M0001:M0004

I	Atom	X	Y	Z
1	C	-0.1274932541	-0.6297069691	-1.3883509018

2	C	1.3222664348	-0.2706724877	-0.9874207659
3	C	1.1789627220	0.3935982882	0.3852184018
4	C	-0.1690551404	1.1010851655	0.2377950922
5	O	-0.9678385906	0.1259703456	-0.4809378345
6	C	1.7687366008	0.7863220661	-1.9796835190
7	N	0.7747069648	0.9531730725	-2.8919734321
8	C	-0.3148435451	0.0090680109	-2.8107703982
9	C	-0.8236227190	1.4205060351	1.5716382504
10	C	-0.8945498962	0.1658036142	2.4245459856
11	C	0.4752628004	-0.4761123426	2.6350489456
12	O	1.1047680531	-0.6723258755	1.3366012936
13	O	2.8001296391	1.4190497342	-1.9268631623
14	C	-0.3220567898	-0.9656453293	-3.9666352449
15	O	-1.1308576719	-1.8461688840	-4.1048315099
16	O	0.6520406792	-0.7253013310	-4.8660030949
17	C	-0.5425437489	-2.1054350824	-1.2988876425
18	C	-0.2240360880	-2.6539245610	0.0576701094
19	O	-0.9850407777	-2.7541227788	0.9915741841
20	O	1.0596641507	-3.0641499863	0.1161339135
21	H	1.9887512059	-1.1146721725	-0.9483768647
22	H	1.9962970806	1.0663507655	0.5939805543
23	H	-0.0466758897	1.9881937349	-0.3676558316
24	O	-2.1726886882	1.8727006908	1.4352026394
25	C	-2.3717388990	3.1427473876	0.7958273435
26	H	-1.2826525313	0.4885104444	-2.7944407246
27	H	-0.2232960329	2.1892427377	2.0510146030
28	H	0.9072451773	1.5367602120	-3.6897915957
29	H	-1.3335255374	0.3977279292	3.3840460567
30	H	-1.5319229945	-0.5301048759	1.9017979632
31	H	0.6165392858	-1.3411908842	-5.6134991146
32	H	-0.0115859768	-2.6899612743	-2.0297549595
33	H	-1.6036855427	-2.1697732171	-1.4724591389
34	H	1.3916501687	-3.1106071004	1.0266567400
35	H	-2.1391223712	3.1014719948	-0.2610612815
36	H	-1.7717037103	3.9195368709	1.2607276026
37	H	-3.4168117385	3.3814085401	0.9167126726

38	O	0.3496013120	-1.7634203622	3.1893691093
39	H	-0.2945578341	-2.2835492476	2.6783393748
40	C	1.4073358110	0.2968894218	3.5510107176
41	H	1.6008901521	1.2962208809	3.1842154135
42	H	2.3409074684	-0.2434543167	3.6184239041
43	H	0.9661502616	0.3579611360	4.5358461461

(7*S**,9*S**)-**S2C,** M0001:M0005

I	Atom	X	Y	Z
1	C	-0.4955211249	0.0565820154	-1.5895521277
2	C	-0.1451080704	-1.4011808620	-1.2112923405
3	C	0.4811629309	-1.2789429635	0.1792703213
4	C	1.1821836235	0.0753565417	0.0836232270
5	O	0.2069242797	0.8801667680	-0.6311060888
6	C	0.9339946119	-1.8277988229	-2.1875679633
7	N	1.1074525083	-0.8231602250	-3.0855533107
8	C	0.1786514315	0.2795536179	-2.9878747588
9	C	1.4679637369	0.6941487500	1.4348739089
10	C	0.1784738698	0.7760460968	2.2392015969
11	C	-0.4731060937	-0.5996158494	2.4064006326
12	O	-0.6185025327	-1.2168106540	1.0989322457
13	O	1.5756093570	-2.8533060880	-2.1314884304
14	C	-0.7882696821	0.2426698177	-4.1420029372
15	O	-0.9829420995	-0.6683979234	-4.8963660362
16	O	-1.4383529454	1.4295432475	-4.2333640549
17	C	-1.9807362188	0.4476429599	-1.5591451142
18	C	-2.5760363175	0.1136668433	-0.2265936694
19	O	-2.7076158866	0.8690007579	0.7087729092
20	O	-2.9814595859	-1.1705417808	-0.1900591319
21	H	-0.9886463607	-2.0692115424	-1.2076575534
22	H	1.1466129127	-2.0987720740	0.3996105514
23	H	2.0934892764	0.0030088650	-0.4909155578
24	O	2.0400253513	1.9799489995	1.1731888369
25	C	2.7067659363	2.6148956265	2.2761498335
26	H	0.6585846999	1.2439262937	-2.9654142444

27	H	2.1970673406	0.0664819884	1.9400658201
28	H	1.7438067752	-0.9226520046	-3.8475623434
29	H	0.3459706515	1.1911138729	3.2236226284
30	H	-0.4965403660	1.4164970269	1.6927476234
31	H	-2.0751660029	1.4502885106	-4.9635059212
32	H	-2.5210656105	-0.1048253836	-2.3117043078
33	H	-2.0559207642	1.5076850497	-1.7328363302
34	H	-3.0528857139	-1.5118597206	0.7155305066
35	H	3.4856856938	1.9776991258	2.6844017687
36	H	2.0178278811	2.8783526510	3.0687752091
37	H	3.1527876149	3.5147187262	1.8820881275
38	O	-1.7801509540	-0.4759406866	2.9150686918
39	H	-2.2820987570	0.1700666323	2.3882678867
40	C	0.2624822221	-1.5400220340	3.3453926247
41	H	1.2732478169	-1.7381708811	3.0144140323
42	H	-0.2850144675	-2.4709032945	3.3856665610
43	H	0.2883690319	-1.1069479941	4.3354966778

(7*S**,9*S**)-**S2C**, M0001:M0006

ATOM		X	Y	Z
1	C	0.1122221208	0.0859013551	-1.3984781966
2	C	0.6240388724	-1.2495978974	-0.8598570335
3	C	0.9840313255	-0.9728645939	0.5821924886
4	C	1.4709094419	0.4898579903	0.5421791881
5	O	0.9168921305	1.0628424528	-0.6697872625
6	C	1.8845811141	-1.4936441030	-1.6749874398
7	N	1.8231591893	-0.6969796266	-2.7731543258
8	C	0.6626701836	0.1647136813	-2.8461285003
9	C	1.0164162598	1.2684908369	1.7866179250
10	C	0.9791703480	0.2928071768	2.9641050363
11	C	-0.1561601419	-0.7275993481	2.7532604178
12	O	-0.2215684781	-1.0820800633	1.3485032752
13	O	2.7905016911	-2.2409360656	-1.3761417150
14	C	-0.2595279861	-0.3020086878	-3.9400042920
15	O	-0.0703959491	-1.1965213203	-4.7104744695
16	O	-1.3377677455	0.5261006619	-4.0205739350

17	C	-1.3443977119	0.4161285074	-1.1422176972
18	C	-2.3011287918	-0.5996735638	-1.6628440871
19	O	-2.0343163130	-1.6607038253	-2.1736854878
20	O	-3.5764291207	-0.1987210715	-1.4752176328
21	H	-0.0793647761	-2.0562421494	-0.9700439093
22	H	1.7472553229	-1.6506904787	0.9338244333
23	H	2.5485779829	0.5164428155	0.4714126150
24	O	-0.3143622775	1.7986220225	1.6288724426
25	C	-0.4016095223	3.1256605544	1.0569737370
26	H	0.9202930652	1.1959391483	-3.0388570945
27	H	1.7003362271	2.0847055440	1.9847024708
28	H	2.5002901549	-0.7575457824	-3.5019259072
29	H	1.9395709564	-0.2011589058	3.0521890368
30	H	0.7738269151	0.8162457059	3.8864310450
31	H	-1.9418373052	0.2631392433	-4.7309369612
32	H	-1.5974585940	1.3705153197	-1.5782614818
33	H	-1.4617800612	0.4779090394	-0.0713743012
34	H	-4.2135361144	-0.8720848081	-1.7563643520
35	H	0.0480886856	3.1374074914	0.0796087349
36	H	0.0872375514	3.8450208198	1.7039967644
37	H	-1.4530085437	3.3590702743	0.9907852441
38	O	-1.3908306379	-0.1527063553	3.0976061185
39	H	-1.4681593293	0.6871540281	2.6160490357
40	C	0.0035644026	-1.9900016998	3.5756350202
41	H	0.9107552558	-2.5147777877	3.3047700628
42	H	-0.8485695245	-2.6257141637	3.3832362624
43	H	0.0278197271	-1.7424223714	4.6283647277

(7*S**,9*S**)-**S2C,** M0001:M0007

I	Atom	X	Y	Z
1	C	-0.0975272505	-0.1559127888	-1.3471506099
2	C	0.4392288444	-1.4781788835	-0.7904004241
3	C	0.8435495521	-1.1692642106	0.6356292449
4	C	1.3218143982	0.2938520993	0.5438482044
5	O	0.7152570131	0.8390323806	-0.6554102053

6	C	1.6864372548	-1.7360383072	-1.6283658677
7	N	1.5968642160	-0.9621230827	-2.7351157090
8	C	0.4151516048	-0.1106130723	-2.8006424336
9	C	0.9147056150	1.0933651643	1.7917011832
10	C	0.9108163118	0.1380952454	2.9871340883
11	C	-0.2410728082	-0.8729712807	2.8363761857
12	O	-0.3329963869	-1.2766811855	1.4418311511
13	O	2.5971811444	-2.4801453129	-1.3314254902
14	C	-0.5072007528	-0.6629901667	-3.8574156166
15	O	-1.6718280561	-0.9470407060	-3.7825574930
16	O	0.1887909201	-0.7900612624	-5.0155042994
17	C	-1.5627322334	0.1012972986	-1.0512752334
18	C	-2.0230420309	1.4621869715	-1.4684483912
19	O	-2.8383679957	2.1447802656	-0.9076926822
20	O	-1.4425455265	1.8782162443	-2.6307708050
21	H	-0.2550519824	-2.3033488694	-0.8326334222
22	H	1.6239556058	-1.8363183186	0.9690940760
23	H	2.3955116351	0.3208956921	0.4290959526
24	O	-0.4151348386	1.6230307692	1.6591861191
25	C	-0.5097085106	2.9681063676	1.1279953499
26	H	0.6497102659	0.9079835399	-3.0463107028
27	H	1.6092909484	1.9079727813	1.9546825393
28	H	2.2689427565	-0.9875443880	-3.4696293837
29	H	1.8674946524	-0.3661818801	3.0511848322
30	H	0.7431293626	0.6808410475	3.9057749224
31	H	-0.3572852649	-1.1362836736	-5.7362979716
32	H	-1.6995387517	0.0072200040	0.0111900165
33	H	-2.1748288207	-0.6192061481	-1.5717690340
34	H	-1.7838804236	2.7391982649	-2.9141317042
35	H	0.0139289774	3.0284523361	0.1879329978
36	H	-0.0946647951	3.6779363197	1.8334773359
37	H	-1.5573805082	3.1568059937	0.9754307661
38	O	-1.4620495593	-0.2785001478	3.1815913868
39	H	-1.5462214827	0.5445692868	2.6710639128
40	C	-0.0774406887	-2.1128218254	3.6913844450
41	H	0.8197718570	-2.6531710448	3.4188921734

42	H	-0.9398382299	-2.7434529068	3.5328884833
43	H	-0.0311960387	-1.8349886103	4.7355621126

(7*S**,9*S**)-**S2C,** M0001:M0008

I	Atom	X	Y	Z
1	C	-0.0275720889	0.0559064014	-1.4004712002
2	C	-0.5228353040	1.4095492479	-0.8947869115
3	C	-0.9203060626	1.1691475539	0.5460793596
4	C	-1.4274562172	-0.2902034695	0.5244789031
5	O	-0.8939950973	-0.8822477080	-0.6866839448
6	C	-1.7674094316	1.6572169983	-1.7355182730
7	N	-1.6709200939	0.8784045313	-2.8407771807
8	C	-0.5299841228	-0.0443316844	-2.8577050689
9	C	-0.9795864068	-1.0565357736	1.7775504301
10	C	-0.9670988544	-0.0689383335	2.9452776214
11	C	0.1734103365	0.9478893630	2.7467921292
12	O	0.2657783257	1.2873047909	1.3380867516
13	O	-2.6842883750	2.3964180077	-1.4469548653
14	C	0.3977821224	0.4432373731	-3.9352191090
15	O	0.3270163921	0.1467366627	-5.0941358183
16	O	1.2701527514	1.3830056942	-3.4816716129
17	C	1.4225739296	-0.2523794024	-1.0732400289
18	C	1.9117388113	-1.4624219790	-1.7982190867
19	O	1.5173377501	-1.8601585753	-2.8678391342
20	O	2.9167049903	-2.0831627395	-1.1368082199
21	H	0.1919464070	2.2102822363	-0.9927328072
22	H	-1.6846813025	1.8647303081	0.8581495296
23	H	-2.5059187374	-0.3045629797	0.4632698481
24	O	0.3572740655	-1.5732061655	1.6330265666
25	C	0.4691301214	-2.9030474219	1.0639841198
26	H	-0.8254394485	-1.0496261063	-3.0830407859
27	H	-1.6569268993	-1.8782110639	1.9741456551
28	H	-2.4019905646	0.8312941691	-3.5176956141
29	H	-1.9277436061	0.4290857426	3.0032963377
30	H	-0.7851118443	-0.5795517452	3.8795725328

31	H	1.8080813120	1.7518221149	-4.1985102212
32	H	1.4936297834	-0.3984710751	-0.0126756503
33	H	2.0501246314	0.5763166198	-1.3656729218
34	H	3.2822962149	-2.8240256670	-1.6439123574
35	H	-0.1388367989	-2.9759375300	0.1777525444
36	H	0.1701137740	-3.6473101203	1.7914957100
37	H	1.5063254667	-3.0273146682	0.8034601125
38	O	1.4009912046	0.3802926207	3.1216497991
39	H	1.4979875578	-0.4575977951	2.6403989961
40	C	-0.0047751346	2.2205346378	3.5497731019
41	H	-0.9053466097	2.7415359307	3.2512152194
42	H	0.8525504691	2.8527914018	3.3703019759
43	H	-0.0547234164	1.9857395970	4.6045135681

(7*S**,9*S**)-**S2C**, M0001:M0009

I	Atom	X	Y	Z
1	C	-0.5667503683	0.0918353887	-1.5547594825
2	C	-0.2074971771	-1.3644019062	-1.1738485174
3	C	0.4456186445	-1.2366049908	0.2048347994
4	C	1.1388897857	0.1199186684	0.0915929837
5	O	0.1445948826	0.9185856853	-0.6020619441
6	C	0.8529768633	-1.8006059226	-2.1659080294
7	N	1.0290856901	-0.7952125394	-3.0614835703
8	C	0.1155488227	0.3181082414	-2.9514173324
9	C	1.4508024720	0.7420963130	1.4353077626
10	C	0.1771458239	0.8229492491	2.2643691928
11	C	-0.4659639804	-0.5545922413	2.4495570169
12	O	-0.6336486419	-1.1797771327	1.1478390392
13	O	1.4767927180	-2.8385282253	-2.1268053091
14	C	-0.8161323728	0.4167947772	-4.1383188556
15	O	-1.7153521207	1.2107426041	-4.2365352929
16	O	-0.4992736398	-0.4450695472	-5.1253732835
17	C	-2.0508299603	0.4832618323	-1.4979102148
18	C	-2.6153713881	0.1495876150	-0.1520029247
19	O	-2.7316018829	0.9023472891	0.7874905317

20	O	-3.0167400167	-1.1371498049	-0.1065331971
21	H	-1.0511261152	-2.0317932246	-1.1475976081
22	H	1.1195579496	-2.0532109759	0.4116765382
23	H	2.0380190841	0.0496203164	-0.5022599260
24	O	2.0147805061	2.0283409342	1.1580264978
25	C	2.6986528340	2.6705175134	2.2462117616
26	H	0.6263845515	1.2676843254	-2.8736675423
27	H	2.1914851034	0.1173342512	1.9271542953
28	H	1.6225883796	-0.9110210395	-3.8543419138
29	H	0.3622183021	1.2419992777	3.2439634146
30	H	-0.5110651582	1.4582120760	1.7285596637
31	H	-1.0795830004	-0.3445686748	-5.8950184655
32	H	-2.6098920321	-0.0565194271	-2.2424179152
33	H	-2.1309304355	1.5431137766	-1.6699262207
34	H	-3.0749647803	-1.4751143296	0.8010691126
35	H	3.4868629801	2.0378653952	2.6435963475
36	H	2.0229525566	2.9353943671	3.0496242595
37	H	3.1345209748	3.5699797622	1.8401749809
38	O	-1.7629725491	-0.4334574410	2.9823284127
39	H	-2.2786466617	0.2041940037	2.4581103608
40	C	0.2909913684	-1.4880845033	3.3784521830
41	H	1.2961340734	-1.6835138170	3.0292155444
42	H	-0.2520250684	-2.4208488199	3.4330945620
43	H	0.3337629835	-1.0504090995	4.3659382848

(7*S**,9*S**)-**S2C**, M0001:M0010

I	Atom	X	Y	Z
1	C	-0.3805902033	-0.0243999860	-1.4484526975
2	C	0.0239334939	-1.4614797613	-1.0480709572
3	C	0.6643186495	-1.2973734431	0.3307738268
4	C	1.3171078200	0.0788755260	0.2153494768
5	O	0.3050600819	0.8385327046	-0.5100124481
6	C	1.0952797301	-1.8765181924	-2.0369637591
7	N	1.2159629798	-0.8886873438	-2.9628918676
8	C	0.2678365833	0.1959804119	-2.8602694530

9	C	1.6035408255	0.7049564407	1.5705640926
10	C	0.3175936617	0.7565565735	2.3872208062
11	C	-0.2884904588	-0.6392857304	2.5670484834
12	O	-0.4259541211	-1.2628926073	1.2615471204
13	O	1.7681313294	-2.8809285096	-1.9742522937
14	C	-0.7144096668	0.1356706689	-4.0014556661
15	O	-0.8946932630	-0.7763953989	-4.7577067367
16	O	-1.3947864123	1.3070720967	-4.0798632369
17	C	-1.8800103755	0.3086268192	-1.4052045600
18	C	-2.4392744498	-0.0095073217	-0.0525235710
19	O	-2.5719693232	0.7686454387	0.8643918422
20	O	-2.8065814460	-1.3016619462	0.0261717723
21	H	-0.7993474907	-2.1541238230	-1.0174062971
22	H	1.3620318178	-2.0895199191	0.5527449005
23	H	2.2230793300	0.0291348944	-0.3713159208
24	O	2.2914305333	1.9546468938	1.4675575048
25	C	1.5564411767	3.1206708581	1.0340813285
26	H	0.7302365026	1.1694687433	-2.8576853537
27	H	2.3207728988	0.0604175583	2.0607650525
28	H	1.8363519496	-0.9884614768	-3.7381128474
29	H	0.5057164938	1.1850421433	3.3622697516
30	H	-0.4075393070	1.3610088006	1.8643705863
31	H	-2.0415823980	1.3146377961	-4.8017172101
32	H	-2.4067617001	-0.2904608620	-2.1319264261
33	H	-2.0060886648	1.3580770360	-1.6115603443
34	H	-2.8437437196	-1.6230378328	0.9415719900
35	H	0.8170203837	3.4160950487	1.7676655400
36	H	1.0702992139	2.9493779799	0.0877456043
37	H	2.2916192768	3.9055777787	0.9412759971
38	O	-1.5983298428	-0.5537361633	3.0792210616
39	H	-2.1162155855	0.0879942629	2.5637184070
40	C	0.4796504037	-1.5527563932	3.5051825963
41	H	1.4949526425	-1.7198138650	3.1719234208
42	H	-0.0378378915	-2.5005513449	3.5506812742
43	H	0.4958385419	-1.1154745536	4.4935492102

(7*S**,9*S**)-**S2C,** M0001:M0011

I	Atom	X	Y	Z
1	C	-0.2975121526	-0.0607782422	-1.4541463682
2	C	0.4836481802	-1.3258162112	-1.0288415844
3	C	0.9398349733	-1.0226894385	0.4000196630
4	C	1.1616204564	0.4879183419	0.3455014628
5	O	0.0264208576	0.9354607160	-0.4524979253
6	C	1.7061967214	-1.3759173277	-1.9243544060
7	N	1.6161424923	-0.3544016677	-2.8157254072
8	C	0.3838311110	0.3963481304	-2.7942901959
9	C	1.1472421877	1.1301190840	1.7227465616
10	C	-0.1516292011	0.7737358500	2.4355752405
11	C	-0.3263540549	-0.7436440968	2.5582245232
12	O	-0.1747839807	-1.3434409919	1.2422483830
13	O	2.6288805259	-2.1533488509	-1.8216208918
14	C	-0.4399964127	0.1968146140	-4.0465648600
15	O	-1.5174782481	0.6993936747	-4.2347424426
16	O	0.1780048209	-0.5666063572	-4.9679403428
17	C	-1.8273388212	-0.1768332315	-1.5255899065
18	C	-2.3624673919	-0.6869886845	-0.2230102631
19	O	-2.7840696514	-0.0175752298	0.6920652408
20	O	-2.3344593634	-2.0338635830	-0.1889208216
21	H	-0.0967143888	-2.2312757692	-1.0700190008
22	H	1.8239007400	-1.5789639164	0.6695480638
23	H	2.0828768964	0.7248754446	-0.1665743233
24	O	1.4331012806	2.5308589715	1.6777911726
25	C	0.4119260774	3.4340667095	1.1975505539
26	H	0.5471773035	1.4599699420	-2.6948575527
27	H	1.9870902824	0.7157167513	2.2639982666
28	H	2.2779025405	-0.2595538055	-3.5556990216
29	H	-0.1712866316	1.2089374417	3.4254082917
30	H	-0.9839015274	1.1511610692	1.8617491906
31	H	-0.3474012479	-0.6584117010	-5.7772291916
32	H	-2.1140317964	-0.8655118916	-2.3016875182
33	H	-2.2364848646	0.7986362454	-1.7272290560

34	H	-2.3429739240	-2.3831317135	0.7166357915
35	H	-0.4268709211	3.4801886244	1.8803177433
36	H	0.0608474785	3.1447126113	0.2208413440
37	H	0.8832880580	4.4041325953	1.1542609307
38	O	-1.6349938345	-1.0648713313	2.9672422883
39	H	-2.2821366920	-0.5966192098	2.4119075982
40	C	0.6084375933	-1.4148143263	3.5483124903
41	H	1.6487551118	-1.2648959314	3.2934521925
42	H	0.3932914614	-2.4739443049	3.5493698163
43	H	0.4224679555	-1.0191490033	4.5367742707

(*7R**,*9S**)-**S2D**, M0002:M0001

I	Atom	X	Y	Z
1	C	0.0967376891	0.3544905798	-1.7416824008
2	C	-1.2818471377	-0.0898115111	-1.1983343633
3	C	-0.9928449474	-0.5186089475	0.2436200323
4	C	0.4172047858	-1.0815572788	0.1183671896
5	O	1.0632698777	-0.1515238445	-0.7908673354
6	C	-1.6609811717	-1.3160699471	-2.0055485326
7	N	-0.7069684249	-1.5020492460	-2.9580672041
8	C	0.2814664352	-0.4553737469	-3.0716882210
9	C	1.1820684230	-1.1068423306	1.4251028045
10	C	1.1178714386	0.2482842415	2.1183479117
11	C	-0.3094695762	0.7778213694	2.2870383878
12	O	-0.9853391724	0.6964780796	1.0043913857
13	O	-2.6086822814	-2.0393058791	-1.7986206304
14	C	0.0324632940	0.3594337903	-4.3137842139
15	O	-0.9610098716	0.3748376796	-4.9840610393
16	O	1.1287522464	1.1059429919	-4.5994533177
17	C	0.3274215466	1.8639689363	-1.9111279436
18	C	0.0491083456	2.5788978267	-0.6250230845
19	O	0.8662280111	2.8802687358	0.2147461663
20	O	-1.2590609894	2.8764126469	-0.5133341781
21	H	-2.0344736392	0.6786165634	-1.2355255913
22	H	-1.7045252833	-1.2331539590	0.6202635558
23	H	0.3910741347	-2.0735362935	-0.3049944466
24	C	-1.1289740674	0.0901644160	3.3611510562
25	O	-0.2901516062	2.1529699483	2.6078763956
26	O	0.5334273888	-2.1615010848	2.1606501707
27	C	1.2840575213	-2.7386813352	3.2434033826
28	H	1.2943568015	-0.8213082589	-3.0936885215
29	H	2.2153124396	-1.3682657972	1.2274272364
30	H	-0.7949318114	-2.2323391699	-3.6321946344
31	H	1.5957119684	0.2212150915	3.0884107253
32	H	1.6534905673	0.9437233636	1.4881787453
33	H	1.0109206674	1.6528584132	-5.3907168223

34	H	-0.3439808855	2.2540583397	-2.6596175222
35	H	1.3535764979	2.0211590038	-2.1973568518
36	H	-1.5354562243	2.9942061987	0.4099128767
37	H	-1.1773409246	-0.9702446780	3.1816617274
38	H	-2.1201258427	0.5218328961	3.3608040727
39	H	-0.6708333044	0.2756520769	4.3234955508
40	H	0.2847578053	2.6325149352	1.9873291424
41	H	1.4579170481	-2.0224950182	4.0364723323
42	H	2.2380910326	-3.1174002915	2.8925916691
43	H	0.6917111959	-3.5557395065	3.6244443378

(7*R**,9*S**)-**S2D,** M0002:M0002

ATOM		X	Y	Z
1	C	-0.4187228330	0.1457027963	-1.7239784571
2	C	0.0401764728	-1.2316711443	-1.1869038211
3	C	0.4803520124	-0.9456301594	0.2531440107
4	C	1.0324930825	0.4685976157	0.1270848791
5	O	0.0895112185	1.1113296229	-0.7699567843
6	C	1.2657093470	-1.6007944202	-2.0004129266
7	N	1.4566898451	-0.6293300004	-2.9342261266
8	C	0.4005992108	0.3477327305	-3.0484926701
9	C	1.0666939120	1.2288125544	1.4363242702
10	C	-0.2795221246	1.1504395775	2.1449425112
11	C	-0.7947661930	-0.2819984119	2.3151914125
12	O	-0.7246149520	-0.9533564546	1.0287187099
13	O	1.9827943815	-2.5578995709	-1.8134792156
14	C	-0.3902823029	0.1989655368	-4.3288440334
15	O	-1.3234870509	0.8961821198	-4.6332406327
16	O	0.0814008481	-0.7746914742	-5.1315745657
17	C	-1.9295894165	0.3775451662	-1.8737387074
18	C	-2.6256338571	0.0804821516	-0.5819241894
19	O	-2.9252959862	0.8833723339	0.2722322587
20	O	-2.9111253925	-1.2327603523	-0.4810105201
21	H	-0.7233626062	-1.9895661851	-1.2190179882
22	H	1.2051787487	-1.6535503377	0.6178359400
23	H	2.0204354447	0.4500889113	-0.3060498510

24	C	-0.0850860366	-1.0995432647	3.3766023696
25	O	-2.1646139291	-0.2753212695	2.6549546953
26	O	2.1363264250	0.5876908940	2.1572162218
27	C	2.7166659242	1.3392588888	3.2374956359
28	H	0.7664814366	1.3638434971	-3.0199337122
29	H	1.3163705998	2.2650859730	1.2389662246
30	H	2.1622617067	-0.7264822290	-3.6323072289
31	H	-0.2457798194	1.6254334216	3.1163730708
32	H	-0.9871633792	1.6816937864	1.5247562458
33	H	-0.4194570982	-0.8390339090	-5.9588704401
34	H	-2.3353529841	-0.2688099897	-2.6325354615
35	H	-2.0902516850	1.4086980688	-2.1410557013
36	H	-3.0237716811	-1.5178082383	0.4400748153
37	H	0.9735425428	-1.1368521960	3.1833809289
38	H	-0.5072496771	-2.0949003993	3.3776949748
39	H	-0.2629413369	-0.6471442273	4.3432178624
40	H	-2.6584038763	0.2914896617	2.0378069144
41	H	2.0075231241	1.5002627255	4.0396110207
42	H	3.0801560733	2.2996338565	2.8876219582
43	H	3.5451118614	0.7548023439	3.6063061028

(7*R**,9*S**)-**S2D**, M0002:M0003

I	Atom	X	Y	Z
1	C	-0.4687322742	0.1821176318	-1.6118834463
2	C	-0.1359429728	-1.2392580325	-1.0995548167
3	C	0.3607842399	-1.0129668421	0.3317954450
4	C	1.0505921398	0.3394603680	0.2026184646
5	O	0.1597327754	1.0837618585	-0.6692713629
6	C	1.0266020436	-1.7138609048	-1.9491763088
7	N	1.2653661925	-0.7694988649	-2.8994247750
8	C	0.3080473883	0.3099356447	-2.9678905410
9	C	1.1862814862	1.0788738807	1.5198307117
10	C	-0.1413261091	1.1257143757	2.2611831329
11	C	-0.8010445947	-0.2468290240	2.4267785047
12	O	-0.8213235260	-0.9057835215	1.1308806603

13	O	1.6732228363	-2.7214793436	-1.7721813981
14	C	-0.5660196500	0.1512202402	-4.1844375872
15	O	-0.6785589339	-0.8225099032	-4.8740993027
16	O	-1.2366500676	1.3064551916	-4.4204251882
17	C	-1.9550371372	0.5538785428	-1.7246627483
18	C	-2.6481139067	0.3310512401	-0.4155996964
19	O	-2.8389580777	1.1642237673	0.4402368880
20	O	-3.0638927184	-0.9444421461	-0.3025677551
21	H	-0.9707398238	-1.9183493808	-1.1225595671
22	H	1.0176760458	-1.7941705292	0.6751281569
23	H	2.0142165729	0.2178166422	-0.2707401763
24	C	-0.1709389493	-1.1447571429	3.4726841897
25	O	-2.1612037542	-0.1002998624	2.7802517002
26	O	2.1486674687	0.3545628961	2.3104698608
27	C	3.5230723914	0.7650414059	2.1956165022
28	H	0.7637058114	1.2856130202	-2.9940389684
29	H	1.5309742526	2.0886791571	1.3316311304
30	H	1.9584357440	-0.9171161163	-3.6019744592
31	H	-0.0017432021	1.5684322294	3.2371107211
32	H	-0.8091851397	1.7433217269	1.6781907360
33	H	-1.8189795035	1.2472661915	-5.1927887515
34	H	-2.4315458533	-0.0719388941	-2.4628170781
35	H	-2.0269678719	1.5927266674	-1.9988353755
36	H	-3.1638465677	-1.2198173635	0.6234204275
37	H	0.8841065262	-1.2587463442	3.2940650995
38	H	-0.6732735478	-2.1019895621	3.4468502974
39	H	-0.3221313740	-0.6987921922	4.4461791437
40	H	-2.5953469810	0.5358599302	2.1867716978
41	H	3.6380760676	1.8112032794	2.4557974041
42	H	3.9104766160	0.6063947407	1.1958267882
43	H	4.0814659379	0.1589953420	2.8916116402

(7*R**,9*S**)-**S2D,** M0002:M0004

I	Atom	X	Y	Z
1	C	-0.5154962521	0.2363472491	-1.5941997912

2	C	-0.1708132364	-1.1853005900	-1.0883623172
3	C	0.3279373822	-0.9645084631	0.3441198897
4	C	1.0069274619	0.3940325582	0.2211407922
5	O	0.1089870547	1.1362669643	-0.6440937805
6	C	0.9962711237	-1.6480636563	-1.9390022732
7	N	1.2469579556	-0.6862936949	-2.8685199658
8	C	0.2790825278	0.3815735709	-2.9412590132
9	C	1.1408453278	1.1271633655	1.5420207943
10	C	-0.1829074929	1.1543980040	2.2909421808
11	C	-0.8269645811	-0.2261476416	2.4517502032
12	O	-0.8502462079	-0.8755537113	1.1504417228
13	O	1.6350475213	-2.6640819272	-1.7796400806
14	C	-0.5616447344	0.3191676960	-4.1964843372
15	O	-1.4221606556	1.1141113375	-4.4734678036
16	O	-0.2260099005	-0.7020527701	-5.0080889611
17	C	-2.0017572148	0.6098123361	-1.6963746521
18	C	-2.6861219331	0.3653129794	-0.3870681509
19	O	-2.8869782149	1.1844979493	0.4799335325
20	O	-3.0862395207	-0.9175916308	-0.2875704837
21	H	-1.0004255599	-1.8706687960	-1.1121898999
22	H	0.9936147719	-1.7426076222	0.6780594441
23	H	1.9708438617	0.2819916447	-0.2541754737
24	C	-0.1771551410	-1.1245897230	3.4852125582
25	O	-2.1844935932	-0.0971062164	2.8187373012
26	O	2.1173395565	0.4090508791	2.3214202756
27	C	3.4838355505	0.8446464345	2.2066951237
28	H	0.7341708931	1.3607410082	-2.9147675721
29	H	1.4724865079	2.1420712048	1.3577327032
30	H	1.9173098693	-0.8398892229	-3.5907401387
31	H	-0.0435332114	1.5923756197	3.2691245608
32	H	-0.8607412267	1.7677989110	1.7151730998
33	H	-0.7581391669	-0.7122076293	-5.8181052231
34	H	-2.4876103255	0.0113448160	-2.4475537979
35	H	-2.0737353784	1.6539311163	-1.9509586861
36	H	-3.1883477561	-1.2018304611	0.6352908390
37	H	0.8772403895	-1.2268713492	3.2953977436

38	H	-0.6705823905	-2.0864967208	3.4591255421
39	H	-0.3225711001	-0.6856720700	4.4628795785
40	H	-2.6313617813	0.5367800509	2.2322846990
41	H	3.5835831354	1.8865548650	2.4892635058
42	H	3.8663980587	0.7149321503	1.2006978958
43	H	4.0571576263	0.2326311858	2.8851784158

(7*R**,9*S**)-**S2D,** M0002:M0005

I	Atom	X	Y	Z
1	C	-0.3031246063	-0.0383504383	-1.5906870178
2	C	0.5788478218	-1.1864847303	-1.0715231654
3	C	0.9819805069	-0.7901425987	0.3446701263
4	C	0.9583787453	0.7470809767	0.3107615341
5	O	0.0734452004	1.1019063533	-0.7974314422
6	C	1.8212269225	-1.1334790236	-1.9507102498
7	N	1.5659334668	-0.2918753467	-2.9838451251
8	C	0.2256341353	0.2424249482	-3.0338306453
9	C	0.4090595657	1.3593358222	1.6004720717
10	C	0.6939269100	0.4368667339	2.7829176159
11	C	-0.0168194437	-0.9126832025	2.6250706897
12	O	-0.0206261815	-1.3011881964	1.2272919908
13	O	2.8677867618	-1.6982480757	-1.7260039568
14	C	-0.5752580627	-0.4606851737	-4.0972590024
15	O	-0.3230210138	-1.5093675197	-4.6194648435
16	O	-1.6763597898	0.2715109622	-4.4001404712
17	C	-1.8286057075	-0.2335395623	-1.5273162108
18	C	-2.2593821050	-0.5216520645	-0.1208179265
19	O	-2.4718955876	0.3074913454	0.7377532055
20	O	-2.3806408082	-1.8395101933	0.0941454124
21	H	0.1071003035	-2.1560245519	-1.0878453506
22	H	1.9559142706	-1.1832430910	0.5908119952
23	H	1.9424914598	1.1430106415	0.1143985244
24	C	0.6124693330	-2.0190133467	3.4472404558
25	O	-1.3808271000	-0.8575398602	2.9968558487
26	O	1.0553492873	2.6059396463	1.8892299654

27	C	0.6009773979	3.7189147170	1.0927392398
28	H	0.1932327003	1.3051030115	-3.2034134969
29	H	-0.6507662124	1.4992596761	1.4613672636
30	H	2.2395686157	-0.1378207915	-3.7029409459
31	H	1.7651825879	0.2993655192	2.8632307035
32	H	0.3457963110	0.9012526650	3.6933406181
33	H	-2.2306285960	-0.1497437166	-5.0743297628
34	H	-2.1229012332	-1.0647998993	-2.1454141137
35	H	-2.2938034807	0.6778426763	-1.8662897707
36	H	-2.3309466733	-2.0651092179	1.0380703385
37	H	1.6414342350	-2.1760495459	3.1513309678
38	H	0.0508175686	-2.9280692431	3.2873519765
39	H	0.5735633338	-1.7606444036	4.4967828180
40	H	-1.8727618271	-0.2218672690	2.4489545121
41	H	-0.4457154940	3.9265803075	1.2849765438
42	H	0.7254778874	3.5243144754	0.0353442656
43	H	1.1984885948	4.5689305847	1.3841548141

(7*R**,9*S**)-**S2D**, M0002:M0006

I	Atom	X	Y	Z
1	C	-0.3702329510	0.0024083689	-1.5759694976
2	C	0.5072691960	-1.1510920041	-1.0596901815
3	C	0.9286963876	-0.7566495612	0.3533036855
4	C	0.9073235679	0.7810676038	0.3209039489
5	O	0.0180466060	1.1401265059	-0.7807770740
6	C	1.7450606026	-1.1105143563	-1.9467899614
7	N	1.5024531100	-0.2498658947	-2.9665019714
8	C	0.1689694022	0.2952441757	-3.0167248504
9	C	0.3671536491	1.3908149102	1.6148794137
10	C	0.6830635152	0.4749837128	2.7938276749
11	C	-0.0233385879	-0.8788072824	2.6532254727
12	O	-0.0556428176	-1.2699645090	1.2551491791
13	O	2.7803066546	-1.7024875607	-1.7377339150
14	C	-0.6675515178	-0.2990797492	-4.1249053993
15	O	-1.7750503406	0.0834648685	-4.4030676622

16	O	-0.0543441633	-1.2995688902	-4.7834458566
17	C	-1.8972090077	-0.1695257941	-1.4987636351
18	C	-2.3050464500	-0.4981771060	-0.0949231242
19	O	-2.5234492495	0.3004746774	0.7900756342
20	O	-2.3928859689	-1.8257004447	0.0846529569
21	H	0.0268010908	-2.1163474574	-1.0674289299
22	H	1.9067369060	-1.1501344704	0.5824180425
23	H	1.8913475923	1.1757186035	0.1209496760
24	C	0.6311030384	-1.9800128090	3.4627632616
25	O	-1.3774297335	-0.8291228135	3.0553082893
26	O	0.9991695815	2.6477685711	1.8891549524
27	C	0.5164873202	3.7514318280	1.0961167165
28	H	0.1572284263	1.3676425934	-3.1366735858
29	H	-0.6969254198	1.5139486757	1.4917578012
30	H	2.1612310112	-0.1303708568	-3.7050424218
31	H	1.7566951212	0.3449555221	2.8535066847
32	H	0.3489160191	0.9390094052	3.7096029664
33	H	-0.6053628323	-1.6566752435	-5.4964615251
34	H	-2.2247016887	-0.9650730836	-2.1433811306
35	H	-2.3505006254	0.7619987216	-1.7973899510
36	H	-2.3354356232	-2.0753347250	1.0214784636
37	H	1.6545568255	-2.1311094571	3.1451932922
38	H	0.0714826072	-2.8926587498	3.3166948380
39	H	0.6131698096	-1.7202230704	4.5125742547
40	H	-1.8917911471	-0.2229554738	2.4946230112
41	H	-0.5300302543	3.9446175333	1.3038534594
42	H	0.6277172053	3.5547906384	0.0377755984
43	H	1.1059431322	4.6109844471	1.3758813990

(7*R**,9*S**)-**S2D**, M0002:M0007

I	Atom	X	Y	Z
1	C	-0.3698305666	0.0737504809	-1.7512018881
2	C	0.0555139489	-1.3149489001	-1.2153243496
3	C	0.5325351166	-1.0389092817	0.2119205666
4	C	1.0928504114	0.3719100807	0.0895691103

5	O	0.1577931471	1.0291659752	-0.8004101977
6	C	1.2458401514	-1.7397152098	-2.0533242733
7	N	1.4431925102	-0.7915964080	-3.0074005831
8	C	0.4466112165	0.2521553209	-3.0825208953
9	C	1.1316974942	1.1290335311	1.4014460563
10	C	-0.2314277253	1.0960696743	2.0842363236
11	C	-0.7557807542	-0.3270549898	2.2436607232
12	O	-0.6618720447	-1.0478843233	1.0092809129
13	O	1.9394142035	-2.7135132150	-1.8626811470
14	C	-0.3799090495	0.0941092838	-4.3304055204
15	O	-0.4642659901	-0.8794060305	-5.0251118462
16	O	-1.0383058171	1.2499889336	-4.5935028864
17	C	-1.8786216246	0.2915476783	-1.9337485804
18	C	-2.6444605346	-0.0636758673	-0.6894019196
19	O	-3.1437652892	-1.1432104681	-0.4893416592
20	O	-2.7176807660	0.9588401441	0.1766077777
21	H	-0.7379740445	-2.0450981644	-1.2202715349
22	H	1.2544014840	-1.7560155791	0.5634565372
23	H	2.0819376732	0.3425414510	-0.3418669230
24	C	-0.1189681340	-1.1232763312	3.3706988397
25	O	-2.1748218996	-0.2183028063	2.4686498285
26	O	2.1642699743	0.4550847784	2.1459742594
27	C	2.7893416280	1.2127765191	3.1977846060
28	H	0.8621775161	1.2461967449	-3.0716219759
29	H	1.4146018060	2.1571165522	1.2087403249
30	H	2.1515724467	-0.9058781871	-3.7004969712
31	H	-0.2070135556	1.5794870838	3.0511720427
32	H	-0.9119753591	1.6234444180	1.4367279953
33	H	-1.5984105142	1.1906167482	-5.3819118238
34	H	-2.2311653990	-0.3628739325	-2.7160062002
35	H	-2.0499281054	1.3229288564	-2.1925872235
36	H	-2.8306020296	0.6524006752	1.1041342143
37	H	0.9495076783	-1.1534403593	3.2380128476
38	H	-0.5153639910	-2.1315411840	3.3673273750
39	H	-0.3547703434	-0.6532254353	4.3163941364
40	H	-2.5772111423	-1.0972576191	2.3803605346

41	H	2.0896843040	1.4511673163	3.9893544264
42	H	3.2122365263	2.1330453717	2.8100475030
43	H	3.5789454430	0.5934466736	3.5935814572

(7*R**,9*S**)-**S2D**, M0002:M0008

I	Atom	X	Y	Z
1	C	0.2788090558	-0.0446166582	-1.7416386508
2	C	-0.6232695264	1.1112142002	-1.2759876350
3	C	-1.0163726800	0.7712205422	0.1571830791
4	C	-0.9680917888	-0.7653662619	0.1913288477
5	O	-0.0653778789	-1.1503462751	-0.8903506268
6	C	-1.8663114888	0.9955901502	-2.1481487446
7	N	-1.5997538939	0.1078798005	-3.1382773216
8	C	-0.2512816843	-0.4080032483	-3.1661135606
9	C	-0.4203873606	-1.3099168543	1.5060539530
10	C	-0.7038910060	-0.3287827093	2.6465492813
11	C	-0.0065494954	1.0177066198	2.4221668234
12	O	-0.0146789240	1.3381364827	1.0092266028
13	O	-2.9210136581	1.5550562215	-1.9484280133
14	C	0.5343874552	0.2508105380	-4.2682763477
15	O	0.2652757187	1.2682154715	-4.8417582919
16	O	1.6445580248	-0.4799724917	-4.5405406624
17	C	1.8007979597	0.1813292756	-1.6972208275
18	C	2.2298677643	0.5355029182	-0.3061452043
19	O	2.4495126013	-0.2513084894	0.5902908851
20	O	2.3408204919	1.8628116760	-0.1508877510
21	H	-0.1693685111	2.0874131836	-1.3370185533
22	H	-1.9943012490	1.1615991513	0.3913735864
23	H	-1.9395954094	-1.1949469127	0.0107691278
24	C	-0.6441609100	2.1546754390	3.1947953110
25	O	1.3585948255	0.9904103611	2.7935917405
26	O	-1.0992486930	-2.5540408889	1.7480103019
27	C	-0.3700501288	-3.5258486908	2.5153164610
28	H	-0.2027457108	-1.4776110218	-3.2785627914
29	H	0.6375676606	-1.4668875433	1.3735453965

30	H	-2.2752234744	-0.0983750835	-3.8423330861
31	H	-1.7762963508	-0.1945738694	2.7192494030
32	H	-0.3521594314	-0.7249217075	3.5888382592
33	H	2.1885717870	-0.0856601338	-5.2388575504
34	H	2.0778341926	0.9906388403	-2.3512920743
35	H	2.2791896452	-0.7363146252	-1.9988782489
36	H	2.2938876619	2.1309393218	0.7816921458
37	H	-1.6762983298	2.2859895656	2.8975488405
38	H	-0.0939555820	3.0614670939	2.9886232354
39	H	-0.5967155336	1.9473135634	4.2552732611
40	H	1.8584711342	0.3498405573	2.2577813293
41	H	-0.1785811037	-3.1819476030	3.5256048253
42	H	0.5750654926	-3.7662853862	2.0415981607
43	H	-0.9875316682	-4.4100345195	2.5543050839

(7*R**,9*S**)-**S2D,** M0002:M0009

I	Atom	X	Y	Z
1	C	-0.3595454076	0.0705327559	-1.7213706294
2	C	0.5402098539	-1.0885139467	-1.2568153769
3	C	0.9593246828	-0.7425674440	0.1690770774
4	C	0.9130887087	0.7944981185	0.1970567370
5	O	-0.0004870521	1.1777157349	-0.8739737613
6	C	1.7739469103	-0.9921595145	-2.1448809927
7	N	1.5163974125	-0.0905355688	-3.1237923722
8	C	0.1764640293	0.4417452000	-3.1452405682
9	C	0.3824855136	1.3433907204	1.5161638032
10	C	0.7017556679	0.3747413017	2.6569714193
11	C	0.0067947642	-0.9772321505	2.4596580754
12	O	-0.0189531615	-1.3088098297	1.0483569054
13	O	2.8171628152	-1.5805129228	-1.9656531940
14	C	-0.6483419199	-0.0969187665	-4.2903651992
15	O	-1.7798909610	0.2450739183	-4.5208840016
16	O	0.0114930982	-0.9888456016	-5.0526937625
17	C	-1.8829474442	-0.1343799457	-1.6534194360
18	C	-2.2800557819	-0.5205308359	-0.2619699088

19	O	-2.4990386082	0.2405270763	0.6562210471
20	O	-2.3585080288	-1.8546167486	-0.1336200031
21	H	0.0765598576	-2.0609883435	-1.3008877628
22	H	1.9427519532	-1.1309873759	0.3831190134
23	H	1.8836361407	1.2212944510	0.0042605957
24	C	0.6711172261	-2.1045532372	3.2241152515
25	O	-1.3472404017	-0.9531787195	2.8656563091
26	O	1.0476830400	2.5991231740	1.7339671750
27	C	0.3166371418	3.5690102993	2.5019852421
28	H	0.1548499340	1.5194658856	-3.2048185092
29	H	-0.6802327498	1.4837052708	1.4034878794
30	H	2.1705865685	0.0723229894	-3.8580034957
31	H	1.7766082017	0.2486109793	2.7052151074
32	H	0.3690283249	0.7754167331	3.6041411951
33	H	-0.5303036496	-1.3050846209	-5.7915199921
34	H	-2.1959497393	-0.9110988654	-2.3271588290
35	H	-2.3529975649	0.7993678320	-1.9165141973
36	H	-2.2989673513	-2.1402454744	0.7924707763
37	H	1.6965809722	-2.2315838020	2.9026704117
38	H	0.1215707282	-3.0161713573	3.0384256099
39	H	0.6483884232	-1.8897015180	4.2839498293
40	H	-1.8703590217	-0.3438538414	2.3155213257
41	H	0.1447360517	3.2330595007	3.5184665162
42	H	-0.6385103928	3.7915960235	2.0398661414
43	H	0.9224712156	4.4618724659	2.5227585479

(7*R**,9*S**)-**S2D**, M0002:M0010

I	Atom	X	Y	Z
1	C	-0.4879154757	0.1616039669	-1.6251654027
2	C	-0.1671280015	-1.2645752470	-1.1148214509
3	C	0.3753968919	-1.0483243926	0.2995928157
4	C	1.0534221731	0.3094221269	0.1680675981
5	O	0.1529727085	1.0573334154	-0.6843015536
6	C	0.9593716721	-1.7764327013	-1.9914922020
7	N	1.2061470338	-0.8386612796	-2.9445649582

8	C	0.2962175644	0.2837129837	-2.9813869282
9	C	1.2020947250	1.0430285344	1.4883196244
10	C	-0.1306560267	1.1150430553	2.2206094429
11	C	-0.7731960235	-0.2582076592	2.3863285328
12	O	-0.7829859027	-0.9703308109	1.1413005265
13	O	1.5795023652	-2.8033986522	-1.8268066304
14	C	-0.5787548626	0.2022598724	-4.2034740107
15	O	-0.7664902719	-0.7580056739	-4.8965152890
16	O	-1.1427408174	1.4111324957	-4.4453842215
17	C	-1.9769483142	0.5114584842	-1.7546924358
18	C	-2.7285069471	0.2039371578	-0.4888570966
19	O	-3.3098043531	-0.8334623529	-0.2862950628
20	O	-2.6824034004	1.2141638249	0.3919958241
21	H	-1.0194415135	-1.9251638236	-1.1043270627
22	H	1.0460877236	-1.8294976839	0.6152742363
23	H	2.0124513122	0.1890886527	-0.3164857670
24	C	-0.1793680456	-1.1167957198	3.4908823935
25	O	-2.1701624862	-0.0247391424	2.6551116435
26	O	2.1461660621	0.3021279447	2.2853381073
27	C	3.5321866302	0.6709828269	2.1631231001
28	H	0.7934605421	1.2396856742	-2.9795665442
29	H	1.5629243689	2.0476713931	1.3037579983
30	H	1.8783953579	-1.0070284954	-3.6624017821
31	H	0.0015045476	1.5705413297	3.1913280379
32	H	-0.7912394010	1.7142032910	1.6166612799
33	H	-1.7299220015	1.4025240000	-5.2161919823
34	H	-2.4110082269	-0.0980722919	-2.5322021773
35	H	-2.0672885880	1.5575735471	-1.9948709422
36	H	-2.7801498158	0.9022882024	1.3204226257
37	H	0.8860918058	-1.2028654773	3.3607000216
38	H	-0.6371460343	-2.0987541413	3.4668799234
39	H	-0.3858111290	-0.6513995393	4.4455015581
40	H	-2.6486718327	-0.8670347289	2.5972452328
41	H	3.6752512952	1.7197307749	2.3977347481
42	H	3.9156836753	0.4762585752	1.1684340710
43	H	4.0724110158	0.0669776839	2.8751941583

(7*R**,9*S**)-**S2D,** M0002:M0011

I	Atom	X	Y	Z
1	C	-0.4088146972	0.1353878612	-1.7352362589
2	C	0.0271699736	-1.2543864198	-1.2096365096
3	C	0.5097466041	-0.9871181059	0.2180974026
4	C	1.0545179917	0.4305070982	0.1064434534
5	O	0.1079944616	1.0860408778	-0.7719579601
6	C	1.2153174280	-1.6690995502	-2.0554326883
7	N	1.4202118866	-0.7037123289	-2.9899822169
8	C	0.4236654166	0.3379684951	-3.0572377728
9	C	1.0923962777	1.1748838351	1.4255697163
10	C	-0.2679604301	1.1231768353	2.1124672823
11	C	-0.7776137002	-0.3062375435	2.2632758828
12	O	-0.6773559029	-1.0199795591	1.0251890510
13	O	1.9005690745	-2.6532399756	-1.8861693830
14	C	-0.3852966747	0.2938786564	-4.3320027884
15	O	-1.1867224245	1.1343905571	-4.6469757421
16	O	-0.1077081739	-0.7752968609	-5.1042301126
17	C	-1.9185824652	0.3481769626	-1.9126857784
18	C	-2.6743839514	-0.0254166183	-0.6676122920
19	O	-3.1596873729	-1.1132879368	-0.4751267205
20	O	-2.7557847932	0.9864000913	0.2094211652
21	H	-0.7630209493	-1.9880949353	-1.2142268258
22	H	1.2425669227	-1.6997573141	0.5559648718
23	H	2.0417217026	0.4149946257	-0.3302048092
24	C	-0.1323298311	-1.1021142767	3.3857226058
25	O	-2.1979025632	-0.2144918756	2.4897539017
26	O	2.1342685520	0.5032289042	2.1591446496
27	C	2.7593093438	1.2591930379	3.2122107683
28	H	0.8456328766	1.3295812211	-2.9903051195
29	H	1.3652223038	2.2073726776	1.2420585039
30	H	2.1042814527	-0.8275253877	-3.7047575231
31	H	-0.2458768749	1.5998911826	3.0827994933
32	H	-0.9553254587	1.6482488420	1.4703070812

33	H	-0.6285428680	-0.7786204101	-5.9215631549
34	H	-2.2761150464	-0.2913228032	-2.7030892071
35	H	-2.0975796977	1.3812435594	-2.1613700550
36	H	-2.8722283634	0.6681453461	1.1323771299
37	H	0.9361069356	-1.1237654022	3.2503795974
38	H	-0.5214000081	-2.1131770101	3.3792165260
39	H	-0.3698537398	-0.6376620998	4.3337225182
40	H	-2.5917147159	-1.0948398355	2.3792573926
41	H	2.0631089627	1.4848213293	4.0105213690
42	H	3.1699709691	2.1865286010	2.8281261941
43	H	3.5580215665	0.6450856521	3.5977763610

(7*R**,9*S**)-**S2D**, M0002:M0012

I	Atom	X	Y	Z
1	C	-0.5109140334	0.2297867139	-1.6116486406
2	C	-0.1871284403	-1.2002150371	-1.1129002454
3	C	0.3529768058	-0.9969022568	0.3051294314
4	C	1.0230791129	0.3663097605	0.1903433762
5	O	0.1203733446	1.1179693739	-0.6554989753
6	C	0.9418257248	-1.7026634890	-1.9916387697
7	N	1.2074791582	-0.7457101089	-2.9202306292
8	C	0.2954163088	0.3727791691	-2.9566018789
9	C	1.1648455992	1.0861967478	1.5188452123
10	C	-0.1691299372	1.1407751342	2.2501983925
11	C	-0.8025938875	-0.2383146628	2.4003887880
12	O	-0.8047248403	-0.9392488861	1.1490110967
13	O	1.5481204453	-2.7412532375	-1.8473366471
14	C	-0.5538456324	0.3951572478	-4.2054725703
15	O	-1.2793426393	1.3068135998	-4.5063672735
16	O	-0.4114637235	-0.7067326764	-4.9686144960
17	C	-1.9990600351	0.5826770217	-1.7402128979
18	C	-2.7500866997	0.2522311684	-0.4797050823
19	O	-3.3236099175	-0.7933450086	-0.2945610092
20	O	-2.7113537106	1.2464118719	0.4189651132
21	H	-1.0383650460	-1.8622852478	-1.1086326446

22	H	1.0293773560	-1.7774061993	0.6104849335
23	H	1.9844003678	0.2567252996	-0.2924617593
24	C	-0.2045237796	-1.1027889260	3.4981749176
25	O	-2.2016749143	-0.0173417565	2.6682801457
26	O	2.1151485363	0.3434759726	2.3071353056
27	C	3.4963239486	0.7327462275	2.1958385174
28	H	0.7980365126	1.3267556263	-2.9027837559
29	H	1.5178915332	2.0955796014	1.3455077273
30	H	1.8588018224	-0.9211788547	-3.6548306401
31	H	-0.0421918782	1.5868327085	3.2260788288
32	H	-0.8327500821	1.7421720564	1.6517138294
33	H	-0.9593441937	-0.6691806681	-5.7673454933
34	H	-2.4370189934	-0.0038178421	-2.5311423445
35	H	-2.0894578702	1.6332986290	-1.9629308248
36	H	-2.8147802983	0.9184494803	1.3409427875
37	H	0.8614568015	-1.1826977613	3.3672514324
38	H	-0.6575020055	-2.0867949365	3.4666646396
39	H	-0.4132152488	-0.6459756655	4.4564674504
40	H	-2.6738528440	-0.8621142352	2.5972695785
41	H	3.6262157551	1.7767162370	2.4578369742
42	H	3.8821205283	0.5690127375	1.1962699397
43	H	4.0440409898	0.1170950711	2.8921181602

Summary of ^{13}C chemical shift calculations (*δ* ^{13}C: *ω*B97X-D/6-31G*, Boltzmann distribution; *ω*B97X-V/6-311+G(2df,2p)[6-311G*])

(7*S,9*R**)-S2A, M0003**

	Energy (kJ/mol)	Relative Energy (kJ/mol)	Boltzmann Weights	*a*	*b*	*c*	*d*	*e*	*f*	*g*	*h*	*i*	*j*	*k*	*l*	*m*	*n*
M0003: M0001	-3351089.57	0	25.4%	89.20	55.83	76.90	76.57	171.66	64.08	72.04	35.26	98.54	173.62	38.93	174.34	30.01	54.33
M0003: M0002	-3351089.1	0.47	21.0%	89.27	54.36	76.70	75.76	172.88	66.27	72.12	35.45	98.31	173.02	39.01	175.00	29.99	54.39
M0003: M0003	-3351087.05	2.53	9.1%	88.58	54.91	76.81	75.56	173.36	66.45	72.10	35.52	98.28	170.67	39.89	174.63	30.00	54.40
M0003: M0004	-3351085.47	4.1	4.8%	89.02	54.53	77.38	78.94	172.48	66.05	73.42	31.55	98.38	173.26	39.20	175.09	30.32	55.82
M0003: M0005	-3351087.26	2.32	9.9%	90.57	55.82	74.91	82.86	171.27	62.42	72.63	36.18	96.76	173.26	37.48	175.41	30.93	60.51
M0003: M0006	-3351085.18	4.4	4.3%	89.27	55.60	77.64	79.86	171.57	63.89	73.63	31.25	98.79	173.91	38.97	174.26	30.33	55.96
M0003: M0007	-3351084.49	5.09	3.2%	91.56	52.64	74.82	82.48	172.34	64.31	72.81	36.12	96.62	173.27	36.35	175.07	30.91	60.11
M0003: M0008	-3351083.7	5.88	2.4%	89.18	55.70	77.01	76.64	172.04	64.42	72.21	34.70	99.05	174.28	39.94	170.05	29.88	54.27
M0003: M0009	-3351083.27	6.3	2.0%	88.48	54.79	77.51	78.86	172.77	66.23	73.44	31.57	98.34	170.71	40.02	174.58	30.34	55.91
M0003: M0010	-3351085.35	4.22	4.6%	88.78	54.58	77.08	76.38	174.03	68.25	72.37	33.25	99.35	173.56	40.74	170.54	28.96	54.39
M0003: M0011	-3351084.07	5.51	2.7%	90.11	54.68	78.33	83.91	171.35	64.06	68.18	31.94	98.89	173.96	38.99	174.34	30.42	52.22
M0003: M0012	-3351083.33	6.25	2.0%	89.96	53.52	78.13	83.02	172.21	66.29	68.19	32.00	98.54	173.09	39.30	174.88	30.39	52.27
M0003: M0013	-3351081.6	7.97	1.0%	89.22	55.66	77.03	76.48	172.35	66.03	72.03	35.15	98.57	170.02	39.78	172.16	29.99	54.38
M0003: M0014	-3351083.56	6.01	2.2%	88.50	54.42	76.98	76.55	174.09	69.28	72.36	33.17	99.43	172.45	40.44	170.61	28.91	54.36
M0003: M0015	-3351083.11	6.47	1.9%	88.68	54.40	77.76	79.72	173.59	67.84	73.43	29.65	99.37	173.58	40.66	170.77	29.11	55.76
M0003: M0016	-3351081.1	8.47	0.8%	88.46	56.09	77.04	75.49	172.91	66.86	72.11	35.36	98.46	171.74	41.53	169.58	29.90	54.48
M0003: M0017	-3351080.69	8.89	0.7%	88.97	55.38	77.66	80.03	172.00	64.35	73.62	30.78	99.05	174.50	40.05	170.46	30.18	55.72
M0003: M0018	-3351080.49	9.08	0.6%	88.48	55.82	76.91	76.69	172.57	66.86	72.32	34.51	99.02	171.14	41.08	169.84	29.80	54.31
M0003: M0019	-3351081.95	7.62	1.2%	88.37	52.86	77.05	76.37	173.82	65.58	72.15	35.50	98.32	173.08	41.58	171.56	30.00	54.43
	**Boltsmann averaged**			**89.32**	**55.00**	**76.75**	**77.82**	**172.37**	**65.24**	**72.19**	**34.57**	**98.32**	**173.03**	**39.11**	**174.11**	**30.09**	**55.27**

(7*R,9*R**)-S2B, M0004**

	Energy (kJ/mol)	Relative Energy (kJ/mol)	Boltzmann Weights	*a*	*b*	*c*	*d*	*e*	*f*	*g*	*h*	*i*	*j*	*k*	*l*	*m*	*n*
M0004: M0001	-3351096.98	1.4	9.8%	90.02	55.95	73.08	74.52	171.62	63.64	76.11	34.59	96.98	173.40	38.98	174.35	28.87	56.90
M0004: M0002	-3351096.43	1.95	7.9%	90.06	54.41	72.71	73.84	172.92	65.86	75.84	34.91	97.01	172.97	39.17	174.89	28.73	56.59
M0004: M0003	-3351097.55	0.84	12.3%	89.76	54.77	73.06	78.22	172.40	65.84	76.70	29.15	97.31	173.12	39.39	174.81	28.51	56.05
M0004: M0004	-3351098.38	0	17.3%	89.59	56.26	73.45	78.88	171.45	63.76	77.06	28.80	97.45	173.49	39.10	174.33	28.48	56.04
M0004: M0005	-3351098.23	0.15	16.3%	89.67	55.98	73.56	78.81	171.25	63.64	77.20	28.78	97.64	173.30	38.92	174.27	28.39	56.31
M0004: M0006	-3351093.93	4.46	2.9%	89.54	54.76	72.85	73.69	173.34	65.87	76.24	35.03	96.96	170.57	40.05	174.32	28.73	56.93
M0004: M0007	-3351094.76	3.63	4.0%	89.07	55.05	73.12	78.04	173.07	66.01	76.45	29.45	97.14	170.59	39.98	174.34	28.66	55.97
M0004: M0008	-3351096.49	1.9	8.0%	89.58	54.64	73.65	79.15	173.56	67.67	76.66	27.33	98.22	173.50	40.81	170.85	27.47	55.79
M0004: M0009	-3351094.33	4.06	3.4%	89.79	55.68	73.45	79.07	171.91	63.84	77.17	28.26	97.73	174.02	39.81	170.83	28.35	56.22
M0004: M0010	-3351094.74	3.64	4.0%	89.82	54.66	73.37	74.78	174.14	67.68	76.32	33.00	97.81	173.44	40.73	170.66	27.73	56.82
M0004: M0011	-3351092.46	5.93	1.6%	90.05	55.64	73.04	74.45	172.03	63.84	76.56	34.18	97.30	174.03	39.60	170.73	28.74	56.83
M0004: M0012	-3351095.38	3	5.2%	89.19	54.67	73.51	79.15	173.57	68.81	76.59	27.08	98.21	172.50	40.47	170.89	27.53	55.71
M0004: M0013	-3351093.68	4.7	2.6%	89.44	54.63	73.34	74.91	173.97	68.90	76.35	32.92	97.94	172.36	40.47	170.65	27.71	56.84
M0004: M0014	-3351091.55	6.84	1.1%	89.18	55.99	73.37	79.12	172.21	66.39	76.96	28.17	97.88	170.98	40.70	170.72	28.38	55.94
M0004: M0015	-3351089.79	8.59	0.5%	89.40	55.79	72.99	74.53	172.34	66.39	76.43	34.06	97.35	170.90	40.57	170.50	28.67	56.81
M0004: M0016	-3351089.4	8.98	0.5%	89.53	55.86	72.88	74.27	172.55	65.58	76.25	34.63	97.05	170.38	39.68	171.79	28.81	56.88
M0004: M0017	-3351090.84	7.55	0.8%	89.48	56.05	73.40	78.83	172.00	65.77	76.86	28.67	97.52	170.08	39.93	172.58	28.51	56.11
M0004: M0018	-3351089.61	8.78	0.5%	90.19	55.72	72.99	74.19	172.21	65.61	76.39	34.48	97.01	169.87	39.70	172.61	28.85	56.87
M0004: M0019	-3351090.14	8.24	0.6%	89.00	56.02	73.15	78.72	172.36	65.70	76.39	29.09	97.36	170.39	39.86	171.85	28.68	55.97
M0004: M0020	-3351089.45	8.93	0.5%	88.92	56.49	73.35	77.99	172.41	66.43	76.53	29.19	97.53	171.63	41.71	169.32	28.48	55.93
M0004: M0021	-3351088.91	9.47	0.4%	89.22	53.04	73.22	74.32	173.73	65.27	76.21	34.99	97.04	172.79	41.78	171.31	28.69	56.97
	**Boltsmann averaged**			**89.67**	**55.39**	**73.29**	**77.40**	**172.31**	**65.27**	**76.67**	**30.33**	**97.49**	**172.98**	**39.57**	**173.41**	**28.37**	**56.28**

(7*S,9*S**)-S2C, M0001**

	Energy (kJ/mol)	Relative Energy (kJ/mol)	Boltzmann Weights	*a*	*b*	*c*	*d*	*e*	*f*	*g*	*h*	*i*	*j*	*k*	*l*	*m*	*n*
M0001: M0001	-3351088.85	0	31.3%	91.24	55.72	76.74	83.04	171.18	62.55	73.66	36.64	98.73	173.07	37.85	175.39	24.76	60.76
M0001: M0002	-3351085.44	3.41	7.9%	92.13	52.95	76.68	82.78	172.26	64.48	73.98	36.68	98.70	173.10	36.99	174.71	24.62	60.26
M0001: M0003	-3351087.76	1.09	20.2%	89.43	54.89	77.58	76.15	173.95	67.80	74.98	36.34	100.22	173.48	41.23	174.59	22.28	54.59
M0001: M0004	-3351086.31	2.54	11.2%	89.06	54.93	77.46	76.21	173.65	68.97	74.84	36.26	100.25	172.28	40.89	174.56	22.30	54.52
M0001: M0005	-3351085.74	3.11	8.9%	89.20	54.70	77.85	79.89	173.45	67.54	75.94	32.95	100.13	173.53	41.18	174.81	22.39	55.68
M0001: M0006	-3351082.46	6.39	2.4%	91.41	53.25	76.84	82.74	172.59	64.50	74.04	36.64	98.74	170.38	37.88	174.47	24.58	60.16
M0001: M0007	-3351081.71	7.14	1.8%	91.80	55.58	76.60	83.29	171.80	63.05	73.68	36.53	98.80	173.54	38.87	171.45	24.85	62.12
M0001: M0008	-3351081.72	7.13	1.8%	90.84	55.53	76.49	82.79	172.22	64.03	73.70	36.50	98.88	171.04	37.76	172.83	24.89	60.37
M0001: M0009	-3351084.23	4.62	4.9%	88.91	54.70	77.75	79.87	173.12	68.69	75.87	32.82	100.13	172.53	40.88	174.82	22.40	55.68
M0001: M0010	-3351084.79	4.06	6.1%	90.00	53.87	78.46	83.65	173.18	67.70	71.04	33.46	100.82	173.30	41.29	174.83	22.01	52.22
M0001: M0011	-3351083.45	5.4	3.5%	89.76	53.78	78.38	83.62	173.03	68.84	71.09	33.31	100.95	172.32	41.15	174.94	21.98	52.20
	**Boltsmann averaged**			**90.28**	**54.86**	**77.29**	**80.47**	**172.65**	**65.84**	**74.15**	**35.71**	**99.60**	**172.98**	**39.59**	**174.81**	**23.38**	**57.25**

(7*R,9*S**)-S2D, M0002**

	Energy (kJ/mol)	Relative Energy (kJ/mol)	Boltzmann Weights	*a*	*b*	*c*	*d*	*e*	*f*	*g*	*h*	*i*	*j*	*k*	*l*	*m*	*n*
M0002: M0001	-3351095.03	0	38.2%	89.75	55.01	76.79	79.62	173.58	67.52	74.93	30.00	97.85	173.54	41.19	174.94	23.19	55.48
M0002: M0002	-3351093.95	1.08	24.7%	89.42	55.14	76.76	79.71	173.11	68.81	74.95	29.92	97.94	172.50	41.22	175.15	23.14	55.46
M0002: M0003	-3351091.48	3.55	9.1%	90.04	55.19	76.41	75.27	173.77	67.59	74.96	35.67	98.53	173.48	41.23	174.76	23.97	56.90
M0002: M0004	-3351090.4	4.63	5.9%	89.72	55.13	76.42	75.44	173.56	68.77	75.00	35.58	98.63	172.48	41.01	174.85	23.83	57.01
M0002: M0005	-3351091.04	3.98	7.7%	92.13	54.87	77.88	89.40	173.30	66.46	76.02	38.38	99.90	173.60	41.19	175.67	27.22	56.36
M0002: M0006	-3351090.22	4.81	5.5%	91.75	55.26	77.77	89.34	173.14	67.77	75.90	38.38	99.89	172.40	41.21	175.74	27.24	56.59
M0002: M0007	-3351088.84	6.18	3.2%	89.95	54.64	76.98	79.01	173.20	68.08	74.57	26.67	98.25	173.31	41.88	171.49	26.09	55.62
M0002: M0008	-3351085.93	9.1	1.0%	91.76	54.96	77.60	88.89	173.10	66.40	77.31	35.12	99.97	173.59	41.31	176.00	27.39	55.91
M0002: M0009	-3351085.04	9.99	0.7%	91.55	55.25	77.56	88.88	172.82	67.71	77.33	35.18	99.97	172.60	41.22	176.15	27.33	56.04
M0002: M0010	-3351086.47	8.56	1.2%	90.18	54.67	76.60	74.53	173.72	68.24	74.57	32.74	98.58	173.38	42.08	171.50	26.81	57.22
M0002: M0011	-3351087.76	7.26	2.0%	89.67	54.48	76.91	79.03	173.16	69.16	74.55	26.57	98.20	172.34	41.61	171.74	26.08	55.56
M0002: M0012	-3351085.34	9.69	0.8%	90.10	54.41	76.66	74.74	173.69	69.14	74.42	32.62	98.77	172.26	41.81	171.71	26.85	57.14
**Boltsmann averaged**				**90.03**	**55.04**	**76.88**	**80.31**	**173.41**	**67.91**	**75.09**	**31.89**	**98.32**	**173.11**	**41.24**	**174.85**	**24.11**	**55.87**

[MOUSE BEHAVIORAL ASSAY]

The mouse behavioral assay was performed under approval by the Ethical Committee of Experimental Animal Care at Hokkaido University (14-0081).

[REFERENCES AND NOTES]

[1] a) N. Grimblat, A. M. Sarotti, *Chem. Eur. J.* **2016**, *22*, 12246-12261; b) K. Morokuma, R. Irie, M. Oikawa, *Tetrahedron Lett.* **2019**, *60*, 2067-2069; c) M. Oikawa, Y. Sugeno, H. Tukada, Y. Takasaki, S. Takamizawa, R. Irie, *Bull. Chem. Soc. Jpn.* **2019**, *92*, 1816-1823; d) K. Tanaka, H. Manabe, R. Irie, M. Oikawa, *Bull. Chem. Soc. Jpn.* **2019**, *92*, 1314-1323.

[2] *Tutorial and User's Guide, Spartan '18 for Windows, Macintosh and Linux*, Wavefunction Inc., **2018**.

[3] T. A. Halgren, *J. Comp. Chem.* **1996**, *17*, 490-519.

[4] W. Hehre, P. Klunzinger, B. Deppmeier, A. Driessen, N. Uchida, M. Hashimoto, E. Fukushi, Y. Takata, *J. Nat. Prod.* **2019**, *82*, 2299-2306.

[5] S. G. Smith, J. M. Goodman, *J. Am. Chem. Soc.* **2010**, *132*, 12946-12959.

[NMR SPECTRA OF ALL NEW COMPOUNDS]

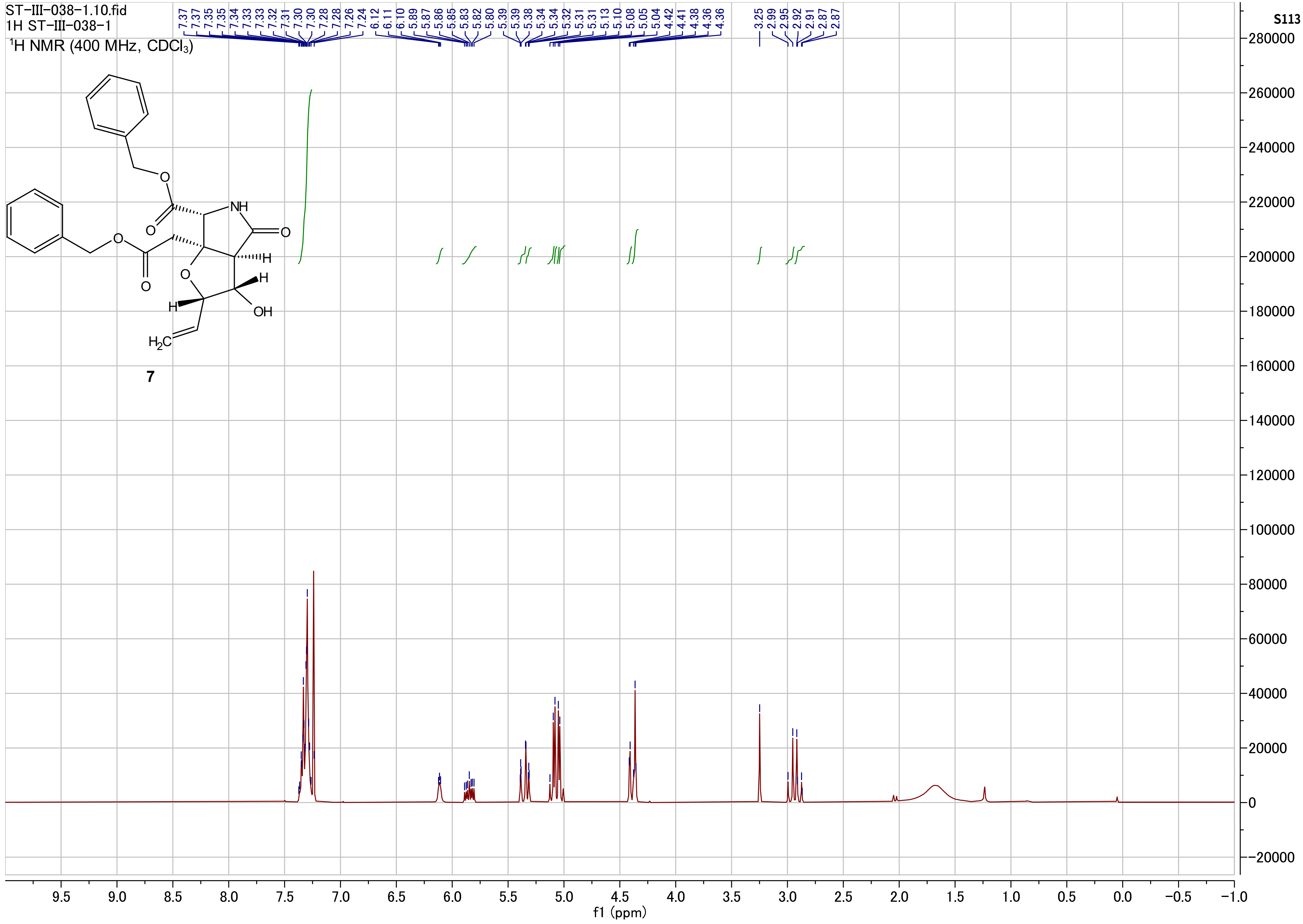
ST-III-038-1.10.fid
1H ST-III-038-1
^{1}H NMR (400 MHz, CDCl$_3$)
7.37 7.37 7.35 7.35 7.34 7.33 7.33 7.32 7.31 7.30 7.30 7.28 7.28 7.26 7.24 6.12 6.11 6.10 5.89 5.87 5.86 5.85 5.83 5.82 5.80 5.39 5.39 5.38 5.34 5.34 5.32 5.31 5.31 5.13 5.10 5.08 5.05 5.04 4.42 4.41 4.38 4.36 4.36
3.25 2.99 2.95 2.92 2.91 2.87 2.87
NH
O
OH
H
H_2C
7
f1 (ppm)

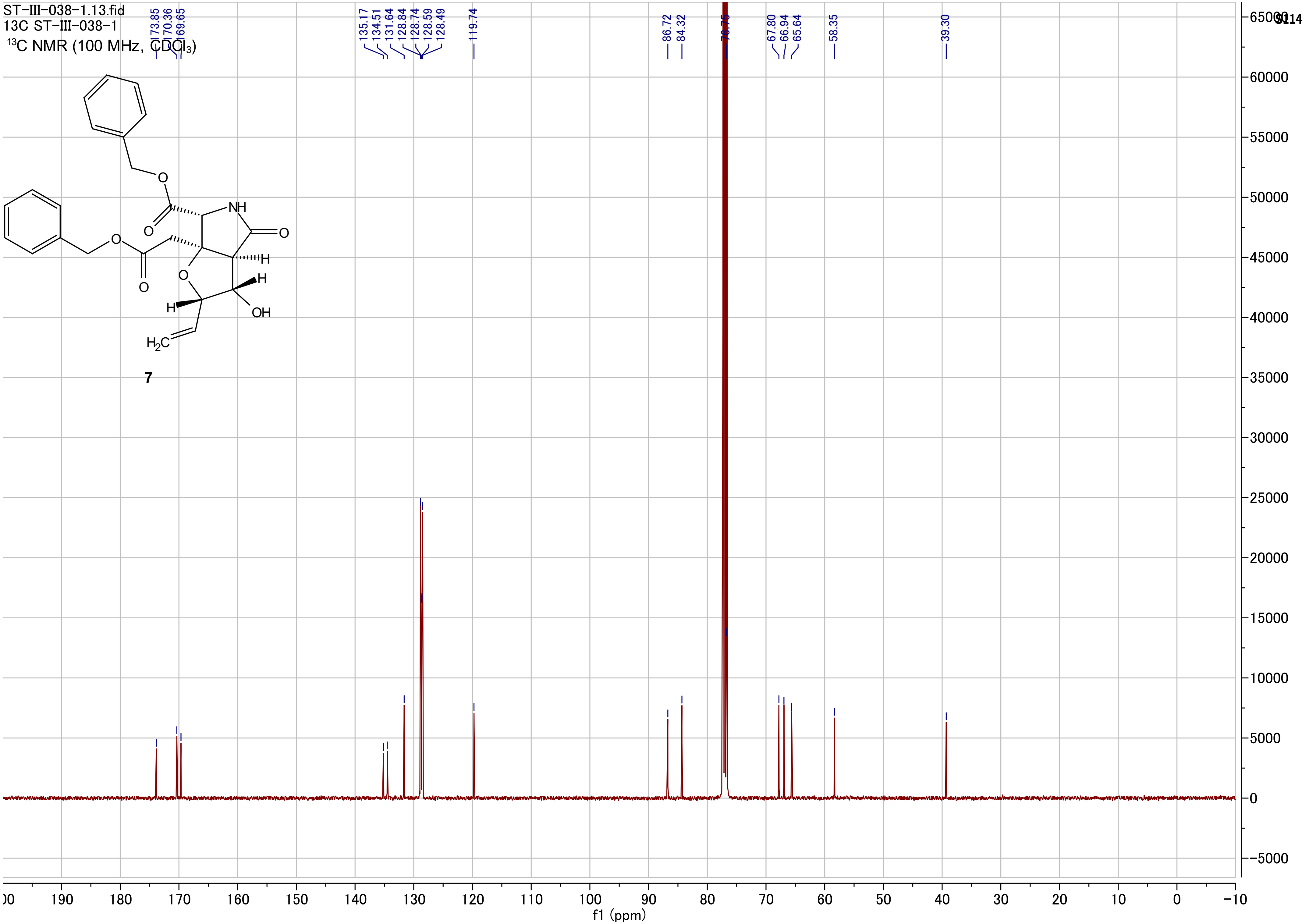
ST-III-038-1.13.fid
13C ST-III-038-1
^{13}C NMR (100 MHz, $CDCl_3$)
173.85
170.36
169.65
135.17
134.51
131.64
128.84
128.74
128.59
128.49
119.74
86.72
84.32
67.80
66.94
65.64
58.35
39.30
NH
O
OH
H
H_2C
7
f1 (ppm)

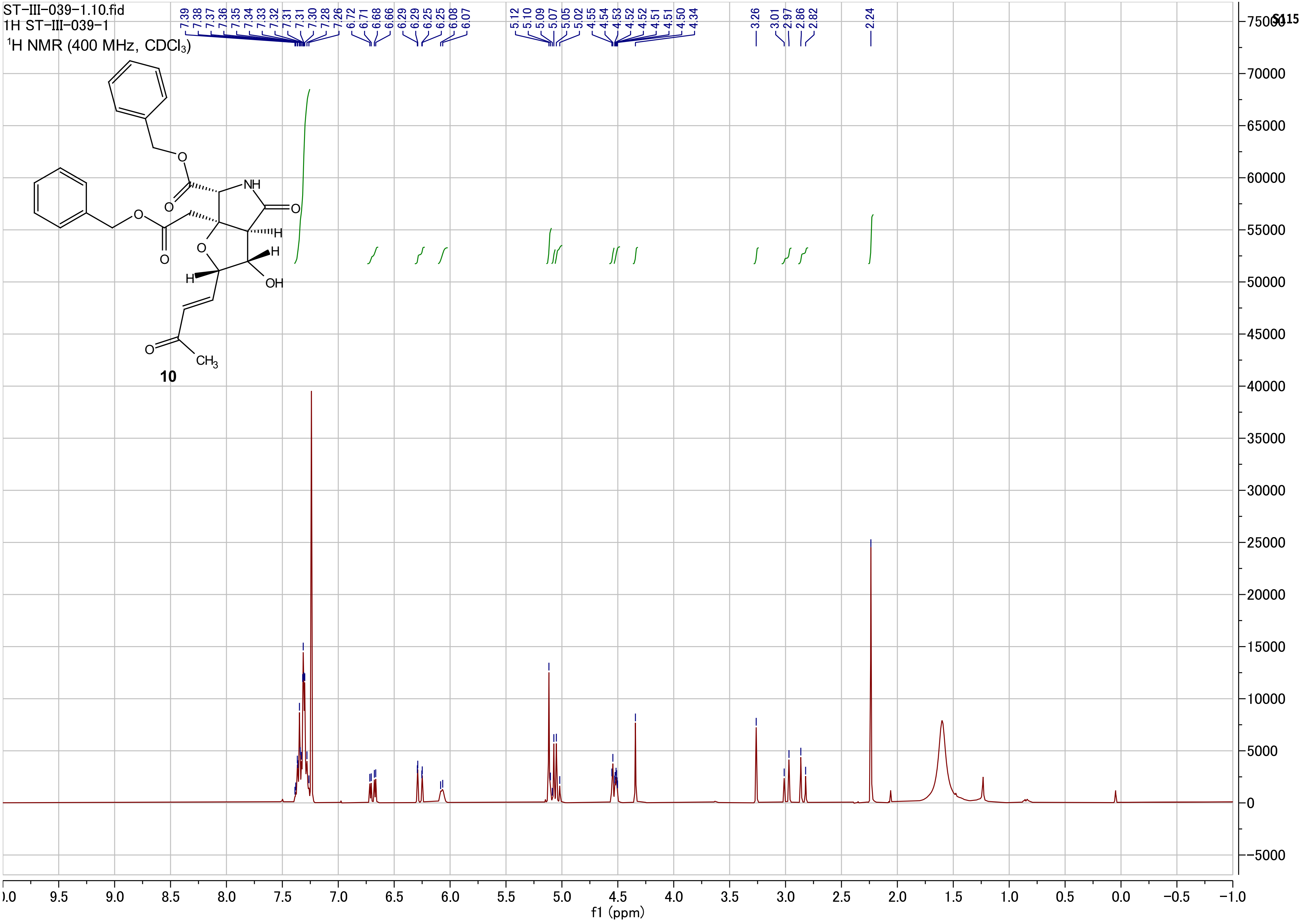
ST-III-039-1.10.fid
1H ST-III-039-1
^{1}H NMR (400 MHz, $CDCl_3$)
7.39 7.38 7.37 7.36 7.35 7.34 7.33 7.32 7.31 7.31 7.30 7.28 7.26 6.72 6.71 6.68 6.66 6.29 6.29 6.25 6.25 6.08 6.07
5.12 5.10 5.09 5.07 5.05 5.02 4.55 4.54 4.53 4.52 4.52 4.51 4.51 4.50 4.34
3.26 3.01 2.97 2.86 2.82
2.24
NH
O
H
OH
CH3
10
f1 (ppm)

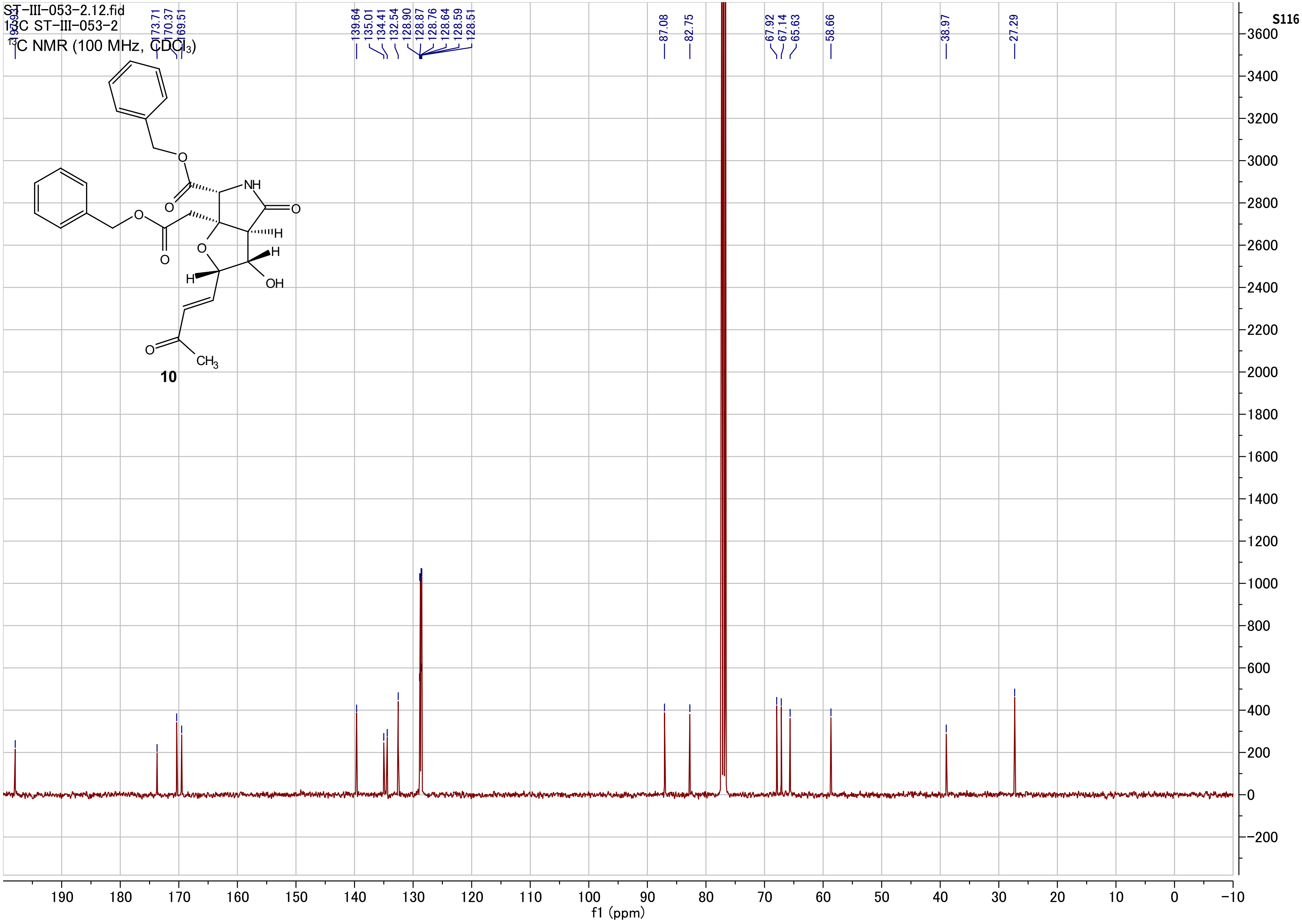
ST-III-053-2.12.fid
13C ST-III-053-2
^{13}C NMR (100 MHz, $CDCl_3$)
173.71
170.37
169.51
139.64
135.01
134.41
132.54
128.90
128.87
128.76
128.64
128.59
128.51
87.08
82.75
67.92
67.14
65.63
58.66
38.97
27.29
NH
O
OH
H
CH_3
10
f1 (ppm)

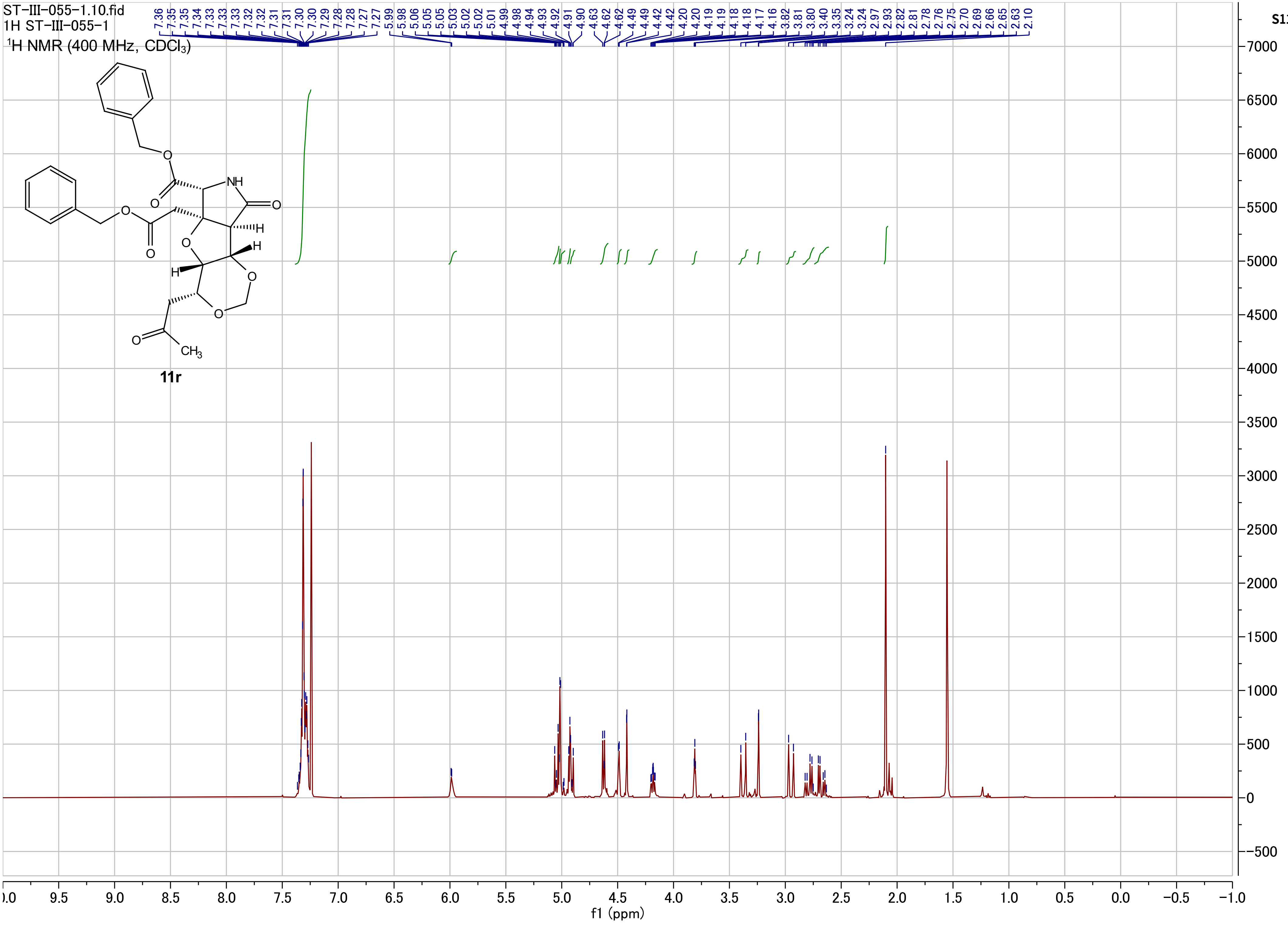

ST-III-055-1.10.fid
1H ST-III-055-1
1H NMR (400 MHz, CDCl3)
7.36 7.35 7.35 7.34 7.33 7.33 7.33 7.32 7.32 7.31 7.31 7.30 7.30 7.29 7.28 7.28 7.27 7.27 5.99 5.98 5.06 5.05 5.05 5.03 5.02 5.02 5.01 4.99 4.98 4.94 4.93 4.92 4.91 4.90 4.63 4.62 4.62 4.49 4.49 4.42 4.42 4.20 4.20 4.19 4.19 4.18 4.18 4.17 4.16 3.82 3.81 3.80 3.40 3.35 3.24 3.24 2.97 2.93 2.82 2.81 2.78 2.76 2.75 2.70 2.69 2.66 2.65 2.63 2.10
NH
O
H
CH3
11r
f1 (ppm)

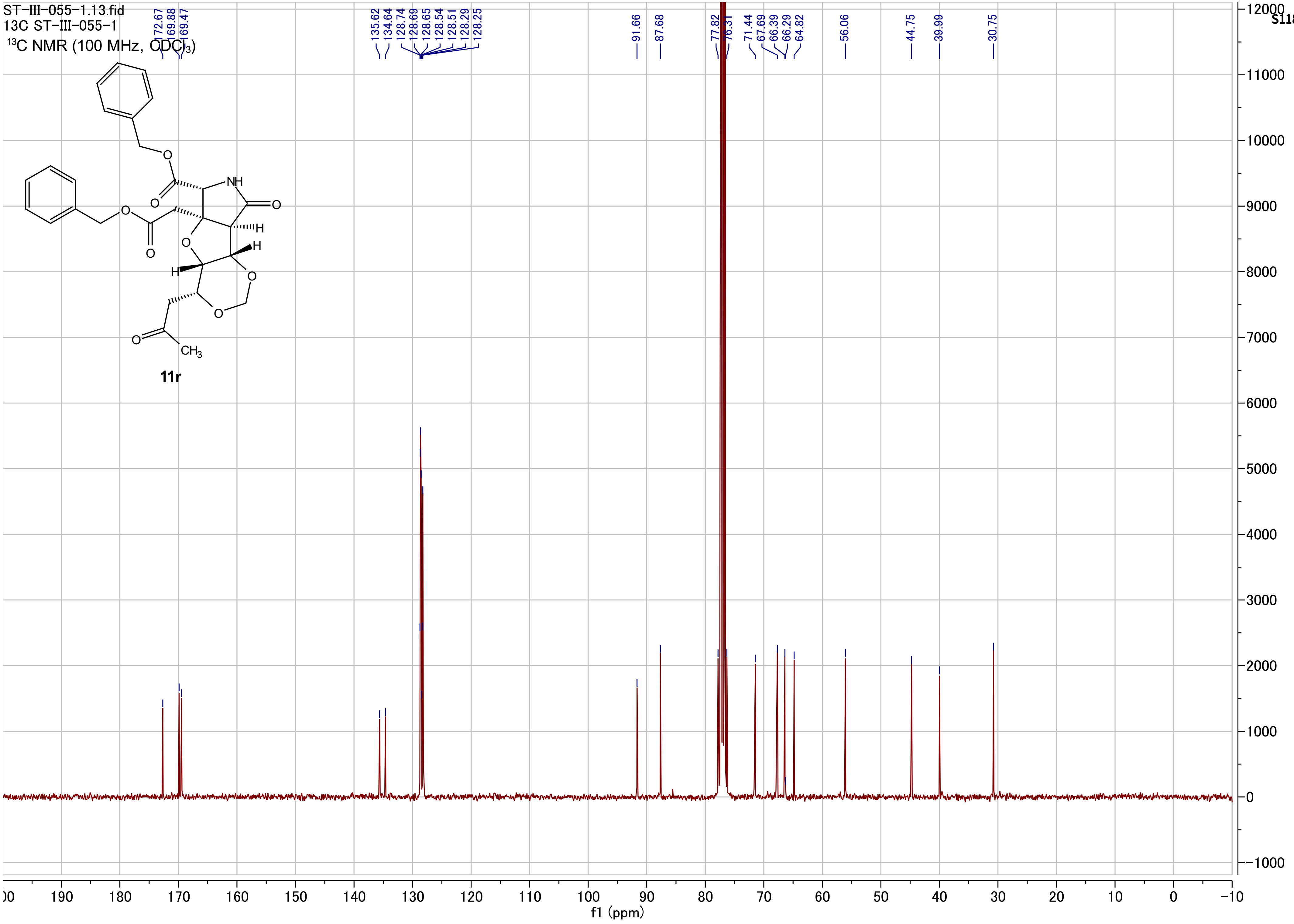

ST-III-055-1.13.fid
13C ST-III-055-1
^{13}C NMR (100 MHz, $CDCl_3$)
172.67
169.88
169.47
135.62
134.64
128.74
128.69
128.65
128.54
128.51
128.29
128.25
91.66
87.68
77.82
76.31
71.44
67.69
66.39
66.29
64.82
56.06
44.75
39.99
30.75
11r
f1 (ppm)

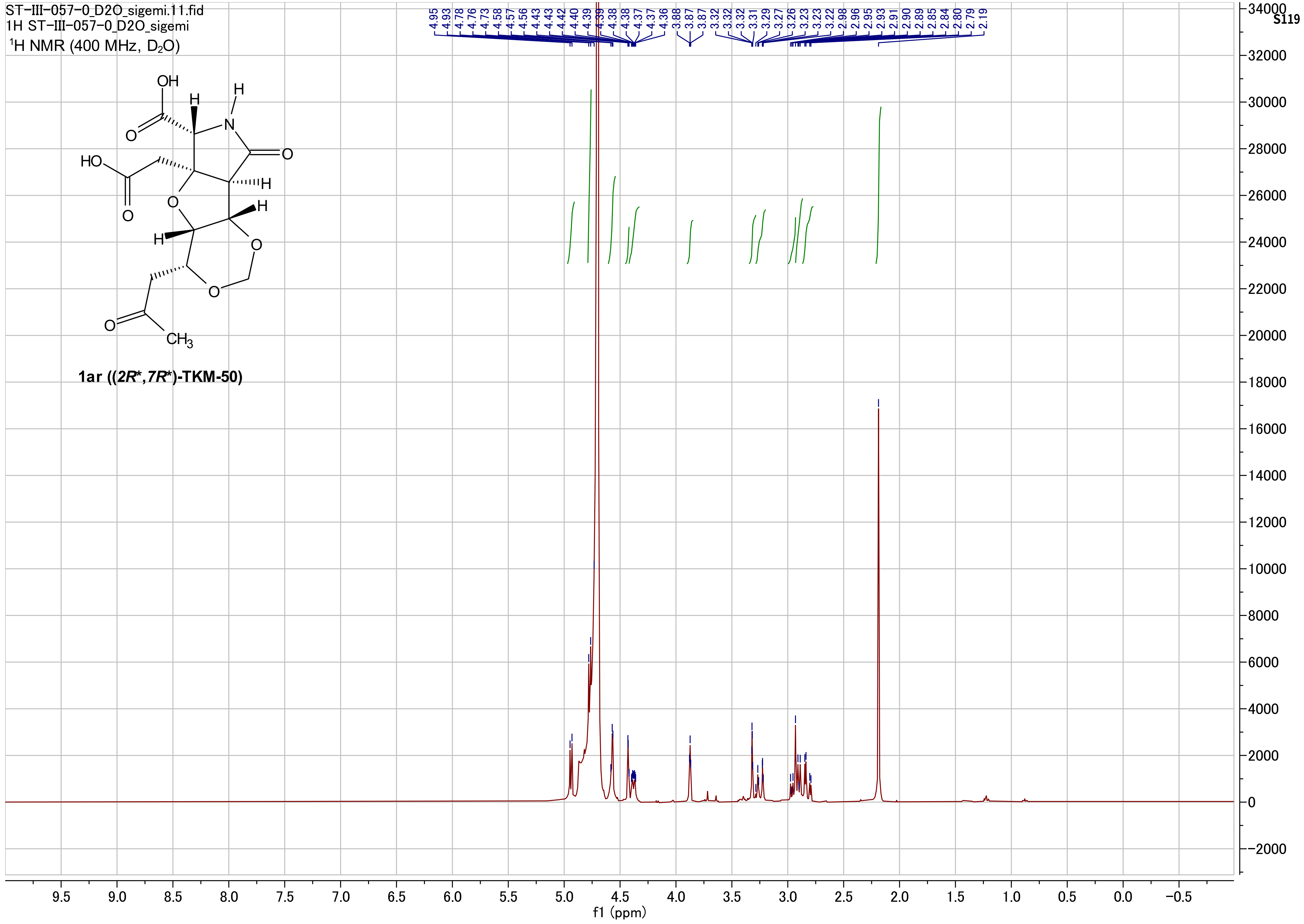

1ar ((2*R,7*R**)-TKM-50)**

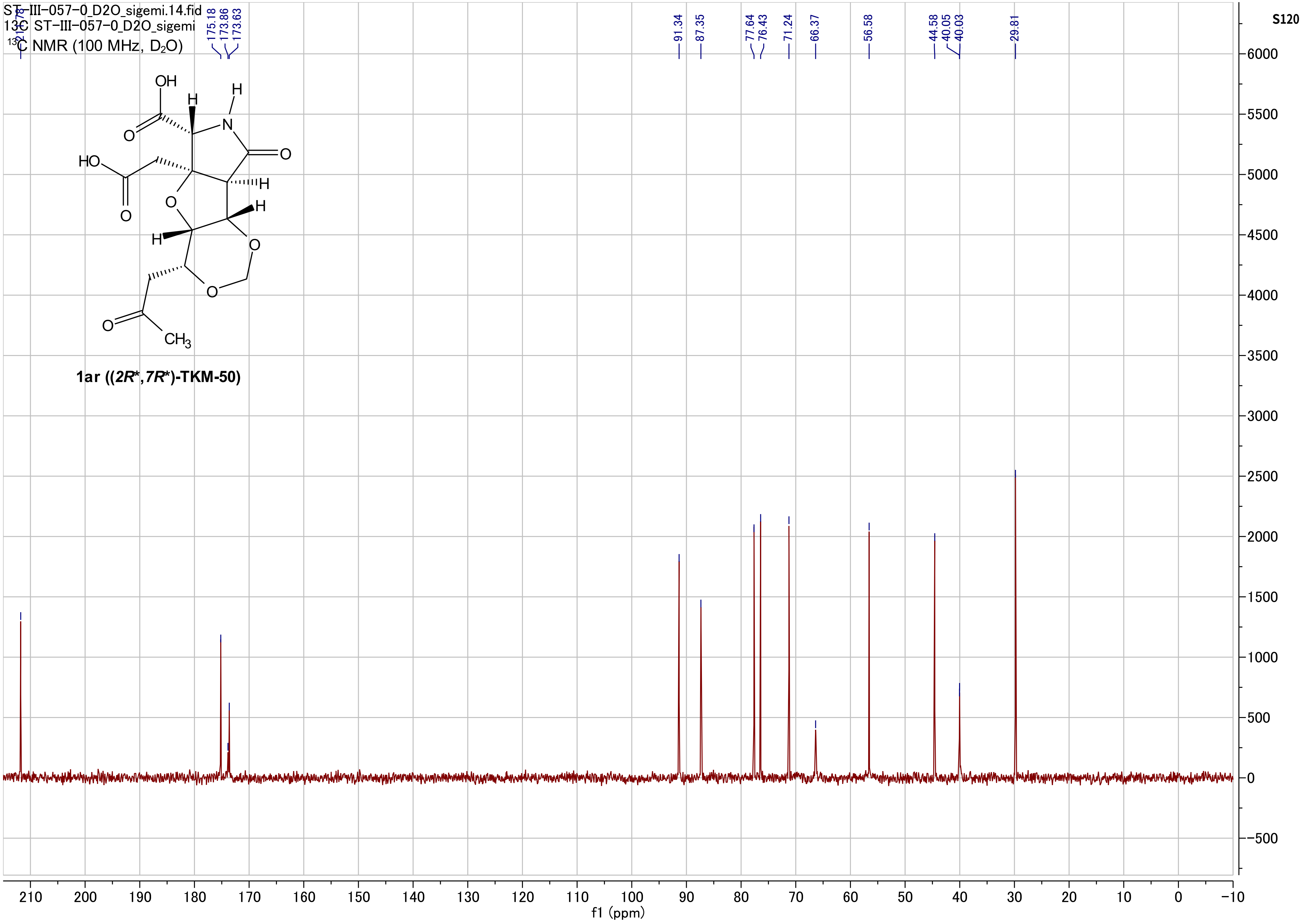
ST-III-057-0_D2O_sigemi.14.fid
13C ST-III-057-0_D2O_sigemi
13C NMR (100 MHz, D2O)
211.78
175.18
173.86
173.63
91.34
87.35
77.64
76.43
71.24
66.37
56.58
44.58
40.05
40.03
29.81
OH
H
H
N
O
O
HO
H
O
O
H
H
O
O
O
CH3
1ar ((2R*,7R*)-TKM-50)
6000
5500
5000
4500
4000
3500
3000
2500
2000
1500
1000
500
0
−500
210
200
190
180
170
160
150
140
130
120
110
100
90
80
70
60
50
40
30
20
10
0
−10
f1 (ppm)

ST-III-059-1-10.fid
1H ST-III-059-1
^{1}H NMR (400 MHz, $CDCl_3$)

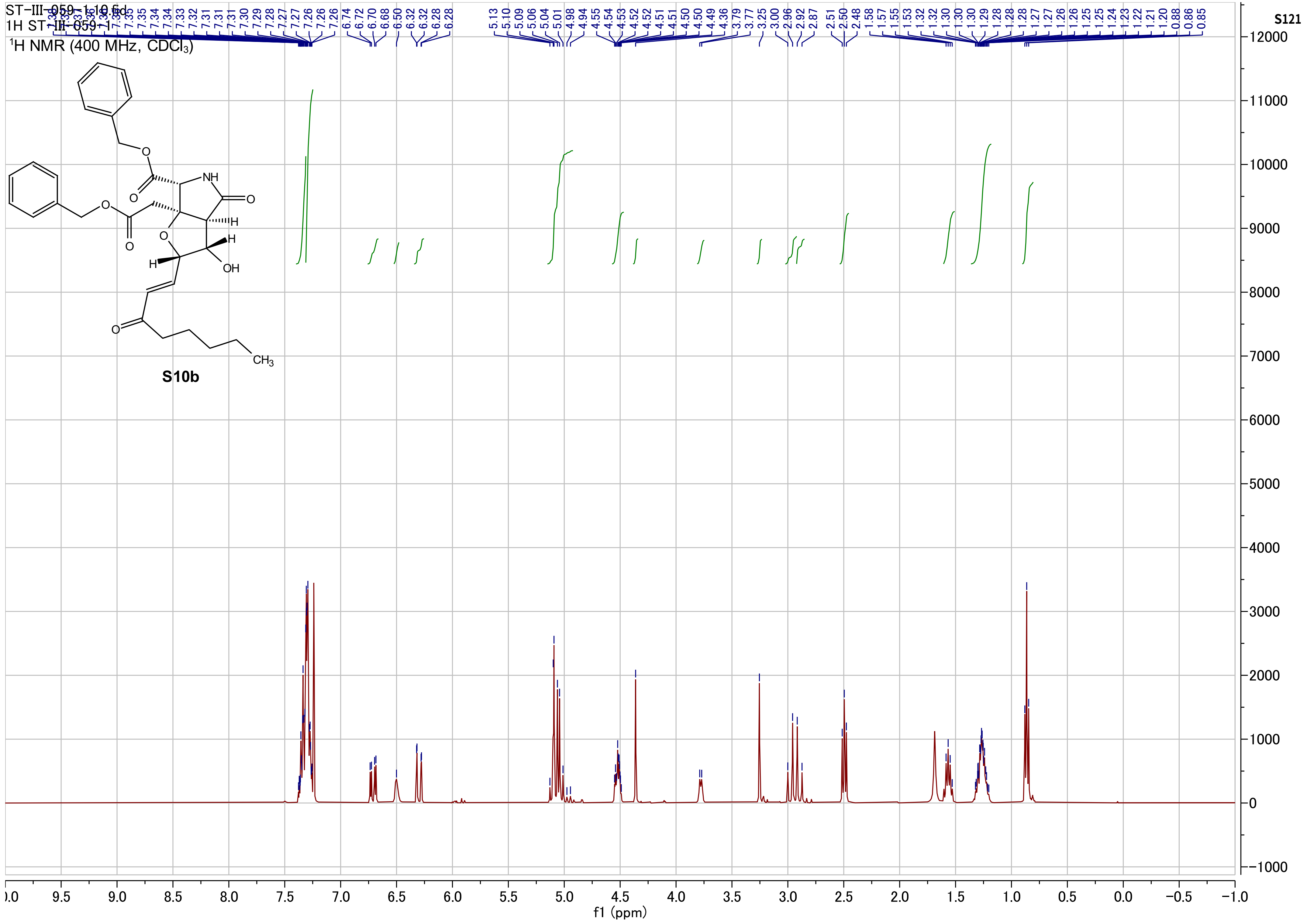

S10b

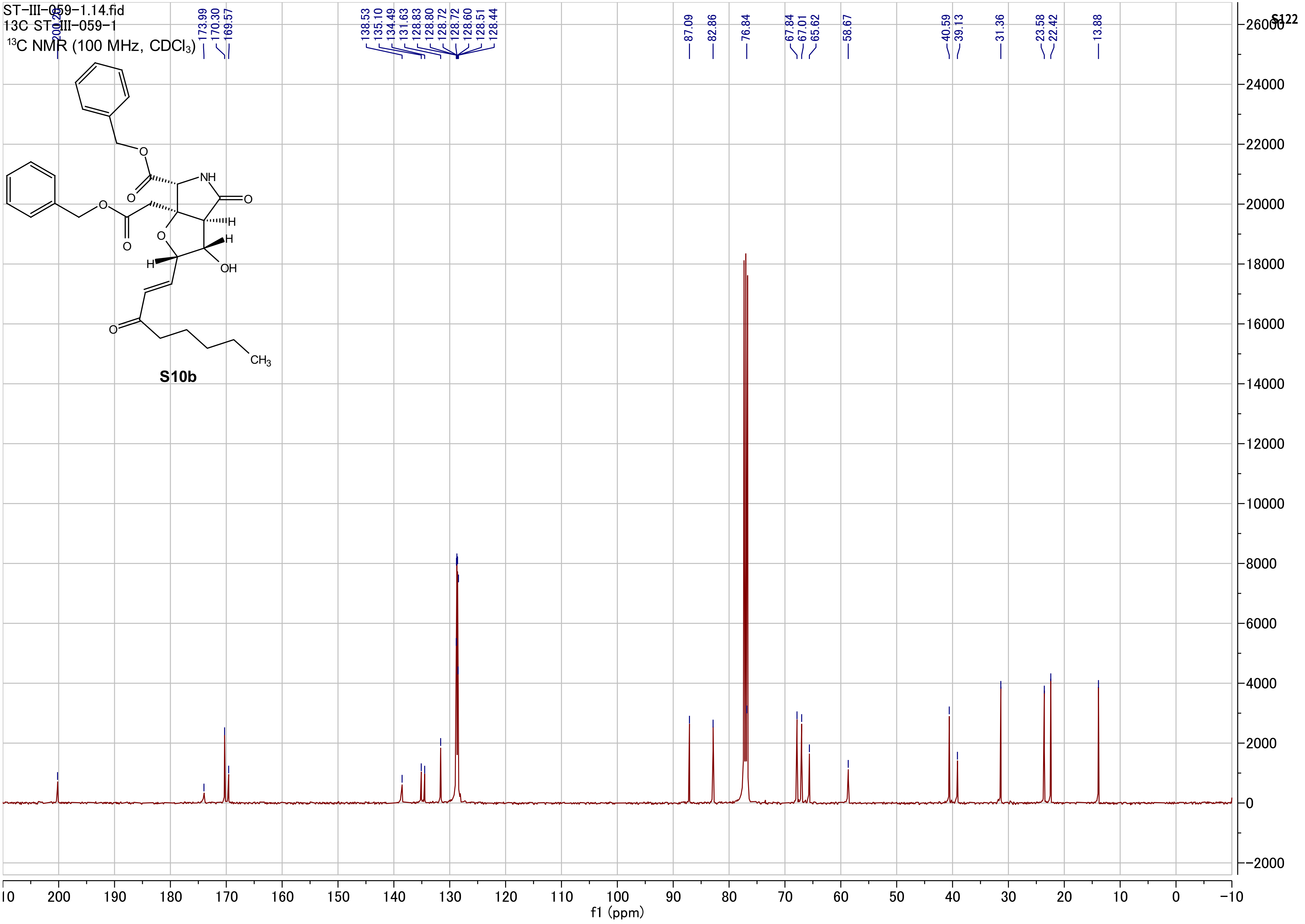
ST-III-059-1.14.fid
13C ST-III-059-1
13C NMR (100 MHz, CDCl3)
200.20
173.99
170.30
169.57
138.53
135.10
134.49
131.63
128.83
128.80
128.72
128.72
128.60
128.51
128.44
87.09
82.86
76.84
67.84
67.01
65.62
58.67
40.59
39.13
31.36
23.58
22.42
13.88
S10b
f1 (ppm)

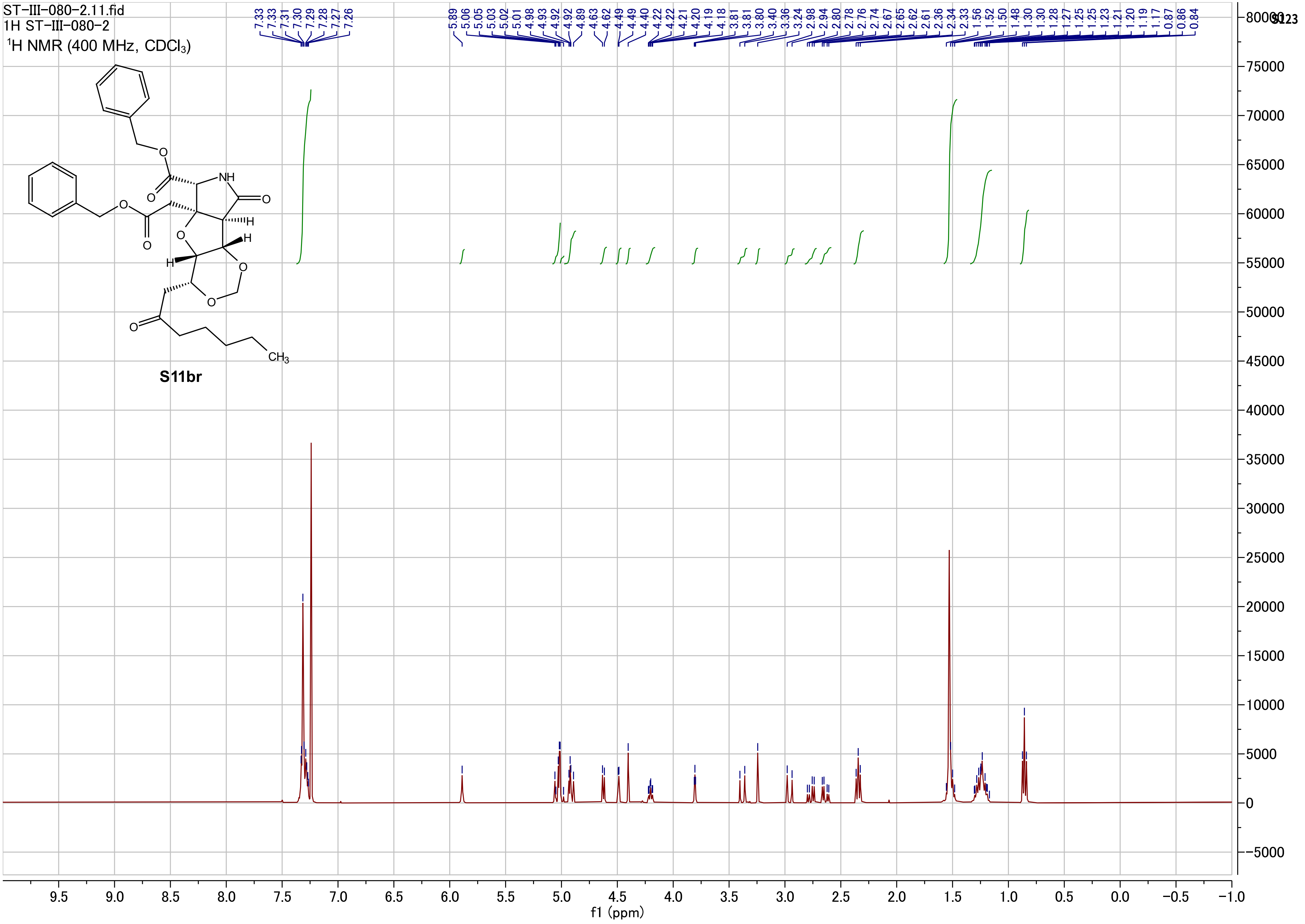

ST-III-080-2.11.fid
1H ST-III-080-2
^{1}H NMR (400 MHz, $CDCl_3$)
7.33 7.33 7.31 7.30 7.29 7.28 7.27 7.26
5.89 5.06 5.05 5.03 5.02 5.01 4.98 4.93 4.92 4.92 4.89 4.63 4.62 4.49 4.49 4.40 4.22 4.22 4.21 4.20 4.19 4.18 3.81 3.81 3.80 3.40 3.36 3.24 2.98 2.94 2.80 2.78 2.76 2.74 2.67 2.65 2.62 2.61 2.36 2.34 2.33 1.56 1.52 1.50 1.48 1.30 1.28 1.27 1.25 1.23 1.21 1.20 1.19 1.17 0.87 0.86 0.84
NH
O
H
CH_3
S11br
f1 (ppm)

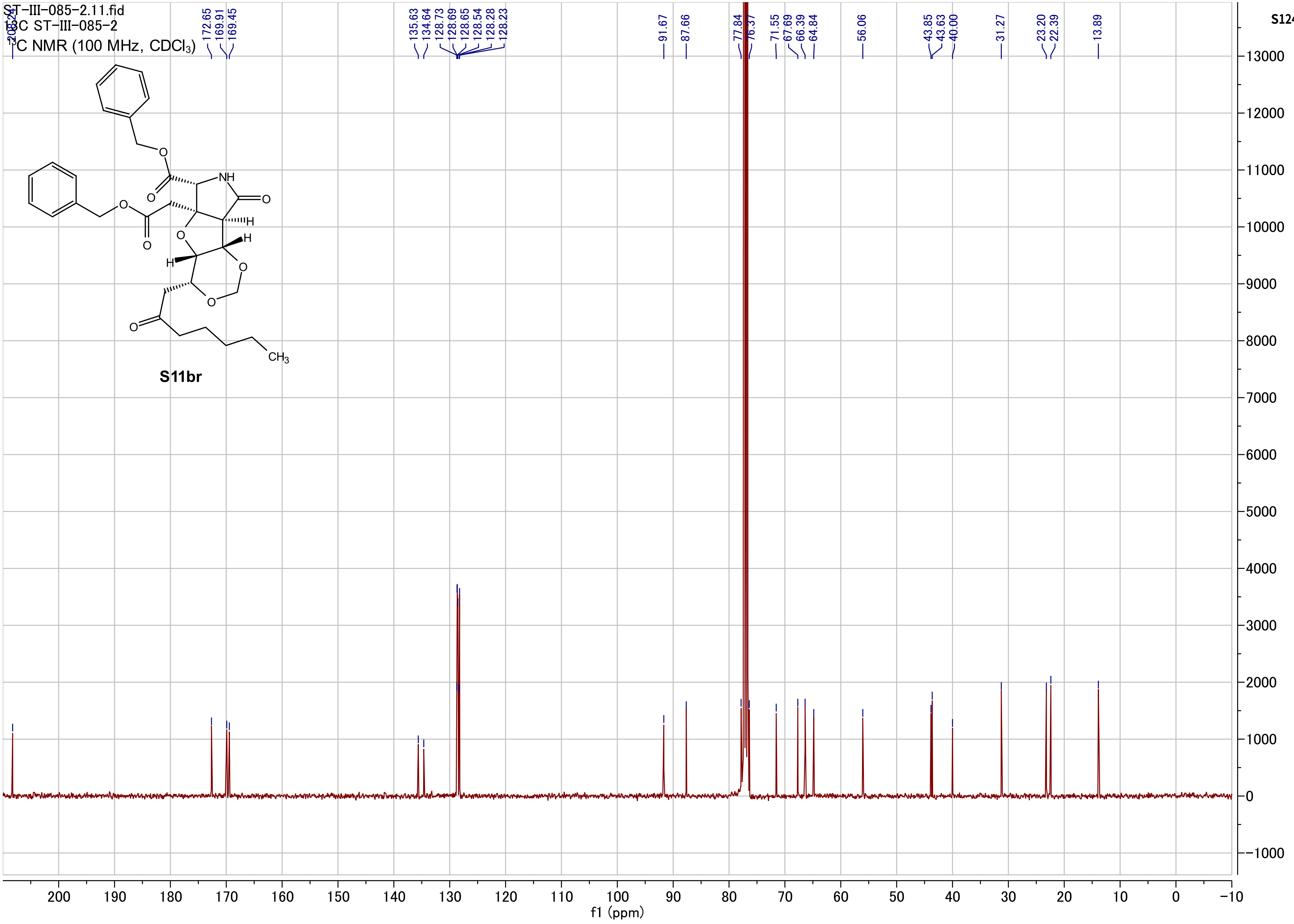
ST−III−085−2.11.fid
13C ST−III−085−2
13C NMR (100 MHz, CDCl3)
208.24
172.65
169.91
169.45
135.63
134.64
128.73
128.69
128.65
128.54
128.28
128.23
91.67
87.66
77.84
76.37
71.55
67.69
66.39
64.84
56.06
43.85
43.63
40.00
31.27
23.20
22.39
13.89
NH
O
H
CH3
S11br
f1 (ppm)

ST-III-085-1.10.fid
1H ST-III-085-1
^{1}H NMR (400 MHz, $CDCl_3$)

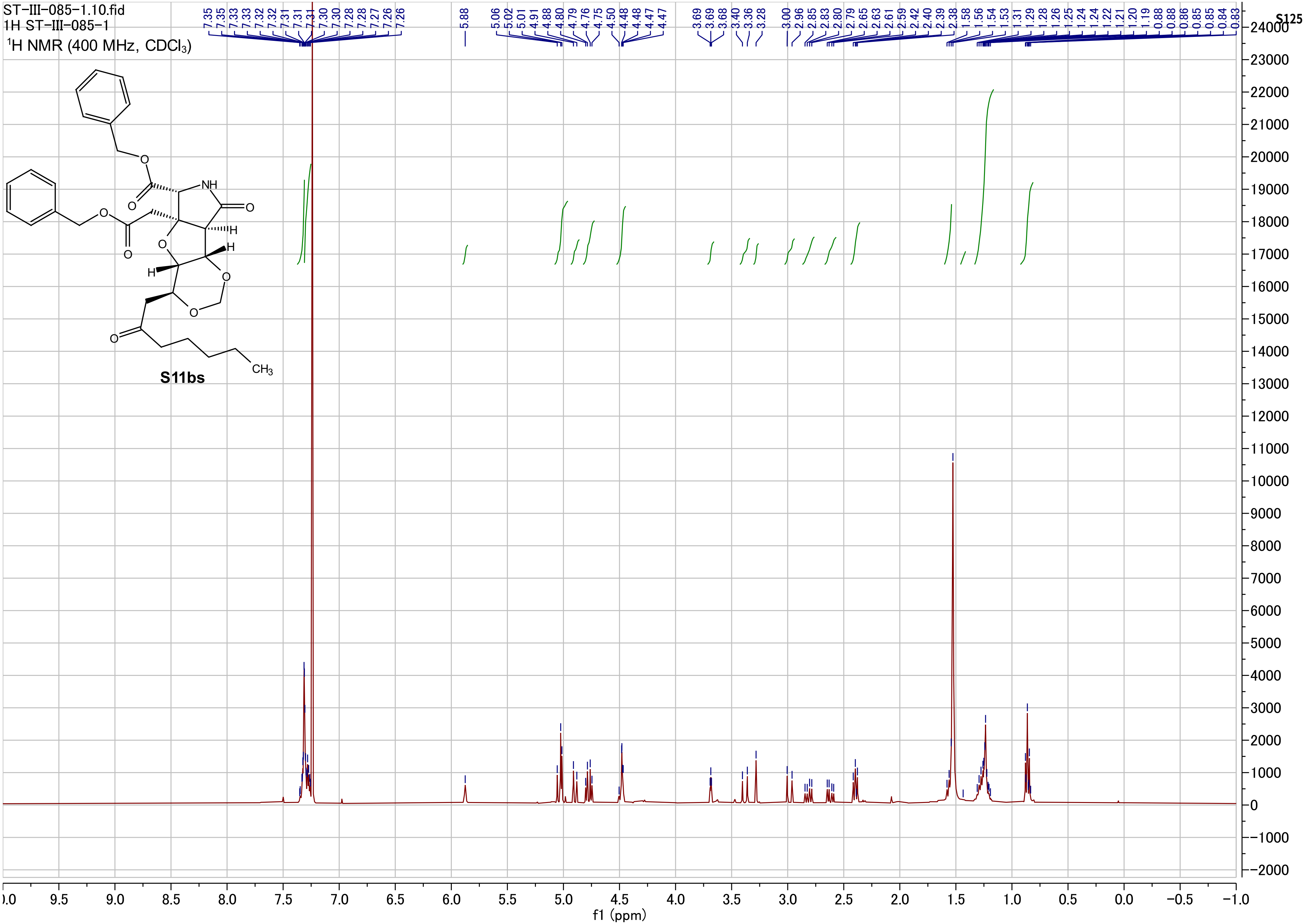

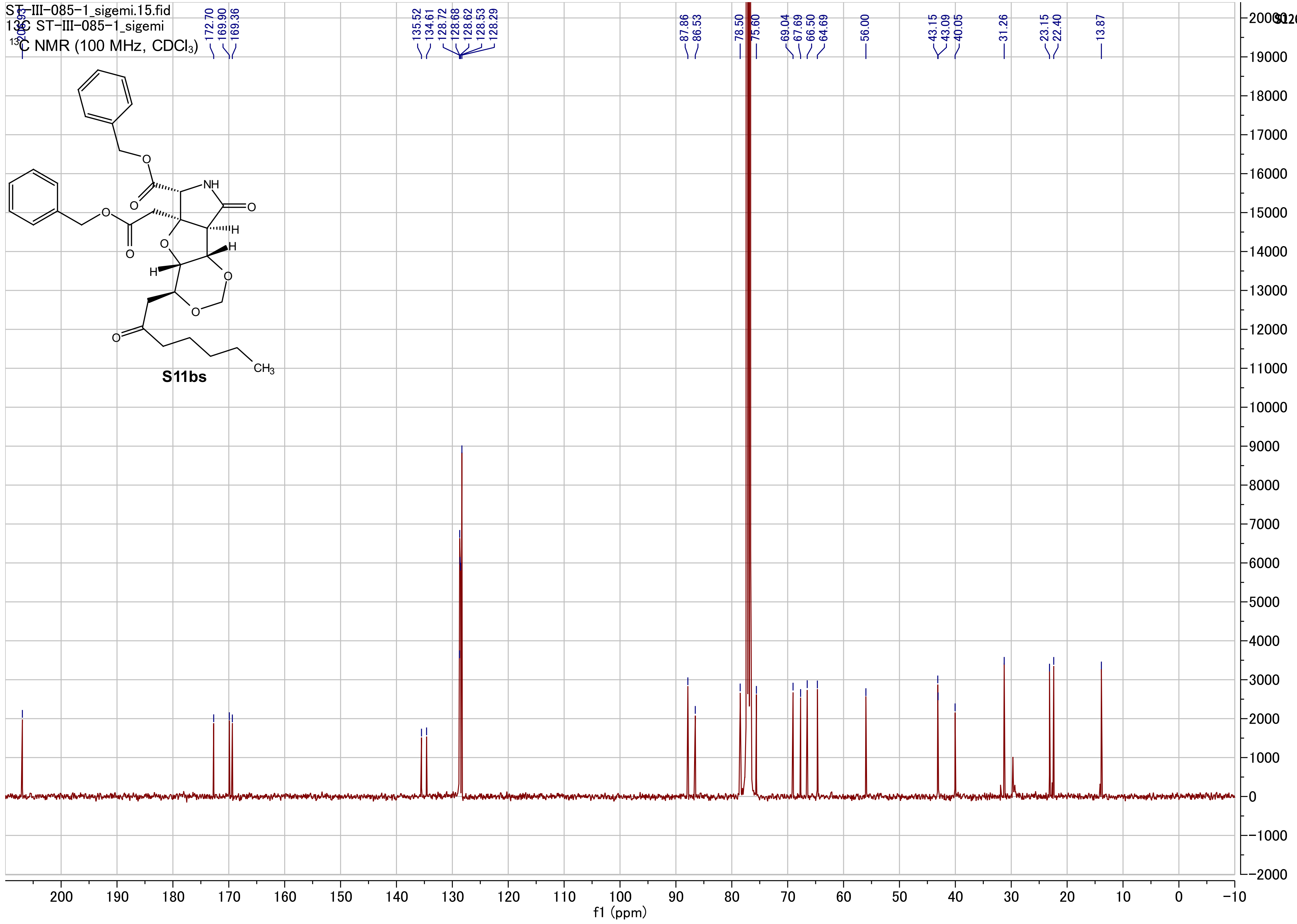

ST–III–085–1_sigemi.15.fid
13C ST–III–085–1_sigemi
^{13}C NMR (100 MHz, $CDCl_3$)
206.93
172.70
169.90
169.36
135.52
134.61
128.72
128.68
128.62
128.53
128.29
87.86
86.53
78.50
75.60
69.04
67.69
66.50
64.69
56.00
43.15
43.09
40.05
31.26
23.15
22.40
13.87
NH
O
H
CH_3
S11bs
f1 (ppm)

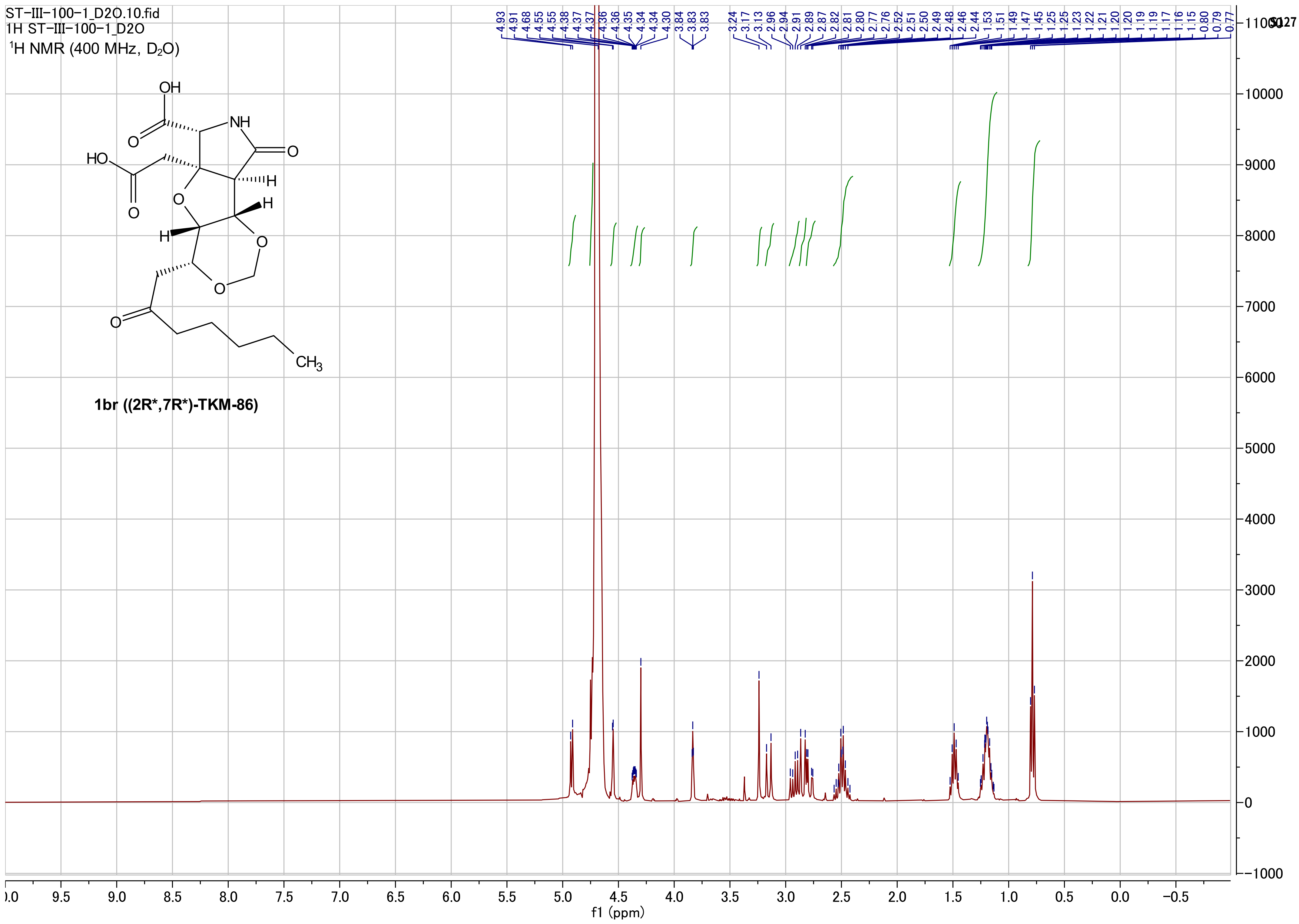
ST-III-100-1_D2O.10.fid
1H ST-III-100-1_D2O
^{1}H NMR (400 MHz, D_2O)
1br ((2R*,7R*)-TKM-86)
OH
NH
O
HO
H
O
CH3
4.93 4.91 4.68 4.55 4.55 4.38 4.37 4.37 4.36 4.36 4.35 4.34 4.34 4.30 3.84 3.83 3.83 3.24 3.17 3.13 2.96 2.94 2.91 2.89 2.87 2.82 2.81 2.80 2.77 2.76 2.52 2.51 2.50 2.49 2.48 2.46 2.44 1.53 1.51 1.49 1.47 1.45 1.25 1.25 1.23 1.22 1.21 1.20 1.20 1.19 1.17 1.16 1.15 0.80 0.79 0.77
f1 (ppm)

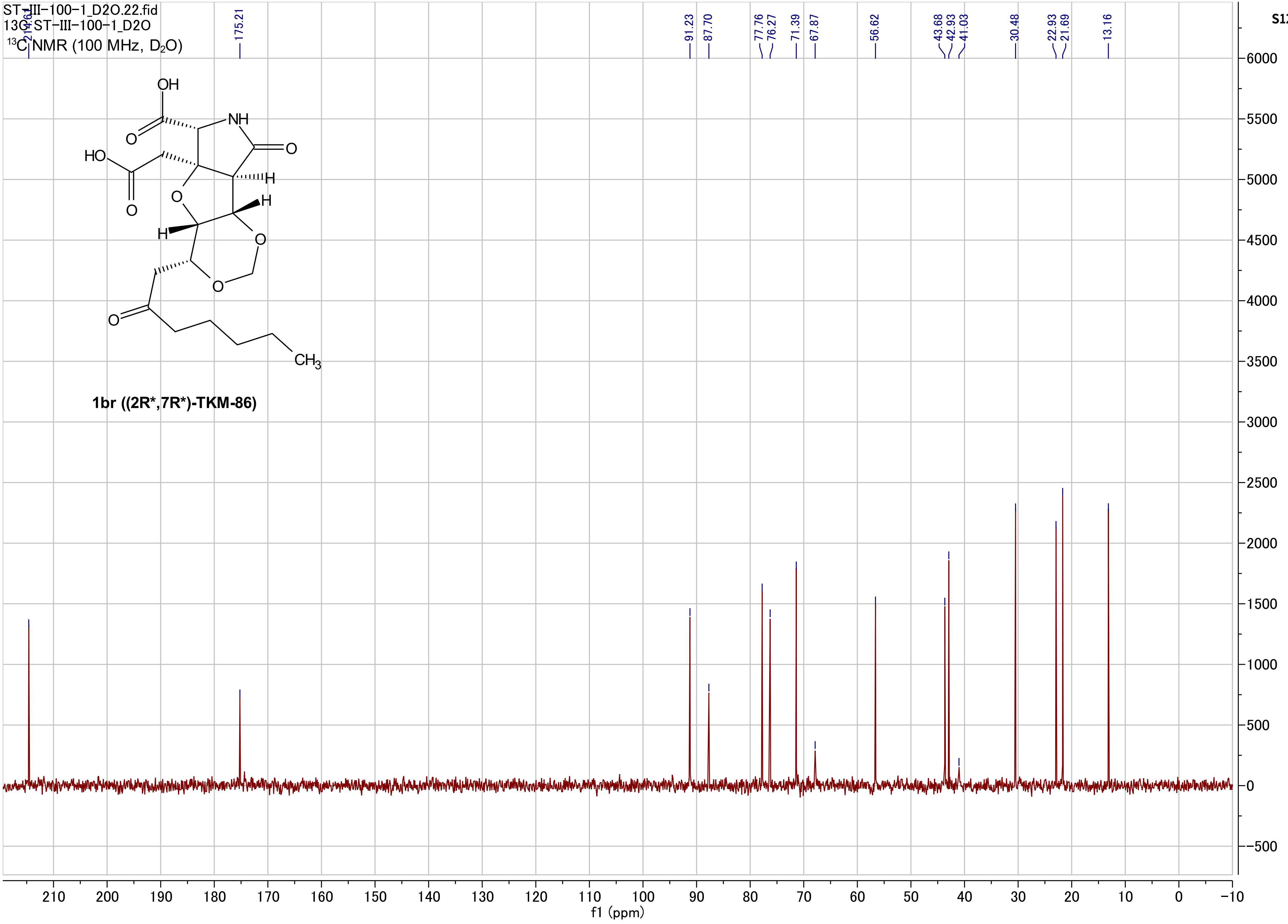
ST-III-100-1_D2O.22.fid
13C-ST-III-100-1_D2O
13C NMR (100 MHz, D2O)
214.61
175.21
91.23
87.70
77.76
76.27
71.39
67.87
56.62
43.68
42.93
41.03
30.48
22.93
21.69
13.16
OH
NH
O
HO
O
O
H
H
H
O
O
O
CH3
1br ((2R*,7R*)-TKM-86)
f1 (ppm)

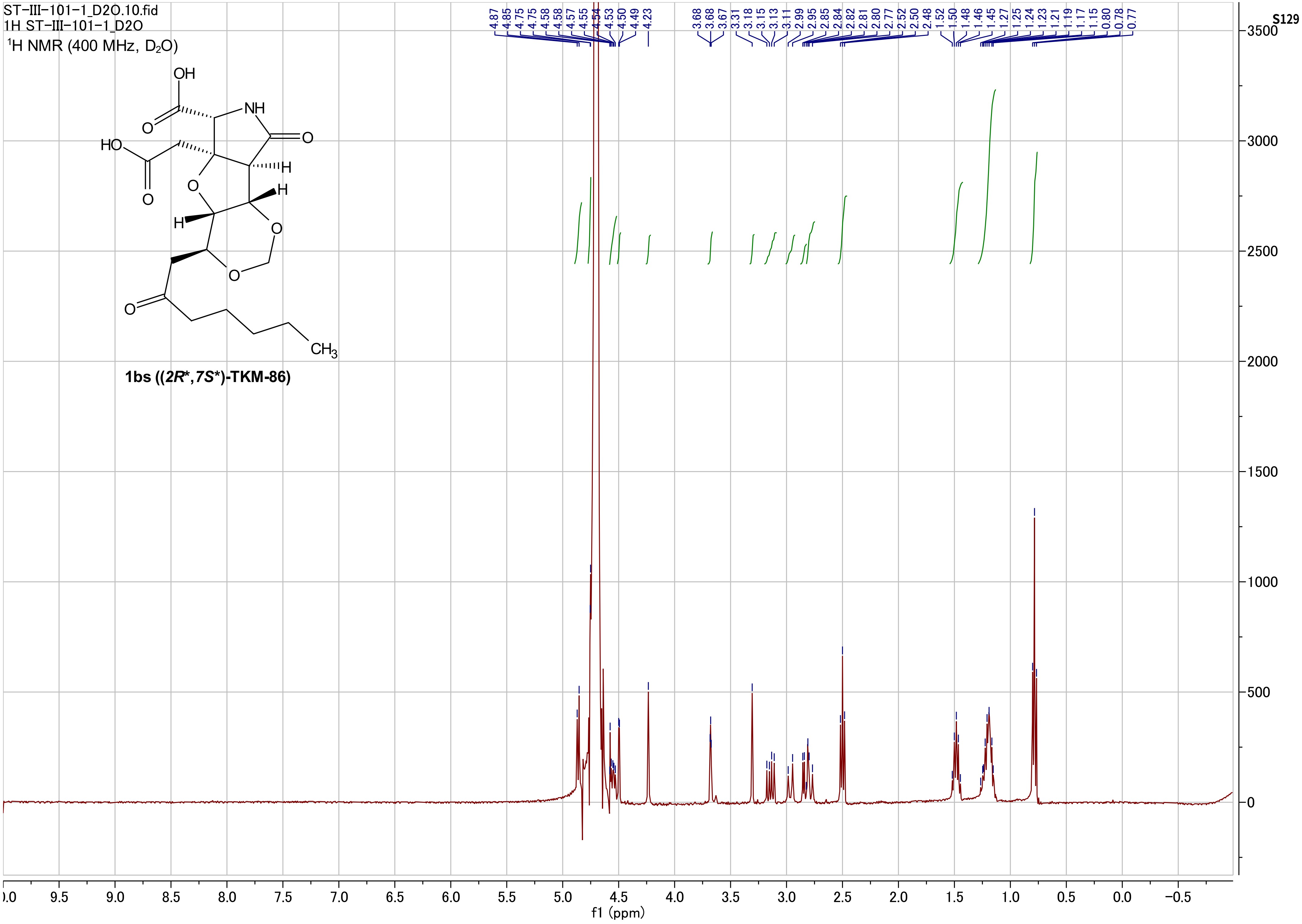

1bs ((2*R,7*S**)-TKM-86)**

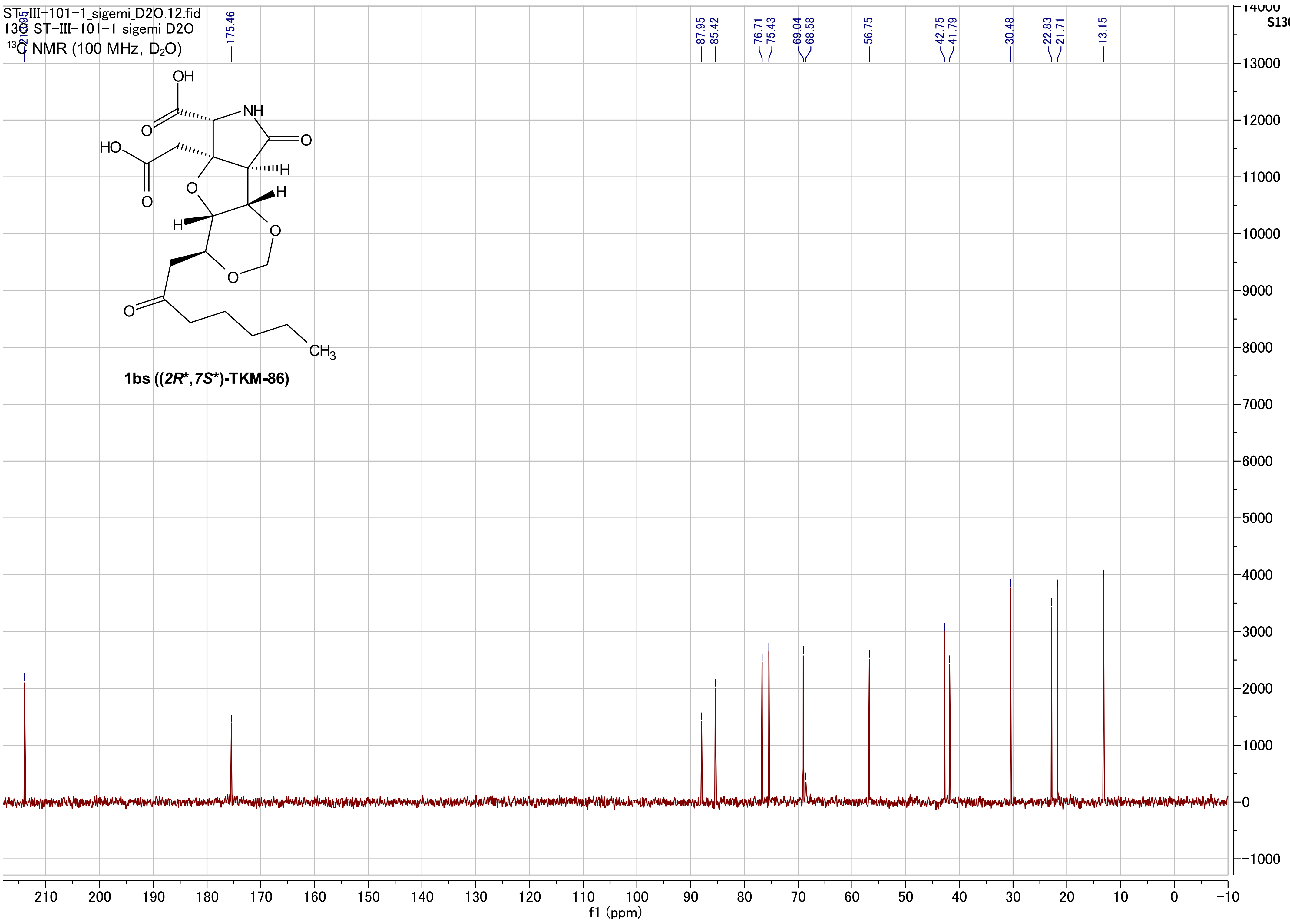
ST-III-101-1_sigemi_D2O.12.fid
13C ST-III-101-1_sigemi_D2O
^{13}C NMR (100 MHz, D_2O)
213.95
175.46
87.95
85.42
76.71
75.43
69.04
68.58
56.75
42.75
41.79
30.48
22.83
21.71
13.15
OH
NH
O
HO
H
CH3
1bs ((2R*,7S*)-TKM-86)
f1 (ppm)

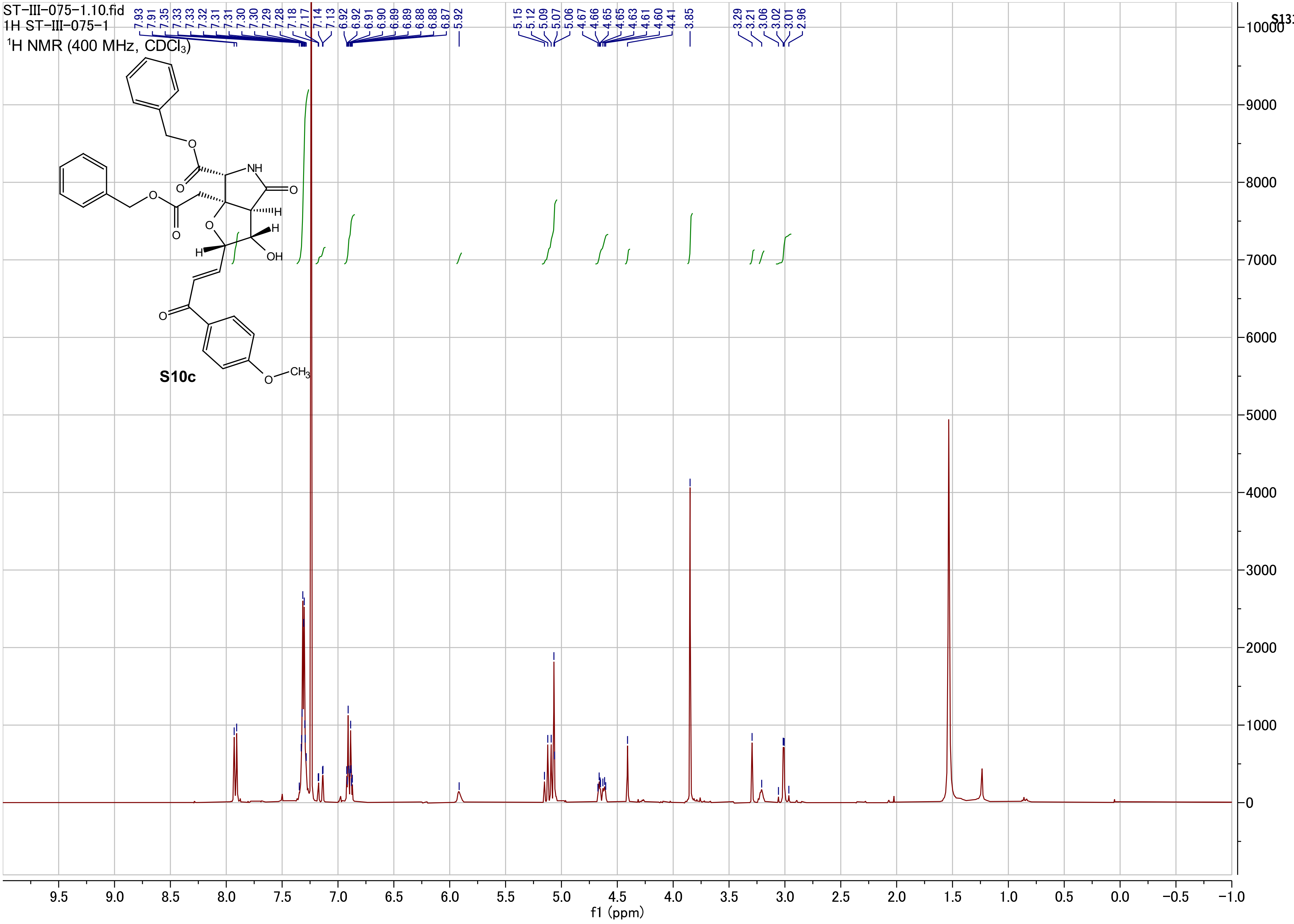
ST-III-075-1.10.fid
1H ST-III-075-1
^{1}H NMR (400 MHz, CDCl$_3$)
7.93 7.91 7.35 7.33 7.33 7.32 7.31 7.31 7.30 7.30 7.29 7.28 7.18 7.17 7.14 7.13 6.92 6.92 6.91 6.90 6.89 6.88 6.88 6.87 5.92 5.15 5.12 5.09 5.07 5.06 4.67 4.66 4.65 4.65 4.63 4.61 4.60 4.41 3.85 3.29 3.21 3.06 3.02 3.01 2.96
S10c
f1 (ppm)

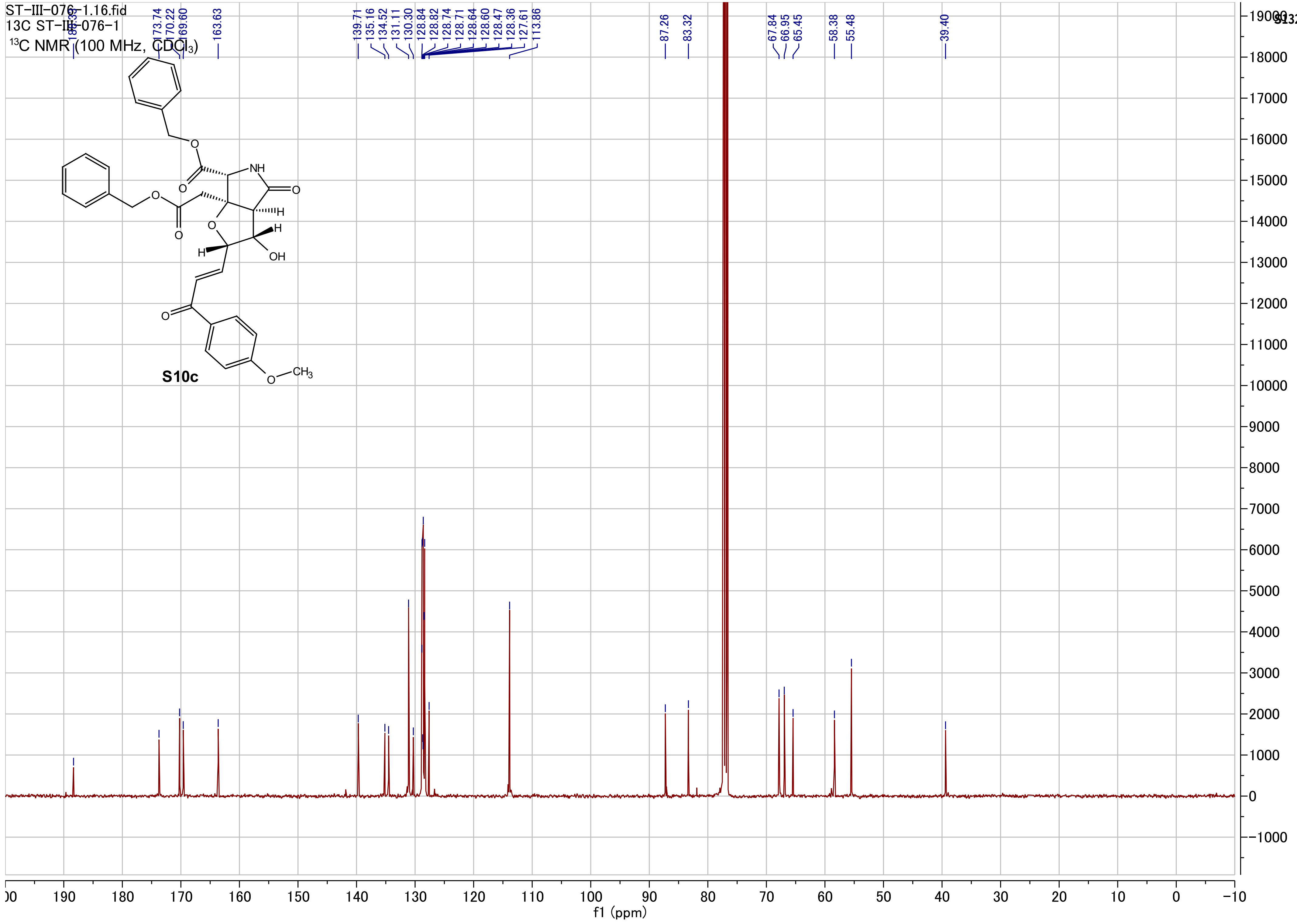
ST-III-076-1.16.fid
13C ST-III-076-1
13C NMR (100 MHz, CDCl3)
173.74
170.22
169.60
163.63
139.71
135.16
134.52
131.11
130.30
128.84
128.82
128.74
128.71
128.64
128.60
128.47
128.36
127.61
113.86
87.26
83.32
67.84
66.95
65.45
58.38
55.48
39.40
NH
OH
O
H
CH3
S10c
f1 (ppm)

ST-III-091-2.10.fid
1H ST-III-091-2
^{1}H NMR (400 MHz, $CDCl_3$)

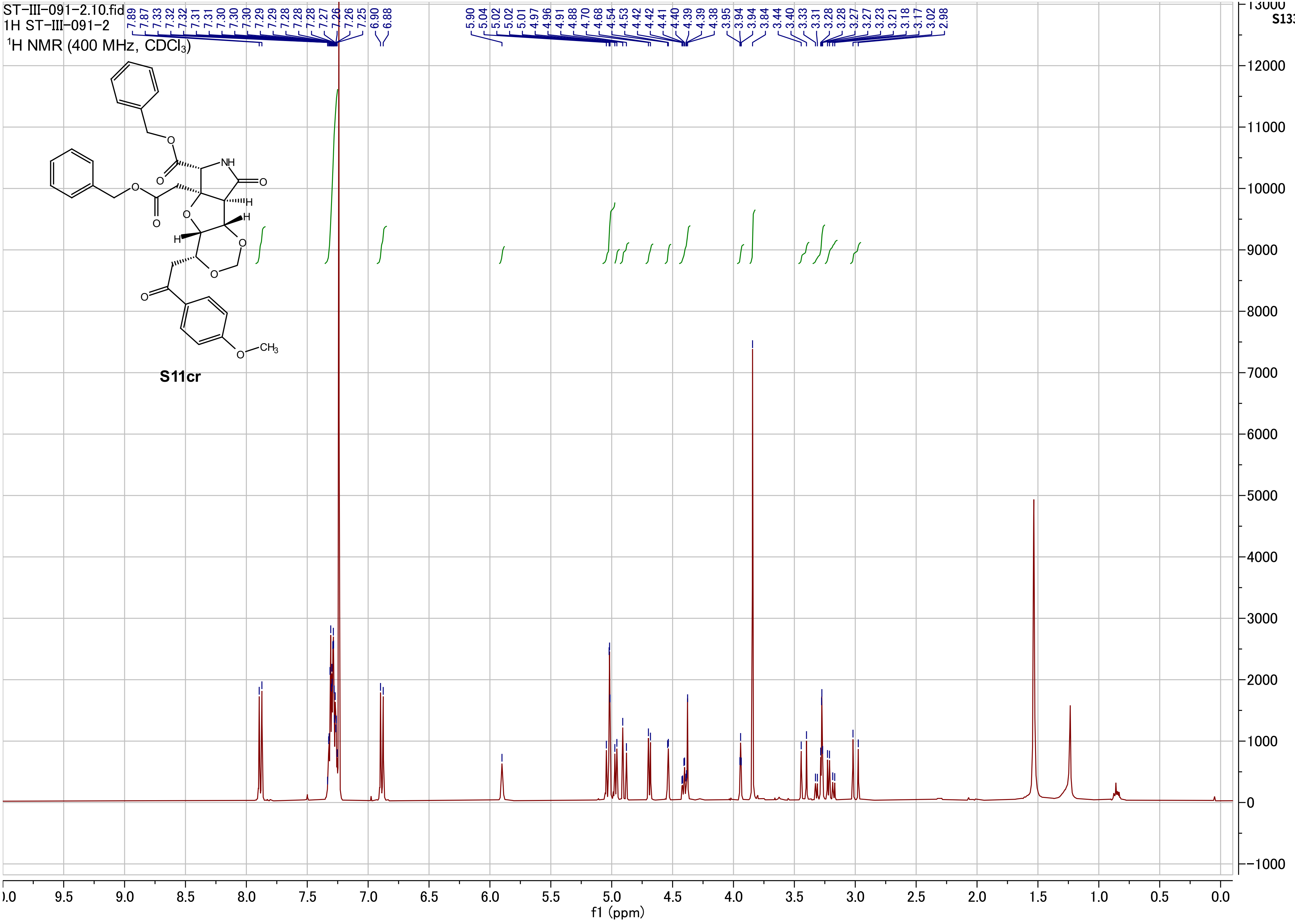

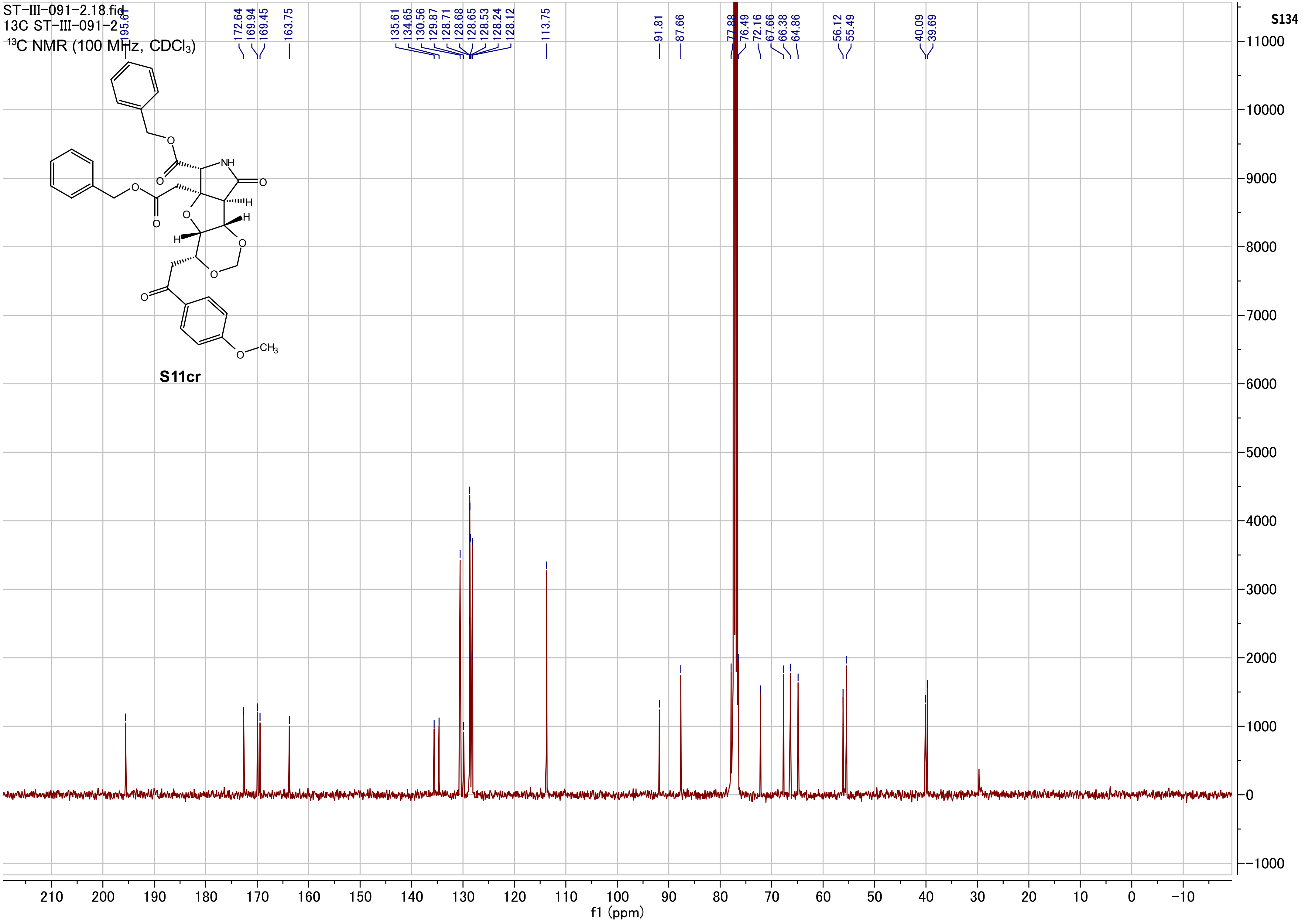
ST-III-091-2.18.fid
13C ST-III-091-2
13C NMR (100 MHz, CDCl3)
195.61
172.64
169.94
169.45
163.75
135.61
134.65
130.56
129.87
128.71
128.68
128.65
128.53
128.24
128.12
113.75
91.81
87.66
77.88
76.49
72.16
67.66
66.38
64.86
56.12
55.49
40.09
39.69
NH
O
H
CH3
S11cr
f1 (ppm)
210
200
190
180
170
160
150
140
130
120
110
100
90
80
70
60
50
40
30
20
10
0
-10
11000
10000
9000
8000
7000
6000
5000
4000
3000
2000
1000
0
-1000

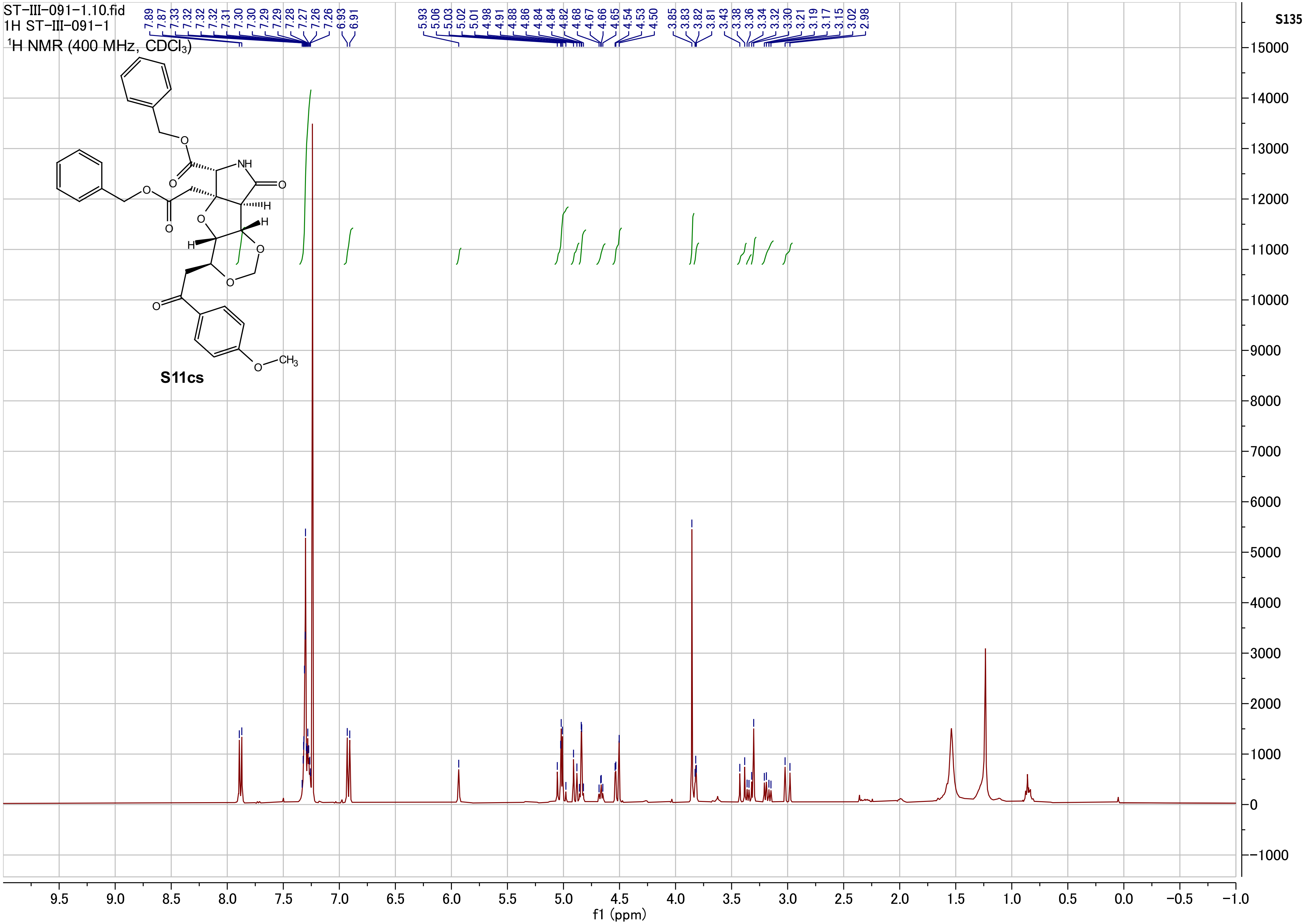
ST−III−091−1.10.fid
1H ST−III−091−1
1H NMR (400 MHz, CDCl3)
7.89 7.87 7.33 7.32 7.32 7.32 7.31 7.30 7.30 7.29 7.29 7.28 7.27 7.26 7.26 6.93 6.91
5.93 5.06 5.03 5.02 5.01 4.98 4.91 4.88 4.86 4.84 4.84 4.82 4.68 4.67 4.66 4.65 4.54 4.53 4.50
3.85 3.83 3.82 3.81 3.43 3.38 3.36 3.34 3.32 3.30 3.21 3.19 3.17 3.15 3.02 2.98
NH
O
H
CH3
S11cs
f1 (ppm)

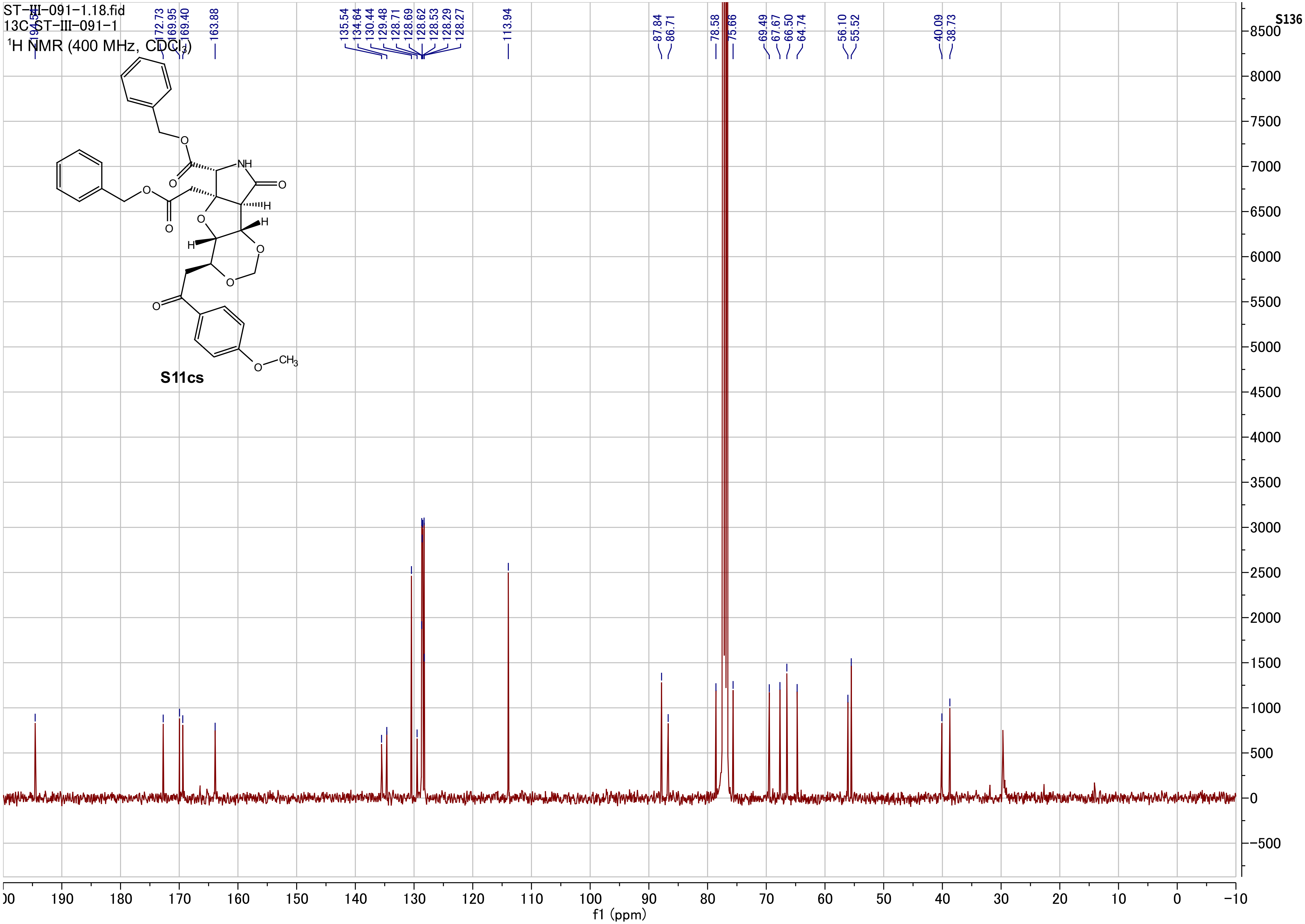

ST−III−091−1.18.fid
13C ST−III−091−1
^{1}H NMR (400 MHz, $CDCl_3$)
194.54
172.73
169.95
169.40
163.88
135.54
134.64
130.44
129.48
128.71
128.69
128.62
128.53
128.29
128.27
113.94
87.84
86.71
78.58
75.66
69.49
67.67
66.50
64.74
56.10
55.52
40.09
38.73
S11cs
f1 (ppm)

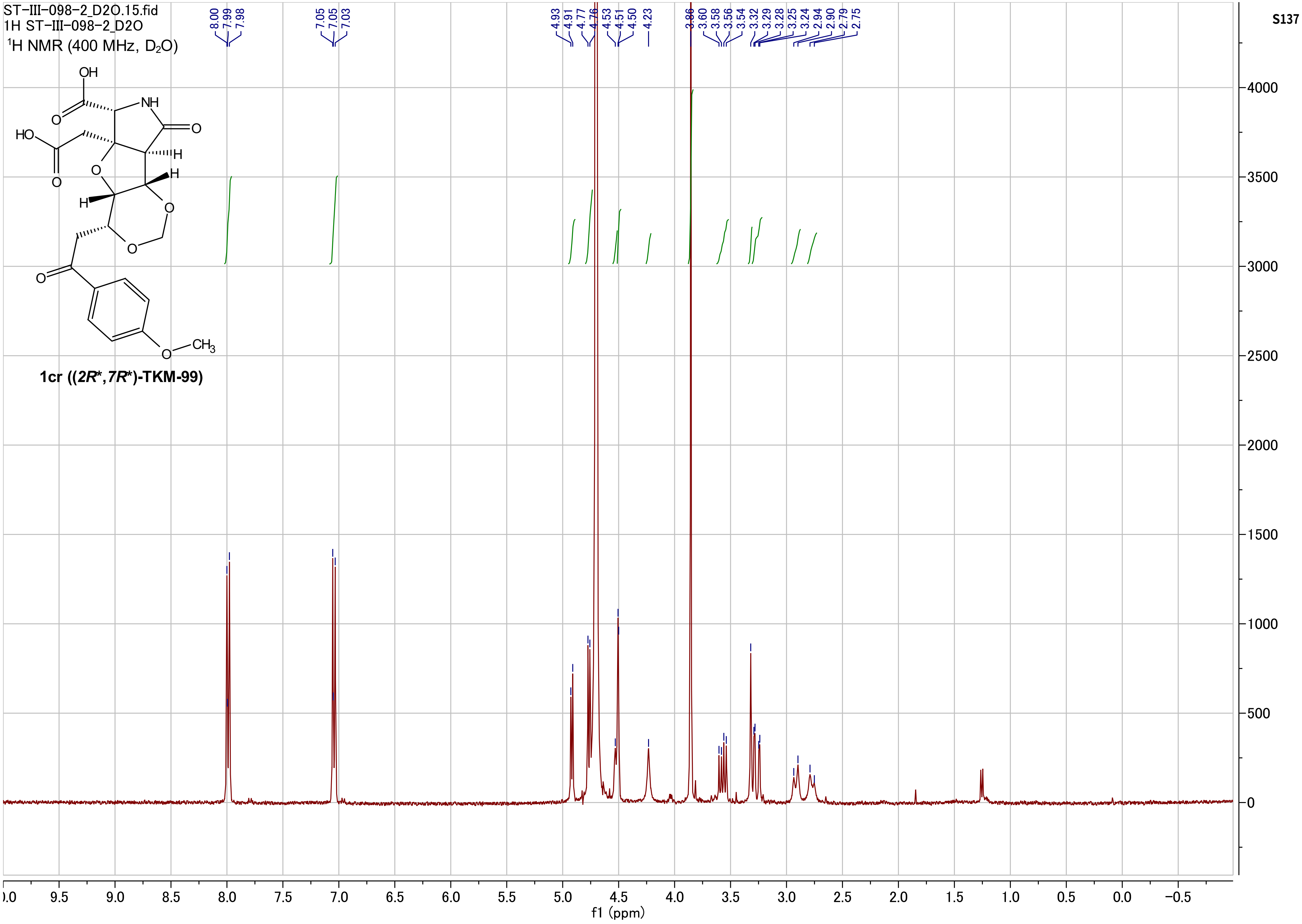

1cr ((2*R,7*R**)-TKM-99)**

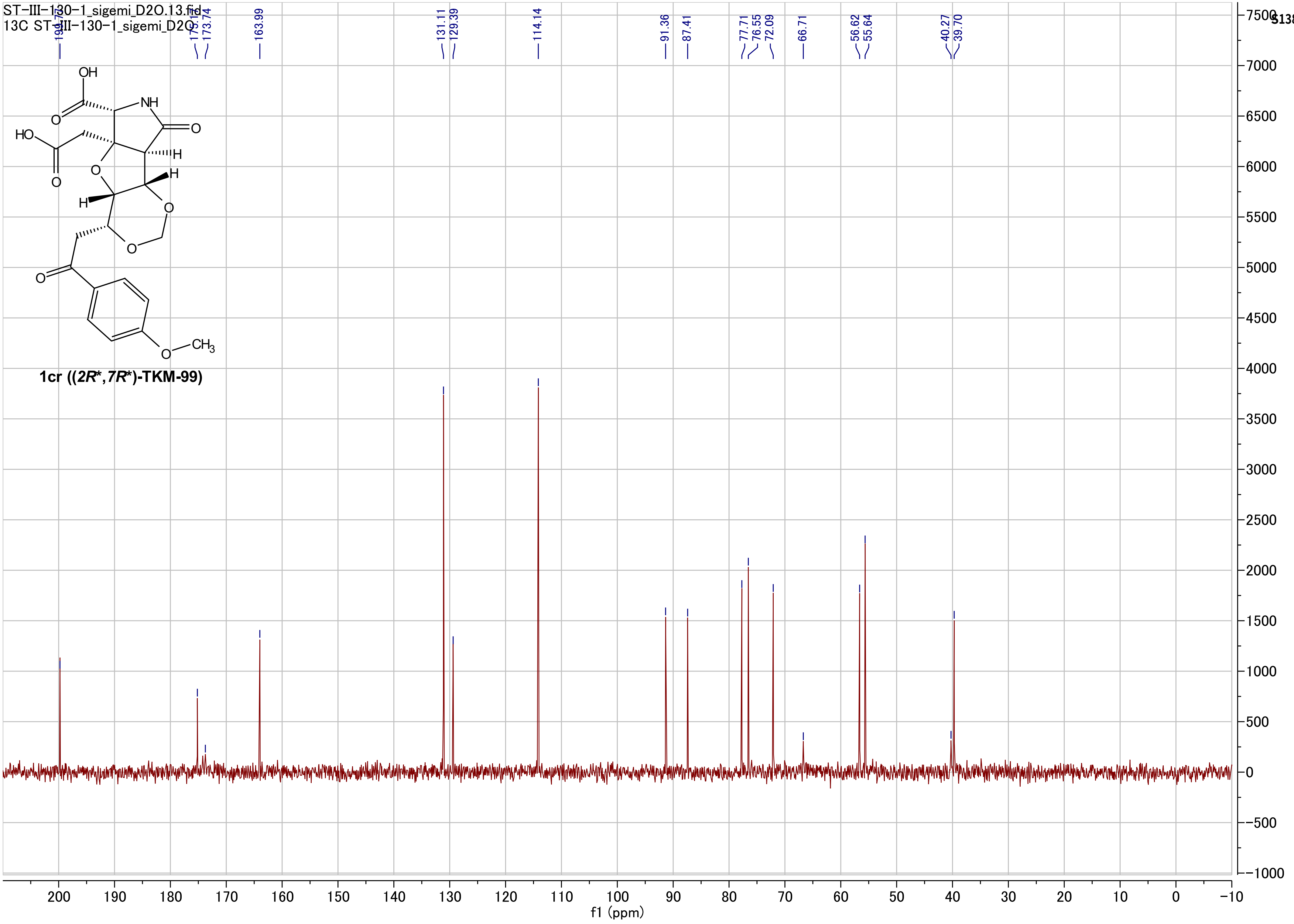

ST−III−130−1_sigemi_D2O.13.fid
13C ST−III−130−1_sigemi_D2O
199.77
175.17
173.74
163.99
131.11
129.39
114.14
91.36
87.41
77.71
76.55
72.09
66.71
56.62
55.64
40.27
39.70
1cr ((2R*,7R*)-TKM-99)
f1 (ppm)

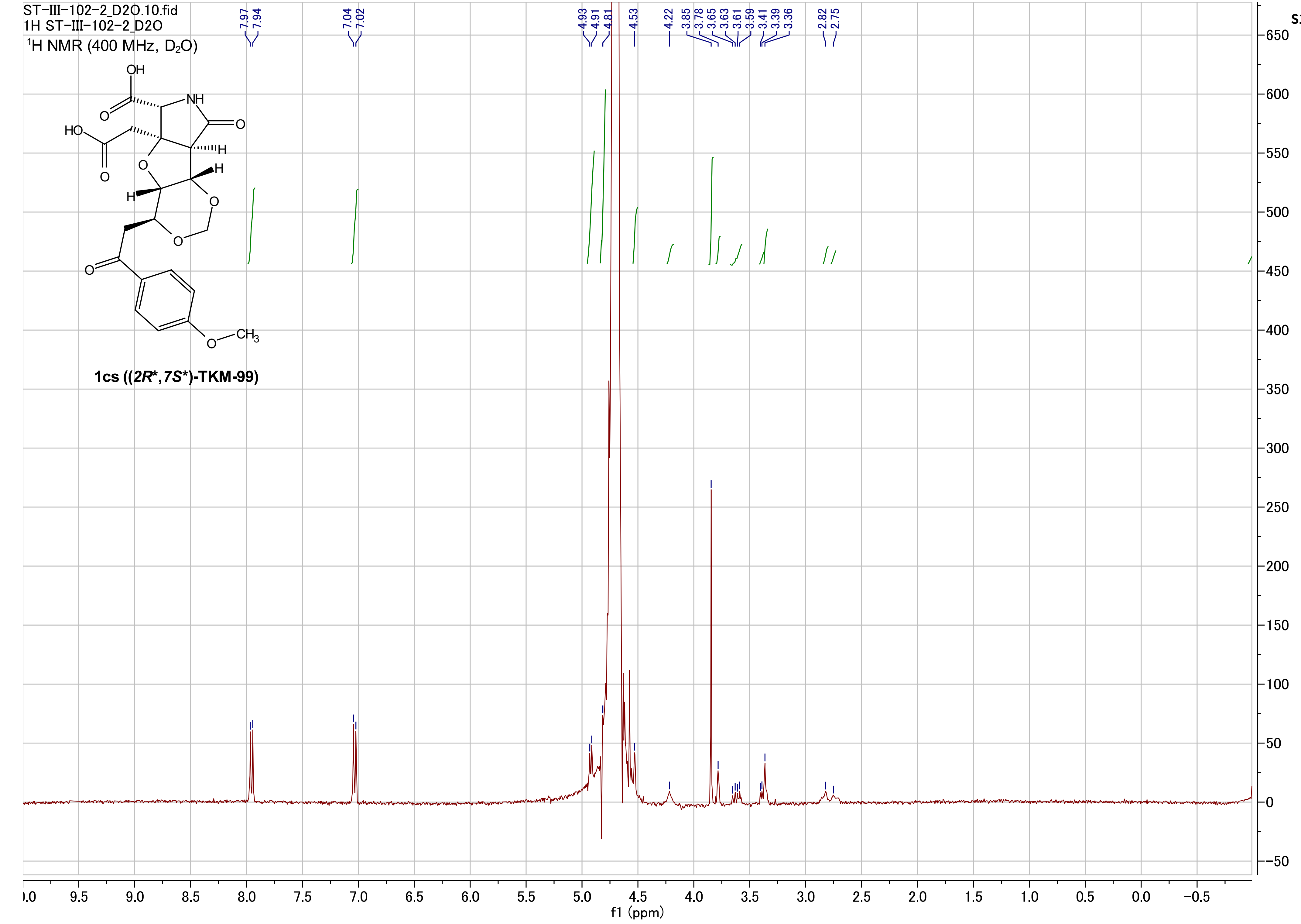
ST-III-102-2_D2O.10.fid
1H ST-III-102-2_D2O
^{1}H NMR (400 MHz, D_2O)
1cs ((2*R**,7*S**)-TKM-99)
7.97
7.94
7.04
7.02
4.93
4.91
4.81
4.53
4.22
3.85
3.78
3.65
3.63
3.61
3.59
3.41
3.39
3.36
2.82
2.75
f1 (ppm)

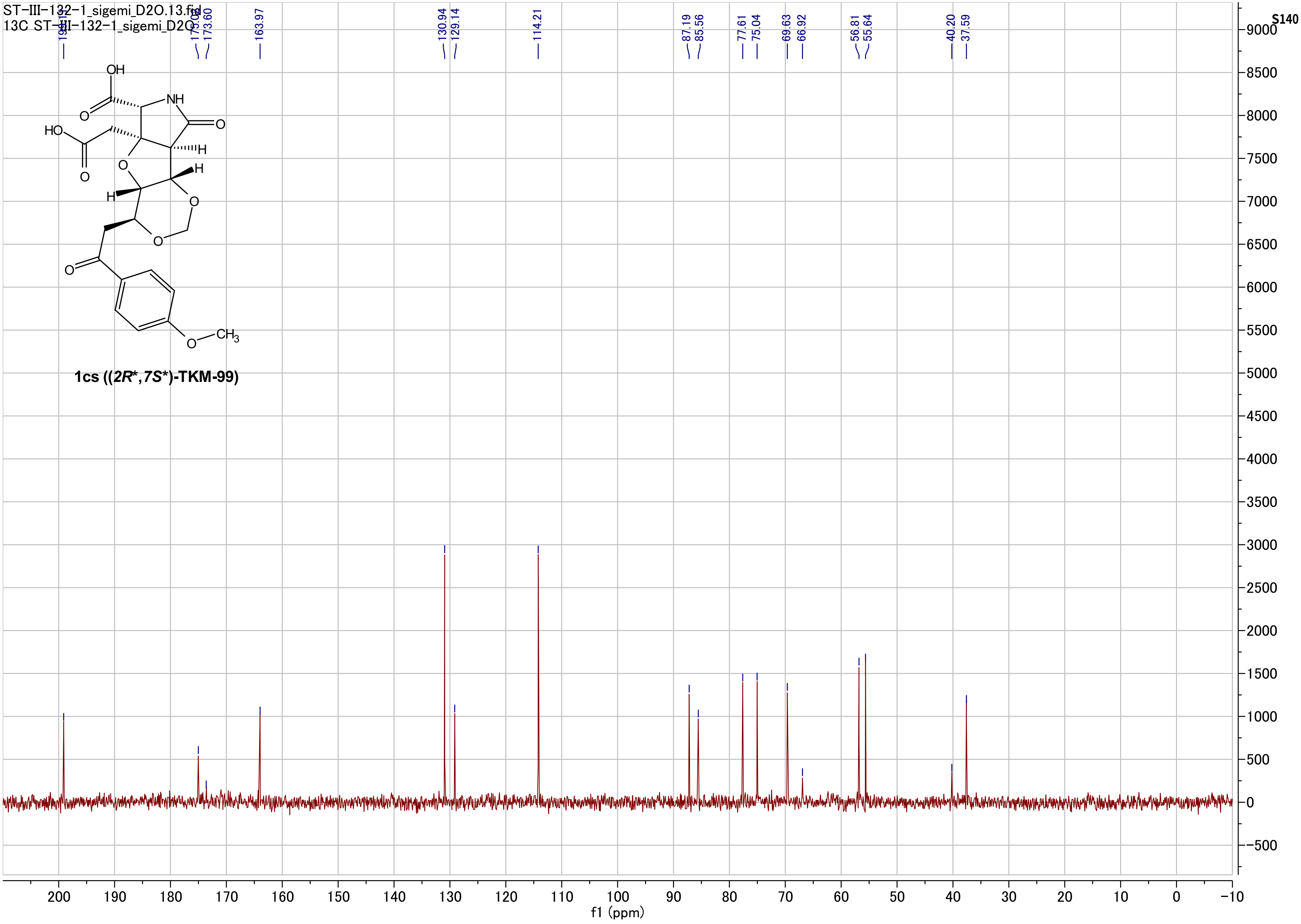

1cs ((2*R,7*S**)-TKM-99)**

ST-III-003-3.10.fid
1H ST-III-003-3
^{1}H NMR (400 MHz, $CDCl_3$)

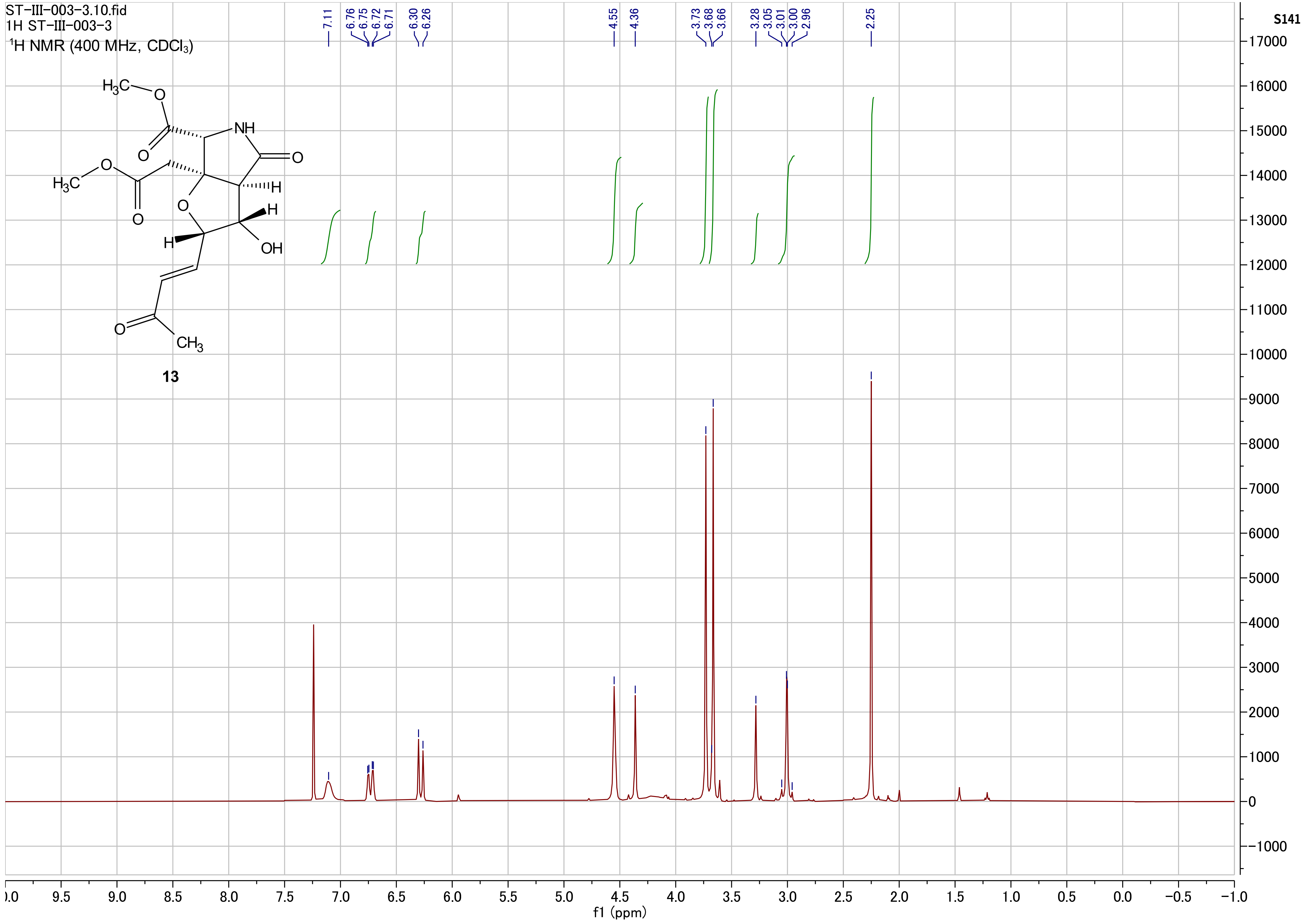

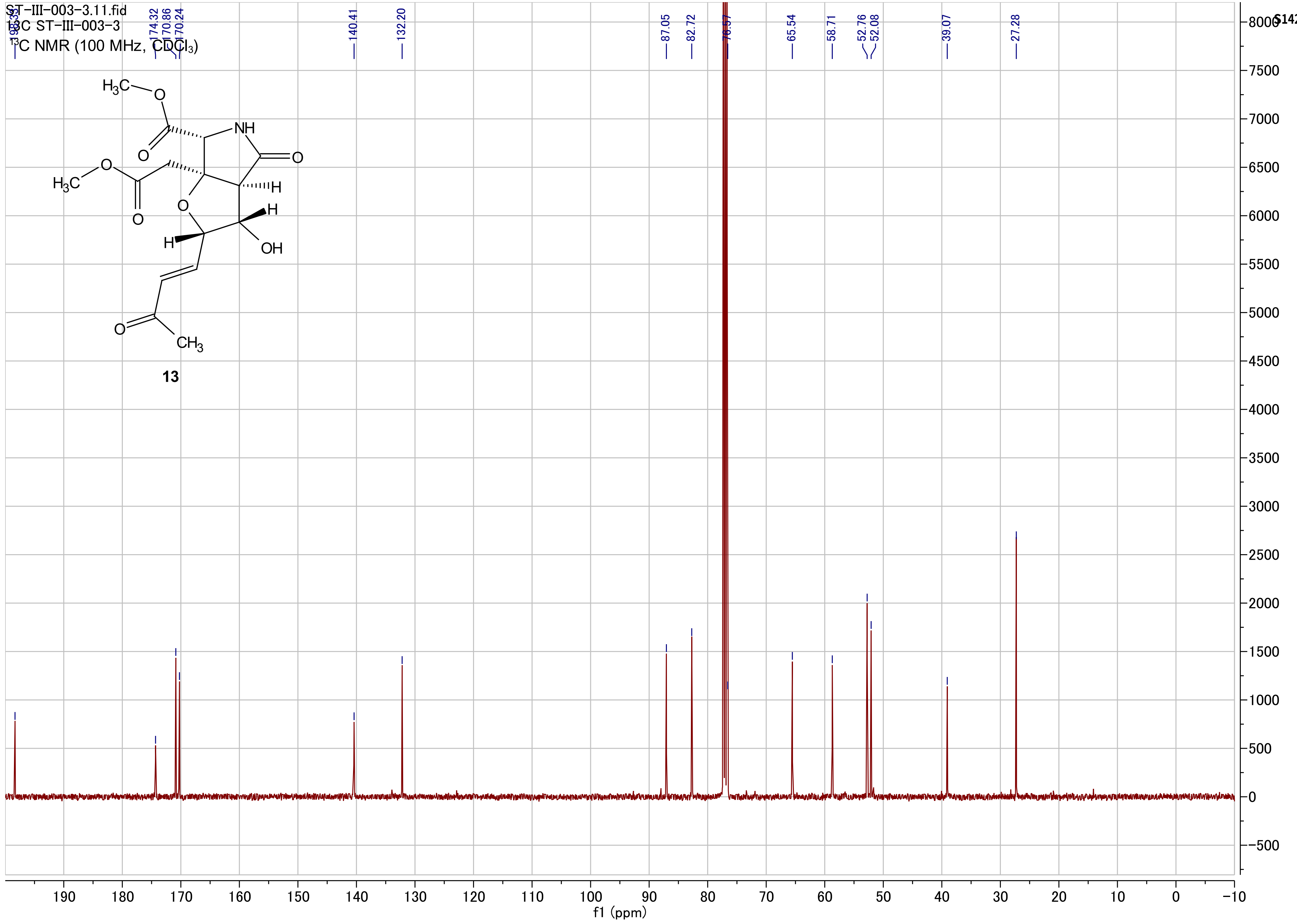

ST−III−003−3.11.fid
13C ST−III−003−3
13C NMR (100 MHz, CDCl3)
198.33
174.32
170.86
170.24
140.41
132.20
87.05
82.72
76.57
65.54
58.71
52.76
52.08
39.07
27.28
H3C
O
NH
O
O
H3C
O
H
O
H
H
OH
O
CH3
13
8000
7500
7000
6500
6000
5500
5000
4500
4000
3500
3000
2500
2000
1500
1000
500
0
−500
190
180
170
160
150
140
130
120
110
100
90
80
70
60
50
40
30
20
10
0
−10
f1 (ppm)

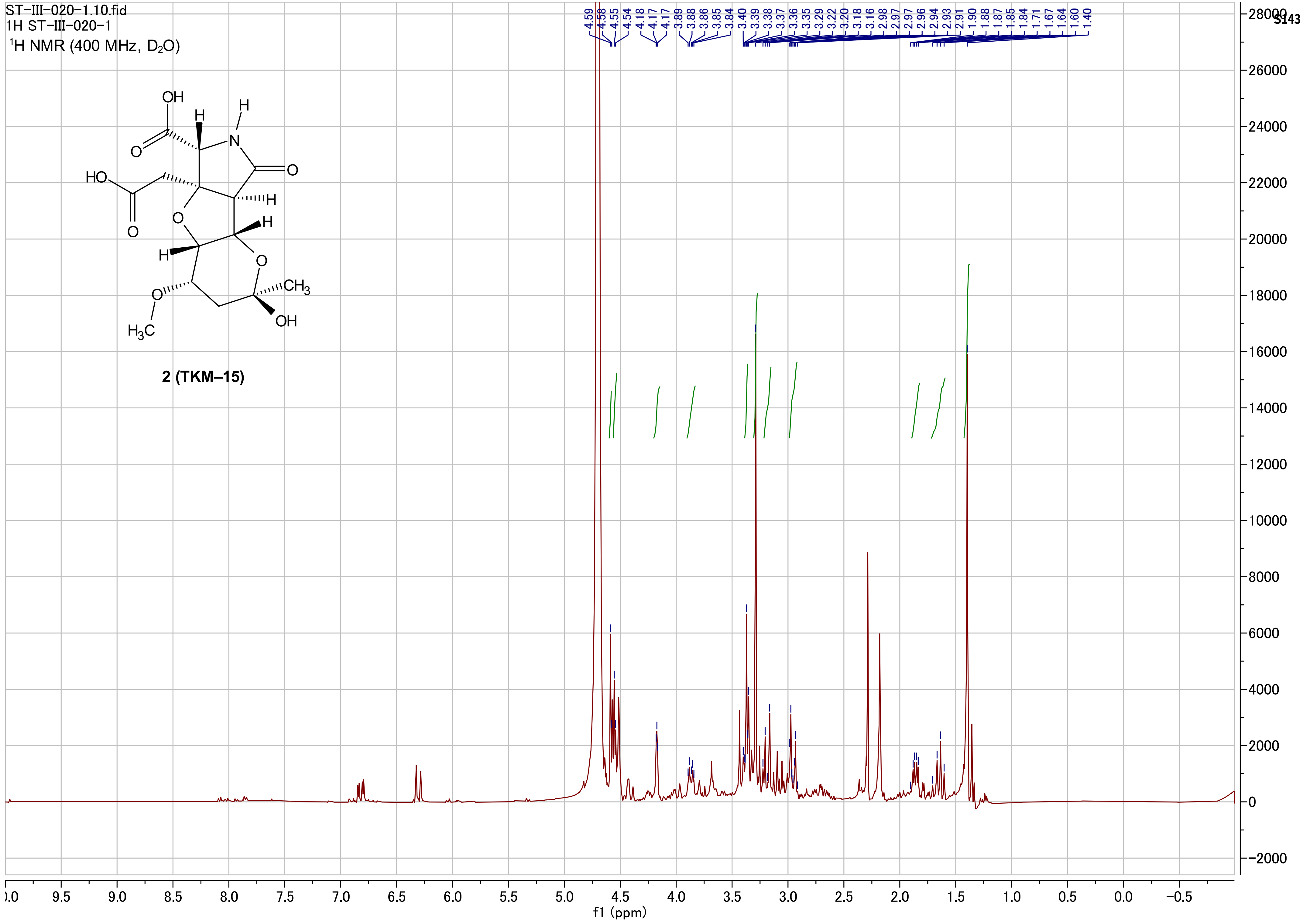
ST-III-020-1.10.fid
1H ST-III-020-1
^{1}H NMR (400 MHz, D_2O)
4.59 4.58 4.55 4.54 4.18 4.17 4.17 3.89 3.88 3.86 3.85 3.84 3.40 3.39 3.38 3.37 3.36 3.35 3.29 3.22 3.20 3.18 3.16 2.98 2.97 2.97 2.96 2.94 2.93 2.91 1.90 1.88 1.87 1.85 1.84 1.71 1.67 1.64 1.60 1.40
2 (TKM–15)
f1 (ppm)

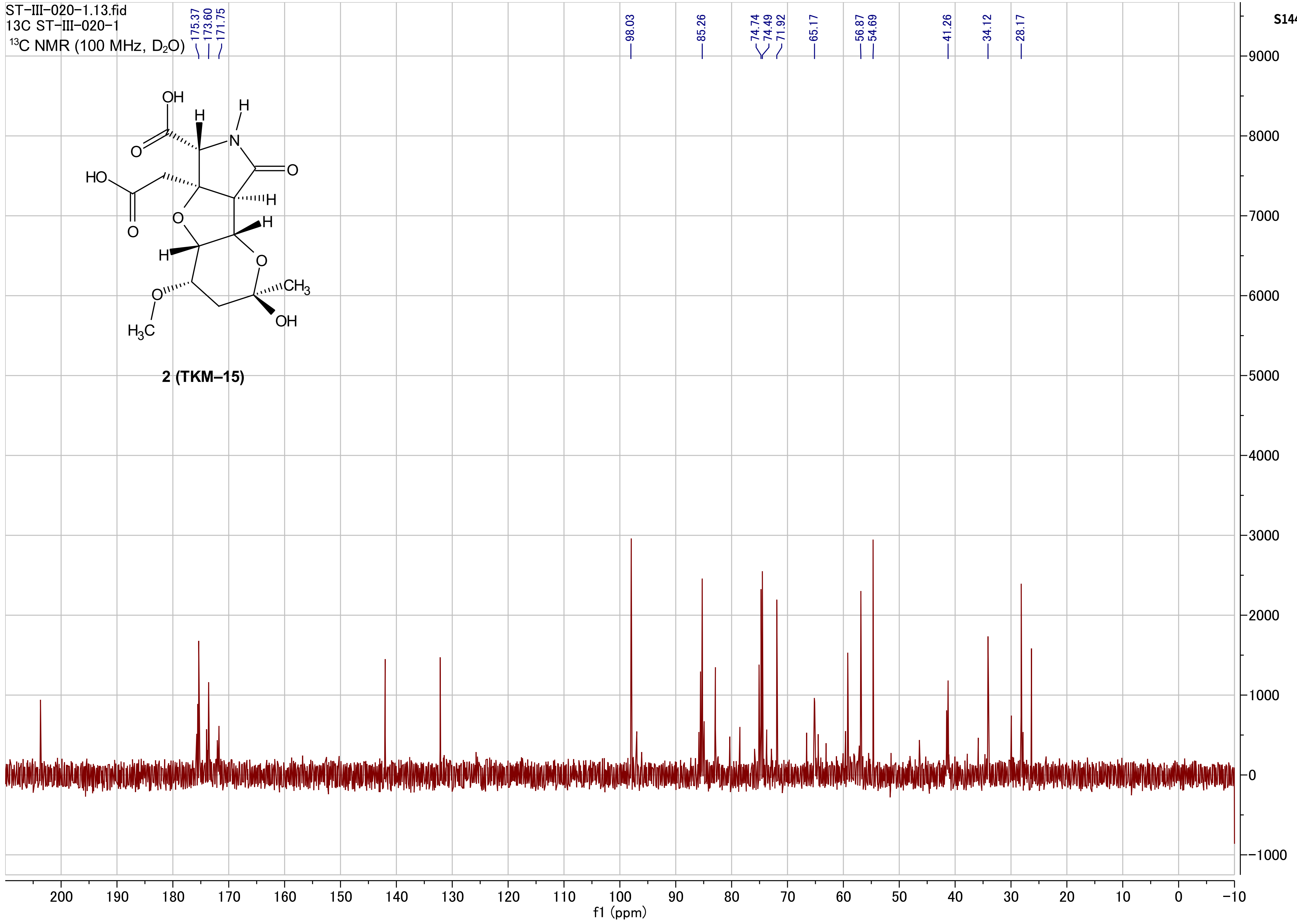
ST-III-020-1.13.fid
13C ST-III-020-1
13C NMR (100 MHz, D2O)
175.37
173.60
171.75
98.03
85.26
74.74
74.49
71.92
65.17
56.87
54.69
41.26
34.12
28.17
2 (TKM–15)
f1 (ppm)
